# An Appealing, Robust Access to Furo-Fused Heteropolycycles

**DOI:** 10.3390/molecules30040948

**Published:** 2025-02-18

**Authors:** Alice Benzi, Lara Bianchi, Gianluca Giorgi, Giovanni Lentini, Massimo Maccagno, Guglielmo Marcantoni Taddei, Giovanni Petrillo, Cinzia Tavani

**Affiliations:** 1 Department of Chemistry and Industrial Chemistry (DCCI), University of Genova, Via Dodecaneso 31, 16146 Genova, Italymassimo.maccagno@unige.it (M.M.); gu.marcantoni@gmail.com (G.M.T.); giovanni.petrillo@unige.it (G.P.); cinzia.tavani@unige.it (C.T.); 2 Department of Biotechnology, Chemistry and Pharmacy, University of Siena, Via A. Moro, 53100 Siena, Italy; gianluca.giorgi@unisi.it; 3 Department of Pharmacy-Pharmaceutical Sciences “Aldo Moro”, University of Bari, Via E. Orabona 4, 70125 Bari, Italy; giovanni.lentini@uniba.it

**Keywords:** nitrostilbenes, heteropolycycles, double coupling, cyclization, electrocyclization

## Abstract

Recently, nitrostilbenes characterized by two different or differently substituted aryl moieties, obtainable from the initial ring-opening of 3-nitrobenzo[*b*]thiophene with amines, have proved, by means of a stepwise double coupling with phenolic-type bidentate C/O nucleophiles, to be valuable precursors of oxygen-containing heteropolycycles and of fully conjugated systems therefrom via an efficient 6π-electrocyclization and final aromatization. Herein, the methodology is extended, after suitable optimization, to diverse heterophenols to afford new appealing heteropolycyclic systems of potential interest as drug leads. The synthetic results are fully consistent with up-to-date quantomechanical calculations. For some of the new molecules, a significant fluorescence is reported, with a potential for future applications, e.g., in the field of optical devices.

## 1. Introduction

**Figure 1:**
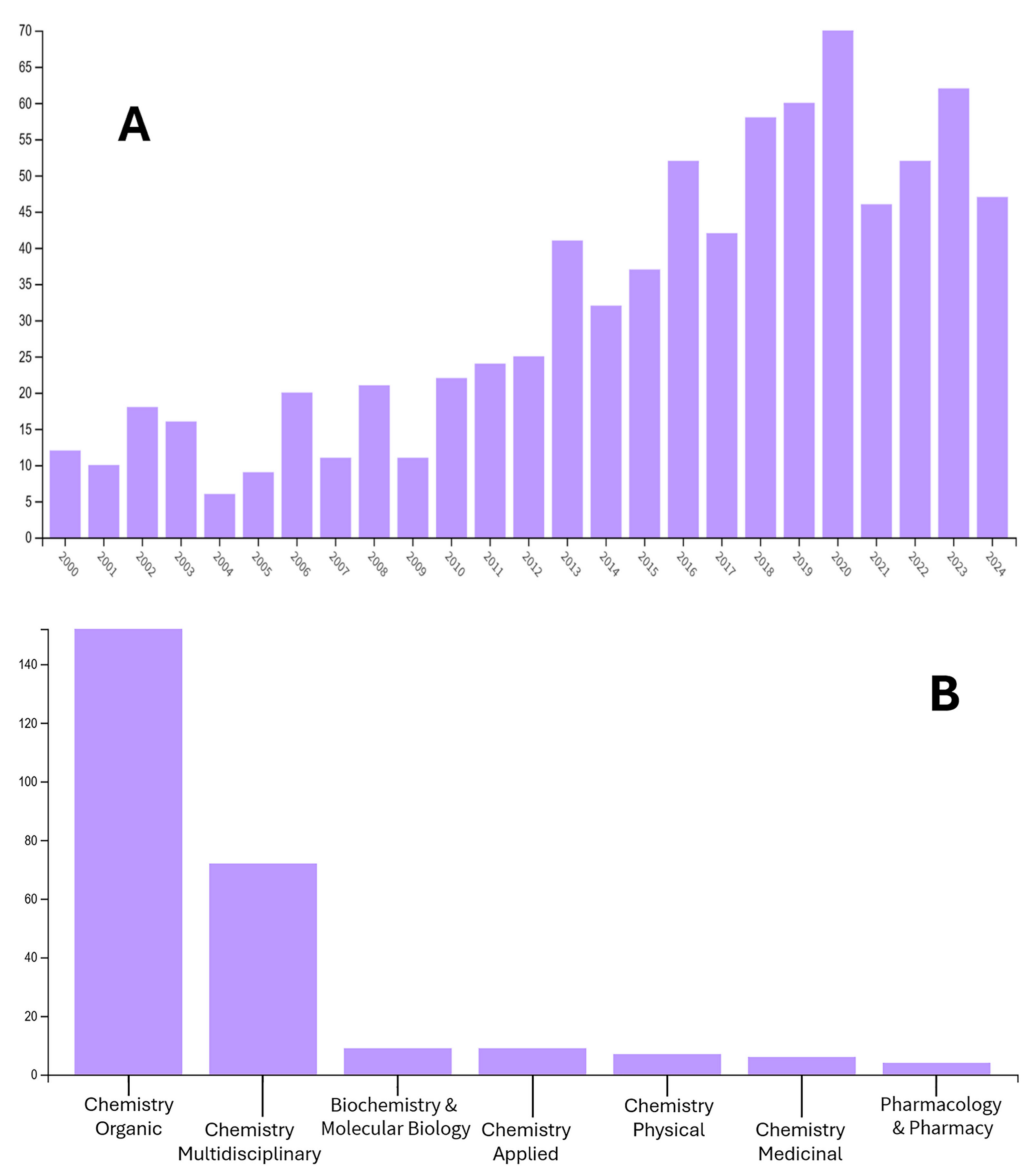
(**A**) Search for heteropolycycles or polyheterocycles in Web of Science: the graphic shows the evident growth of the number of publications (948 over 25 years, 60% in the last decade). (**B**) Limiting the field to fused compounds, the number decreases significantly (224, 24%), and the graphic describes the distribution according to the subject.

The recent literature witnesses an ever-growing number of publications concerning polycyclic systems characterized by the presence of one or more heteroatoms, testifying to the great interest of the scientific community in these compounds (Figure 1) [1]. Accordingly, new and/or more efficient synthetic approaches are desirable in order to further structurally and functionally diversify the final outcomes, including in the perspective of achieving new fields of application [2,3].

Among such an unbelievably wide class of compounds, aza-heterocycles are undoubtedly the most represented, often regarded as privileged structures [4,5]; they are also of outmost relevance from the biological/pharmacological point of view. However, many other rings are also raising great interest, and many other applicative fields can be cited, for instance, that of fluorescent compounds [6].

Within this field, we became interested in novel fused-heteropolycyclic structures, characterized by the presence of furan or dihydrofuran rings, as potential antioxidants imitating phenolic and polyphenolic natural compounds [7].

**Scheme 1:**
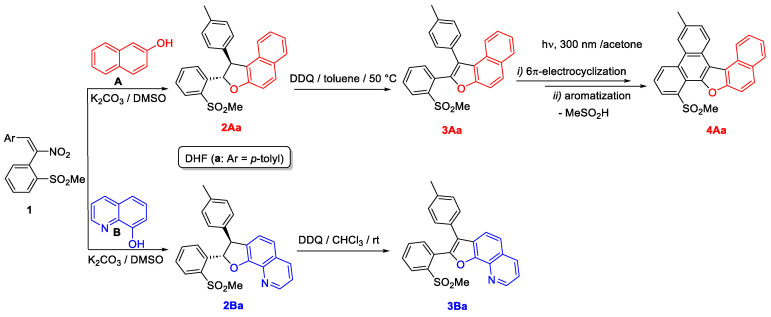
The protocol developed in our previous study [8] generating DHFs, furans, and electrocyclized compounds from 2-naphthol **A** (in red color) or 8-hydroxyquinoline **B** (in blue color). The *p*-tolyl derivative **1a** (Ar = *p*-tol: Figure 2) was chosen as the model compound.

Recently, in our laboratory, the reaction of a series of stilbenes **1** with 2-naphthol **A** (Scheme 1, red) or, for a more limited series of substrates, with 8-hydroxyquinoline **B** (Scheme 1, blue) in K_2_CO_3_/DMSO turned out to be an effective access to fused dihydrofurans (DHF, **2**) and furans (**3**), from which fully conjugated heteropolycyclic systems (**4**) could be generated by a 6π-electrocyclization followed by aromatization [8,9]. The final products exhibit interesting fluorescence properties, apart from potential pharmacological activity. An attempt to extend the reaction to 4-hydroxycoumarin **C** in the same experimental conditions met with failure, and the tentative use of different conditions (DABCO as an organic base in the less polar CHCl_3_) was likewise unsatisfactory [8].

**Figure 2:**
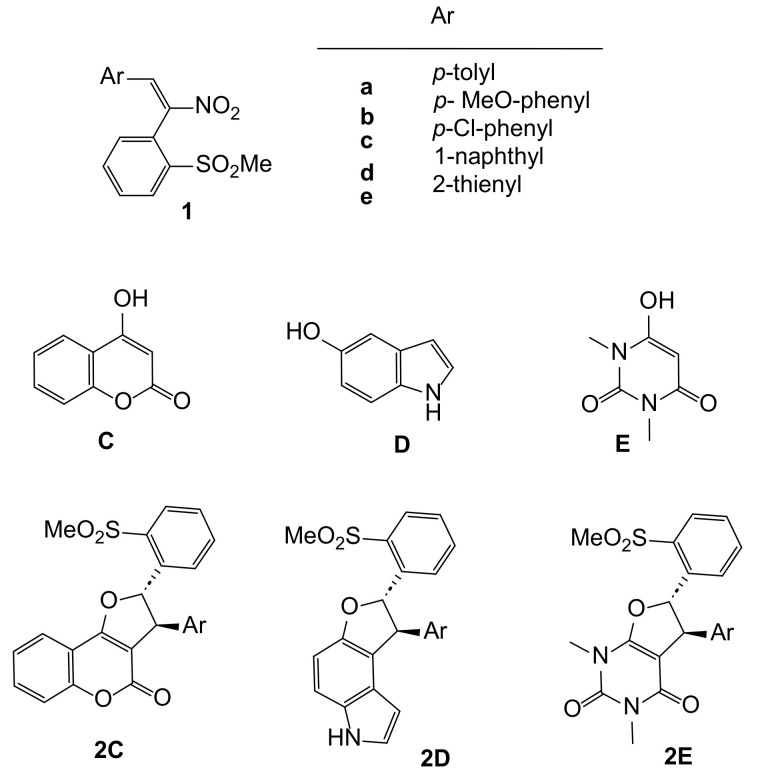
This work: new heterophenols to be tested (**above**) and the relevant structures (**below**) expected from an initial double coupling with nitrostilbenes **1**.

In order to improve the robustness and significance of our synthetic protocol, herein, we present results obtained under optimized experimental conditions from the reaction of nitrostilbenes **1a**–**e** with 8-hydroxyquinoline (**B**) (thus extending the substrate scope) and with some structurally different O- or N-heteroaromatic bidentate (C/O) nucleophiles (**C**, **D** and **E**: Figure 2 where the expected initial coupling products are also sketched) (thus extending the nucleophile scope).

## 2. Results and Discussion

### 2.1. Reactions of Nitrostilbenes 1 with 8-Hydroxyquinoline (***B***)

#### 2.1.1. Synthesis of Dihydrofurans **2B**

**Scheme 2:**
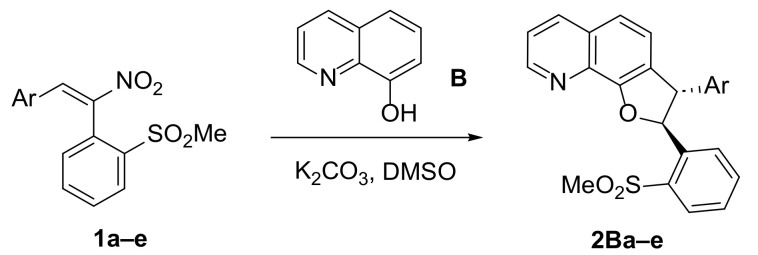
Synthesis of dihydrofuroquinolines **2Ba**–**e**.

Our first target was to extend the range of stilbenes generating dihydrofurans from 8-hydroxyquinoline **B** (Scheme 2) and, thus, increase the substrate scope of our double-coupling methodology. Table 1 collects the data obtained under the same conditions utilized with 2-naphthol **A** [8], i.e., 1 mol equiv. of **B** and 1.2 equiv. of K_2_CO_3_, in DMSO.

**Scheme 3:**
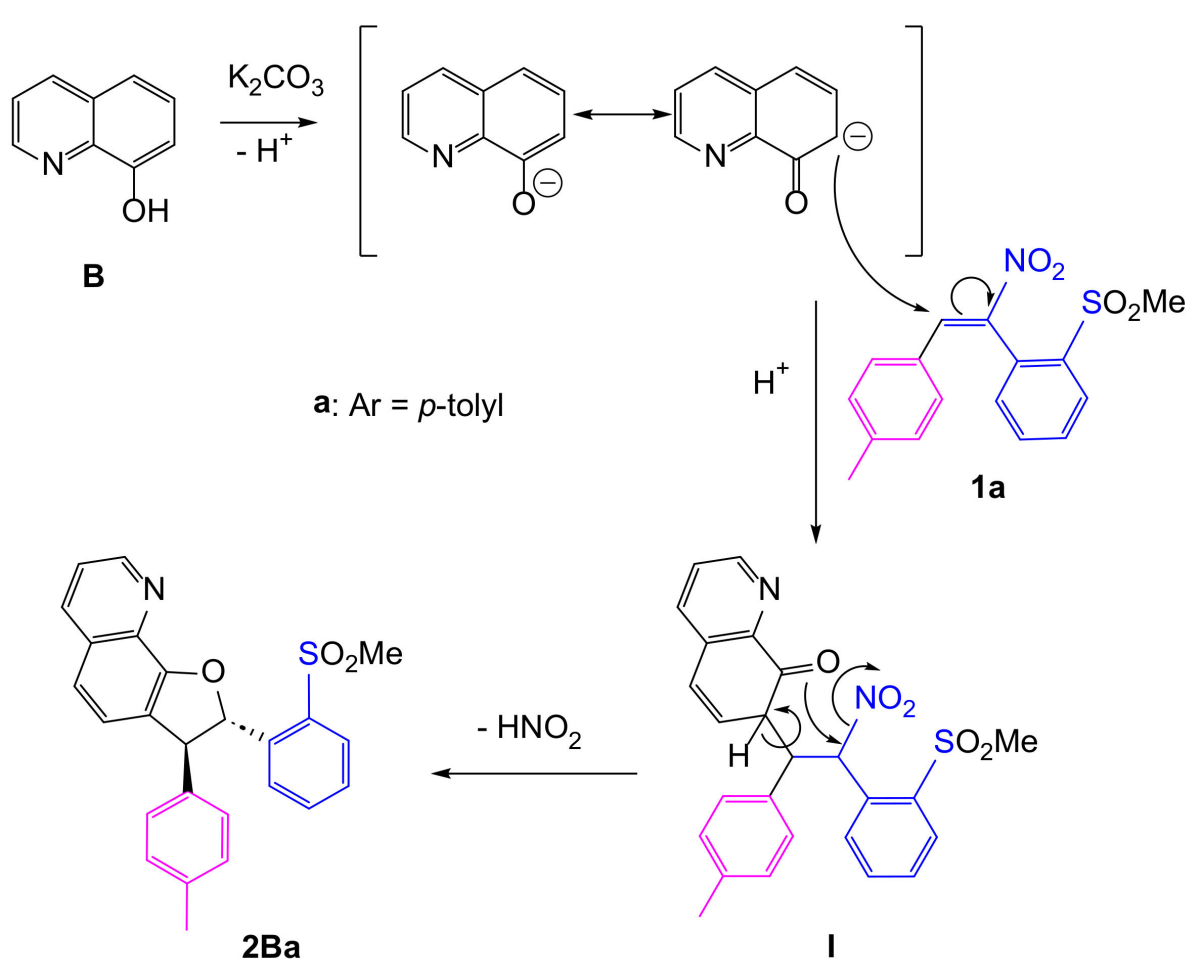
Proposed mechanism for the formation of DHFs from nitrostilbenes and **B**, also applicable to all of the bidentate (C/O) nucleophiles tested herein.

The initial formation of the dihydrofuroquinolines rests on a sequential double coupling between the twofold (C/C) electrophilic nitrostilbene and the twofold (O/C) nucleophilic phenolic derivative, as sketched in Scheme 3.

The proposed mechanism features an initial Michael-type coupling whereby the bidentate anion generated by deprotonation of the phenolic group behaves as a C-nucleophile toward the nitrovinyl moiety of **1**. Re-aromatization of the phenolic ring with intramolecular displacement of the nitro group, most likely assisted by protonation, completes the dihydrofuran ring formation, a mechanism that received definite support in our previous work by the X-ray structural determination of the dihydrofuro-fused derivative **2Aa** of Scheme 1 [8].

**Figure 3:**
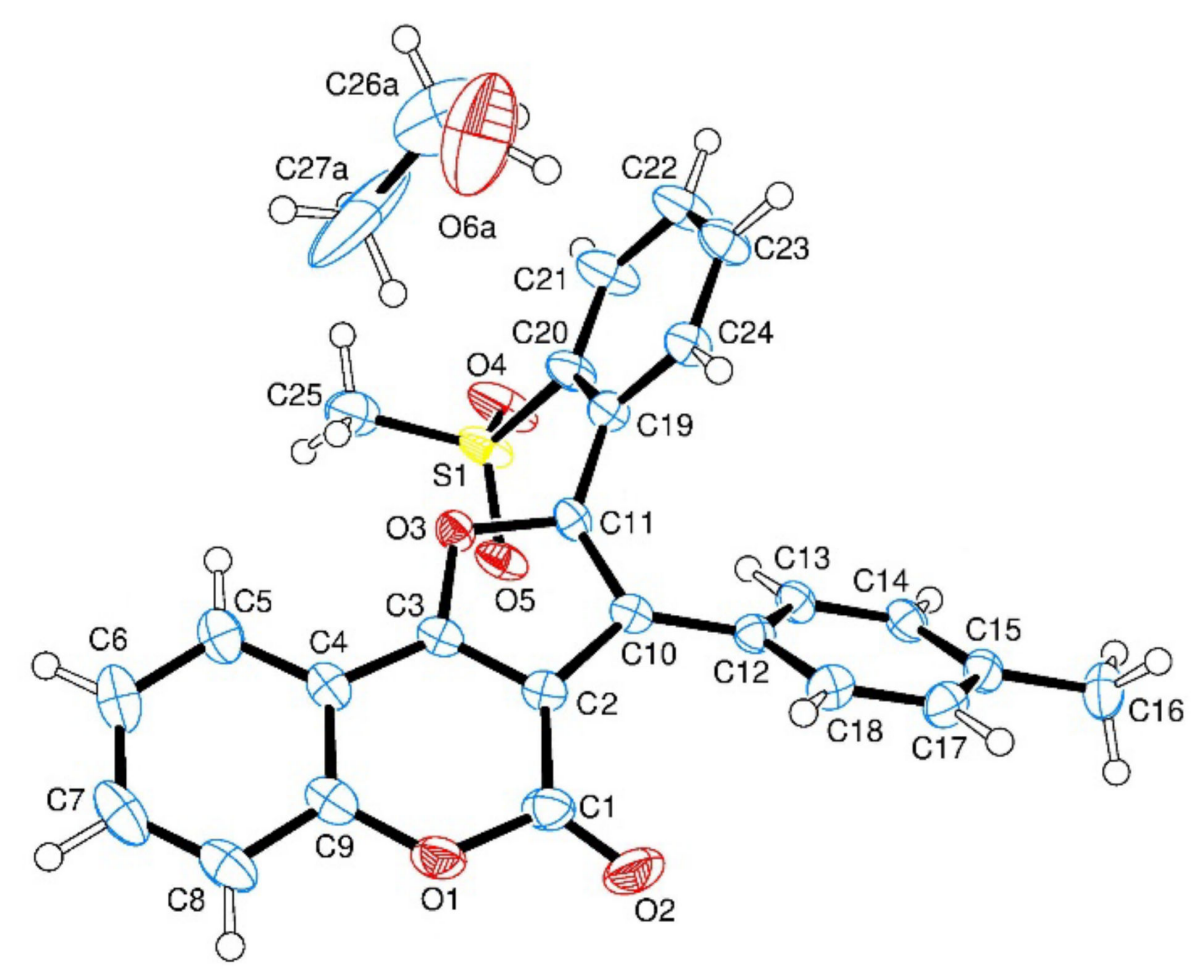
ORTEP obtained by X-ray analysis of the furocoumarin **3Ca** crystallized with a molecule of ethanol. Ellipsoids enclose 50% probability.

The same mechanism most likely holds for all the bidentate nucleophiles tested herein. Thus, as a further sample test, the structure of the furan-fused coumarin **3Ca** has been, in turn, confirmed by an X-ray analysis; the ORTEP reported in Figure 3 also shows the presence of a molecule of the solvent (ethanol) used for the crystallization.

As a matter of fact, C-C couplings of phenols or condensed phenols with electrophiles, such as typical Michael acceptors [10,11,12] as well as carbonyls [13] or nitroalkenes [14] (also envisaged as Friedel–Crafts aromatic substitutions), in some cases eventually leading to fused dihydrofurans [13,14], are well documented in the literature, both in basic [10,11] and in acidic conditions [12,13,14].

As shown by the data in Table 1, the reactions with **1d** and **1e** proved to be very slow, further leading to rather poor final yields. For the sake of completeness and in order to verify the stability of dihydrofurans with time, the reactions on **1a**–**c** were prolonged to 24 h (fourth column): the comparison with our previous data [8] (last column) did not unveil major differences in yields with time but some significant decrease for **2Ba**.

The rather unsatisfactory outcomes with **1d** and **1e** remain somehow surprising in the light of the efficient coupling of such nitrostilbenes experienced with 2-naphthol [8]: at the moment though, no sounding explanation can be provided (regarding **1e**, see hereinafter about the coupling with **C**: Section 2.2.1).

#### 2.1.2. Aromatization of the Dihydrofuroquinolines **2B** to **3B**

**Scheme 4:**
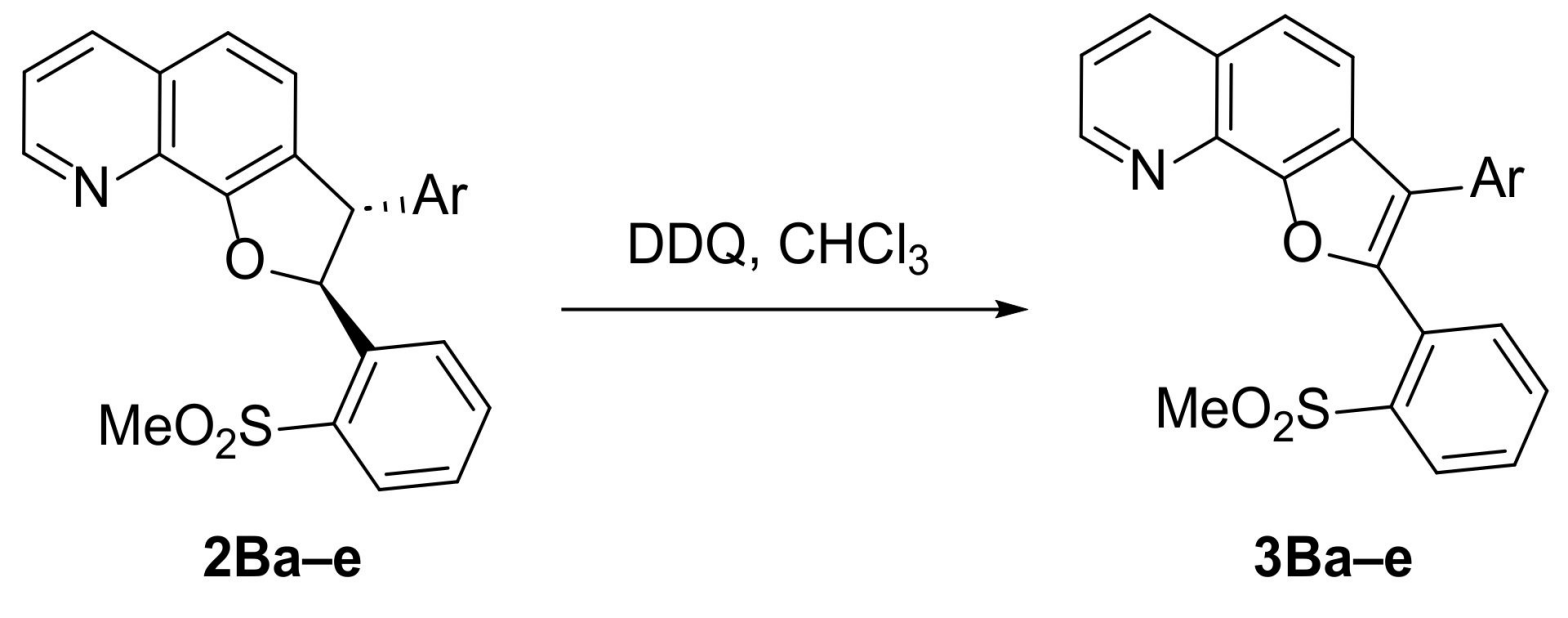
Aromatization of dihydrofuroquinolines **2Ba**–**e**.

The dihydrofurans **2Ba**–**e** were subjected to the oxidative conditions already used for naphthofurans [8] (Scheme 4), observing good to excellent yields of the desired aromatized **3Ba**–**e** (Table 2). The reaction of the naphthyl derivative was too slow at r.t. requiring a higher temperature.

### 2.2. Reactions of Nitrostilbenes 1 with 4-Hydroxycoumarin (***C***)

#### 2.2.1. Synthesis of Dihydrofurans **2C**

The double-coupling protocol was extended to 4-hydroxycoumarin (**C**), preceded by a literature search for suitable reaction conditions: this is because, as already recalled above, the reaction with the model nitrostilbene **1a** under the usual conditions (K_2_CO_3_/DMSO) failed to produce significant amounts of **2Ca**, while the DABCO/CHCl_3_ system led to a very poor yield (13%) [8]. On the other hand, the hydroxycoumarin moiety represents, in particular, a very promising privileged structure as it characterizes the biological activity of both natural and synthetic molecules [15,16,17,18,19,20], thus justifying further synthetic efforts aimed at functional and/or structural/geometrical diversification.

**Scheme 5:**
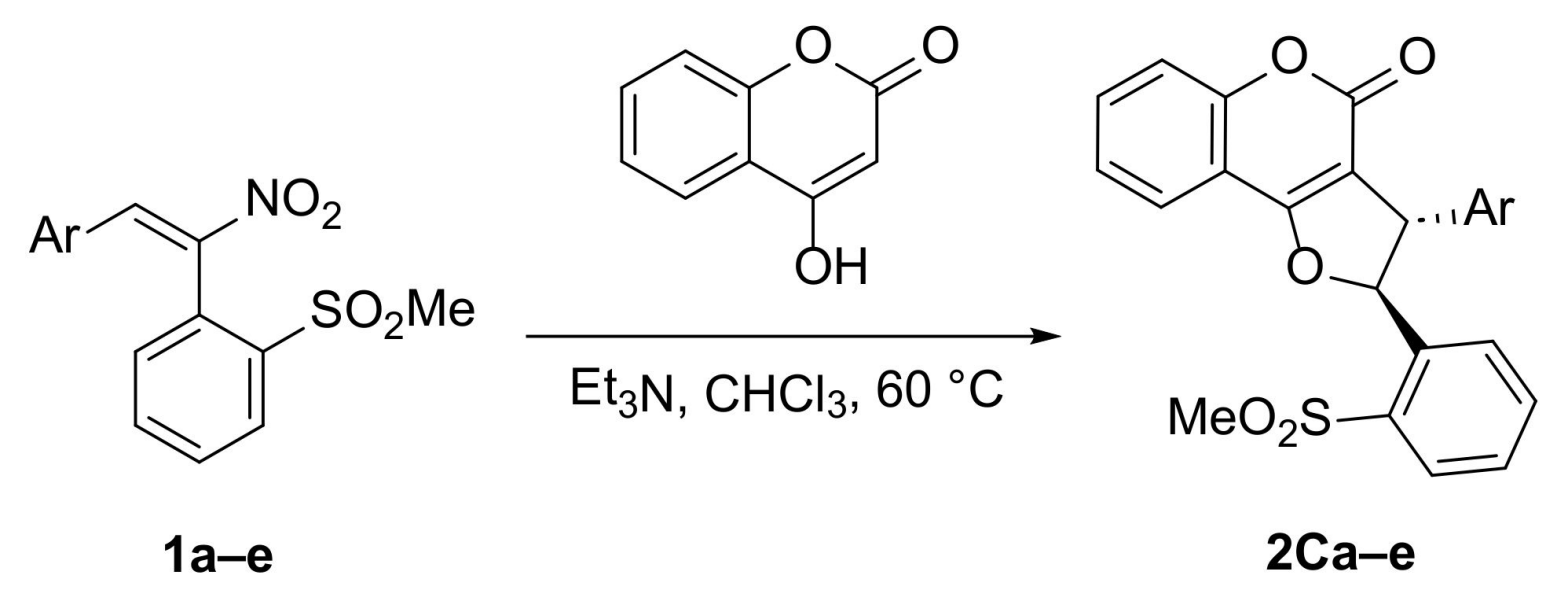
Synthesis of dihydrofurocoumarins **2Ca**–**e**.

Quite rewardingly, far better results were obtained when employing triethylamine (2 mol equiv.) at 60 °C in CHCl_3_ (Scheme 5 and Table 3). Surprisingly enough, in these conditions, **1d** (Ar = 1-naphthyl) lined up with **1a**–**c**, as observed with 2-naphthol **A** [8].

**Scheme 6:**
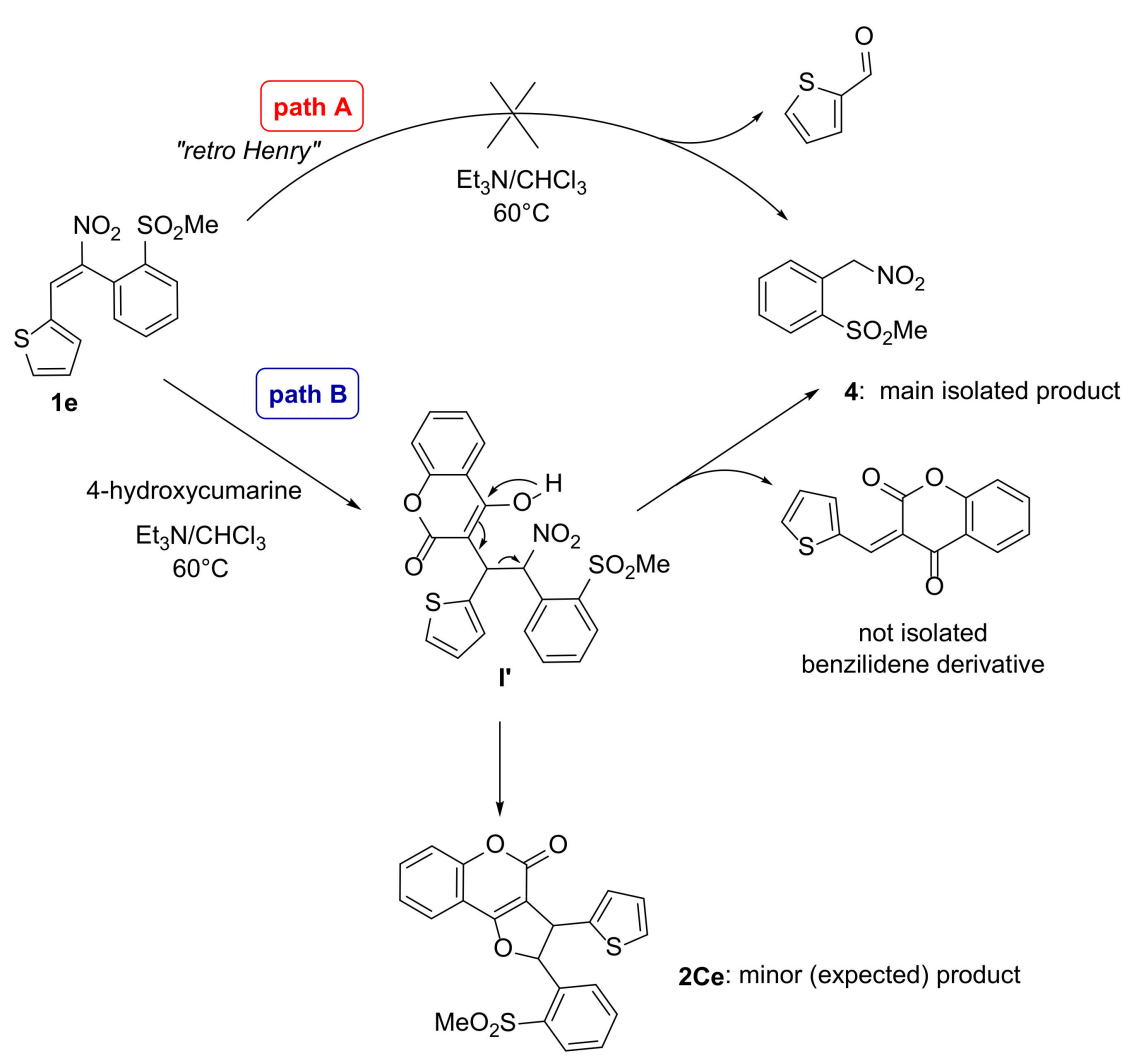
Possible rationalization of the formation of the nitrosulfone **4** in the reaction between **1e** and 4-hydroxycoumarin **C**.

On the other hand, with the 2-thienyl derivative **1e**, only minor amounts of the expected **2Ce** could be isolated. The presence, in the final reaction mixture (79%), of the nitrosulfone **4** (Scheme 6) could suggest the occurrence of a fragmentation of the nitrostilbene via a retro-Henry process [21,22] (Scheme 6, path A), sometimes observed in our laboratories in nitrobutadienes under basic conditions. Nonetheless, a control reaction on **1e** with Et_3_N in CHCl_3_ left the stilbene unchanged, so testifying that a retro-Henry cannot be responsible for the formation of **4**.

Another hypothesis was then considered whereby the main isolated product would derive from an initially formed Michael adduct **I′** (Scheme 6, path B) and then evolve along two competitive pathways. It should be noticed that from the chromatographic column, only **2Ce** and **4** were isolated, indicating the presence of some non-eluted, very polar, colored products.

To overcome the negative outcome above, a different set of experimental conditions was tested, namely, DABCO in EtOH at reflux [23], which, at last, allowed to isolate compound **2Ce** in a satisfactory 74% yield (Table 4). These conditions proved to be more advantageous from the practical point of view as the product precipitates and can be isolated as a pure white solid by filtration: accordingly, for the sake of comparison, the same conditions were also tested with substrates **1c**,**d**, with overall satisfactory results.

#### 2.2.2. Aromatization of the Dihydrofurocoumarins **2C** to **3C**

**Scheme 7:**
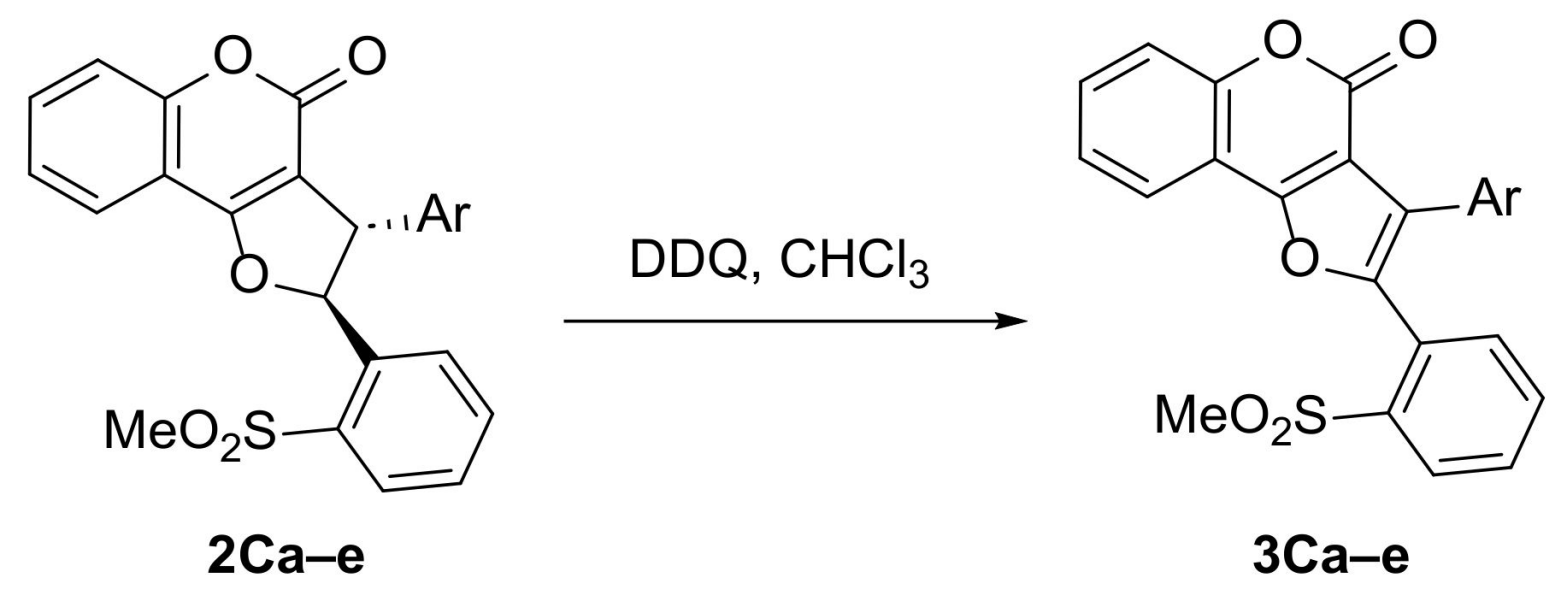
Aromatization of dihydrofurocoumarins **2Ca**–**e**.

The aromatization of **2C** to **3C** with DDQ (Scheme 7) required some optimization of the experimental conditions. With the model substrate **2Ca**, the best results were obtained by raising the number of DDQ molar equivalents (4), temperature (60 °C), and time (5 days) (Table 5). The use of toluene as solvent, which would allow for higher temperatures, gave a far poorer result.

The conditions optimized for **2Ca** were then extended to the other substrates (Table 6), but while **2Cb** and **2Ce** (electron-rich substrates) were easily oxidized, the two remaining DHFs either reacted very slowly (**2Cc**) or did not react at all (**2Cd**).

As to the former outcome, it should be kept in mind that the presence of the electron-withdrawing *p*-Cl substituent would make the substrate electron-poor and, thus, reluctant to oxidation. Please note that an evaluation of the conversion was possible only by ^1^H NMR analysis because any attempt of chromatographic separation failed: **2Cc** and **3Cc** had the same Rf with all the eluents tested. Furthermore, the fluorescence of **3Cc** hid the spot of **2Cc**.

As to the complete failure of the naphthyl derivative **2Cd** to undergo oxidation, it should be stressed that none of many other oxidants tested for its aromatization met with success: I_2_ and DBU in DCM at rt, 24 h [24]; MnO_2_ in DCM at 40 °C, 24 h [25]; PIDA/AcOH, 110 °C, 48 h [26,27]; TEMPO/FeCl_3_/NaNO_2_/O_2_/MeCN/rt/3 h [28].

**Scheme 8:**
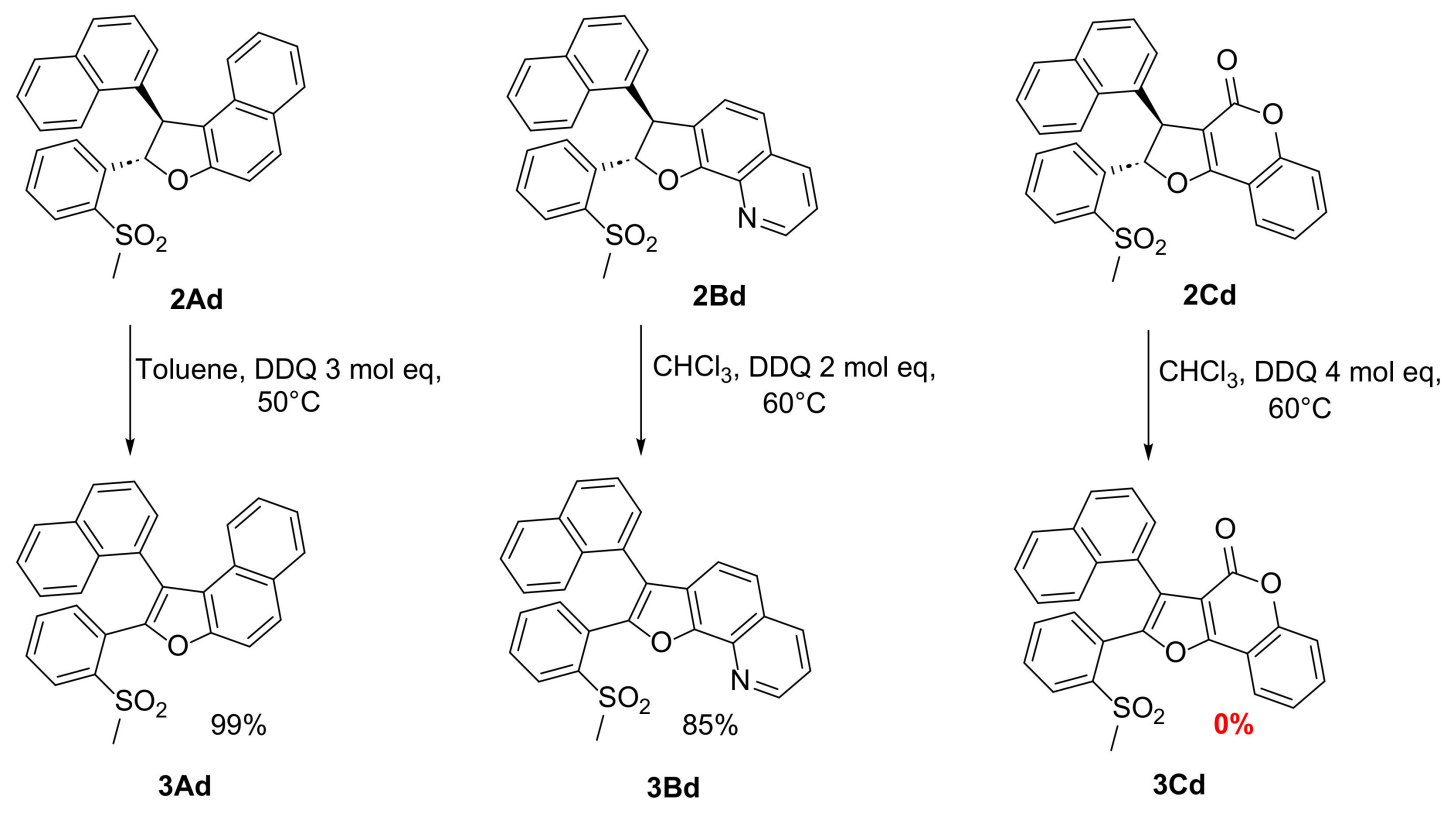
Comparison of aromatization yields for **2Ad** (from ref. [4]), **2Bd**, and **2Cd** in similar experimental conditions.

A tentative qualitative explanation for such a failure could refer to a difficulty of **3Cd** to reach coplanarity of all of its aromatic rings, thus gaining little or no stabilization from an extended conjugation. Anyway, under such a perspective, the result remains very disappointing and somehow unexpected when compared to the results for the corresponding naphthofuran (**2Ad**) [8] and furoquinoline **2Bd** (Scheme 8).

To gain insight into this outcome, we conducted comparative quantomechanical calculations on **2Cd** and the easily oxidized **2Ad** and **2Bd** in order to envisage any stereoelectronic feature possibly related to the observed differences in reactivity.

First, a conformational search procedure was performed on their models (DFT:B3LYP/6-31G*//DFT:B97M-V/6-311+G(2df,2p)[6-311G*]), obtaining all possible local minimum conformations within a 5 kcal/mol window above the global minimum. The conformational space of **2Ad** is more populated (five conformers), while those of **2Bd** and **2Cd** are equally populated (2 conformers).

**Figure 4:**
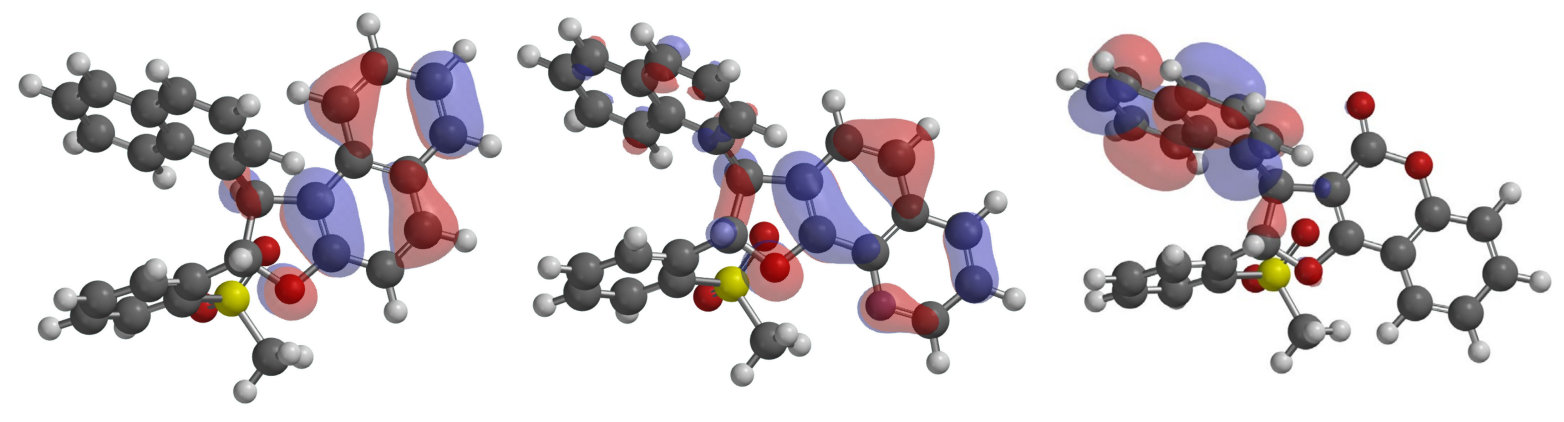

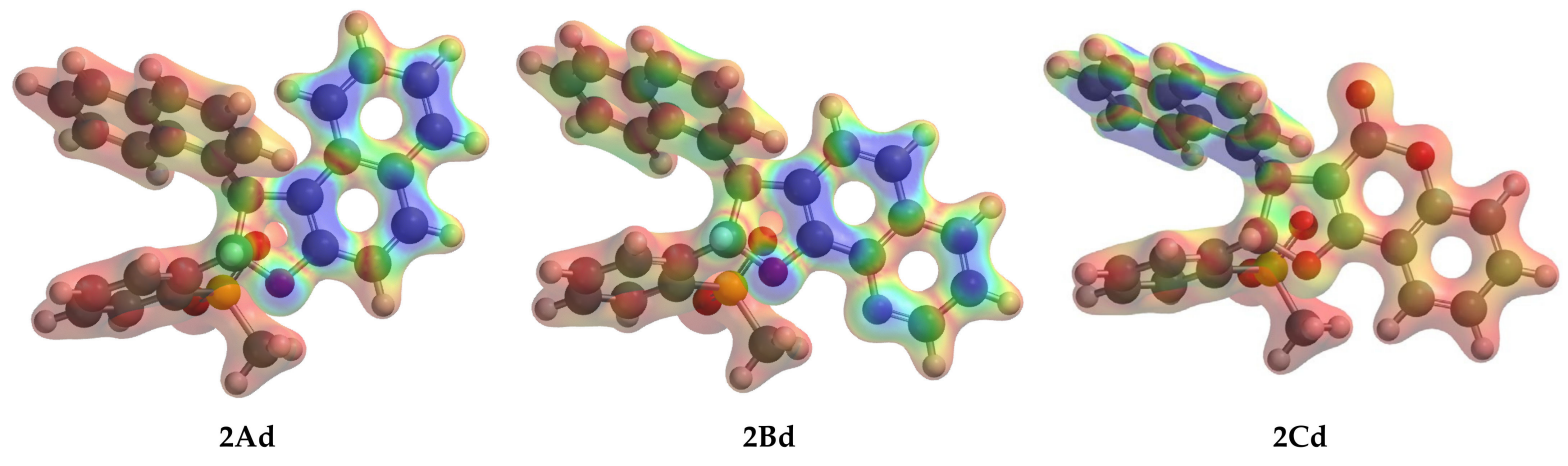
Comparison of the computed HOMO density maps for the most stable conformers of **2Ad**, **2Bd**, and **2Cd**: HOMO orbital distribution (**first row**) and HOMO mapped over the bond electronic density distribution (**second row**).

Subsequently, we calculated the HOMO density map over the sigma bond skeleton (DFT:ωB97X-V/6-311+G**//DFT:ωB97X-V/6-311+G(2df,2p)[6-311G*]) for the most stable conformers of **2Ad**, **2Bd** and **2Cd** (Figure 4). The HOMO is uniformly spread over the tricyclic plane of naphthofuran in **2Ad** and furoquinoline in **2Bd**, while in the furocoumarin **2Cd**, the largest contribution to the HOMO comes from the naphthalene moiety, with minimal contribution from the tricyclic system. Consequently, for the latter, electron movement can hardly occur in the region where aromatization is aimed.

This peculiar HOMO distribution appears to be the primary factor hampering the aromatization of **2Cd**, while hindering steric factors (suggested by the reduced conformational space) might create obstacles in the spatial arrangement of the substituents both in **2Cd** and **2Bd**, preventing optimal conjugation and limiting the effectiveness of aromatization of the furan ring, thus contributing to the unsuccessful aromatization of **2Cd** and reducing the aromatization yield of **2Bd**.

Encouraged by these intriguing results, we extended the same calculations to **2Ca**, **2Cb**, **2Cc**, and **2C** in order to determine whether a similar rationale could also explain the reactivity of the other **2C** compounds (as shown in Table 6), in particular, the relatively modest aromatization tendency of the *p*-Cl-phenyl-derivative **2Cc** compared to the much higher efficiency of the process on its congeners.

The conformational space of **2Cc** (2 conformers within a 5 kcal/mol window above the global minimum) is less populated than those of **2Ca** (5 conformers), **2Cb** (4 conformers), and **2Ce** (4 conformers), and equally populated as that of **2Cd**. This suggests that hindering steric factors may play a role in the less efficient aromatization of **2Cc**.

**Figure 5:**
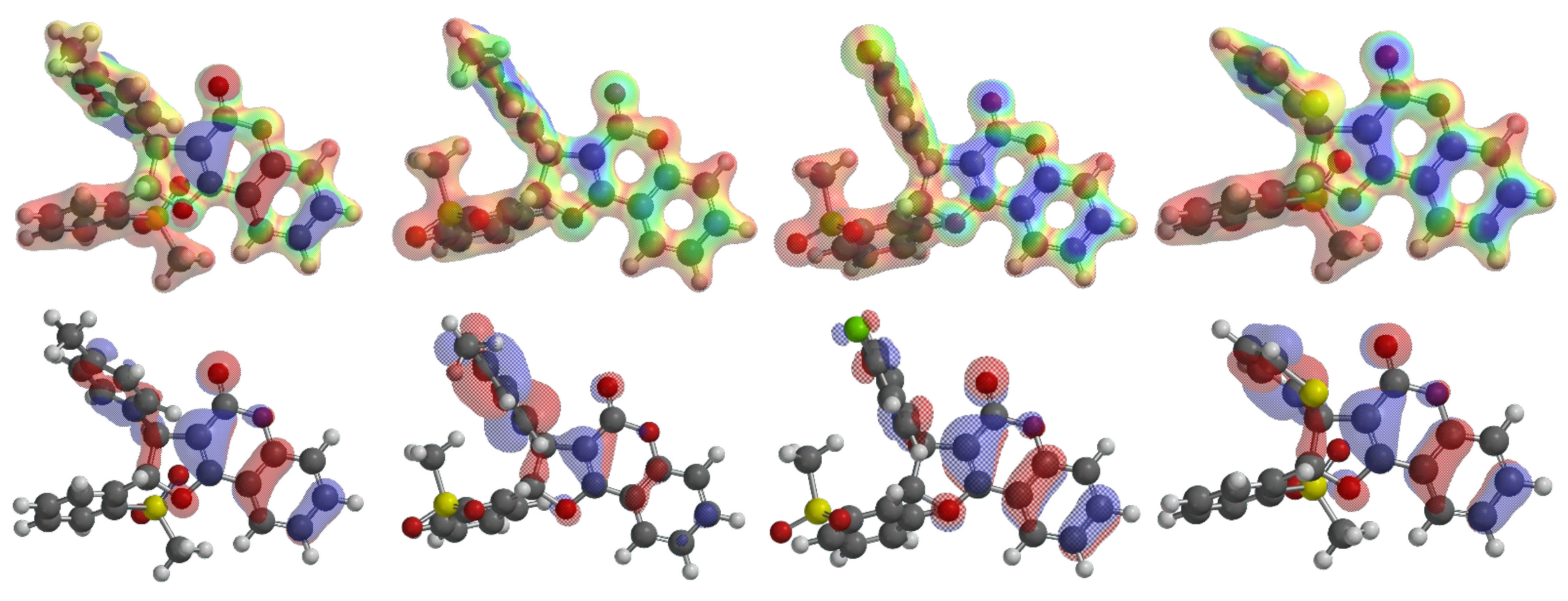

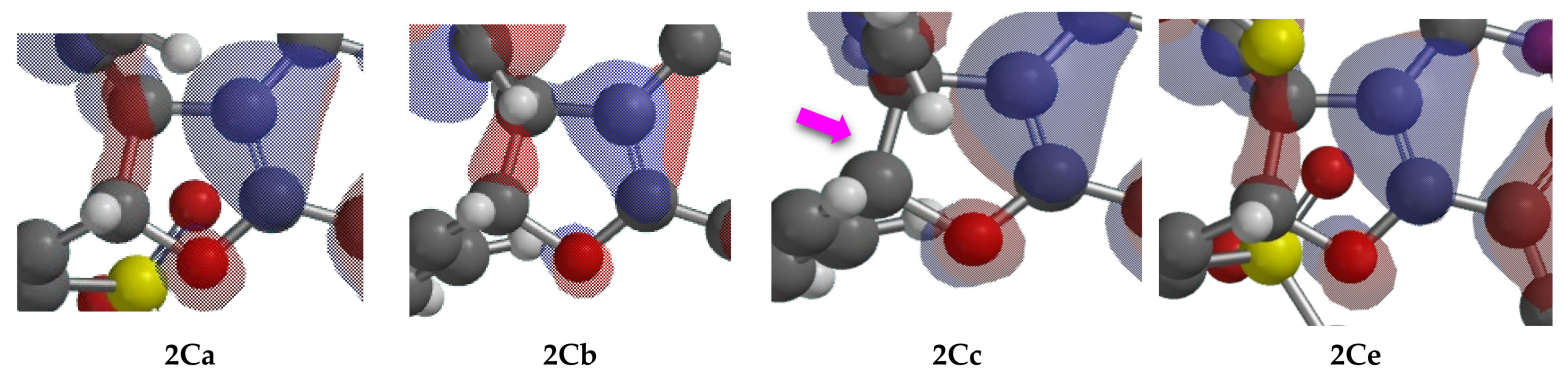
Comparison of the computed HOMO density maps for the most stable conformers of **2Ca**, **2Cb**, **2Cc**, and **2Ce**: HOMO mapped over the bond electronic density distribution (**first row**), HOMO orbital distribution (**second row**) and its zoomed view centered on the furan ring (**third row**).

Additionally, the HOMO density map for all these compounds spreads over both the tricyclic furocoumarin system and the 3-aryl moiety (though not uniformly); interestingly enough, only in the case of **2Cc**, it does not interest the region of the [4,5]-furan bond (Figure 5 (last row)), which is the actual site of the aimed aromatization.

Thus, both steric and electronic factors likely contribute to explaining the experimental outcomes for the aromatization of **2C** to **3C**.

### 2.3. Extension of the Coupling Methodology with Nitrostilbenes 1 to Other Heterocyclic Phenols: 5-Hydroxyindole (***D***) and N,N′-Dimethylbarbituric Acid (***E***)

On the grounds of the rewarding results obtained with **A**, **B**, and **C**, the coupling reaction with nitrostilbenes **1** was applied to other phenols in order to build up new heteropolycyclic systems, especially those containing nitrogen atoms.

#### 2.3.1. Synthesis of Dihydrofurans **2D** and of the Relevant Aromatized Furans **3D**

**Scheme 9:**
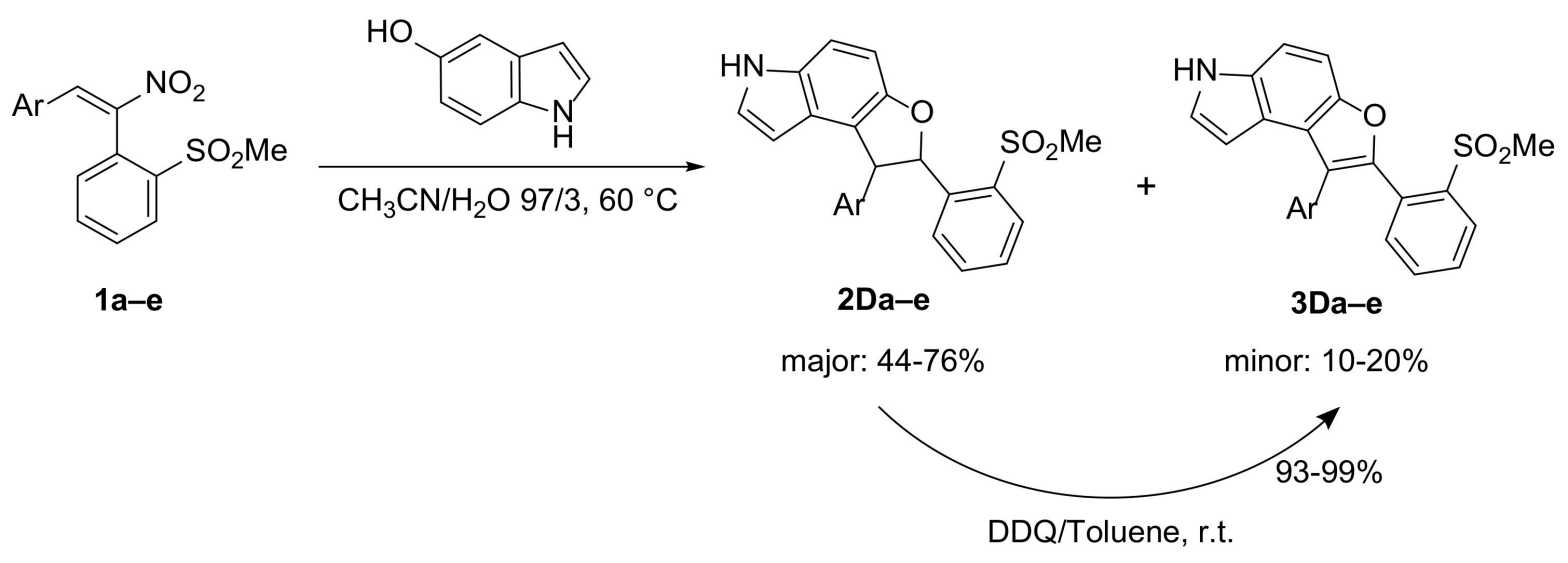
Synthesis of **2D** and **3D** from the initial coupling of **1a**–**e** and **D**.

Conditions and results of the coupling reaction between **1a**–**e** and **D** are reported in Table 7. Surprisingly enough, the expected dihydrofurans were always isolated in a mixture with minor quantities of the corresponding aromatized furan derivatives. The aromatization was easily completed by oxidation with DDQ (Table 7 and Scheme 9).

#### 2.3.2. Synthesis of Dihydrofurans **2E** and of the Relevant Aromatized Furans **3E**

Conditions and results of the coupling reaction between **1a**–**d** and **E** are reported in Table 8.

The aromatization to furan derivatives **3E** has been accomplished on model **2Eb** in order to verify the feasibility of the oxidative step.

### 2.4. 6π-Electrocyclization, Followed by Aromatization, of Furoquinolines ***3Ba***–***e*** and Furocoumarins ***3Ca***,***b***,***e***

Driven by the ever-growing interest of researchers in fluorescence, e.g., for the OLED technology [29], and encouraged by the fluorescence already displayed by both DHFs (**2Ba**) and furans (**3Ba**) in the quinoline series [8], we aimed to build up more extended systems of fully conjugated π-electrons, which would, hopefully, increase fluorescence properties through a 6π-electrocyclization followed by complete aromatization of the condensed furan derivatives described in the previous paragraphs, by applying the methodology proven quite satisfactory for naphtho-fused analogs [8], viz. photostimulation in acetone.

**Scheme 11:**
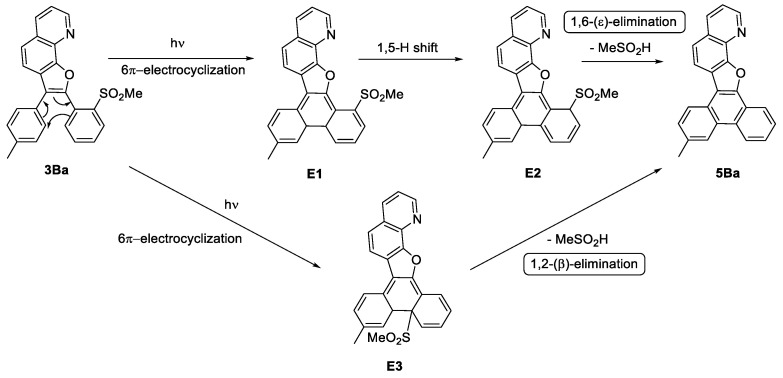
The electrocyclization process on **3B**, followed by 1,5-H-shift and 1,6-sulfinic acid elimination, produces the corresponding fully conjugated heteropolycycles **5B**.

Initially, acetone solutions of **3Ba**–**c** were sealed in a quartz tube and placed in a Rayonet photochemical reactor equipped with 300 nm lamps. Not surprisingly, we never observed the cyclohexadienes expected from the electrocyclization process, which, instead, proceeded to aromatization to **5Ba**–**c** thanks to an easy elimination, under the same conditions, of methanesulfinic acid from **E1** after a 1,5-H-shift to **E2** (Table 9 and Scheme 11 exemplify the process on **3Ba**). On steric grounds, we disfavor the electrocyclization to **E3**.

We observed, however, generally rather modest yields (Table 9, column 5), especially for the electron-rich **3Bb**, possibly due to the partial decomposition of the products by the heat developed. A successful attempt to improve the yields was the use of less energetic radiation (increasing the wavelength λ from 300 to 350 nm). Moreover, we hypothesized that the introduction of a base (DBU) in the system could make the acid elimination faster, thus exposing the products for a shorter time to potentially degrading conditions. Actually, the addition of 1 mol equiv. of DBU made the reactions faster and yields higher (Table 9, columns 7 and 8).

The modified conditions were then applied to substrates **3C** to obtain **5C** again in satisfactory yields (Table 10). From the practical point of view, it is noteworthy that the product **5C** precipitated completely in the reaction solvent, greatly simplifying the work-up; as far as obtaining a pure solid, it was sufficient to wash the precipitate with cold acetone until the solvent became colorless.

### 2.5. Fluorescence Analysis

One goal of the present protocol was to generate molecules endowed with significant fluorescence properties: as already recalled, for quinoline derivatives fluorescence appears in DHF, furans, and electrocyclized products; for coumarins, instead, only furans and electrocyclized derivatives are fluorescent [30], probably thanks to more extended fully conjugated systems.

**Figure 6:**
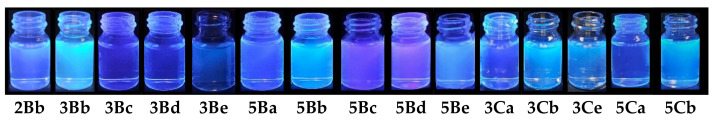
Ethanolic solutions of selected compounds under the UV lamp.

A qualitative visualization of fluorescence under UV irradiation is shown in Figure 6. Quantitatively, the most important fluorescence parameters are the Stokes shift and the quantum yield, calculated as described in the following Equations (1) and (2), respectively [31].

(1) $$\Delta \bar{\nu }={\bar{\nu }}_{ab}-{\bar{\nu }}_{em}=\frac{10^{7}}{\lambda_{{max}_{ab}}}-\frac{10^{7}}{\lambda_{{max}_{em}}}$$

**Equation** (**1**). The formula used to determine the Stokes shift ($\Delta \bar{\nu }$) where ${\bar{\nu }}_{ab},\;{\bar{\nu }}_{em}$ and $\lambda_{{max}_{ab}}$, $\lambda_{{max}_{em}}$ represent, respectively, the wave numbers (in nm) and the wavelengths (in cm^−1^) of the maximum absorption and emission.

(2) $$\phi_{X}=\phi_{R}\;\frac{1-10^{-A_{R}}}{1-10^{-A_{X}}}\;\;\frac{S_{X}}{S_{R}}\;\frac{\mu_{X}^{2}}{\mu_{R}^{2}}$$

**Equation** (**2**). The formula used to calculate the Quantum Yield (*Φ*) where A represents the absorbance at the wavelength of excitation, S is the area of the surface subtended in the spectrum along the interval of the *λ_em_*, and μ is the solvent refraction index; “*X*” refers to the analyzed sample, and “*R*” refers to the standard.

Table 11 collects the data of the compounds analyzed, allowing some comparative considerations among quinoline (green) and coumarin (blue) derivatives.

With regard to the quinoline derivatives, the higher quantum yields are shown, as expected, by the electrocyclized and fully aromatized **5B** compounds, which were prepared on purpose to obtain a more rigid planar system and extended conjugation; among them, the best performer is the 1-naphthyl-derivative **5Bd** due to the presence of the 1-naphthyl ring (as confirmed by the fact that the corresponding 1-naphthyl-substituted **3Bd** is the furoquinoline with the highest quantum yield).

Considering coumarin derivatives, they show generally lower quantum yields, which are rather good for the electrocyclized **5Ca**,**e;** unluckily, the **5Cd** naphthyl-derivative (which would probably show the highest quantum yield of the series) could not be obtained (as previously discussed).

## 3. Experimental Section

### 3.1. Materials and Methods

^1^H NMR and ^13^C NMR spectra were recorded with a JEOL JNM-ECZR 400 spectrometer (Akishima, Japan) at 400 and 100 MHz, respectively; chemical shifts (TMS as internal reference) are reported as δ values (ppm). Signals are designated as follows: s, singlet; d, doublet; dd, doublet of doublets; ddd, doublet of doublet of doublets; t, triplet; tt, triplet of triplets; m, multiplet; br, broad. High-resolution mass spectra (HRMS) were obtained with an Agilent MSD TOF mass spectrometer (Santa Clara, CA, USA) and recorded in positive ion mode with an electrospray (ESI) source. Melting points were determined with a Büchi 535 apparatus (Uster, Switzerland) and are uncorrected. Petroleum ether and light petroleum refer to the fractions with bp 40–60 °C and 80–100 °C, respectively. Silica gel 230–400 mesh or neutral aluminum oxide Acros Organics (Geel, Belgium, 50–200 μm), were used for column chromatography. TLC analyses were performed on commercially prepared 60 F254 silica gel plates (VWR, Radnor, PA, USA) and visualized by UV irradiation (eluant: petroleum ether—ethyl acetate). All commercially available reagents were used as received.

As already reported [32,33], the substrates employed (**1a**–**e**) were obtained starting from 3-nitrobenzo-thiophene, herein prepared in higher yields according to a literature procedure [34,35], different from the one usually employed [36,37].

Absorption spectra were recorded using a Perkin Elmer Lambda9 UV/VIS/NIR Spectrophotometer (Waltham, MA, USA), while fluorescence spectra were recorded (at the excitation wavelengths of 254 nm and 440 nm) with the Perkin Elmer MPF-44A Fluorescence Spectrophotometer and were normalized using Rhodamine B in PMMA as the reference standard. Compounds were analyzed as 100 µmol ethanolic solutions. Calculations were made with software the “LabPlot” (v. 2) software.

### 3.2. Quantum Mechanical Calculations

The models for compounds **2Ad**, **2Bd**, **2Ca**, **2Cb**, **2Cc**, **2Cd**, and **2Ce** were generated from atomic fragments incorporated into the internal fragment library of the computational software *Spartan’24* (v. 1.2.0, Wavefunction Inc. Irvine, CA, USA) starting from the suggested geometries [38] These models were optimized using the systematic/MonteCarlo method with the MMFF94 force field.

A systematic conformational distribution analysis was then performed, following the steps below:

(1) Up to 200 conformers within a 40 Kcal/mol energy window above the global minimum conformer were initially selected for further geometry optimization in the gas phase using ab initio Hartree–Fock calculations at the 3-21G level.
(2) Up to 100 conformers in a 20 Kcal/mol energy window were selected and further optimized using density-functional theory (DFT) implemented with ωB97X-D density functional and 6-31G* basis set.
(3) Up to 50 conformers in a 10 Kcal/mol energy window were selected and further refined by DFT calculations with the ωB97X-V functional and the 6-311+G(2df,2p)[6-311G*] basis set.
(4) Up to 25 conformers in a 5 Kcal/mol window were selected and their optimized structures were confirmed as real minima by IR frequency calculation (no imaginary frequencies).

The HOMO density distributions were calculated at the DFT ωB97X-V/6-311+G(2df,2p)[6-311G*] level on the DFT:ωB97X-V/6-311+G** most stable conformers (i.e., global minimum conformers) of each compound.

The HOMO density distributions were also calculated (in the same way) for the second most stable conformer of each compound and confronted with that of the most stable one, showing no relevant differences.

### 3.3. General Procedure for the Reactions of Substrates ***1a***–***e*** with 8-Hydroxyquinoline ***B***

The procedure was already reported in a previous paper [8] and has been herein extended to substrates **1d** and **1e**. In addition, here reaction times were prolonged, allowing for obtaining better yields.

In a flask, the relevant nitrostilbene **1** (0.15 mmol) was dissolved in DMSO (1 mL), and 8-hydroxyquinoline (0.15 mmol) and K_2_CO_3_ (0.19 mmol) were added as solids under magnetic stirring. The reaction was stirred at room temperature and monitored by TLC. Upon completion (24–45 h), the mixture was diluted with 20 mL of ethyl acetate, washed with HCl 1M (2 × 5 mL), NaHCO_3_ (2 × 5 mL), water (1 × 5 mL), and brine (1 × 5 mL). The organic phase was dried with anhydrous Na_2_SO_4_, and, after filtration, the solvent was evaporated. The crude was purified by column chromatography (petroleum ether/ethyl acetate 1:1 to 1:2), obtaining compounds **2Ba**–**e**. Hereinafter, we report the characterization of the two newly obtained dihydrofuroquinolines.

*2-(2-(Methylsulfonyl)phenyl)-3-(1-naphthyl)-2,3-dihydrofuro[3,2-h]quinoline* (**2Bd**). White solid. M.p. 151.0–152.7 °C (taken-up with E.P/DCM). ^1^H NMR (CDCl_3_, 400 MHz) δ 8.91 (dd, *J* = 4.5, 1.7 Hz, 1H), 8.19 (dd, *J* = 8.4, 1.7 Hz, 1H), 8.10 (dd, *J* = 7.7, 1.7 Hz, 1H), 7.86 (dd, *J* = 8.3, 1.3 Hz, 1H), 7.83–7.76 (m, 1H), 7.68 (s, 1H), 7.65–7.50 (m, 4H), 7.50–7.37 (m, 5H), 7.32–7.19 (m, 1H), 6.78 (br s, 1H), 6.03 (br s, 1H), 2.87 (s, 3H). ^13^C NMR (CDCl_3_, 101 MHz) δ 150.22, 140.38, 138.84, 138.04, 136.34, 136.03, 134.28, 133.85, 132.36, 131.93, 130.19, 129.55, 129.43, 129.02, 128.14, 126.35, 125.93, 125.66, 123.72, 122.74, 121.53, 121.38, 90.16, 53.53, 45.52 (three signals not visible because of isochrony). HRMS (ESI) *m*/*z* calculated [M + H]^+^ C_28_H_22_NO_3_S^+^ 452.1242, found 452.1244.
*2-(2-(Methylsulfonyl)phenyl)-3-(2-thienyl)-2,3-dihydrofuro[3,2-h]quinoline* (**2Be**). White solid. M.p. 174.0–175.6 °C (taken-up with E.P/DCM). ^1^H NMR (CDCl_3_, 400 MHz) δ 8.88 (dd, *J* = 4.2, 1.7 Hz, 1H), 8.20 (dd, *J* = 8.4, 1.7 Hz, 1H), 8.16 (dt, *J* = 7.6, 1.1 Hz, 1H), 7.62–7.58 (m, 2H), 7.54 (ddd, *J* = 7.6, 5.4, 3.5 Hz, 1H), 7.47 (d, *J* = 8.3 Hz, 1H), 7.44 (dd, *J* = 8.4, 4.2 Hz, 1H), 7.35 (dd, *J* = 8.2, 0.8 Hz, 1H), 7.25 (dd, *J* = 5.1, 1.3 Hz, 1H), 6.98 (dd, *J* = 5.1, 3.5 Hz, 1H), 6.95 (dd, *J* = 3.7, 1.3 Hz, 1H), 6.66 (d, *J* = 8.1 Hz, 1H), 5.38 (d, *J* = 8.1 Hz, 1H), 3.14 (s, 3H). ^13^C NMR (CDCl_3_, 101 MHz) δ 154.33, 150.25, 144.22, 139.54, 138.94, 136.35, 135.91, 134.16, 130.32, 129.73, 129.51, 129.47, 127.51, 127.24, 126.27, 125.30, 123.50, 121.65, 121.42, 90.23, 53.94, 45.78. HRMS (ESI) *m*/*z* calculated [M + H]^+^ C_22_H_18_NO_3_S_2_^+^ 408.0650, found 408.0658.

### 3.4. General Procedure for the Reactions of Substrates ***1a**–**e*** with 4-Hydroxycoumarin ***C***

In a round-bottom flask, the substrate **1** (0.157 mmol) was dissolved in chloroform (1.5 mL); then, 4-hydroxycoumarin (0.32 mmol) and Et_3_N (0.32 mmol) were added. The reaction was stirred at 60 °C for 24 h. The crude was purified by column chromatography (petroleum ether/ethyl acetate 2:1, then 1:1 and 1:2). These conditions represent a definite improvement with respect to the first attempt on the model substrate **1a** reported in [8] where an unsatisfactory (37%) yield was obtained. However, they failed when applied to substrate **1e** (Ar = 2-Th).

*2-(2-(Methylsulfonyl)phenyl)-3-(p-tolyl)-2,3-dihydro-4H-furo[3,2-c]chromen-4-one* (**2Ca**). White solid. M.p. 219.9–220.8 °C. ^1^H NMR (CDCl_3_, 400 MHz) δ 8.12 (dd, *J* = 8.0, 1.4 Hz, 1H), 7.75 (dd, *J* = 7.8, 1.7 Hz, 1H), 7.71 (td, *J* = 7.5, 1.4 Hz, 1H), 7.67–7.57 (m, 3H), 7.42 (dd, *J* = 8.5, 1.0 Hz, 1H), 7.33 (td, *J* = 7.6, 1.1 Hz, 1H), 7.20–7.10 (m, 4H), 6.98 (d, *J* = 6.4 Hz, 1H), 4.65 (d, *J* = 6.4 Hz, 1H), 2.73 (s, 3H), 2.30 (s, 3H). ^13^C NMR (CDCl_3_, 101 MHz) δ 166.11, 159.53, 155.57, 138.94, 138.48, 137.93, 136.44, 134.83, 133.09, 130.04, 129.97, 129.90, 128.52, 127.63, 124.29, 123.23, 117.26, 112.28, 105.67, 90.12, 55.37, 45.57, 21.28. HRMS (ESI) *m*/*z* calculated [M + H]^+^ C_25_H_21_O_5_S^+^ 433.1031, found 433.1027.
*3-(4-Methoxyphenyl)-2-(2-(methylsulfonyl)phenyl)-2,3-dihydro-4H-furo[3,2-c]chromen-4-one* (**2Cb**). White solid. M.p. 214.9–216.1 °C. ^1^H NMR (CDCl_3_, 400 MHz) δ 8.12 (dd, *J* = 7.9, 1.4 Hz, 1H), 7.75 (dd, *J* = 7.8, 1.6 Hz, 1H), 7.71 (td, *J* = 7.6, 1.4 Hz, 1H), 7.65–7.56 (m, 3H), 7.42 (dd, *J* = 8.5, 1.1 Hz, 1H), 7.33 (td, *J* = 7.6, 1.1 Hz, 1H), 7.25–7.16 (m, 2H), 6.97 (d, *J* = 6.4 Hz, 1H), 6.90–6.82 (m, 2H), 4.65 (d, *J* = 6.4 Hz, 1H), 3.77 (s, 3H), 2.76 (s, 3H). ^13^C NMR (CDCl_3_, 101 MHz) δ 166.01, 159.52, 159.43, 155.58, 138.96, 138.52, 134.80, 133.06, 131.47, 130.02, 129.90, 128.86, 128.49, 124.27, 123.21, 117.26, 114.64, 112.29, 105.70, 90.10, 55.35, 55.01, 45.60. HRMS (ESI) *m*/*z* calculated [M + H]^+^ C_25_H_21_O_6_S^+^ 449.0981, found 449.0990.
*3-(4-Chlorophenyl)-2-(2-(methylsulfonyl)phenyl)-2,3-dihydro-4H-furo[3,2-c]chromen-4-one* (**2Cc**). White solid. M.p. 253.0–253.7 °C. ^1^H NMR (CDCl_3_, 400 MHz) δ 8.13 (dd, *J* = 7.9, 1.4 Hz, 1H), 7.75 (dd, *J* = 7.8, 1.6 Hz, 1H), 7.70 (td, *J* = 7.6, 1.5 Hz, 1H), 767–7.60 (m, 2H), 7.60–7.55 (m, 1H), 7.44 (dd, *J* = 8.5, 1.0 Hz, 1H), 7.35 (dd, *J* = 7.6, 1.1 Hz, 1H), 7.34–7.28 (m, 2H), 7.26–7.19 (m, 2H), 6.94 (d, *J* = 5.9 Hz, 1H), 4.65 (d, *J* = 5.8 Hz, 1H), 2.88 (s, 3H). ^13^C NMR (CDCl_3_, 101 MHz) δ 166.23, 159.37, 155.62, 138.57, 138.33, 138.14, 134.85, 134.01, 133.31, 130.20, 130.07, 129.36 129.10, 128.16, 124.40, 123.18, 117.37, 112.10, 105.43, 89.92, 54.91, 45.65. HRMS (ESI) *m*/*z* calculated [M + H]^+^ C_24_H_18_ClO_5_S^+^ 453.0485, found 453.0492.
*2-(2-(Methylsulfonyl)phenyl)-3-(1-naphthyl)-2,3-dihydro-4H-furo[3,2-c]chromen-4-one* (**2Cd**). White solid. M.p. 216.0–217.0 °C. ^1^H NMR (CDCl_3_, 400 MHz) δ 8.11 (dd, *J* = 8.1, 1.4 Hz, 1H), 7.91 (d, *J* = 7.9 Hz, 1H), 7.87–7.78 (m, 3H), 7.76 (dd, *J* = 7.8, 1.6 Hz, 1H), 7.70–7.61 (m, 2H), 7.54–7.43 (m, 4H), 7.40 (ddd, *J* = 8.1, 6.8, 1.1 Hz, 1H), 7.34 (td, *J* = 7.6, 1.1 Hz, 1H), 7.26–7.23 (m, 1H), 6.89 (br s, 1H), 5.69 (br s, 1H), 2.29 (s, 3H). ^13^C NMR (CDCl_3_, 101 MHz) δ 166.98, 159.53, 155.70, 139.17, 138.87, 135.08, 134.20, 133.24, 131.46, 130.41, 130.11, 129.30, 128.90, 128.80, 126.54, 126.08, 126.00, 124.33, 123.30, 122.65, 117.29, 112.35, 104.60, 100.02, 90.28, 45.02 (two isochronous carbon). HRMS (ESI) *m*/*z* calculated [M + H]^+^ C_28_H_21_O_5_S^+^ 469.1031, found 469.1023.

### 3.5. Alternative Procedure for the Reaction of Substrates ***1c**–**e*** with 4-Hydroxycoumarin ***C***

In a round-bottom flask, the substrate **1e** (0.157 mmol) was dissolved in ethanol (1.5 mL); then, 4-hydroxycoumarin (0.32 mmol) and DABCO (0.24 mmol) were added. The reaction mixture was heated to 85 °C under magnetic stirring until the substrate disappeared (TLC). The product (a white solid) precipitated in EtOH, so it could be separated by filtration and washed with ethanol. The resulting products were pure enough and chromatography was not necessary. These conditions were also applied to the substrate **1c** and **1d**, confirming the solidity of the method and the easier isolation of the product.

- *2-(2-(Methylsulfonyl)phenyl)-3-(thien-2-yl)-2,3-dihydro-4H-furo[3,2-c]chromen-4-one* (**2Ce**). Pale yellow solid. M.p. 213.0–214.0 °C. ^1^H NMR (CDCl_3_, 400 MHz) δ 8.15 (dd, *J* = 8.0, 1.4 Hz, 1H), 7.74 (dd, *J* = 7.8, 1.6 Hz, 1H), 7.70 (dd, *J* = 7.6, 1.5 Hz, 1H), 7.67–7.57 (m, 3H), 7.44 (d, *J* = 8.4 Hz, 1H), 7.33 (t, *J* = 7.6 Hz, 1H), 7.27–7.23 (m, 1H), 7.05–7.00 (m, 2H), 6.96 (dd, *J* = 5.1, 3.5 Hz, 1H), 5.04 (d, *J* = 6.4 Hz, 1H), 2.87 (s, 3H). ^13^C NMR (CDCl_3_, 101 MHz) δ 166.16, 159.34, 155.62, 142.45, 138.74, 138.21, 134.85, 133.36, 130.28, 130.09, 128.48, 127.47, 126.31, 125.63, 124.37, 123.33, 117.33, 112.15, 105.00, 90.07, 50.58, 45.63. HRMS (ESI) *m*/*z* calculated [M + H]^+^ C_22_H_17_O_5_S_2_^+^ 425.0439, found 425.0442.

### 3.6. Extending the Coupling Methodology to Other Heterocyclic Phenols: 5-Hydroxyindole ***D*** and N,N′-Dimethylbarbituric Acid ***E***

In a round-bottom flask, substrate **1** (0.157 mmol) was dissolved in CH_3_CN/H_2_O (97/3) (1.5 mL); then, 5-hydroxyindole **D** or *N,N*′-dimethylbarbituric acid **E** (0.157 mmol) and K_2_CO_3_ (0.157 mmol) were added. The reaction was stirred at 60 °C for the appropriate time. The crude was purified by column chromatography (petroleum ether/ethyl acetate 2:1, then 1:1 and 1:2). In the case of 5-hydroxyindole **D**, the expected dihydrofurans were always isolated in a mixture with minor quantities of the corresponding aromatized furan derivatives.

*2-(2-(Methylsulfonyl)phenyl)-1-(p-tolyl)-1,6-dihydro-2H-furo[3,2-e]indole* (**2Da**). ^1^H NMR (CDCl_3_, 400 MHz) δ 8.11 (s, 1H), 8.08 (dd, *J* = 8.0, 1.4 Hz, 1H), 7.79 (dd, *J* = 7.9, 1.3 Hz, 1H), 7.62 (td, *J* = 7.6, 1.4 Hz, 1H), 7.50 (ddd, *J* = 8.0, 7.4, 1.4 Hz, 1H), 7.28 (dt, *J* = 8.7, 0.9 Hz, 1H), 7.14 (d, *J* = 8.1 Hz, 2H), 7.10–7.06 (m, 3H), 6.87 (d, *J* = 8.6 Hz, 1H), 6.60 (d, *J* = 7.2 Hz, 1H,), 5.93 (ddd, *J* = 3.1, 2.0, 0.9 Hz, 1H), 4.94 (d, *J* = 7.2 Hz, 1H), 2.76 (s, 3H,), 2.29 (s, 3H). ^13^C NMR (CDCl_3_, 101 MHz) δ 153.51, 141.49, 138.62, 138.47, 136.94, 134.29, 132.61, 129.53, 129.44, 129.15, 128.92, 128.19, 125.78, 124.70, 118.42, 111.25, 105.42, 99.86, 87.23, 58.17, 45.37, 21.23. HRMS (ESI) *m*/*z* calculated [M + H]^+^ C_24_H_22_NO_3_S^+^ 404.1242, found 404.1244.
*8-(4-Methoxyphenyl)-7-(2-(methylsulfonyl)phenyl)-7,8-dihydro-1H-furo[2,3-g]indole* (**2Db**). White solid. M.p. 119.0–120.0 °C. ^1^H NMR (CDCl_3_, 400 MHz) δ 8.14 (s, 1H), 8.08 (dd, *J* = 8.0, 1.4 Hz, 1H), 7.79 (dd, *J* = 7.9, 1.3 Hz, 1H), 7.62 (td, *J* = 7.6, 1.5 Hz, 1H), 7.55–7.45 (m, 1H), 7.28 (dt, *J* = 8.6, 0.9 Hz, 1H), 7.19–7.13 (m, 2H), 7.09 (t, *J* = 2.9 Hz, 1H), 6.87 (d, *J* = 8.7 Hz, 1H), 6.84–6.78 (m, 2H), 6.58 (d, *J* = 7.4 Hz, 1H), 5.92 (ddd, *J* = 3.1, 2.0, 0.9 Hz, 1H), 4.94 (d, *J* = 7.4 Hz, 1H), 3.75 (s, 3H), 2.78 (s, 3H). ^13^C NMR (CDCl_3_, 101 MHz) δ 158.86, 153.46, 141.45, 138.67, 134.28, 133.54, 132.62, 129.46, 129.39, 129.18, 128.92, 125.76, 124.67, 118.49, 114.18, 111.24, 105.44, 99.88, 87.28, 57.78, 55.27, 45.41. HRMS (ESI) *m*/*z* calculated [M + H]^+^ C_24_H_22_NO_4_S^+^ 420.1191, found 420.1188.
*8-(4-Chlorophenyl)-7-(2-(methylsulfonyl)phenyl)-7,8-dihydro-1H-furo[2,3-g]indole* (**2Dc**). Always in mixture with **3Dc**: ^1^H-NMR peaks evaluated by subtraction. ^1^H NMR (CDCl_3_, 400 MHz) δ 8.23 (s, 1H), 8.08 (dd, *J* = 7.9, 1.5 Hz, 1H), 7.72 (dd, *J* = 7.9, 1.4 Hz, 1H), 7.64–7.56 (m, 1H), 7.56–7.45 (m, 1H), 7.29 (dt, *J* = 8.7, 1.0 Hz, 1H), 7.26–7.22 (m, 2H), 7.20–7.16 (m, 2H), 7.10 (t, *J* = 2.9 Hz, 1H), 6.88 (d, *J* = 8.6 Hz, 1H), 6.52 (d, *J* = 6.4 Hz, 1H), 5.92 (ddd, *J* = 3.1, 2.0, 1.0 Hz, 1H), 4.91 (d, *J* = 6.4 Hz, 1H), 2.93 (s, 3H).
*7-(2-(Methylsulfonyl)phenyl)-8-(naphthalen-1-yl)-7,8-dihydro-1H-furo[2,3-g]indole* (**2Dd**). White solid. M.p. 155.0–156.0 °C. ^1^H NMR (CDCl_3_, 400 MHz) δ 8.17 (s, 1H), 8.04 (dd, *J* = 8.0, 1.4 Hz, 1H), 7.96 (d, *J* = 7.5 Hz, 1H), 7.84 (d, *J* = 8.2 Hz, 1H), 7.80–7.73 (m, 1H), 7.69 (td, *J* = 7.7, 1.4 Hz, 1H), 7.63–7.49 (m, 2H), 7.44–7.36 (m, 3H), 7.32 (d, *J* = 8.6 Hz, 1H), 7.29–7.21 (m, 1H), 7.07 (s, 1H), 6.92 (d, *J* = 8.7 Hz, 1H), 6.61 (d, *J* = 6.9 Hz, 1H), 5.96 (s, 1H), 5.79 (t, *J* = 2.6 Hz, 1H), 2.38 (s, 3H). ^13^C NMR (CDCl_3_, 101 MHz) δ 164.31, 153.88, 141.39, 139.00, 137.44, 134.47, 133.88, 133.83, 132.60, 131.91, 129.61, 129.18, 129.06, 127.80, 126.16, 125.76, 125.72, 124.68, 122.90, 117.88, 111.42, 105.79, 100.45, 87.52, 52.91, 44.94 (two isochronous carbons). HRMS (ESI) *m*/*z* calculated [M + H]^+^ C_27_H_22_NO_3_S^+^ 440.1242, found 440.1235.
*7-(2-(Methylsulfonyl)phenyl)-8-(thiophen-2-yl)-7,8-dihydro-1H-furo[2,3-g]indole* (**2De**). White solid. M.p. 138.0–139.0 °C. ^1^H NMR (CDCl_3_, 400 MHz) δ 8.20 (s, 1H), 8.11 (dd, *J* = 7.9, 1.4 Hz, 1H), 7.74 (dd, *J* = 7.8, 1.4 Hz, 1H), 7.61 (td, *J* = 7.6, 1.5 Hz, 1H), 7.52 (td, *J* = 7.7, 1.4 Hz, 1H), 7.30 (d, *J* = 8.9 Hz, 1H), 7.19 (dd, *J* = 5.0, 1.4 Hz, 1H), 7.13 (t, *J* = 2.9 Hz, 1H), 6.97–6.90 (m, 2H), 6.85 (d, *J* = 8.7 Hz, 1H), 6.63 (d, *J* = 7.4 Hz, 1H), 6.11–6.03 (m, 1H), 5.32 (d, *J* = 7.4 Hz, 1H), 2.89 (s, 3H). ^13^C NMR (CDCl_3_, 101 MHz) δ 153.23, 144.70, 140.67, 138.91, 134.30, 132.71, 129.67, 129.16, 127.03, 126.00, 125.80, 124.91, 124.65, 117.70, 111.78, 105.46, 99.80, 87.49, 53.18, 45.42 (two isochronous carbons). HRMS (ESI) *m*/*z* calculated [M + H]^+^ C_21_H_18_NO_3_S_2_^+^ 396.0650, found 396.0659.
*1,3-Dimethyl-6-(2-(methylsulfonyl)phenyl)-5-(p-tolyl)-5,6-dihydrofuro[2,3-d]pyrimidine-2,4(1H,3H)-dione* (**2Ea**). White solid. M.p. 284.0–285.0 °C. ^1^H NMR (CDCl_3_, 400 MHz) δ 8.03 (dd, *J* = 7.8, 1.2 Hz, 1H), 7.72–7.61 (m, 2H), 7.55 (ddd, *J* = 8.6, 6.4, 2.2 Hz, 1H), 7.25 (d, *J* = 8.0 Hz, 2H), 7.17 (d, *J* = 7.9 Hz, 2H), 4.22–4.11 (m, 2H), 3.23 (s, 3H), 3.18 (s, 3H), 3.07 (s, 3H), 2.35 (s, 3H). ^13^C NMR (CDCl_3_, 101 MHz) δ 166.39, 165.69, 152.06, 139.56, 138.27, 133.97, 133.87, 132.12, 129.89, 129.59, 129.30, 129.11, 46.40, 43.78, 40.81, 28.93, 28.76, 21.37 (two isochronous carbons). HRMS (ESI) *m*/*z* calculated [M + H]^+^ C_22_H_23_N_2_O_5_S^+^ 427.1249, found 427.1238.
*5-(4-Methoxyphenyl)-1,3-dimethyl-6-(2-(methylsulfonyl)phenyl)-5,6-dihydrofuro[2,3-d]pyrimidine-2,4(1H,3H)-dione* (**2Eb**). White solid. M.p. 108.0–109.0 °C. Mix stereoisomers. ^1^H NMR (CDCl_3_, 400 MHz) δ 8.08 (dd, *J* = 8.0, 1.4 Hz, 1H), 8.03 (dd, *J* = 8.1, 1.5 Hz, 1H), 7.70–7.61 (m, 3H), 7.58–7.51 (m, 1H), 7.51–7.43 (m, 2H), 7.33–7.26 (m, 4H), 6.95–6.85 (m, 4H), 5.74 (s, 2H), 4.19 (d, *J* = 9.4 Hz, 1H), 4.14 (d, *J* = 9.4 Hz, 1H), 3.81 (s, 3H), 3.80 (s, 3H), 3.47 (s, 3H), 3.32 (s, 3H), 3.25 (s, 3H), 3.22 (s, 3H), 3.18 (s, 3H), 3.07 (s, 3H). ^13^C NMR (CDCl_3_, 101 MHz) δ 166.37, 165.65, 162.27, 160.53, 159.98, 159.62, 152.06, 151.74, 142.42, 139.54, 139.05, 134.68, 133.99, 133.86, 132.10, 130.62, 129.65, 129.58, 129.46, 129.37, 129.10, 128.27, 127.69, 124.69, 114.34, 113.93, 97.30, 91.56, 55.38, 55.36, 47.04, 46.49, 45.49, 43.78, 43.31, 40.77, 29.98, 28.92, 28.75, 28.10. HRMS (ESI) *m*/*z* calculated [M + H]^+^ C_22_H_23_N_2_O_5_S^+^ 443.1199, found 443.1190.
*5-(4-Chlorophenyl)-1,3-dimethyl-6-(2-(methylsulfonyl)phenyl)-5,6-dihydrofuro[2,3-d]pyrimidine-2,4(1H,3H)-dione* (**2Ec**). White solid. M.p. 115.0–116.0 °C. ^1^H NMR (CDCl_3_, 400 MHz) δ 8.02 (d, *J* = 7.9 Hz, 1H), 7.68 (t, *J* = 7.5 Hz, 2H), 7.63 (d, *J* = 7.4 Hz, 1H), 7.56 (t, *J* = 7.6 Hz, 1H), 7.33 (d, *J* = 8.8 Hz, 2H), 7.30 (d, *J* = 8.6 Hz, 2H), 4.15 (s, 2H), 3.23 (s, 3H), 3.18 (s, 3H), 3.07 (s, 3H). ^13^C NMR (CDCl_3_, 101 MHz) δ 166.06, 165.65, 151.89, 139.59, 134.33, 133.88, 133.45, 132.06, 131.54, 130.77, 129.62, 129.30, 128.78, 45.20, 43.85, 41.09, 28.95, 28.81 (two isochronous carbons). HRMS (ESI) *m*/*z* calculated [M + H]^+^ C_21_H_20_ClN_2_O_5_S^+^ 447.0703, found 447.0709.
*1,3-Dimethyl-6-(2-(methylsulfonyl)phenyl)-5-(naphthalen-1-yl)-5,6-dihydrofuro[2,3-d]pyrimidine-2,4(1H,3H)-dione* (**2Ed**). White solid. M.p. 245.0–246.0 °C. ^1^H NMR (CDCl_3_, 400 MHz) δ 8.08 (dd, *J* = 7.8, 1.3 Hz, 1H), 7.93–7.86 (m, 2H), 7.77–7.68 (m, 2H), 7.66–7.57 (m, 3H), 7.54–7.46 (m, 3H), 4.48 (d, *J* = 9.3 Hz, 1H), 4.34 (d, *J* = 9.3 Hz, 1H), 3.29 (s, 3H), 3.10 (s, 3H), 3.02 (s, 3H). ^13^C NMR (CDCl_3_, 101 MHz) δ 166.53, 165.38, 152.07, 139.71, 133.99, 133.92, 133.60, 132.37, 132.17, 129.69, 129.61, 129.35, 129.32, 129.29, 127.34, 127.11, 126.18, 125.29, 122.34, 44.16, 43.86, 41.14, 28.97, 28.80 (two isochronous carbons). HRMS (ESI) *m*/*z* calculated [M + H]^+^ C_25_H_23_N_2_O_5_S^+^ 463.1249, found 463.1240.
*1,3-Dimethyl-6-(2-(methylsulfonyl)phenyl)-5-(thiophen-2-yl)-5,6-dihydrofuro[2,3-d]pyrimidine-2,4(1H,3H)-dione* (**2Ee**). Always in mixture with a minor unknown compound after chromatography. ^1^H NMR (CDCl_3_, 400 MHz) δ 8.02 (dd, *J* = 7.9, 1.3 Hz, 1H), 7.72–7.62 (m, 2H), 7.55 (ddd, *J* = 8.7, 6.8, 1.9 Hz, 1H), 7.29 (dd, *J* = 5.2, 1.2 Hz, 1H), 7.13 (d, *J* = 3.5 Hz, 1H), 7.01 (dd, *J* = 5.1, 3.6 Hz, 1H), 4.20 (s, 2H), 3.26 (s, 3H), 3.17 (s, 3H), 3.07 (s, 3H).

### 3.7. General Procedure for the Aromatization of ***2Ba***–***e*** with DDQ

As already reported in [8], adequate conditions for *N*-heterocycles require only 2 mol equiv. of the oxidant DDQ, in CHCl_3_ at room temperature; the desired furoquinoline was thus obtained within 24 h after chromatography in 91% yields. These successful conditions were then applied to all of the dihydrofuroquinolines prepared, furnishing generally good yields (73–99%) within 24–30 h. Only in the case of Ar = 1-Naphthyl, it was necessary to heat the reaction at reflux, employing 3 mol equiv. of DDQ.

*3-(4-Methyphenyl)-2-(2-(methylsulfonyl)phenyl)furo[3,2-h]quinoline* (**3Ba**). The compound was already characterized in [8].
*3-(4-Methoxyphenyl)-2-(2-(methylsulfonyl)phenyl)furo[3,2-h]quinoline* (**3Bb**). White solid. M.p. 146.5–147.7 °C (taken up with EP/DCM). ^1^H NMR (CDCl_3_, 400 MHz) δ 8.92 (dd, *J* = 4.3, 1.7 Hz, 1H), 8.30–8.24 (m, 2H), 7.83 (d, *J* = 8.6 Hz, 1H), 7.69 (d, *J* = 8.6 Hz, 1H), 7.60 (td, *J* = 7.7, 1.5 Hz, 1H), 7.53 (td, *J* = 7.6, 1.5 Hz, 1H), 7.45 (dd, *J* = 8.3, 4.3 Hz, 1H), 7.43–7.37 (m, 3H), 6.96–6.88 (m, 2H), 3.81 (s, 3H), 3.62 (s, 3H). ^13^C NMR (CDCl_3_, 101 MHz) δ 159.23, 150.28, 149.35, 149.13, 141.19, 137.08, 136.42, 133.43, 133.34, 130.83, 130.66, 129.93, 128.04, 126.83, 123.66, 123.51, 120.75, 120.64, 120.03, 114.44, 55.35, 46.10 (two isochronous carbons). HRMS (ESI) *m*/*z* calculated [M + H]^+^ C_25_H_20_NO_4_S^+^ 430.1235, found 430.1241.
*3-(4-Chlorophenyl)-2-(2-(methylsulfonyl)phenyl)furo[3,2-h]quinoline* (**3Bc**). The compound has been already characterized [8].
*2-(2-(Methylsulfonyl)phenyl)-3-(naphth-1-yl)furo[3,2-h]quinoline* (**3Bd**). White solid. M.p. 167.2–169.3 °C (taken up with EP/DCM). ^1^H NMR (CDCl_3_, 400 MHz) δ 8.96 (dd, *J* = 4.4, 1.6 Hz, 1H), 8.27 (dd, *J* = 8.3, 1.7 Hz, 1H), 8.23 (dd, *J* = 8.0, 1.3 Hz, 1H), 7.97 (dd, *J* = 8.4, 1.1 Hz, 1H), 7.93–7.86 (m, 2H), 7.61 (d, *J* = 8.6 Hz, 1H), 7.57 (dd, *J* = 7.0, 1.3 Hz, 1H), 7.51–7.43 (m, 4H), 7.47–7.35 (m, 2H), 7.31 (td, *J* = 7.6, 1.3 Hz, 1H), 7.20 (dd, *J* = 7.7, 1.4 Hz, 1H), 3.77 (s, 3H). ^13^C NMR (CDCl_3_, 101 MHz) δ 151.00, 150.35, 149.27, 140.64, 137.16, 136.51, 133.89, 133.20, 132.62, 132.46, 130.72, 130.37, 129.82, 129.46, 129.37, 128.77, 128.72, 128.45, 126.99, 126.57, 126.19, 126.11, 125.82, 123.57, 120.83, 120.52, 119.69, 46.38. HRMS (ESI) *m*/*z* calculated [M + H]^+^ C_28_H_20_NO_3_S^+^ 450.1086, found 450.1095.
*2-(2-(Methylsulfonyl)phenyl)-3-(thien-2-yl)furo[3,2-h]quinoline* (**3Be**). White solid. M.p. 146.5–147.7 °C (taken up with EP/DCM). ^1^H NMR (CDCl_3_, 400 MHz) δ 8.93 (dd, *J* = 4.3, 1.7 Hz, 1H),8.32–8.26 (m, 2H), 8.02 (d, *J* = 8.6 Hz, 1H), 7.74 (d, *J* = 8.6 Hz, 1H), 7.71–7.63 (m, 2H), 7.62 (td, *J* = 7.3, 1.8 Hz, 1H), 7.47 (dd, *J* = 8.3, 4.3 Hz, 1H), 7.32 (dd, *J* = 5.1, 1.2 Hz, 1H), 7.15 (dd, *J* = 3.6, 1.2 Hz, 1H), 7.05 (dd, *J* = 5.1, 3.6 Hz, 1H), 3.44 (s, 3H). ^13^C NMR (CDCl_3_, 101 MHz) δ150.44, 149.39, 149.32, 141.39, 136.91, 136.49, 133.72, 133.43, 132.47, 130.63, 130.56, 130.20, 127.65, 127.30, 127.08, 126.94, 125.91, 123.81, 120.99, 120.07, 115.16, 45.83. HRMS (ESI) *m*/*z* calculated [M + H]^+^ C_22_H_16_NO_3_S_2_^+^ 406.0493, found 406.0493.

### 3.8. General Procedure for Aromatization of ***2Ca***,***b***,***c***,***e*** and ***2Eb*** with DDQ

The relevant dihydrofuran (0.12 mmol) was dissolved in chloroform (3.0 mL) and DDQ (0.48 mmol) was added. The reaction was heated at 60 °C and maintained under magnetic stirring, until completion (monitored by TLC). Then, an aqueous saturated NaHCO_3_ solution (8 mL) was added, and the resulting mixture was extracted with CHCl_3_ (3 × 15 mL). The combined organic layers were dried over anhydrous Na_2_SO_4_, filtered, and concentrated in vacuo. The reaction mixture was purified by column chromatography (petroleum ether/ethyl acetate 1:1) to afford the desired aromatized products **3Ca**,**b**,**e**, and **3Eb**: as mentioned in footnote *a* of Table 6, **3Cc** could be isolated only in admixture with **2Cc**.

*2-(2-(Methylsulfonyl)phenyl)-3-(p-tolyl)-4H-furo[3,2-c]chromen-4-one* (**3Ca**). White solid. M.p. 135.0–136.0 °C (XX EtOH). ^1^H NMR (CDCl_3_, 400 MHz) δ 8.22 (dd, *J* = 7.9, 1.5 Hz, 1H), 7.78 (dd, *J* = 7.8, 1.6 Hz, 1H), 7.65 (td, *J* = 7.7, 1.5 Hz, 1H), 7.61–7.50 (m, 2H), 7.47 (d, *J* = 8.3 Hz, 1H), 7.40–7.33 (m, 2H), 7.31 (d, *J* = 7.9 Hz, 2H), 7.10 (d, *J* = 7.8 Hz, 2H), 3.16 (s, 3H), 2.31 (s, 3H). ^13^C NMR (CDCl_3_, 101 MHz) δ 157.58, 157.40, 152.92, 148.68, 140.83, 138.24, 134.20, 133.70, 131.13, 130.76, 130.68, 130.28, 129.14, 125.92, 124.61, 123.72, 120.61, 117.52, 112.73, 110.30, 45.24, 21.40 (two isochronous carbons). HRMS (ESI) *m*/*z* calculated [M + H]^+^ C_25_H_19_O_5_S^+^ 431.0875, found 431.0868.
*3-(4-Methoxyphenyl)-2-(2-(methylsulfonyl)phenyl)-4H-furo[3,2-c]chromen-4-one* (**3Cb**). White solid. M.p. 113.0–114.0 °C (XX EtOH). ^1^H NMR (CDCl_3_, 400 MHz) δ 8.23 (dd, *J* = 7.9, 1.4 Hz, 1H), 7.78 (dd, *J* = 7.8, 1.5 Hz, 1H), 7.65 (td, *J* = 7.7, 1.5 Hz, 1H), 7.59 (td, *J* = 7.5, 1.5 Hz, 1H), 7.54 (ddd, *J* = 8.7, 7.2, 1.6 Hz, 1H), 7.48 (dd, *J* = 8.4, 1.2 Hz, 1H), 7.40–7.30 (m, 4H), 6.86–6.80 (m, 2H), 3.78 (s, 3H), 3.19 (s, 3H). ^13^C NMR (CDCl_3_, 101 MHz) δ 159.61, 157.70, 157.39, 152.90, 148.45, 140.81, 134.20, 133.75, 131.72, 131.14, 130.75, 130.72, 129.20, 124.63, 123.41, 121.10, 120.60, 117.54, 113.90, 112.74, 110.26, 55.31, 45.27. HRMS (ESI) *m*/*z* calculated [M + H]^+^ C_25_H_19_O_6_S^+^ 447.0824, found 447.0818.
*2-(2-(Methylsulfonyl)phenyl)-3-(thiophen-2-yl)-4H-furo[3,2-c]chromen-4-one* (**3Ce**). White solid. M.p. 241.0–242.0 °C (XX EtOH). ^1^H NMR (CDCl_3_, 400 MHz) δ 8.29–8.23 (m, 1H), 7.83 (dd, *J* = 7.9, 1.6 Hz, 1H), 7.81–7.73 (m, 3H), 7.67–7.62 (m, 1H), 7.55 (ddd, *J* = 8.7, 7.3, 1.6 Hz, 1H), 7.48 (dd, *J* = 8.5, 1.2 Hz, 1H), 7.34 (ddd, *J* = 8.2, 7.3, 1.2 Hz, 1H), 7.20 (dd, *J* = 5.1, 1.2 Hz, 1H), 7.02 (dd, *J* = 5.1, 3.7 Hz, 1H), 2.91 (s, 3H). ^13^C NMR (CDCl_3_, 101 MHz) δ 157.87, 157.65, 152.94, 147.79, 141.33, 134.95, 133.88, 131.73, 131.44, 130.70, 130.64, 129.69, 128.80, 127.62, 126.68, 124.72, 121.08, 117.88, 117.34, 112.47, 109.29, 44.67. HRMS (ESI) *m*/*z* calculated [M + H]^+^ C_22_H_15_O_5_S^+^ 423.0283, found 423.0290.
*5-(4-Methoxyphenyl)-1,3-dimethyl-6-(2-(methylsulfonyl)phenyl)furo[2,3-d]pyrimidine-2,4(1H,3H)-dione* (**3Eb**). White solid. M.p. 223.0–224.0 °C (XX EtOH). ^1^H NMR (CDCl_3_, 400 MHz) δ 8.25–8.19 (m, 1H), 7.71–7.63 (m, 2H), 7.43–7.37 (m, 1H), 7.21–7.14 (m, 2H), 6.78–6.72 (m, 2H), 3.75 (s, 3H), 3.67 (s, 3H), 3.32 (s, 3H), 2.96 (s, 3H). ^13^C NMR (CDCl_3_, 101 MHz) δ 159.64, 158.29, 154.20, 150.53, 146.24, 140.02, 134.17, 133.08, 131.34, 130.39, 129.82, 126.80, 121.37, 114.36, 99.71, 55.37, 44.42, 29.70, 28.33 (two isochronous carbons). HRMS (ESI) *m*/*z* calculated [M + H]^+^ C_22_H_21_N_2_O_6_S^+^ 441.1042, found 441.1038.

### 3.9. General Procedure for Aromatization of ***2Da***–***e*** with DDQ

The relevant dihydrofuran **2D** (0.12 mmol) was dissolved in toluene (3.0 mL) and DDQ (0.48 mmol) was added. The reaction was maintained under magnetic stirring, until completion (monitored by TLC). Then, an aqueous saturated NaHCO_3_ solution (8 mL) was added, and the resulting mixture was extracted with CHCl_3_ (3 × 15 mL). The combined organic layers were dried over anhydrous Na_2_SO_4_, filtered, and concentrated in vacuo. The reaction mixture was purified by column chromatography (petroleum ether/ethyl acetate 1:1) to afford the desired products **3Da**–**e**.

*7-(2-(Methylsulfonyl)phenyl)-8-(p-tolyl)-1H-furo[2,3-g]indole* (**3Da**). White solid. M.p. 152.0–153.0 °C. ^1^H NMR (CDCl_3_, 400 MHz) δ 8.40 (s, 1H), 8.23 (dd, *J* = 7.9, 1.5 Hz, 1H), 7.54 (td, *J* = 7.7, 1.5 Hz, 1H), 7.47 (td, *J* = 7.5, 1.5 Hz, 1H), 7.44–7.40 (m, 2H), 7.35 (s, 2H), 7.32 (dd, *J* = 7.6, 1.5 Hz, 1H), 7.19–7.13 (m, 3H), 6.54 (dd, *J* = 3.2, 1.9 Hz, 1H), 3.37 (s, 3H), 2.37 (s, 3H). ^13^C NMR (CDCl_3_, 101 MHz) δ 150.05, 147.35, 140.64, 137.12, 133.95, 133.21, 132.66, 131.22, 130.30, 130.17, 129.73, 129.38, 129.20, 123.76, 121.23, 120.37, 120.33, 109.01, 105.96, 101.88, 45.15, 21.41. HRMS (ESI) *m*/*z* calculated [M + H]^+^ C_24_H_20_NO_3_S^+^ 402.1086, found 402.1080.
*8-(4-Methoxyphenyl)-7-(2-(methylsulfonyl)phenyl)-1H-furo[2,3-g]indole* (**3Db**). White solid. M.p. 137.0–138.0 °C. ^1^H NMR (CDCl_3_, 400 MHz) δ 8.38 (s, 1H), 8.23 (dd, *J* = 7.9, 1.5 Hz, 1H), 7.54 (td, *J* = 7.7, 1.5 Hz, 1H), 7.51–7.43 (m, 3H), 7.34 (s, 2H), 7.32 (dd, *J* = 7.5, 1.5 Hz, 1H), 7.18–7.16 (m, 1H), 6.93–6.87 (m, 2H), 6.54 (dd, *J* = 3.2, 2.1 Hz, 1H), 3.82 (s, 3H), 3.38 (s, 3H). ^13^C NMR (CDCl_3_, 101 MHz) δ 159.04, 150.02, 147.25, 140.65, 133.94, 133.21, 132.65, 131.45, 131.24, 130.32, 129.36, 125.01, 123.81, 120.90, 120.41, 120.35, 113.96, 109.00, 105.98, 101.81, 55.31, 45.17. HRMS (ESI) *m*/*z* calculated [M + H]^+^ C_24_H_20_NO_4_S^+^ 418.1035, found 418.11040.
*8-(4-Chlorophenyl)-7-(2-(methylsulfonyl)phenyl)-1H-furo[2,3-g]indole* (**3Dc**). White solid. M.p. 162.0–163.0 °C. ^1^H NMR (CDCl_3_, 400 MHz) δ 8.43 (s, 1H), 8.24 (dd, *J* = 7.9, 1.4 Hz, 1H), 7.57 (td, *J* = 7.7, 1.4 Hz, 1H), 7.54–7.45 (m, 3H), 7.37–7.30 (m, 4H), 7.27 (dd, *J* = 7.6, 1.4 Hz, 1H), 7.17 (d, *J* = 3.0 Hz, 1H), 6.49 (t, *J* = 2.5 Hz, 1H), 3.41 (s, 3H). ^13^C NMR (CDCl_3_, 101 MHz) δ 150.09, 147.80, 140.75, 133.85, 133.42, 133.39, 132.71, 131.61, 131.42, 130.78, 130.46, 129.72, 128.78, 124.03, 120.12, 119.89, 109.26, 105.94, 101.60, 45.30 (two isochronous carbons). HRMS (ESI) *m*/*z* calculated [M + H]^+^ C_23_H_17_ClNO_3_S^+^ 422.0539, found 422.0537.
*7-(2-(Methylsulfonyl)phenyl)-8-(naphthalen-1-yl)-1H-furo[2,3-g]indole* (**3Dd**). White solid. M.p. 276.0–277.0 °C. ^1^H NMR (CDCl_3_, 400 MHz) δ 8.30 (s, 1H), 8.18 (dd, *J* = 8.0, 1.3 Hz, 1H), 8.00 (d, *J* = 8.4 Hz, 1H), 7.93–7.84 (m, 2H), 7.58 (dd, *J* = 7.0, 1.3 Hz, 1H), 7.51–7.29 (m, 6H), 7.30–7.21 (m, 1H), 7.17 (dd, *J* = 7.7, 1.4 Hz, 1H), 6.95 (t, *J* = 2.9 Hz, 1H), 5.59 (t, *J* = 2.5 Hz, 1H), 3.53 (s, 3H). ^13^C NMR (CDCl_3_, 101 MHz) δ 149.94, 148.94, 140.02, 133.74, 133.15, 133.00, 132.70, 132.55, 130.89, 130.51, 130.47, 129.30, 129.24, 128.31, 128.22, 126.70, 126.41, 126.05, 125.75, 123.85, 121.85, 120.50, 119.39, 109.16, 105.87, 101.73, 45.52. HRMS (ESI) *m*/*z* calculated [M + H]^+^ C_27_H_20_NO_3_S^+^ 438.1086, found 438.1082.
*7-(2-(Methylsulfonyl)phenyl)-8-(thiophen-2-yl)-1H-furo[2,3-g]indole* (**3De**). White solid. M.p. 200.0–201.0 °C. ^1^H NMR (CDCl_3_, 400 MHz) δ 8.44 (1H, s), 8.25–8.20 (m, 1H), 7.64–7.54 (m, 2H), 7.52–7.47 (m, 1H), 7.37–7.33 (m, 2H), 7.30 (dd, *J* = 5.1, 1.2 Hz, 1H), 7.27–7.24 (m, 1H), 7.20 (t, *J* = 2.8 Hz, 1H), 7.06 (dd, *J* = 5.1, 3.5 Hz, 1H), 6.72 (dd, *J* = 3.2, 2.1 Hz, 1H), 3.24 (s, 3H). ^13^C NMR (CDCl_3_, 101 MHz) δ 150.00, 148.21, 140.87, 134.08, 133.41, 133.20, 132.79, 130.60, 130.16, 129.96, 128.31, 127.31, 126.04, 124.03, 120.09, 120.01, 114.81, 109.39, 106.02, 101.78, 44.91. HRMS (ESI) *m*/*z* calculated [M + H]^+^ C_21_H_16_NO_3_S_2_^+^ 394.0493, found 394.0497.

### 3.10. General Procedure for 6π-Electrocyclization of ***3B*** and ***3C***

The relevant furan **3B** or **3C** (0.072 mmol) was dissolved in 3,5 mL of acetone (spectroscopic grade) and put in a 25 mL quartz tube; the tube was sealed, placed in a Rayonet photochemical reactor, and irradiated by UV light at 350 nm. The progress of the reaction was followed in TLC; after completion, the solvent was removed under reduced pressure and the residue was purified by column chromatography (petroleum ether/ethyl acetate) or crystallization. From the reactions, only the fully aromatized products **5B** and **5C** were isolated and characterized due to a fast methanesulfinic acid elimination (see Scheme 11).

*9-Methylphenanthro[9′,10′:4,5]furo[3,2-h]quinoline* (**5Ba**). White solid. M.p. 209.2–210.8 °C (taken up with EP/DCM). ^1^H NMR (CDCl_3_, 400 MHz) δ 9.11 (d, *J* = 3.4 Hz, 1H), 8.82 (dd, *J* = 8.0, 1.6 Hz, 1H), 8.75 (d, *J* = 7.4 Hz, 1H), 8.61–8.54 (m, 2H), 8.51 (d, *J* = 8.6 Hz, 1H), 8.33 (dt, *J* = 8.2, 1.2 Hz, 1H), 7.84 (d, *J* = 8.5 Hz, 1H), 7.78–7.67 (m, 2H), 7.60 (d, *J* = 9.0 Hz, 1H), 7.51 (dd, *J* = 8.4, 4.1 Hz, 1H), 2.67 (s, 3H). ^13^C NMR (CDCl_3_, 101 MHz) δ 151.50, 150.68, 150.42,137.06, 136.53, 135.11, 130.36, 130.28, 129.09, 128.99, 128.72, 127.25, 127.19, 126.84, 126.07, 124.98, 123.95, 123.89, 123.53, 123.41, 122.46, 122.27, 120.93, 22.16. HRMS (ESI) *m*/*z* calculated [M + H]^+^ C_24_H_16_NO^+^ 334.1154, found 334.1150.
*9-Methoxyphenanthro[9′,10′:4,5]furo[3,2-h]quinoline* (**5Bb**). White solid. M.p. 217.8–218.3 °C (taken up with EP/DCM). ^1^H NMR (CDCl_3_, 400 MHz) δ 9.10 (dd, *J* = 4.3, 1.7 Hz, 1H), 8.80 (dd, *J* = 8.1, 1.5 Hz, 1H), 8.66 (dd, *J* = 8.1, 1.3 Hz, 1H), 8.58 (d, *J* = 8.8 Hz, 1H), 8.45 (d, *J* = 8.6 Hz, 1H), 8.32 (dd, *J* = 8.3, 1.7 Hz, 1H), 8.17 (d, *J* = 2.6 Hz, 1H), 7.82 (d, *J* = 8.6 Hz, 1H), 7.75 (ddd, *J* = 7.9, 7.0, 1.4 Hz, 1H), 7.70 (ddd, *J* = 8.5, 7.0, 1.7 Hz, 1H) 7.50 (dd, *J* = 8.2, 4.2 Hz, 1H), 7.40 (dd, *J* = 8.8, 2.5 Hz, 1H), 4.05 (s, 3H). ^13^C NMR (CDCl_3_, 101 MHz) δ 157.53, 150.69, 150.65, 150.39, 137.04, 136.50, 130.10, 129.97, 127.46, 127.00, 126.84, 125.34, 124.80, 123.47, 123.44, 122.64, 122.53, 122.32, 120.91, 120.80, 116.83, 115.23, 106.19, 55.63. HRMS (ESI) *m*/*z* calculated [M + H]^+^ C_24_H_16_NO_2_^+^ 350.1103, found 350.1110.
*9-Chlorophenanthro[9′,10′:4,5]furo[3,2-h]quinoline* (**5Bc**). White solid. M.p. 211.4–212.9 °C (taken up with EP/DCM). ^1^H NMR (CDCl_3_, 400 MHz) δ 9.11 (d, *J* = 2.6 Hz, 1H), 8.81 (d, *J* = 7.8 Hz, 1H), 8.72 (d, *J* = 2.2 Hz, 1H), 8.66 (d, *J* = 8.1 Hz, 1H), 8.58 (d, *J* = 8.6 Hz, 1H), 8.44 (d, *J* = 8.6 Hz, 1H), 8.34 (dd, 1H), 7.84 (d, *J* = 8.4 Hz, 1H), 7.82–7.66 (m, 3H), 7.53 (dd, *J* = 8.3, 4.3 Hz, 1H). ^13^C NMR (CDCl_3_, 101 MHz) δ 151.83, 150.84, 150.53, 137.00, 136.55, 131.51, 129.93, 129.53, 128.05, 127.84, 127.64, 126.99, 126.59, 125.38, 124.51, 123.86, 123.76, 123.49, 122.61, 122.36, 121.16, 120.56, 114.85. HRMS (ESI) *m*/*z* calculated [M + H]^+^ C_23_H_13_ClNO^+^ 354.0607, found 354.0601.
*Chryseno[5′,6′:4,5]furo[3,2-h]quinoline* (**5Bd**). White solid. M.p. 261.1–262.9 °C (XX MeOH). ^1^H NMR (CDCl_3_, 400 MHz) δ 9.18–9.09 (m, 2H), 8.99–8.91 (m, 1H), 8.82–8.76 (m, 1H), 8.74 (d, *J* = 9.0 Hz, 1H), 8.65 (d, *J* = 8.8 Hz, 1H), 8.33 (dd, *J* = 8.2, 1.7 Hz, 1H), 8.07 (d, *J* = 8.7 Hz, 1H), 8.03 (d, *J* = 8.9 Hz, 1H), 7.83–7.67 (m, 5H), 7.53 (dd, *J* = 8.2, 4.3 Hz, 1H). ^13^C NMR (CDCl_3_, 101 MHz) δ 153.35, 150.96, 150.70, 137.22, 136.38, 132.94, 130.47, 129.84, 128.10, 127.66, 127.22, 127.08, 126.89, 126.83, 126.27, 125.94, 125.39, 125.16, 123.74, 122.88, 122.50, 122.17, 121.71, 121.21, 115.75 (two signals not visible because of isochrony). HRMS (ESI) *m*/*z* calculated [M + H]^+^ C_27_H_16_NO^+^ 370.1154, found 370.1158.
*Thieno[2″,3″:3′,4′]naphtho [2′,1′:4,5]furo[3,2-h]quinoline* (**5Be**). White solid. M.p. 232.5–233.7 °C (taken up with EP/DCM). ^1^H NMR (CDCl_3_, 400 MHz) δ 9.12 (dd, *J* = 4.3, 1.7 Hz, 1H), 8.90–8.81 (m, 1H), 8.50–8.41 (m, 1H), 8.35 (dd, *J* = 8.3, 1.7 Hz, 1H), 8.26 (d, *J* = 8.5 Hz, 1H), 8.11 (d, *J* = 5.3 Hz, 1H), 7.87 (d, *J* = 8.5 Hz, 1H), 7.76–7.67 (m, 2H), 7.66 (d, *J* = 5.3 Hz, 1H), 7.53 (dd, *J* = 8.2, 4.3 Hz, 1H). ^13^C NMR (CDCl_3_, 101 MHz) δ 151.13, 150.76, 150.48, 136.96, 136.75, 133.58, 130.11, 128.79, 127.46, 127.04, 126.11, 124.44, 124.20, 123.79, 123.50, 122.50, 122.33, 121.08, 120.37, 120.01, 115.07. HRMS (ESI) *m*/*z* calculated [M + H]^+^ C_21_H_12_NOS^+^ 326.0561, found 326.0558.
*9-Methyl-6H-phenanthro[9′,10′:4,5]furo[3,2-c]chromen-6-one* (**5Ca**). White solid. M.p. 265.0–266.0 °C (taken up with EP/DCM). ^1^H NMR (CDCl_3_, 400 MHz) δ 9.57 (d, *J* = 8.4 Hz, 1H), 8.64 (dd, *J* = 7.0, 2.3 Hz, 1H), 8.42 (s, 1H), 8.35 (dd, *J* = 7.5, 1.9 Hz, 1H), 8.09 (dd, *J* = 7.8, 1.6 Hz, 1H), 7.74–7.62 (m, 2H), 7.62–7.53 (m, 2H), 7.50 (dd, *J* = 8.5, 1.1 Hz, 1H), 7.42 (t, *J* = 7.5 Hz, 1H), 2.62 (s, 3H). ^13^C NMR (CDCl_3_, 101 MHz) δ 158.74, 158.61, 153.14, 150.02, 136.26, 131.34, 130.17, 129.38, 128.86, 128.07, 127.24, 124.67, 124.42, 123.51, 123.02, 121.57, 121.16, 120.83, 117.25, 117.19, 112.77, 108.51, 22.20 (two isochronous carbons). HRMS (ESI) *m*/*z* calculated [M + H]^+^ C_24_H_15_O_3_^+^ 351.0943, found 351.0950.
*9-Methoxy-6H-phenanthro[9′,10′:4,5]furo[3,2-c]chromen-6-one* (**5Cb**). White solid. M.p. 250.0–251.0 °C (taken up with EP/DCM). ^1^H NMR (CDCl_3_, 400 MHz) δ 9.59 (d, *J* = 9.0 Hz, 1H), 8.54 (d, *J* = 8.0 Hz, 1H), 8.32 (dd, *J* = 7.5, 1.8 Hz, 1H), 8.07 (dd, *J* = 7.9, 1.6 Hz, 1H), 7.99 (d, *J* = 2.6 Hz, 1H), 7.69–7.60 (m, 2H), 7.58 (td, *J* = 7.9, 7.2, 1.6 Hz, 1H), 7.49 (d, *J* = 8.3 Hz, 1H), 7.42 (t, *J* = 7.5 Hz, 1H), 7.34 (dd, *J* = 9.0, 2.6 Hz, 1H), 4.01 (s, 3H). ^13^C NMR (CDCl_3_, 101 MHz) δ 158.70, 158.67, 158.21, 153.10, 149.24, 131.33, 130.39, 129.75, 127.46, 127.06, 124.68, 123.54, 121.55, 121.32, 120.86, 117.27, 117.18, 116.60, 112.77, 108.34, 105.55, 55.53 (two signals not visible because of isochrony). HRMS (ESI) *m*/*z* calculated [M + H]^+^ C_24_H_15_O_4_^+^ 367.0892, found 367.0898.
**Crystal structure of 3Ca**
A single crystal of compound **3Ca** was submitted to X-ray data collection on a Bruker APEX-II CCD diffractometer (Billerica, MA, USA) with a graphite monochromated Cu-Kα radiation (λ = 1.5418 Å) at 100 K. The structure was solved by direct methods implemented in the SHELXS-97 program [39]. The refinement was carried out by full-matrix anisotropic least squares on F 2 for all reflections for non-H atoms using the SHELXL-97 program (Version 2019/2) [40]. **3Ca** crystallizes with a molecule of ethanol. Crystallographic data for this structure have been deposited at the Cambridge Crystallographic Data Centre as supplementary publication no. CCDC 2418973. Copies of the data can be obtained, free of charge, by application to CCDC, 12 Union Road, Cambridge CB2 1EZ, UK; (fax: + 44-(0)-1223-336-033; or e-mail: deposit@ccdc.cam.ac.uk).

## 4. Conclusions

By exploiting the double-coupling reaction between twofold (C/C) electrophilic molecules such as nitrostilbenes **1** and twofold (C/O) nucleophiles provided by an enol fragment within an aromatic phenolic ring such as **B**–**E** (Scheme 1 and Scheme 2 and Figure 2), a successful and quite robust synthesis of structurally different fused heteropolycycles, characterized by the presence of *O* or *O* and *N* heteroatoms, has been accomplished.

Following the initial double coupling to DHFs, further steps, namely, oxidative (DDQ) aromatization of the furan ring, 6π-electrocyclization, and full aromatization via methanesulfinic acid elimination, allow access to more complex, completely conjugated fused systems. For some of these systems, a significant fluorescence has been observed and measured and is applicable, e.g., in the field of optical devices.

Furthermore, on the grounds of both chemical diversity and/or spatial arrangement, biological/pharmacological activities are envisaged for at least some classes of molecules herein, which could also become leads for the design of new drugs. As a matter of fact, preliminary studies, in particular on furocoumarins, are quite promising, with results that still need completion and refinement and will be published in due time.

On the synthetic side, refined quantomechanical calculations fully rationalize on the grounds of both steric and electronic factors, in particular the complete failure of aromatization of **2Cd** to **3Cd**: a surely quite surprising result if compared, e.g., to the easy aromatization (in turn perfectly supported by quantomechanical calculations) of the **2Ad** and **2Bd** congeners.

## Figures and Tables

**Scheme 10:**
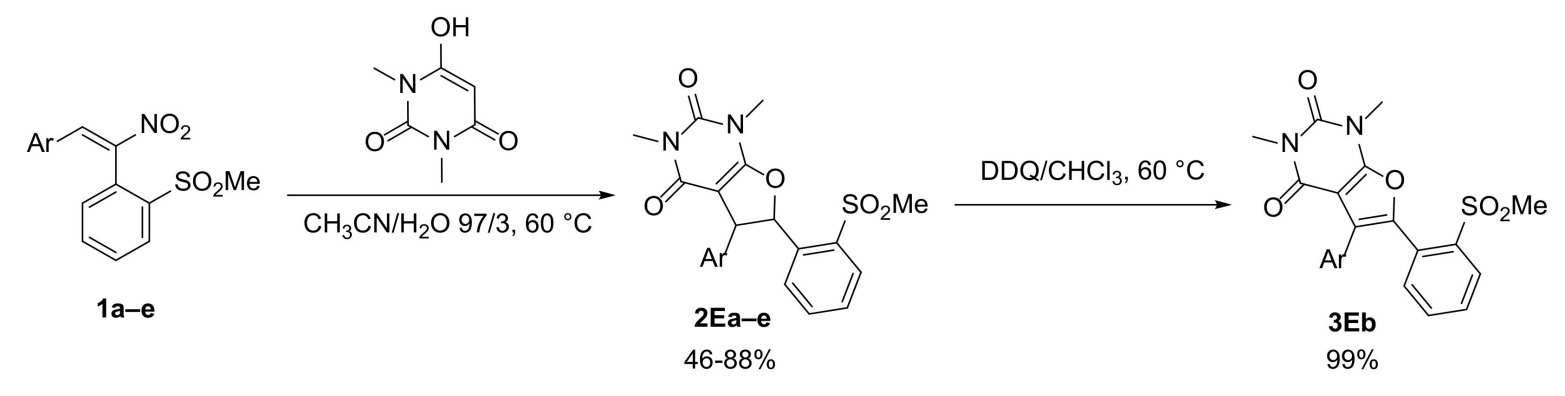
Synthesis of **2E** and **3E** from the initial coupling of **1a**–**e** and **E**.

**Table 1:** Results obtained in the reactions of **1a**–**e** with 8-hydroxyquinoline in the presence of K_2_CO_3_ (1.2 equiv.), in DMSO at room T (Scheme 3).

Ar in 1		t	Yields in 2Ba–e	t ^a^	Yields in 2Ba–c ^a^
*p*-Tolyl	**a**	24 h	70%	4 h	90%
*p*-MeO-Phenyl	**b**	24 h	66%	4 h	58%
*p*-Cl-Phenyl	**c**	24 h	74%	4 h	81%
1-Naphthyl	**d**	45 h	30%		
2-Thienyl	**e**	24 h	40%		

^a^ Data from ref. [8].

**Table 2:** Oxidative aromatization of **2Ba**–**e** to furoquinolines **3Ba**–**e** with DDQ (2 mol equiv.) in CHCl_3_ (Scheme 4).

Ar in 2B		t	T	Yields in 3Ba–e
*p*-Tolyl	**a**	24 h	r.t.	91%
*p*-MeO-Phenyl	**b**	24 h	r.t.	90%
*p*-Cl-Phenyl	**c**	27 h	r.t.	73%
1-Naphthyl	**d**	30 h	60 °C	85%
2-Thienyl	**e**	24 h	r.t.	99%

**Table 3:** Results obtained in the reactions of **1a**–**e** with 4-hydroxycoumarin, in Et_3_N/CHCl_3_ at 60 °C for 24 h.

Ar in 1		Yields in 2Ca–e
*p*-Tolyl	**a**	79%
*p*-MeO-Phenyl	**b**	68%
*p*-Cl-Phenyl	**c**	83%
1-Naphthyl	**d**	76%
2-Thienyl	**e**	10% ^a^

^a^ A different reaction took over: see the text below.

**Table 4:** Results in reactions of **1c**–**e** with 4-hydroxycoumarin in DABCO/EtOH at reflux.

Ar in 1		t	Yields in 2Ce,c,d
2-Thienyl	**e**	16 h	74%
*p*-Cl-Phenyl	**c**	4 h	70%
1-Naphthyl	**d**	24 h	76%

**Table 5:** Search for the best conditions for the aromatization of **2Ca** to furocoumarin **3Ca** with DDQ.

Solvent	DDQ mol equiv.	T	t	Yields in 3Ca
CHCl_3_	2	r.t.	24 h	14%
CHCl_3_	3	60 °C	48 h	40%
Toluene	8	110 °C	28 h	12%
CHCl_3_	4	60 °C	5 days	90%

**Table 6:** Oxidative aromatization of **2Ca**–**e** to furocoumarins **3Ca**–**e** with DDQ (4 mol equiv.) in CHCl_3_ at 60 °C.

Ar in 2C		t	Yields in 3Ca–e
*p*-Tolyl	**a**	96 h	99%
*p*-MeO-Phenyl	**b**	48 h	99%
*p*-Cl-Phenyl	**c**	11 days	42% ^a^
1-Naphthyl	**d**	28 h	0%
2-Thienyl	**e**	24 h	99%

^a^ Not isolated. Yields evaluated by ^1^HNMR analysis of chromatographically isolated mixtures with **2Cc**.

**Table 7:** Results obtained in the reactions of **1a**–**e** with 5-hydroxyindole in CH_3_CN/H_2_O 97/3 at 60 °C, followed by complete aromatization of DHFs **2D** in DDQ/toluene at r.t. for 10 min. (see Scheme 9).

		CH_3_CN/H_2_O 97/3, 60 °C					DDQ/Tol, r.t. 10 min
Ar in 1		t	2D	3D	Ar in 2D		3D
*p*-Tolyl	**a**	16 h	76%	12%	*p*-Tolyl	**a**	93%
*p*-MeO-Phenyl	**b**	40 h	66%	12%	*p*-MeO-Phenyl	**b**	99%
*p*-Cl-Phenyl	**c**	5 h	44%	20%	*p*-Cl-Phenyl	**c**	98%
1-Naphthyl	**d**	48 h	60%	10%	1-Naphthyl	**d**	97%
2-Thienyl	**e**	52 h	61%	16%	2-Thienyl	**e**	99%

**Table 8:** Results obtained in the reactions of 1a–e with *N,N*′-dimethylbarbituric acid in CH_3_CN/H_2_O 97/3 at 60 °C for 24 h and of the following aromatization of the representative 2Eb in DDQ/CHCl_3_ at 60 °C (see Scheme 10).

		CH_3_CN/H_2_O 97/3, 60 °C, 24 h			DDQ/CHCl_3_, 60 °C, 29 h
Ar in 1		2E	Ar in 2E		3E
*p*-Tolyl	**a**	68%			
*p*-MeO-Phenyl	**b**	48%	*p*-MeO-Phenyl	**b**	99%
*p*-Cl-Phenyl	**c**	71%			
1-Naphthyl	**d**	88%			
2-Thienyl	**e**	46% *			

* Not isolated. Yields evaluated by ^1^HNMR analysis of chromatographically isolated mixtures with an unknown product.

**Table 9:** 6π-Electrocyclization, followed by aromatization, of **3Ba**–**e** to compounds **5Ba**–**e** in acetone in a Rayonet photochemical reactor.

Ar in 3B		hν	t	5B	hν ^a^	t ^a^	5B
*p*-Tolyl	**a**	300	24 h	52%	350	16 h	70%
*p*-MeO-Phenyl	**b**	300	24 h	29%	350	16 h	73%
*p*-Cl-Phenyl	**c**	300	24 h	57%	350	17 h	62%
1-Naphthtyl	**d**	300	24 h		350	24 h	68%
2-Thyenyl	**e**	300	24 h		350	16 h	50%

^a^ In the presence of 1 mol equiv. of DBU.

**Table 10:** The 6π-electrocyclization reaction of **3Ca**,**b**,**e** followed by oxidative aromatizatio to generate compounds **5Ca**,**b**,**e** (acetone solution, 1 mol equiv. of DBU, irradiation at 350 nm for 16 h).

Ar in 3C		5C
*p*-Tolyl	**a**	70%
*p*-MeO-Phenyl	**b**	73%
2-Thienyl	**e**	65% ^a^

^a^ We could only assume that the yield refers to the expected product because the reaction proceeded similarly to the other terms: unfortunately, any attempt to characterize the product failed due to its insolubility in any solvent tested.

**Table 11:** Fluorescence data for quinoline derivatives **2Bb**, **3Bb**–**e** and **5Ba**–**e**; and for coumarin derivatives **3Ca**,**b**,**e** and **5Ca**,**b**.

Compound	λ_max_ (ab) [nm]	λ_max_ (em) [nm]	$\Delta \bar{\nu }$ [cm^−1^]	ϕ
**2Bb**	330	411	5972	0.037
**3Bb**	329	433	7300	0.160
**3Bc**	324	396	5612	0.200
**3Bd**	297	407	9100	0.410
**3Be**	325	434	7728	0.151
**5Ba**	327	420	6772	0.694
**5Bb**	328	442	7863	0.459
**5Bc**	326	407	6105	0.258
**5Bd**	305	407	8217	0.712
**5Be**	290	423	10,842	0.478
**3Ca**	334	405	5249	0.206
**3Cb**	341	438	6494	0.109
**3Ce**	332	442	7496	0.126
**5Ca**	364	412	3201	0.427
**5Cb**	371	441	4278	0.329

## Data Availability

Data are contained within the article and Supplementary Materials. **3Ca** crystallizes with a molecule of ethanol. Crystallographic data for this structure have been deposited at the Cambridge Crys-tallographic Data Centre as supplementary publication no. CCDC 2418973. Copies of the data can be obtained, free of charge, by application to CCDC, 12 Union Road, Cambridge CB2 1EZ, UK; (fax: + 44-(0)-1223-336-033; or e-mail: depos-it@ccdc.cam.ac.uk).

## Supplementary Materials

The following supporting information can be downloaded at: https://www.mdpi.com/article/10.3390/molecules30040948/s1. ^1^H and ^13^C NMR spectra for all compounds and high-resolution HOMO surfaces for all computed model compounds.
