# Supplementary material for: An Appealing, Robust Access to Furo-Fused Heteropolycycles
Source: Molecules. 2025 Feb 18;30(4):948. doi: 10.3390/molecules30040948 (PMC11858213; doi:10.3390/molecules30040948)
Supplement: Supplementary file 1 [file molecules-30-00948-s001.zip › molecules-3328989-supplementary.pdf]

Supplementary Materials

An appealing, robust access to furo-fused heteropolycycles

Alice Benzi[1], Lara Bianchi[1], Gianluca Giorgi[2], Giovanni Lentini[3], Massimo Maccagno[1], Guglielmo Marcantoni-Taddei[1], Giovanni Petrillo[1], and Cinzia Tavani[1].

1 - Department of Chemistry and Industrial Chemistry (DCCI), University of Genova, Via Dodecaneso 31, 16146 Genova, Italy; alice.benzi93@gmail.com (A.B.); lara.bianchi@unige.it (L.B.); massi-mo.maccagno@unige.it (M.M.); giovanni.petrillo@unige.it (G.P.); cinzia.tavani@unige.it (C.T.)
2 - Department of Biotechnology, Chemistry and Pharmacy, University of Siena, Via A. Moro, 53100 Siena, Ita-ly; gianluca.giorgi@unisi.it
3 - Department of Pharmacy - Pharmaceutical Sciences "Aldo Moro", University of Bari, Via E. Orabona 4, 70125 Bari, Italy; giovanni.lentini@uniba.it

*Correspondence: lara.bianchi@unige.it

Table of Contents

High resolution HOMO surfaces for all computed model compounds S-2

^{1}H and ^{13}C NMR spectra for all compounds S-5

**2Ad**	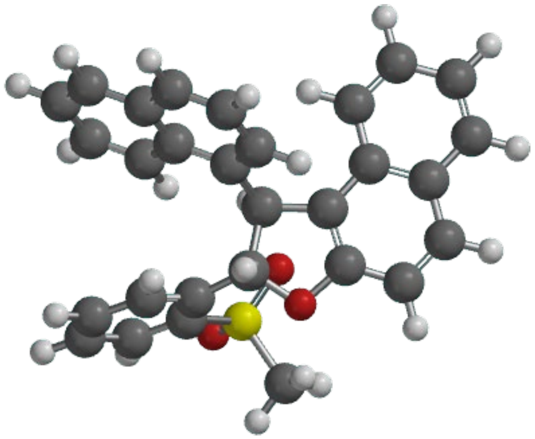	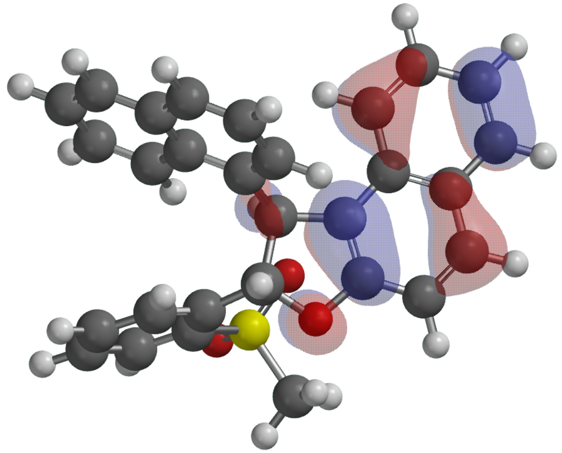	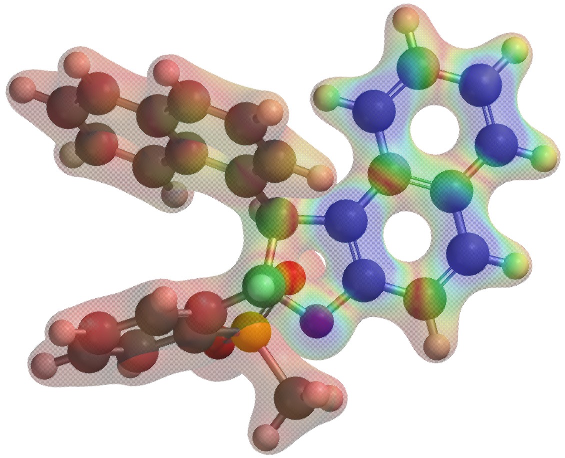
**2Bd**	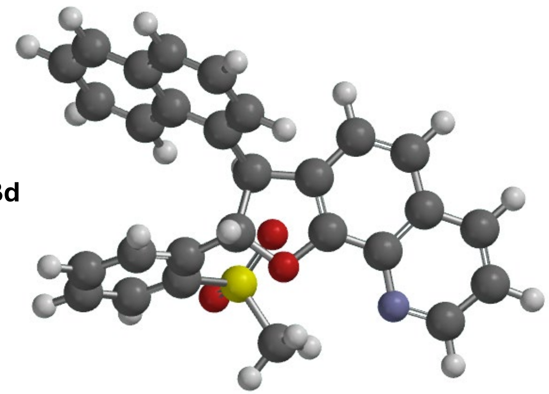	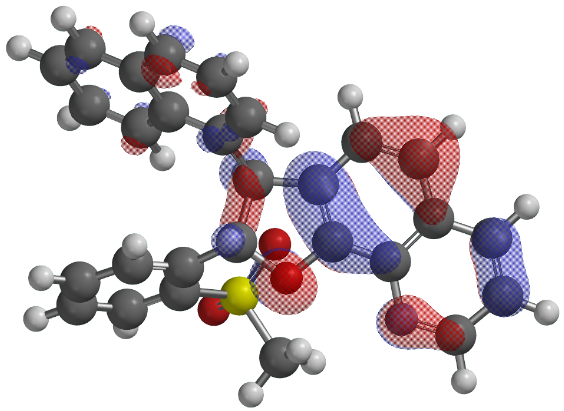	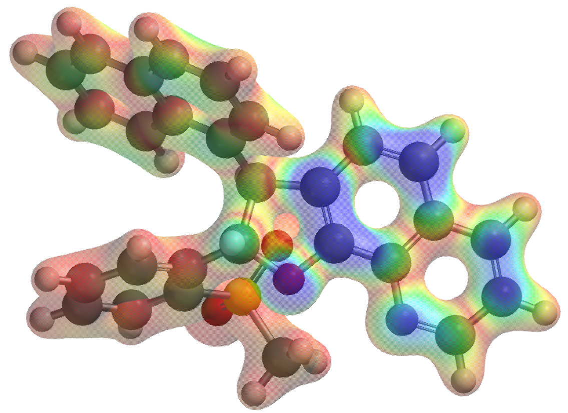
**2Cd**	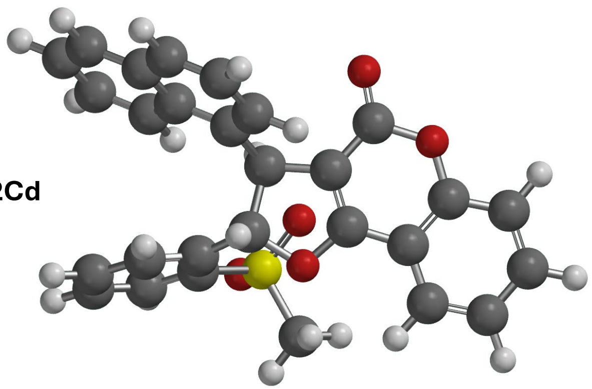	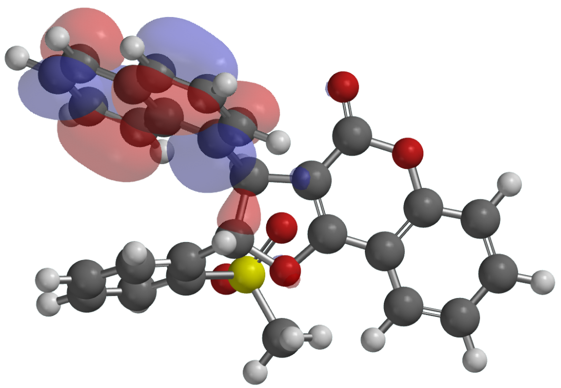	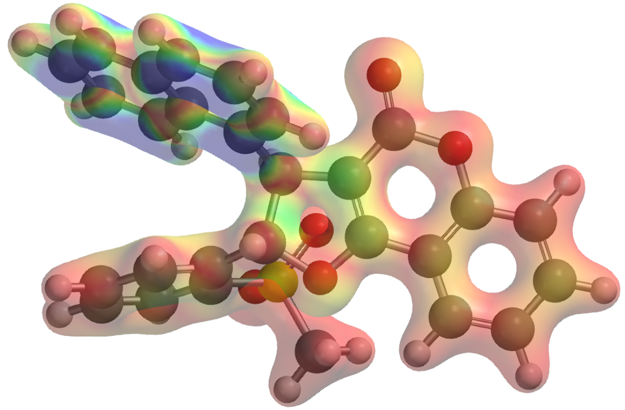

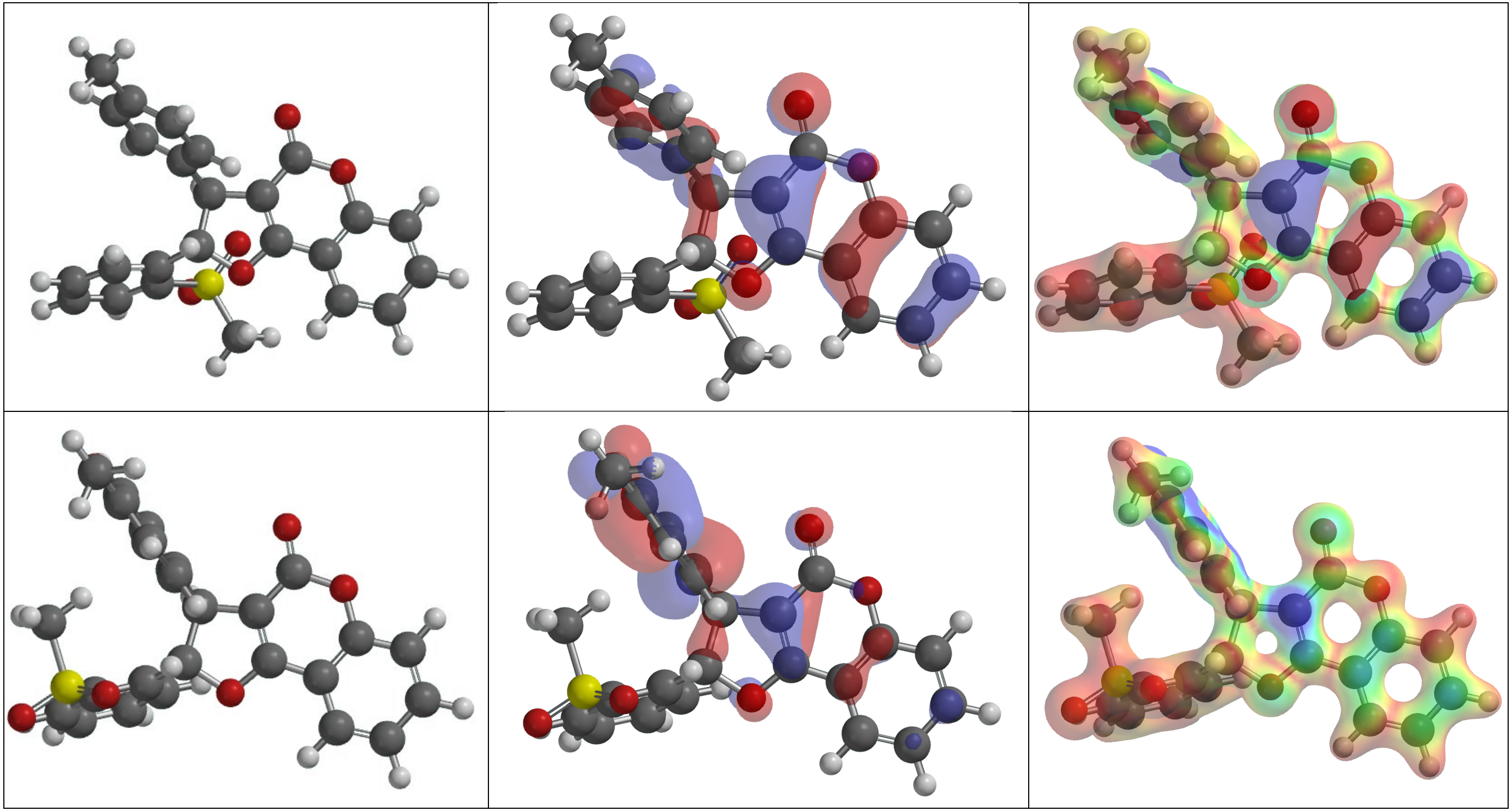

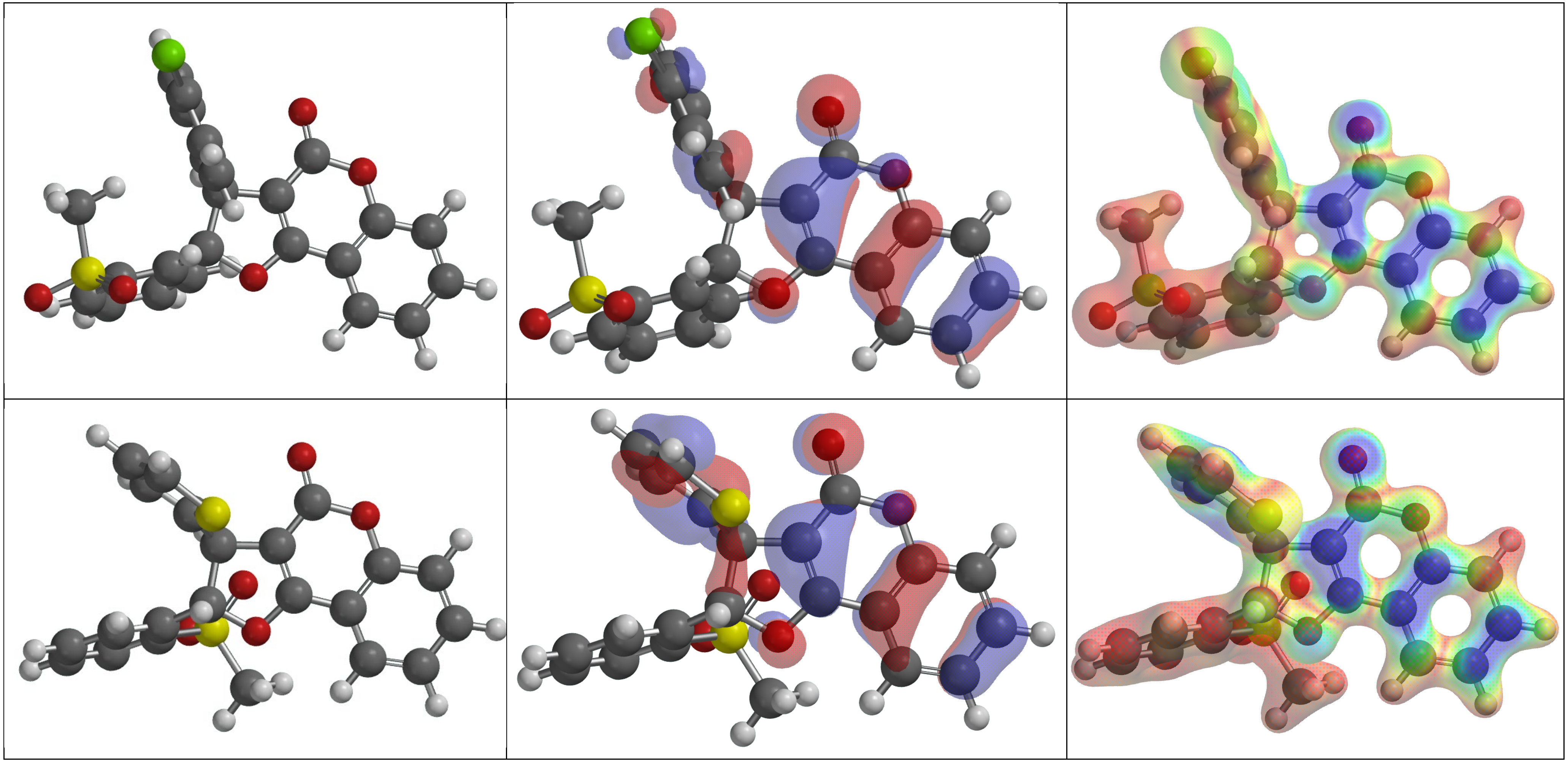

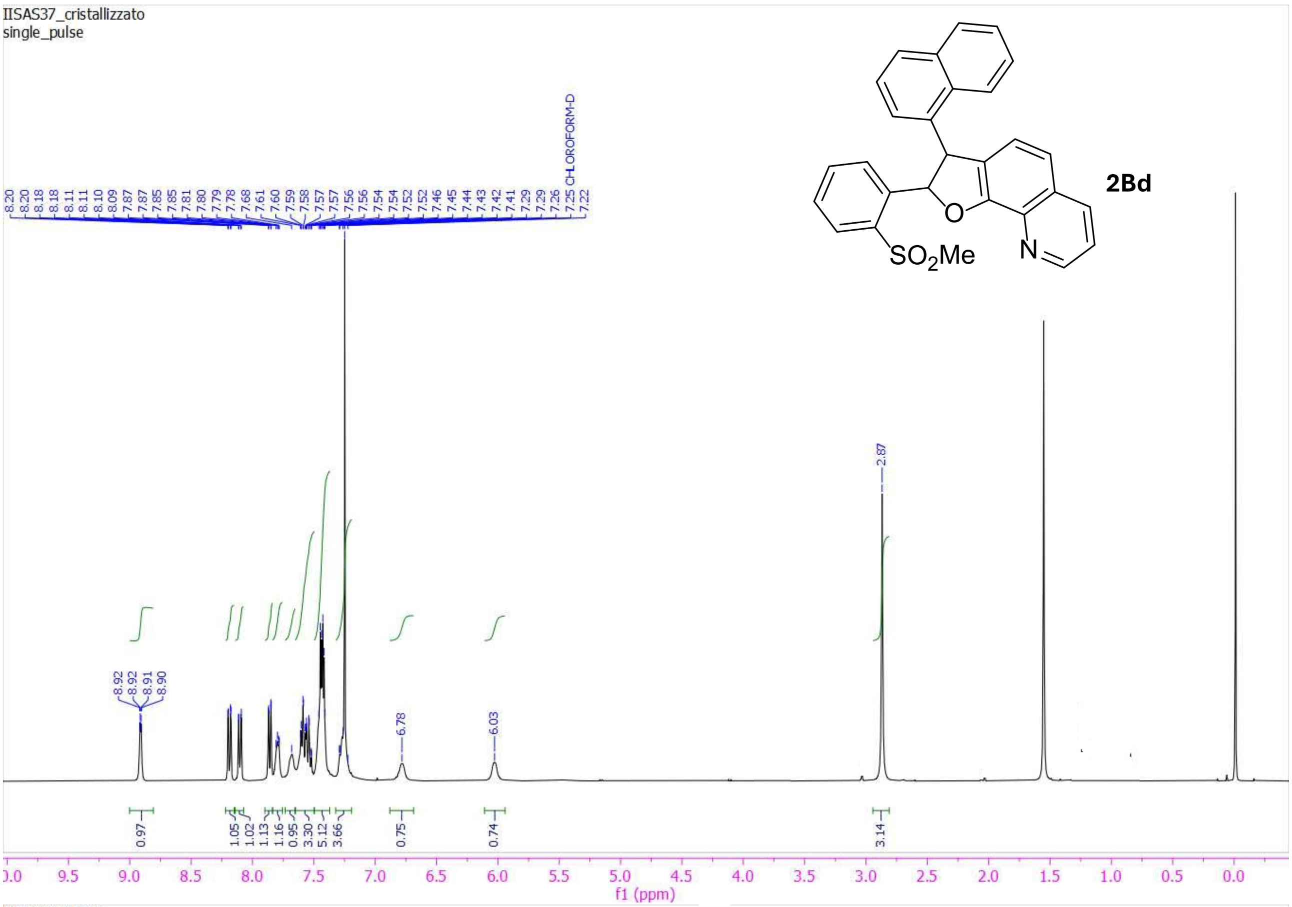
IISAS37_cristallizzato
single_pulse
2Bd
SO2Me
O
N
CHLOROFORM-D
8.20 8.20 8.18 8.11 8.11 8.10 8.09 7.87 7.87 7.85 7.85 7.81 7.80 7.79 7.78 7.68 7.61 7.60 7.59 7.58 7.57 7.57 7.56 7.56 7.54 7.54 7.52 7.52 7.46 7.45 7.44 7.43 7.42 7.41 7.29 7.29 7.26 7.25 7.22
8.92 8.92 8.91 8.90
6.78
6.03
2.87
0.97 1.05 1.02 1.13 1.16 0.95 3.30 5.12 3.66 0.75 0.74 3.14
f1 (ppm)

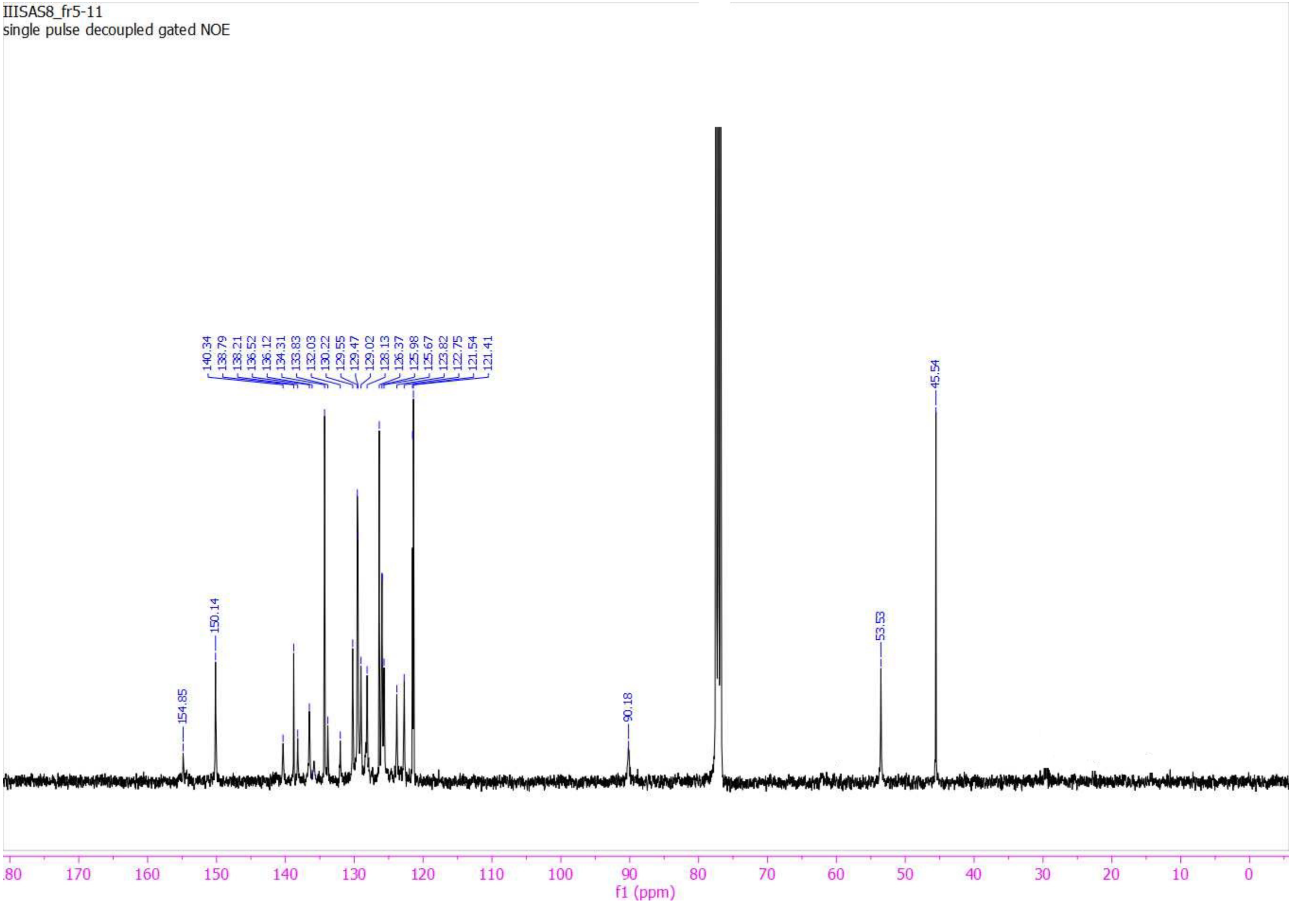
IIISAS8_fr5-11
single pulse decoupled gated NOE
140.34 138.79 138.21 136.52 136.12 134.31 133.83 132.03 130.22 129.55 129.47 129.02 128.13 126.37 125.98 125.67 123.82 122.75 121.54 121.41
154.85
150.14
90.18
53.53
45.54
f1 (ppm)

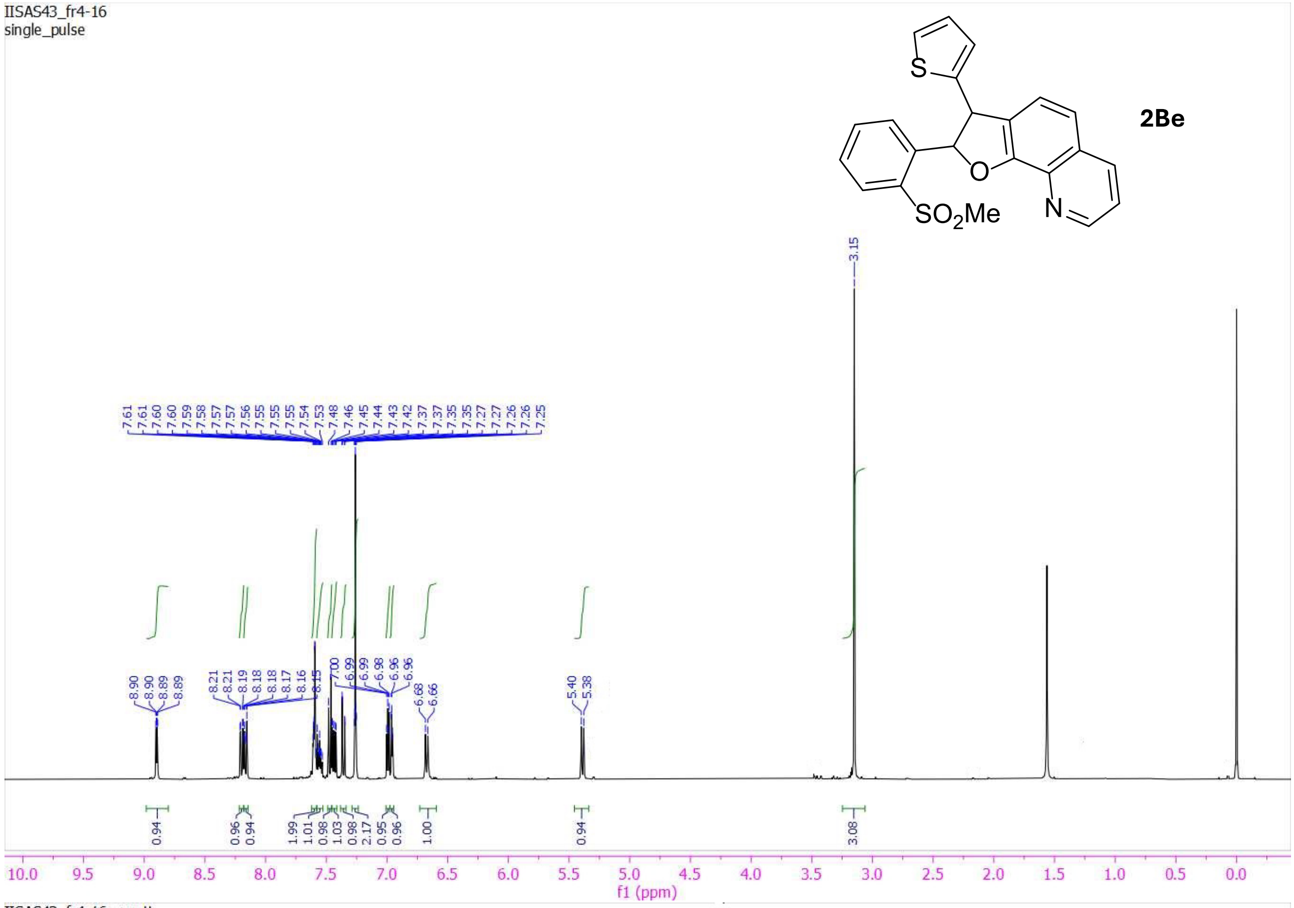
IISAS43_fr4-16
single_pulse
2Be
SO2Me
S
O
N
7.61
7.61
7.60
7.60
7.59
7.58
7.57
7.57
7.56
7.55
7.55
7.55
7.54
7.53
7.48
7.46
7.45
7.44
7.43
7.42
7.37
7.37
7.35
7.35
7.27
7.27
7.26
7.26
7.25
3.15
8.90
8.90
8.89
8.89
8.21
8.21
8.19
8.18
8.18
8.17
8.16
8.15
7.00
6.99
6.99
6.98
6.98
6.96
6.96
6.68
6.66
5.40
5.38
0.94
0.96
0.94
1.99
1.01
0.98
1.03
0.98
2.17
0.95
0.96
1.00
0.94
3.08
f1 (ppm)

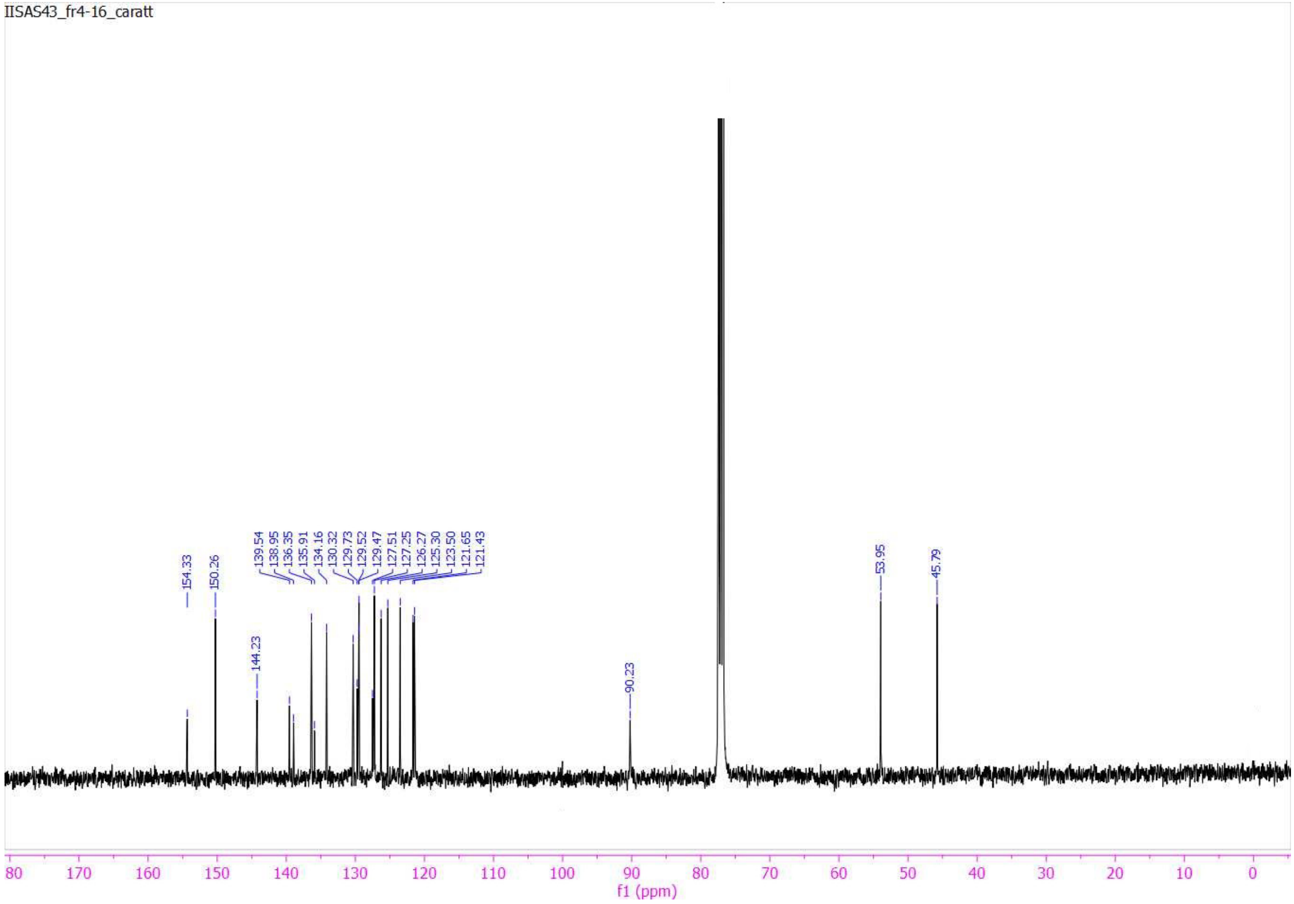
IISAS43_fr4-16_caratt
154.33
150.26
144.23
139.54
138.95
136.35
135.91
134.16
130.32
129.73
129.52
129.47
127.51
127.25
126.27
125.30
123.50
121.65
121.43
90.23
53.95
45.79
f1 (ppm)

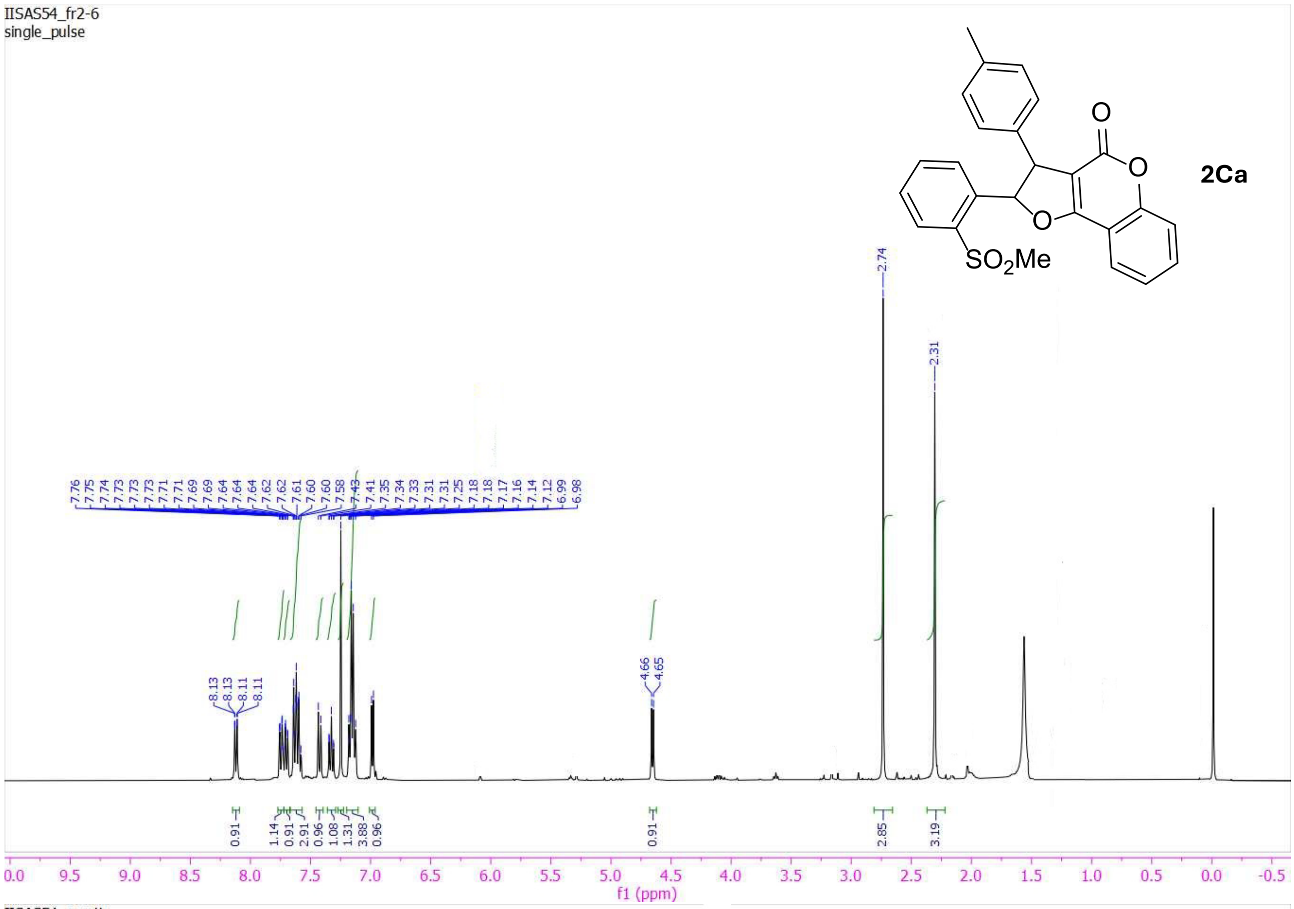
IISAS54_fr2-6
single_pulse
2Ca
SO2Me
7.76 7.75 7.74 7.73 7.73 7.73 7.71 7.71 7.69 7.64 7.64 7.64 7.62 7.62 7.61 7.60 7.60 7.58 7.43 7.41 7.35 7.34 7.33 7.31 7.31 7.25 7.18 7.18 7.17 7.16 7.14 7.12 6.99 6.98
8.13 8.13 8.11 8.11
4.66 4.65
2.74
2.31
0.91 1.14 0.91 2.91 0.96 1.08 1.31 3.88 0.96 0.91 2.85 3.19
f1 (ppm)

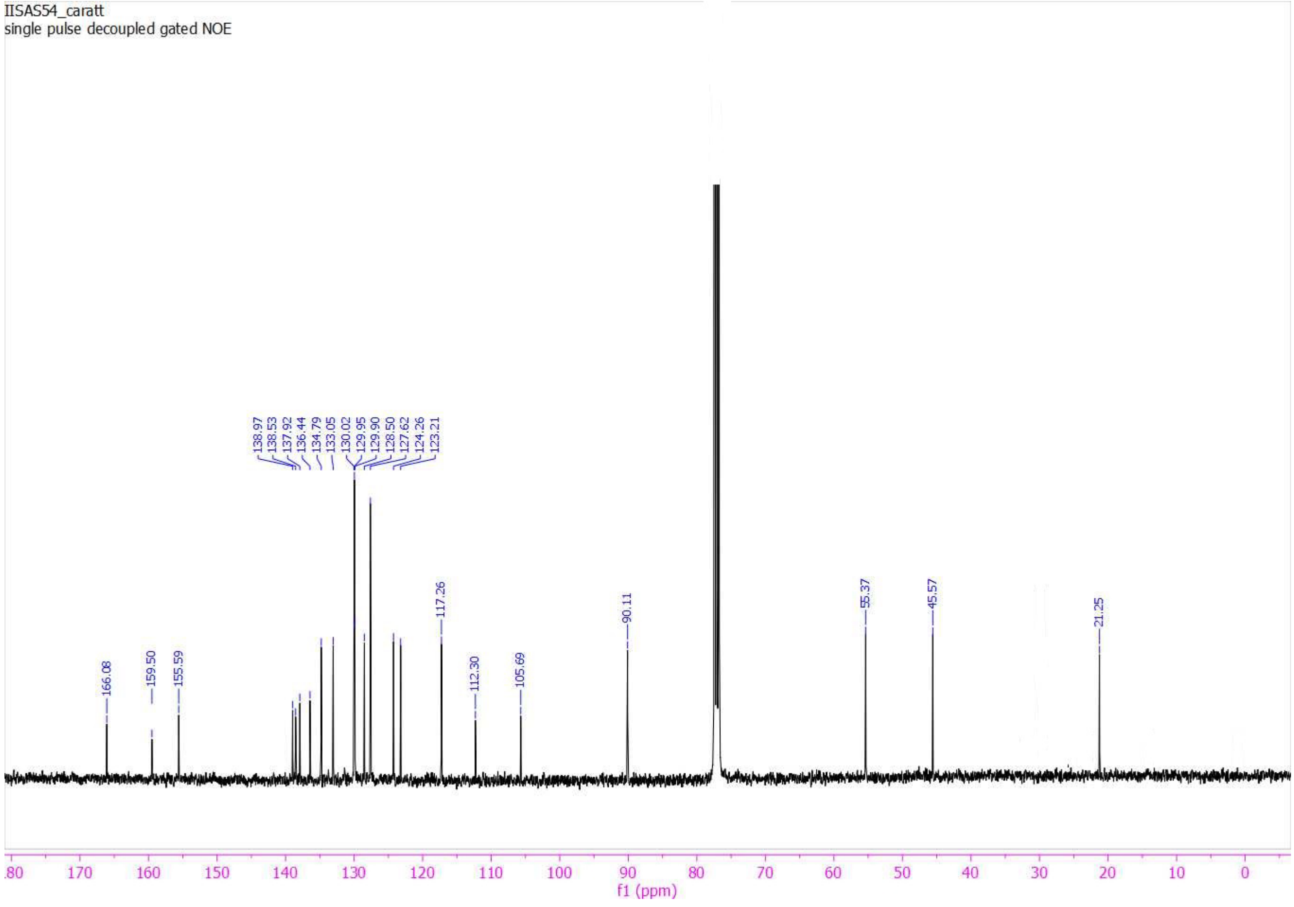
IISAS54_caratt
single pulse decoupled gated NOE
138.97 138.53 137.92 136.44 134.79 133.05 130.02 129.95 129.90 128.50 127.62 124.26 123.21
166.08 159.50 155.59 117.26 112.30 105.69 90.11 55.37 45.57 21.25
f1 (ppm)

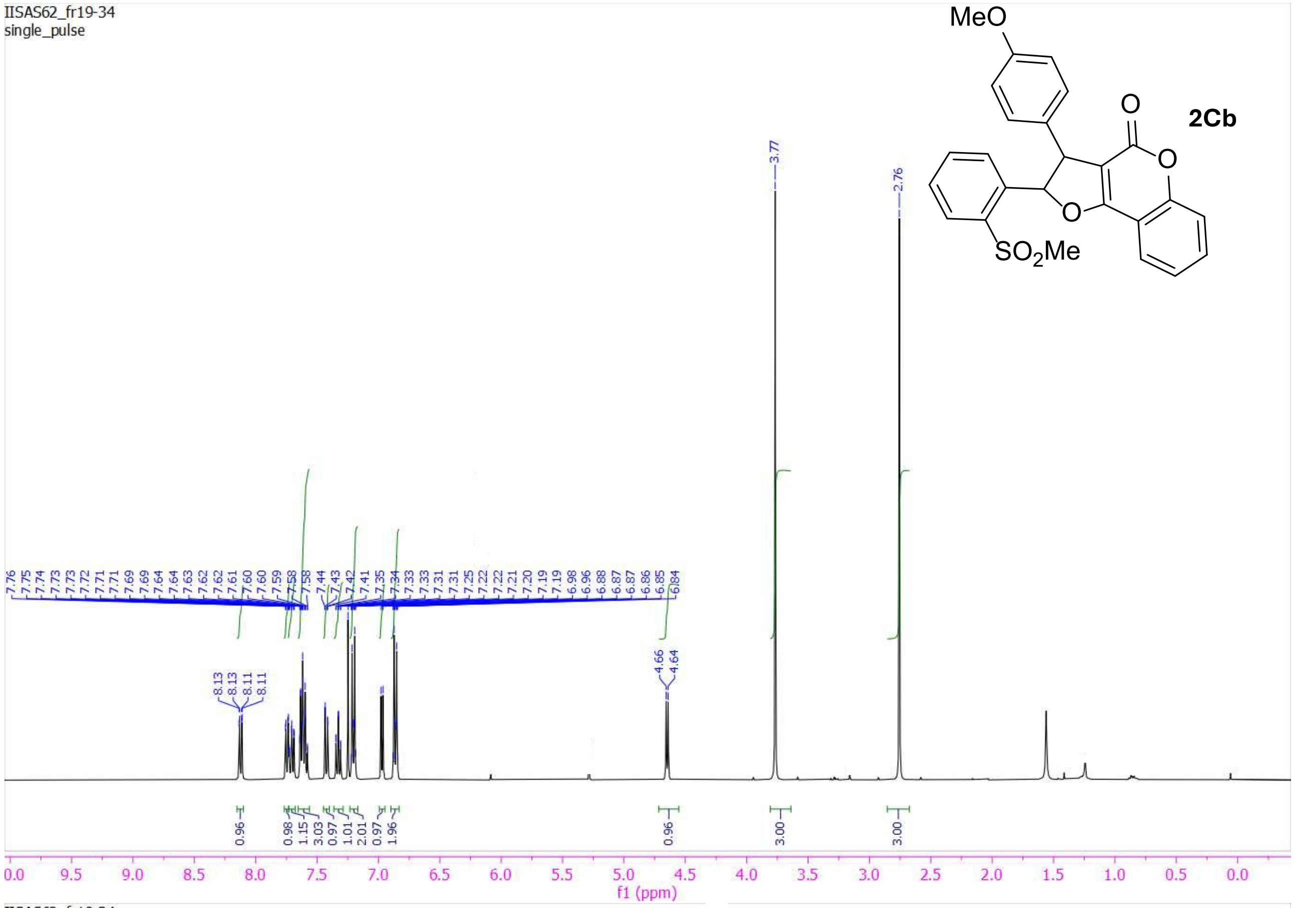
IISAS62_fr19-34
single_pulse
MeO
O
O
O
SO2Me
2Cb
7.76 7.75 7.74 7.73 7.73 7.72 7.71 7.71 7.69 7.69 7.64 7.64 7.63 7.62 7.62 7.61 7.60 7.60 7.59 7.58 7.58 7.44 7.43 7.42 7.41 7.35 7.34 7.33 7.33 7.31 7.31 7.25 7.22 7.22 7.21 7.20 7.19 6.98 6.96 6.88 6.87 6.87 6.86 6.85 6.84
8.13 8.13 8.11 8.11
4.66 4.64
3.77
2.76
0.96 0.98 1.15 3.03 0.97 1.01 2.01 0.97 1.96 0.96 3.00 3.00
f1 (ppm)

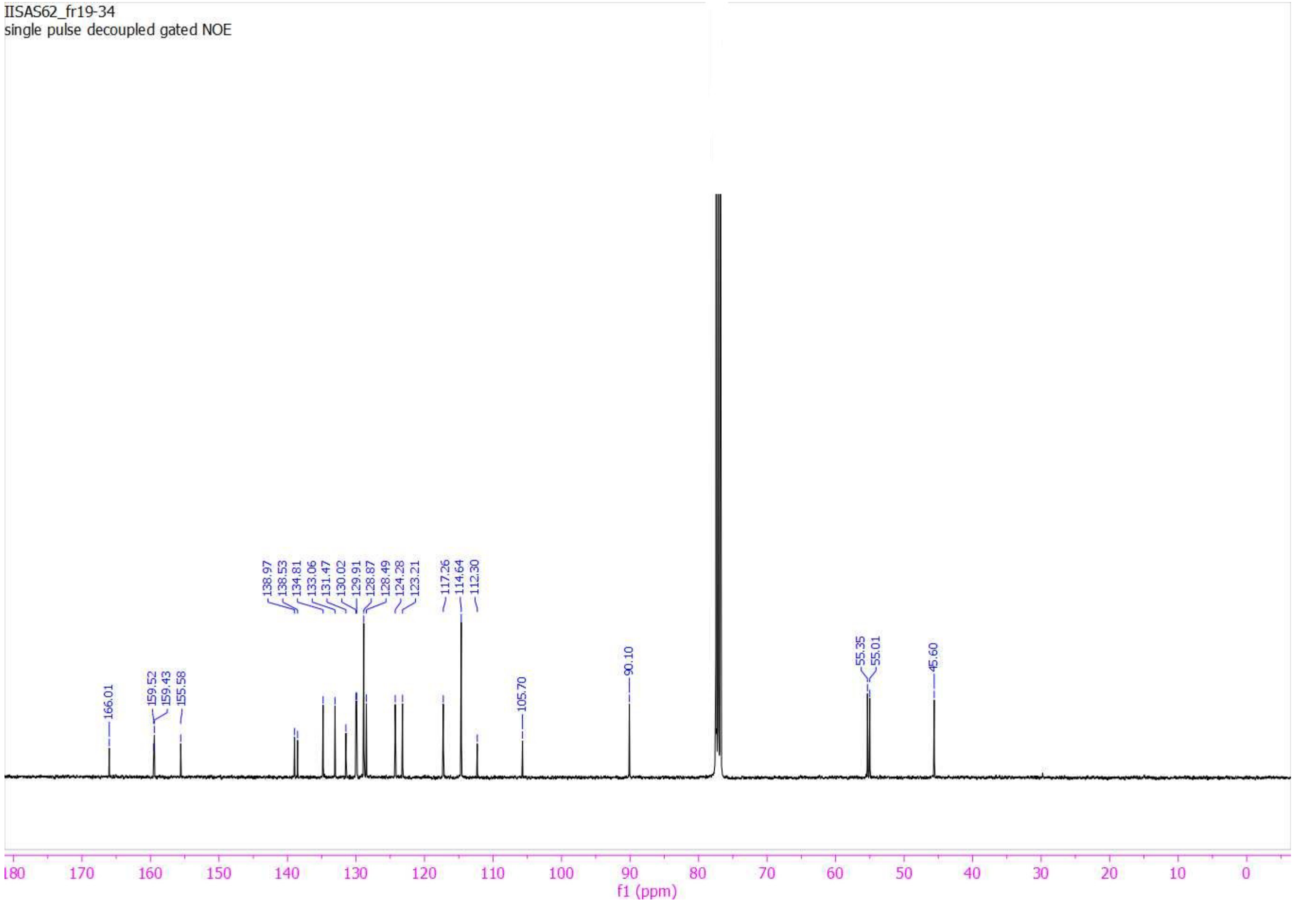
IISAS62_fr19-34
single pulse decoupled gated NOE
166.01
159.52 159.43 155.58
138.97 138.53 134.81 133.06 131.47 130.02 129.91 128.87 128.49 124.28 123.21
117.26 114.64 112.30
105.70
90.10
55.35 55.01
45.60
f1 (ppm)

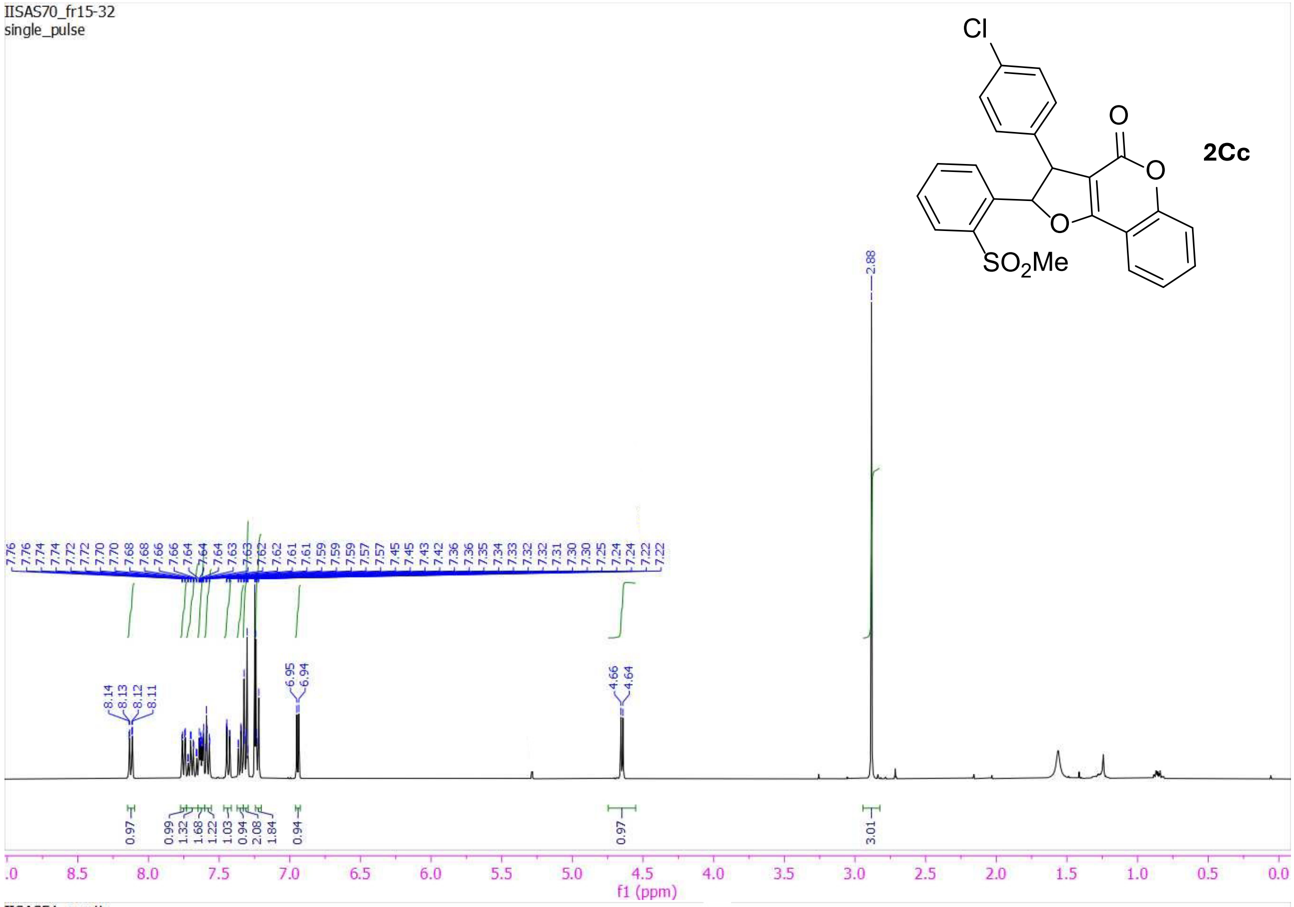
IISAS70_fr15-32
single_pulse
Cl
O
O
O
SO2Me
2Cc
7.76 7.76 7.74 7.74 7.72 7.72 7.70 7.70 7.68 7.68 7.66 7.66 7.64 7.64 7.64 7.63 7.63 7.62 7.62 7.61 7.61 7.59 7.59 7.59 7.57 7.57 7.45 7.45 7.43 7.42 7.36 7.36 7.35 7.34 7.33 7.32 7.32 7.31 7.30 7.30 7.25 7.24 7.24 7.22 7.22
8.14 8.13 8.12 8.11
6.95 6.94
4.66 4.64
2.88
0.97
0.99 1.32 1.68 1.22 1.03 0.94 2.08 1.84
0.94
0.97
3.01
f1 (ppm)

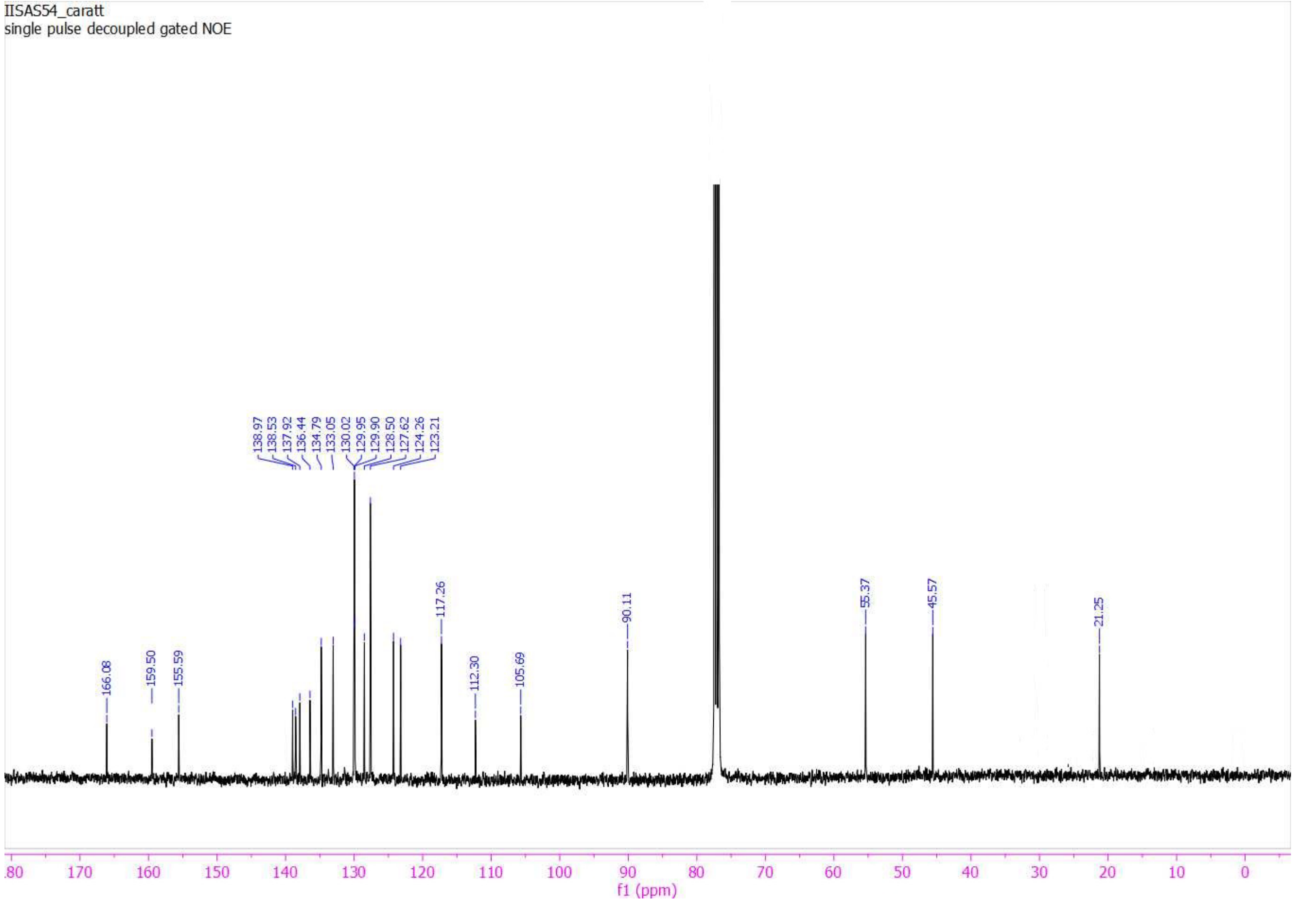
IISAS54_caratt
single pulse decoupled gated NOE
138.97 138.53 137.92 136.44 134.79 133.05 130.02 129.95 129.90 128.50 127.62 124.26 123.21
166.08
159.50
155.59
117.26
112.30
105.69
90.11
55.37
45.57
21.25
f1 (ppm)

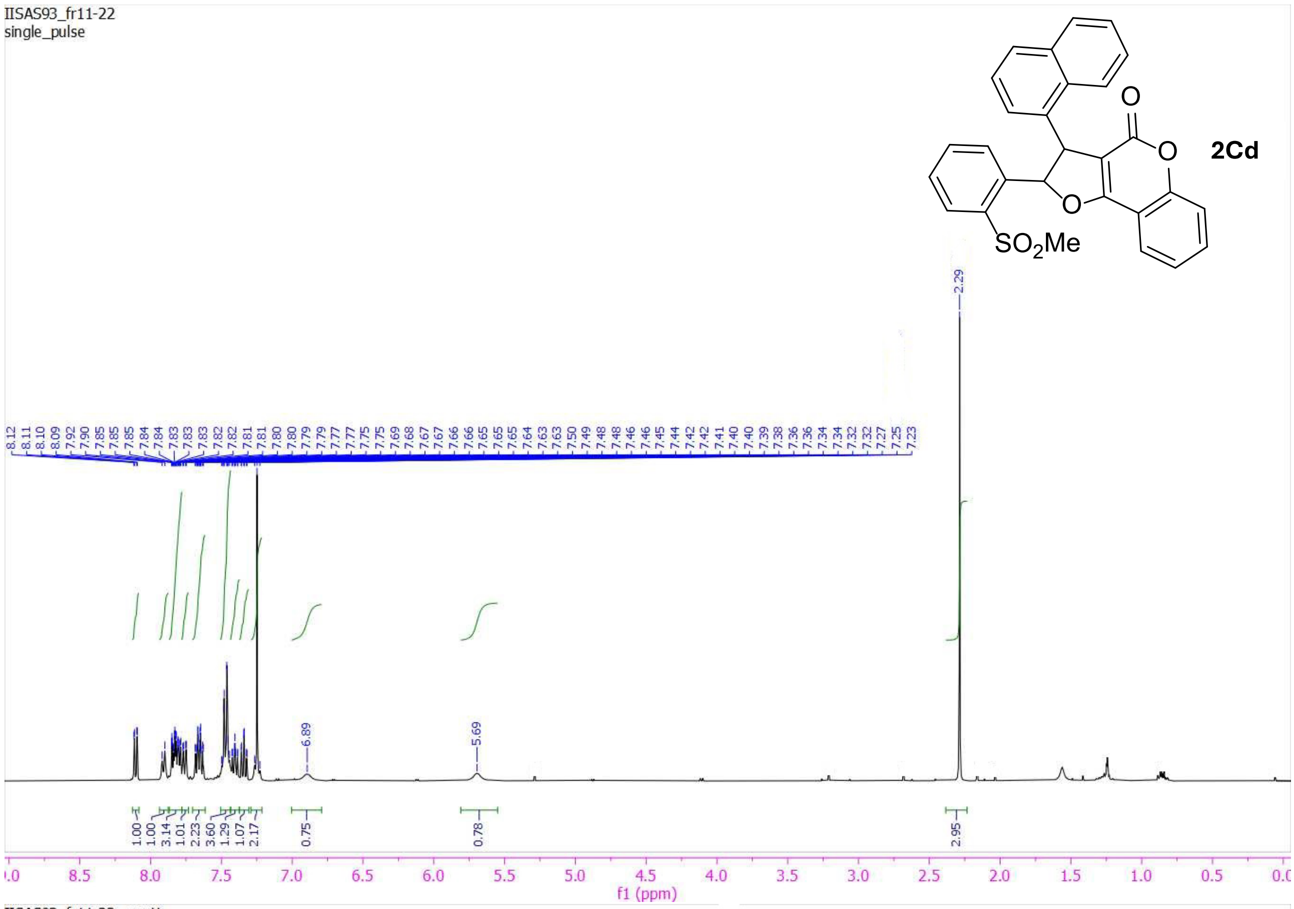

IISAS93_fr11-22
single_pulse
2Cd
O
O
O
SO2Me
8.12 8.11 8.10 8.09 7.92 7.90 7.85 7.85 7.85 7.84 7.84 7.83 7.83 7.83 7.82 7.82 7.81 7.81 7.80 7.80 7.79 7.79 7.77 7.75 7.75 7.69 7.68 7.67 7.66 7.65 7.65 7.65 7.64 7.63 7.63 7.50 7.49 7.48 7.48 7.46 7.46 7.45 7.44 7.42 7.41 7.41 7.40 7.40 7.39 7.38 7.36 7.36 7.34 7.32 7.32 7.27 7.25 7.23
6.89
5.69
2.29
1.00 1.00 3.14 1.01 2.23 3.60 1.29 1.07 2.17
0.75
0.78
2.95
f1 (ppm)

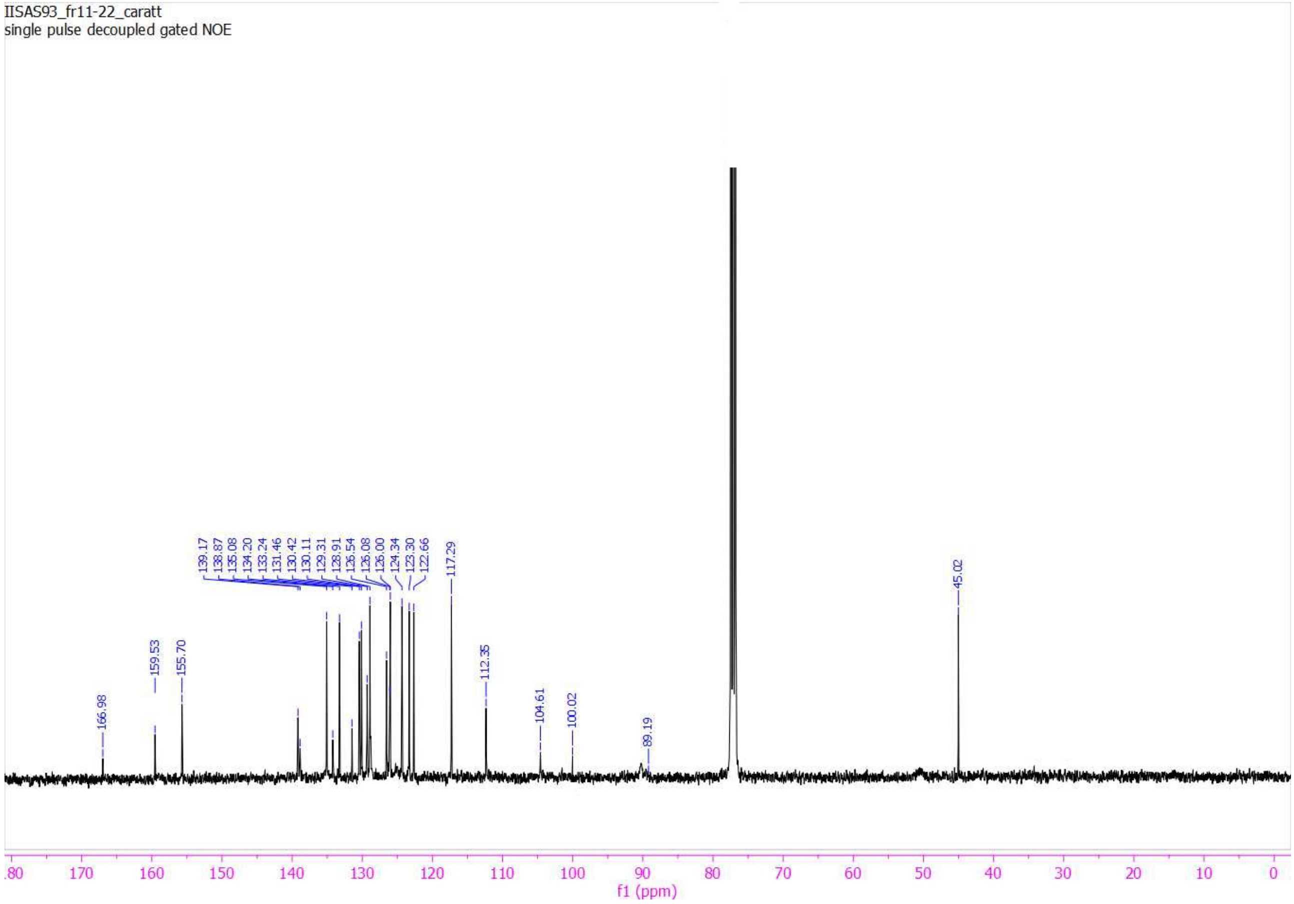

IISAS93_fr11-22_caratt
single pulse decoupled gated NOE
166.98
159.53
155.70
139.17 138.87 135.08 134.20 133.24 131.46 130.42 130.11 129.31 128.91 126.54 126.08 126.00 124.34 123.30 122.66
117.29
112.35
104.61
100.02
89.19
45.02
f1 (ppm)

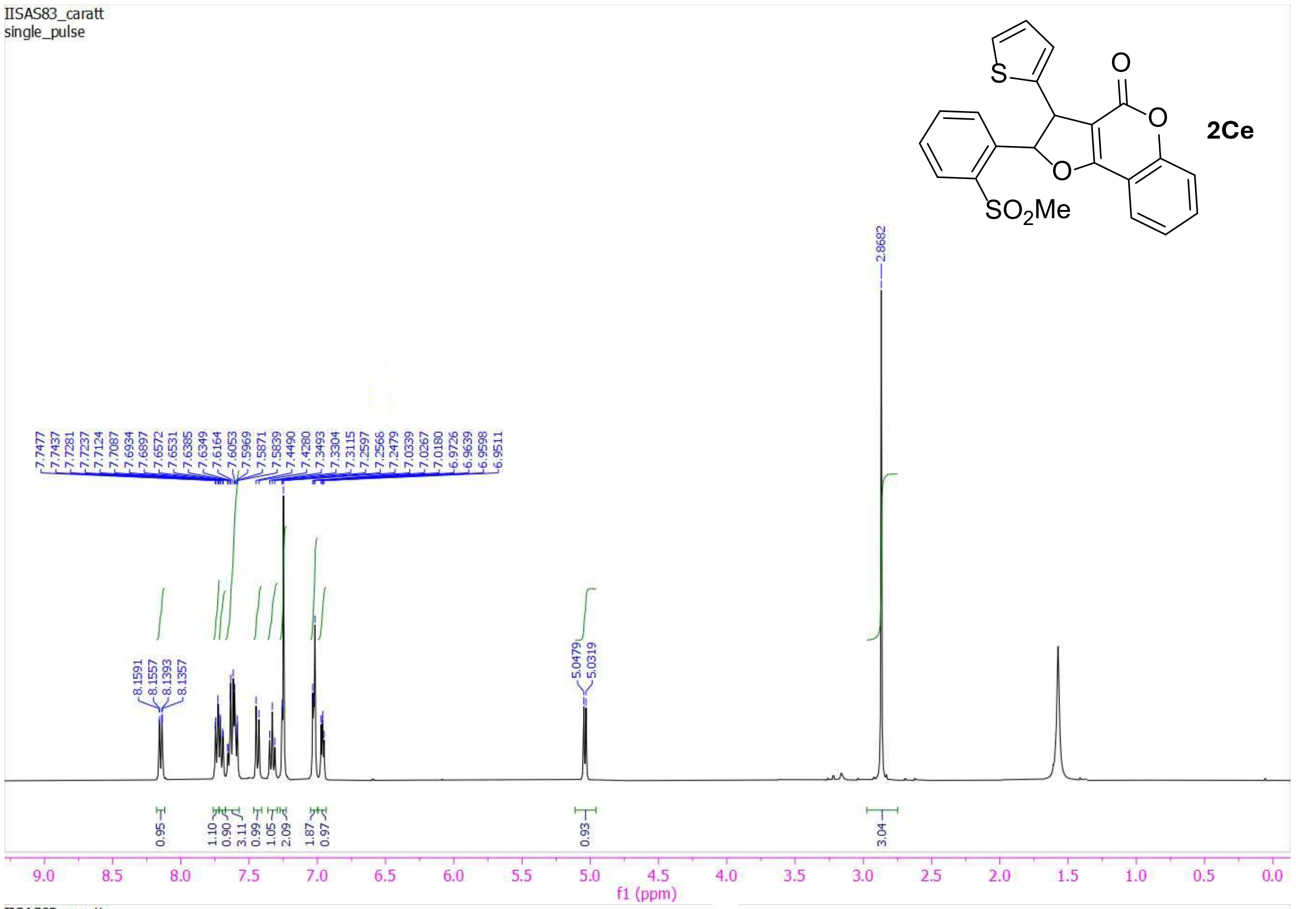
IISAS83_caratt
single_pulse
2Ce
SO2Me
7.7477 7.7437 7.7281 7.7237 7.7124 7.7087 7.6934 7.6897 7.6572 7.6531 7.6385 7.6349 7.6164 7.6053 7.5969 7.5871 7.5839 7.4490 7.4280 7.3493 7.3304 7.3115 7.2597 7.2566 7.2479 7.0339 7.0267 7.0180 6.9726 6.9639 6.9598 6.9511
8.1591 8.1557 8.1393 8.1357
5.0479 5.0319
2.8682
0.95 1.10 0.90 3.11 0.99 1.05 2.09 1.87 0.97 0.93 3.04
f1 (ppm)

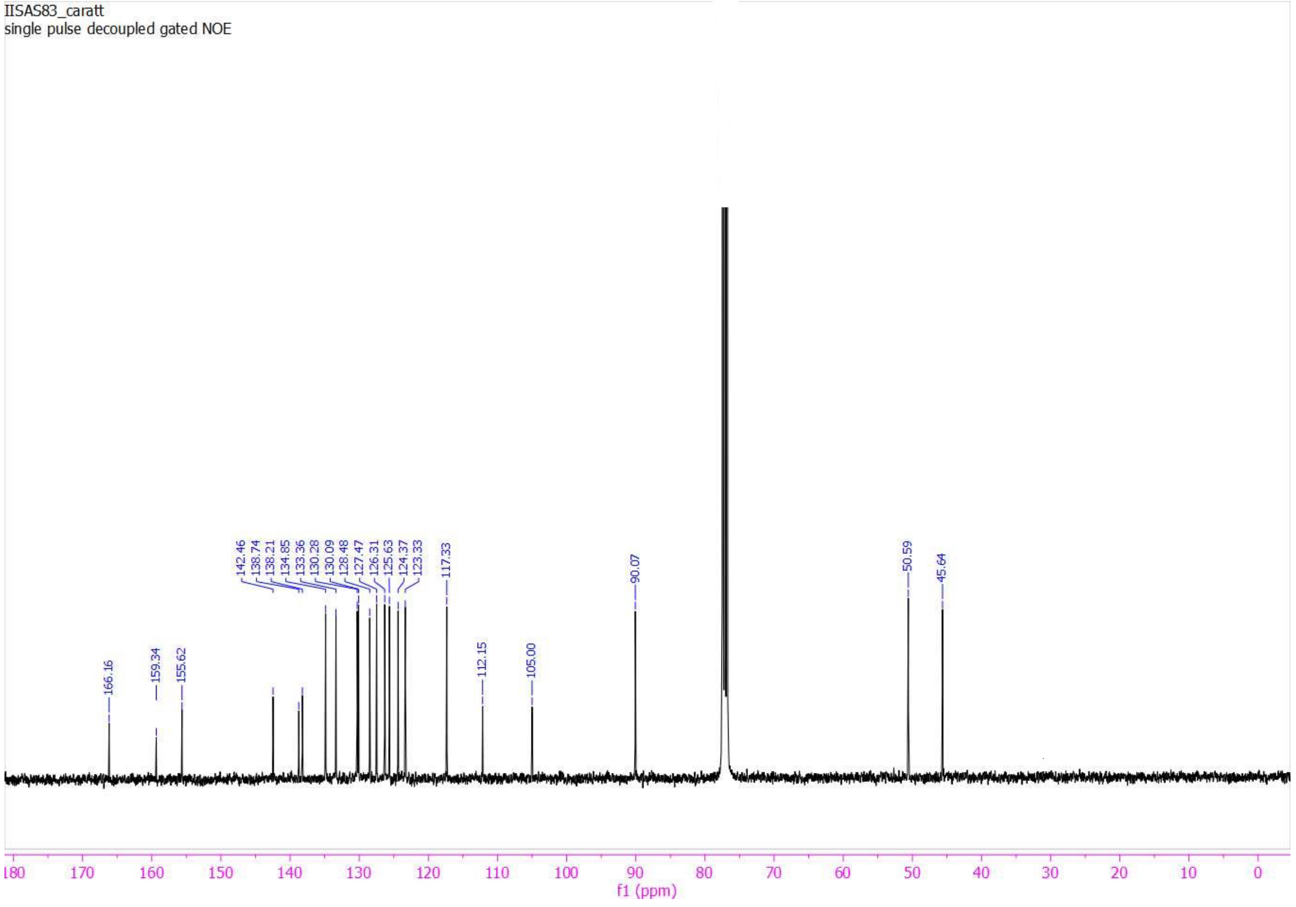
IISAS83_caratt
single pulse decoupled gated NOE
166.16 159.34 155.62 142.46 138.74 138.21 134.85 133.36 130.28 130.09 128.48 127.47 126.31 125.63 124.37 123.33 117.33 112.15 105.00 90.07 50.59 45.64
f1 (ppm)

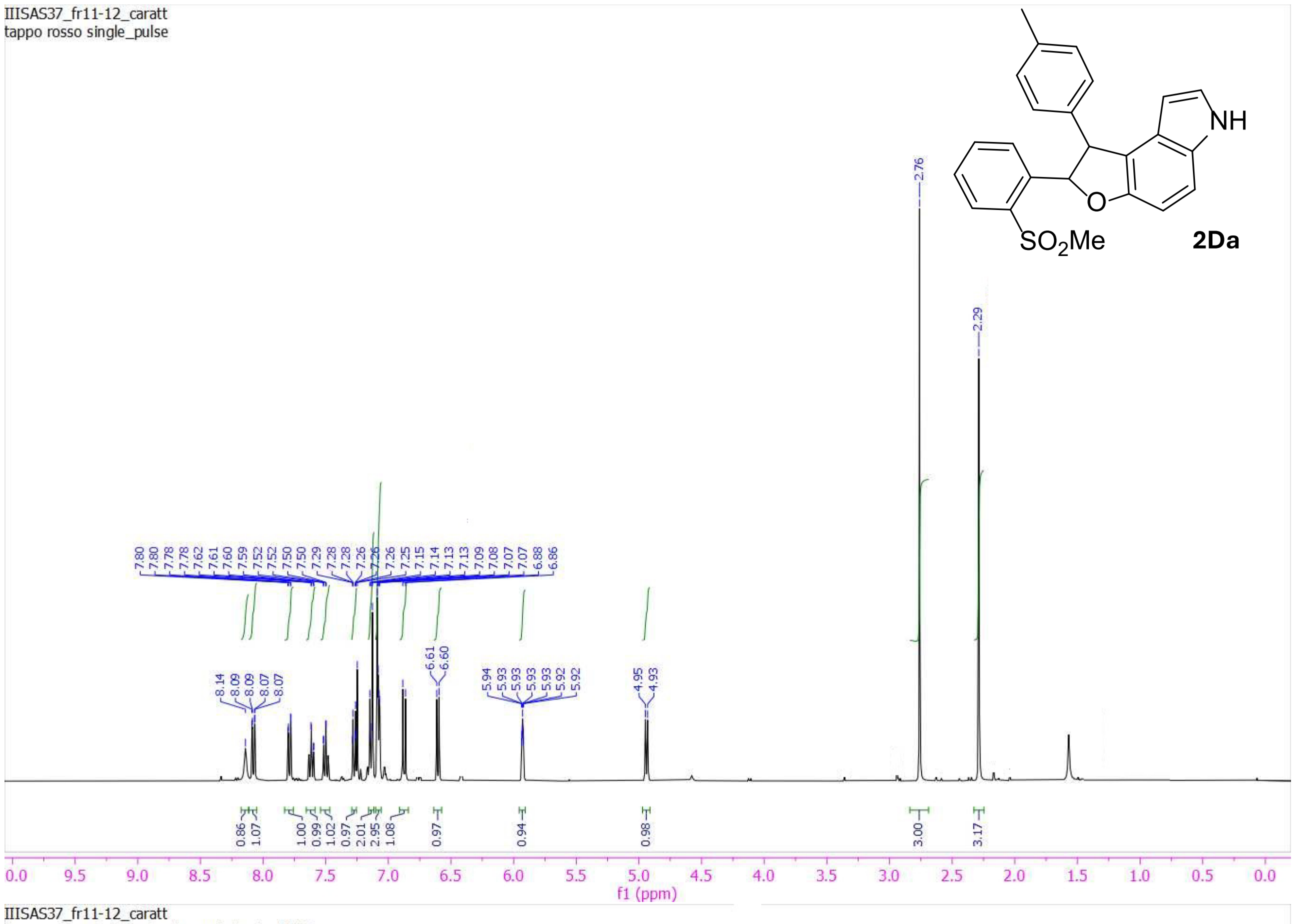
IIISAS37_fr11-12_caratt
tappo rosso single_pulse
SO2Me
NH
O
2Da
7.80 7.80 7.78 7.78 7.62 7.61 7.60 7.59 7.52 7.52 7.50 7.50 7.29 7.28 7.28 7.26 7.26 7.26 7.25 7.15 7.14 7.13 7.13 7.09 7.08 7.07 7.07 6.88 6.86
8.14 8.09 8.09 8.07 8.07
6.61 6.60
5.94 5.93 5.93 5.93 5.93 5.92 5.92
4.95 4.93
2.76
2.29
0.86 1.07 1.00 0.99 1.02 0.97 2.01 2.95 1.08 0.97 0.94 0.98 3.00 3.17
f1 (ppm)

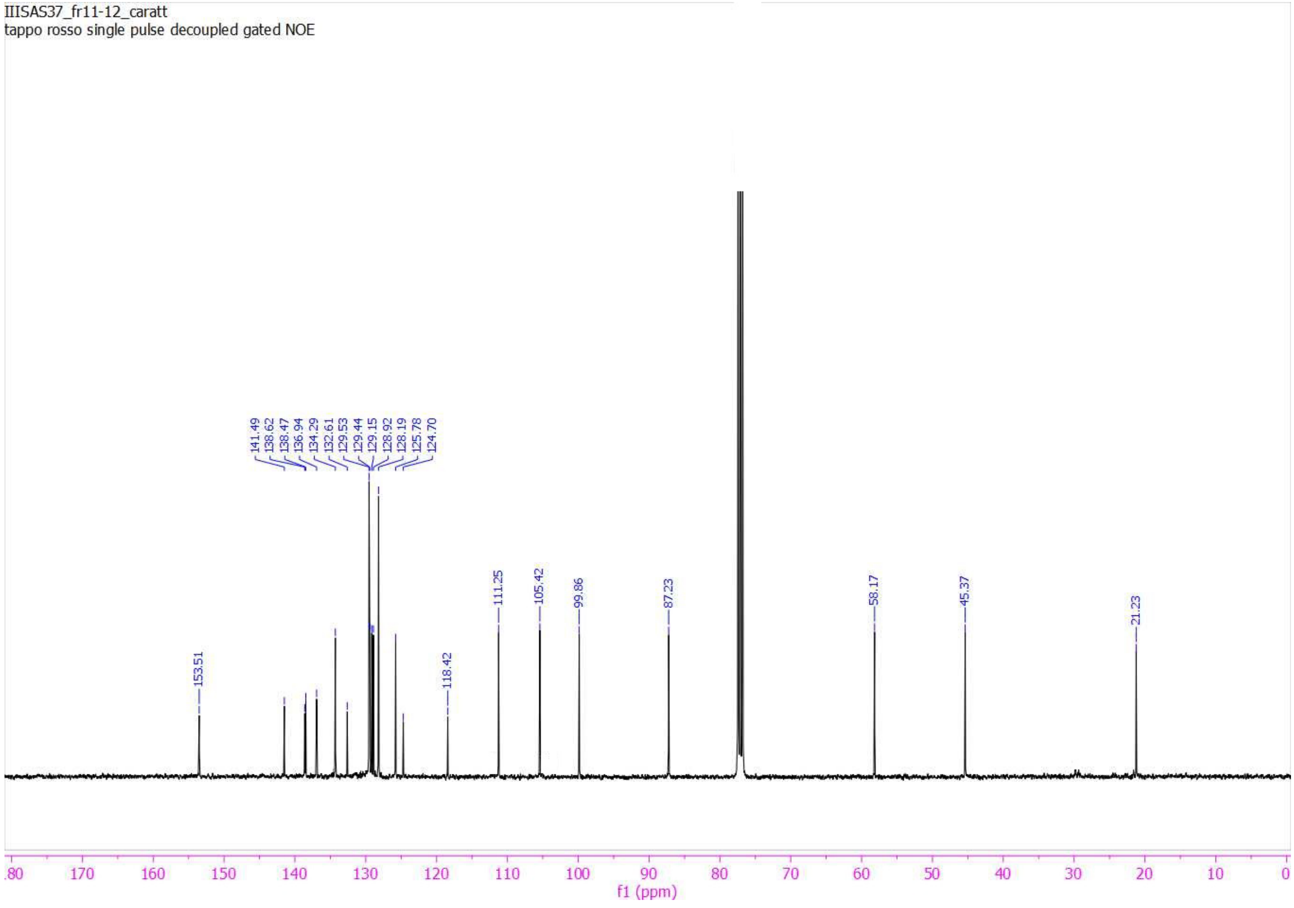
IIISAS37_fr11-12_caratt
tappo rosso single pulse decoupled gated NOE
141.49 138.62 138.47 136.94 134.29 132.61 129.53 129.44 129.15 128.92 128.19 125.78 124.70
153.51
118.42
111.25
105.42
99.86
87.23
58.17
45.37
21.23
f1 (ppm)

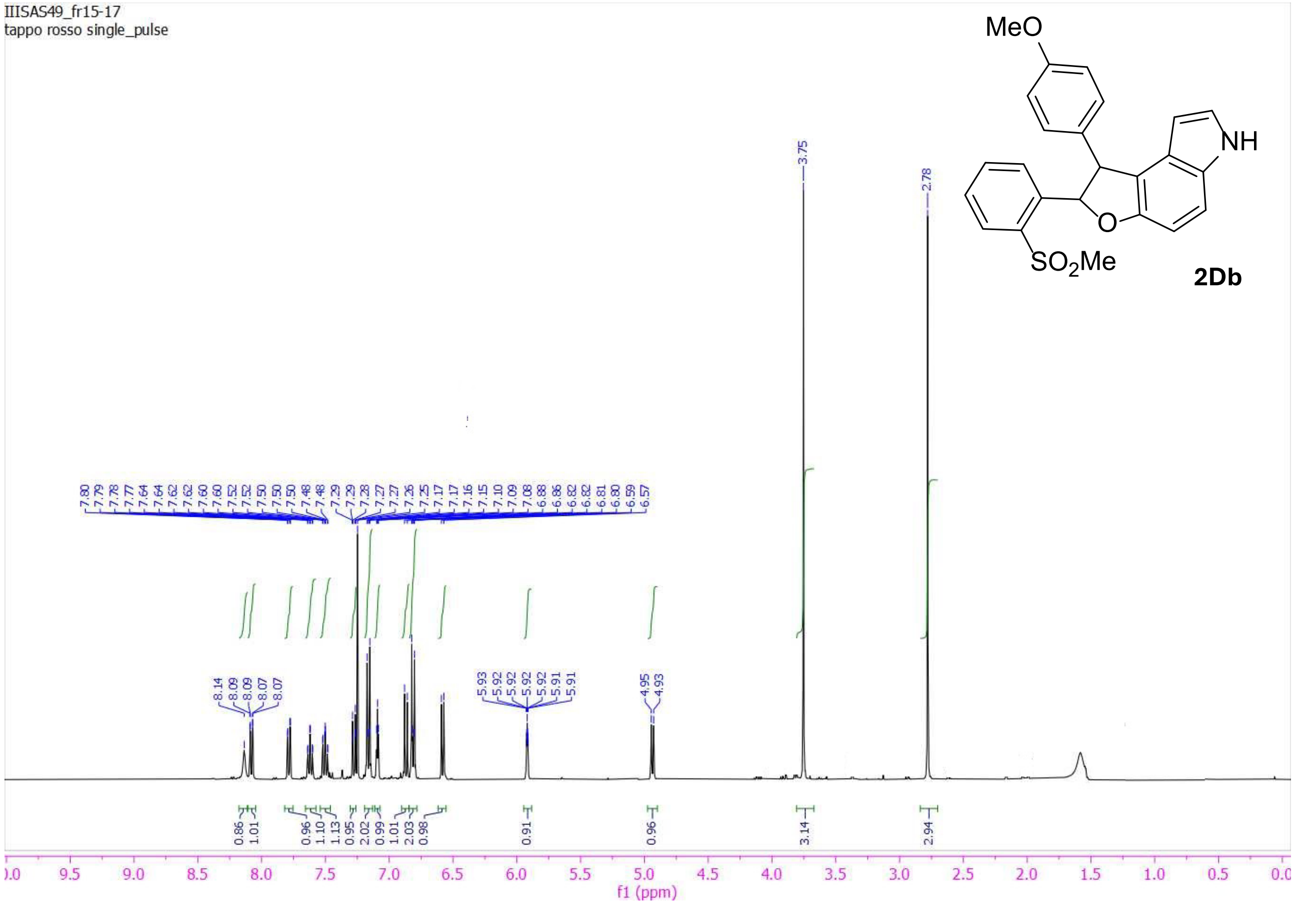
IIISAS49_fr15-17
tappo rosso single_pulse
MeO
NH
O
SO2Me
2Db
3.75
2.78
7.80 7.79 7.78 7.77 7.64 7.62 7.62 7.60 7.60 7.52 7.52 7.50 7.50 7.50 7.48 7.48 7.29 7.29 7.28 7.27 7.27 7.26 7.25 7.17 7.17 7.16 7.15 7.10 7.09 7.08 6.88 6.86 6.82 6.81 6.80 6.59 6.57
8.14 8.09 8.09 8.07 8.07
5.93 5.92 5.92 5.92 5.92 5.91 5.91
4.95 4.93
0.86 1.01 0.96 1.10 1.13 0.95 2.02 0.99 1.01 2.03 0.98 0.91 0.96 3.14 2.94
f1 (ppm)

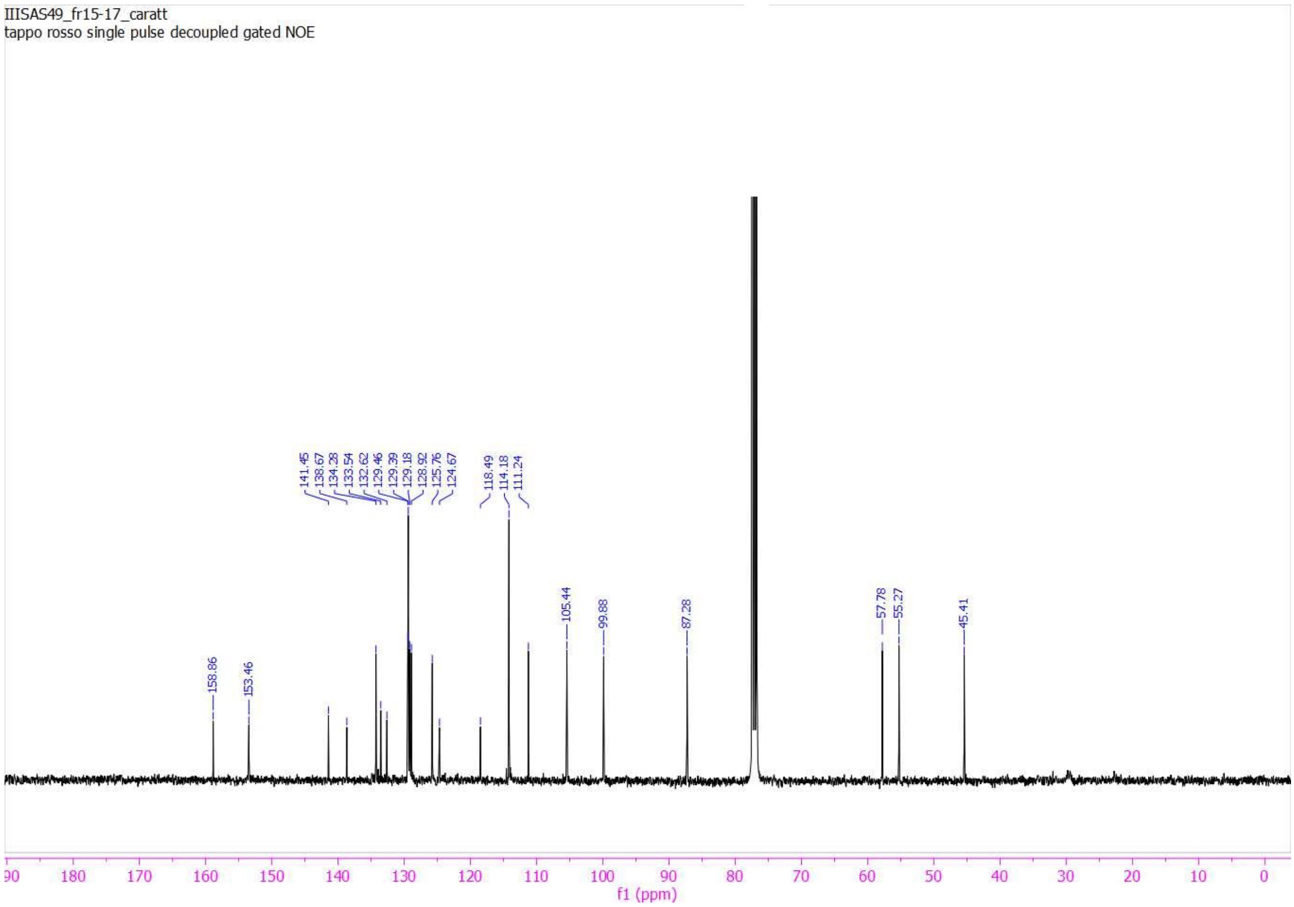
IIISAS49_fr15-17_caratt
tappo rosso single pulse decoupled gated NOE
141.45 138.67 134.28 133.54 132.62 129.46 129.39 129.18 128.92 125.76 124.67
118.49 114.18 111.24
158.86 153.46 105.44 99.88 87.28 57.78 55.27 45.41
f1 (ppm)

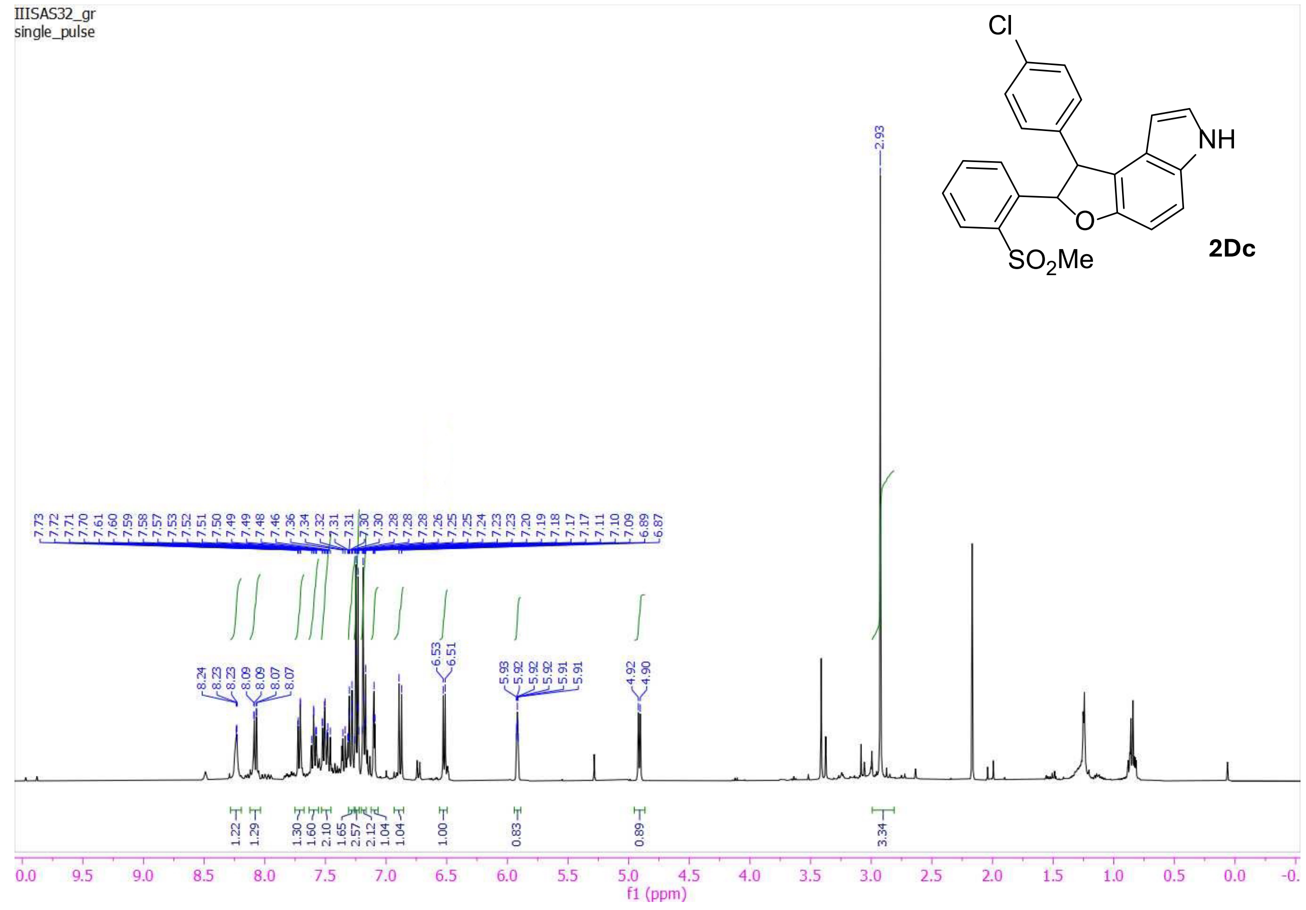
IIISAS32_gr
single_pulse
Cl
NH
O
SO2Me
2Dc
f1 (ppm)

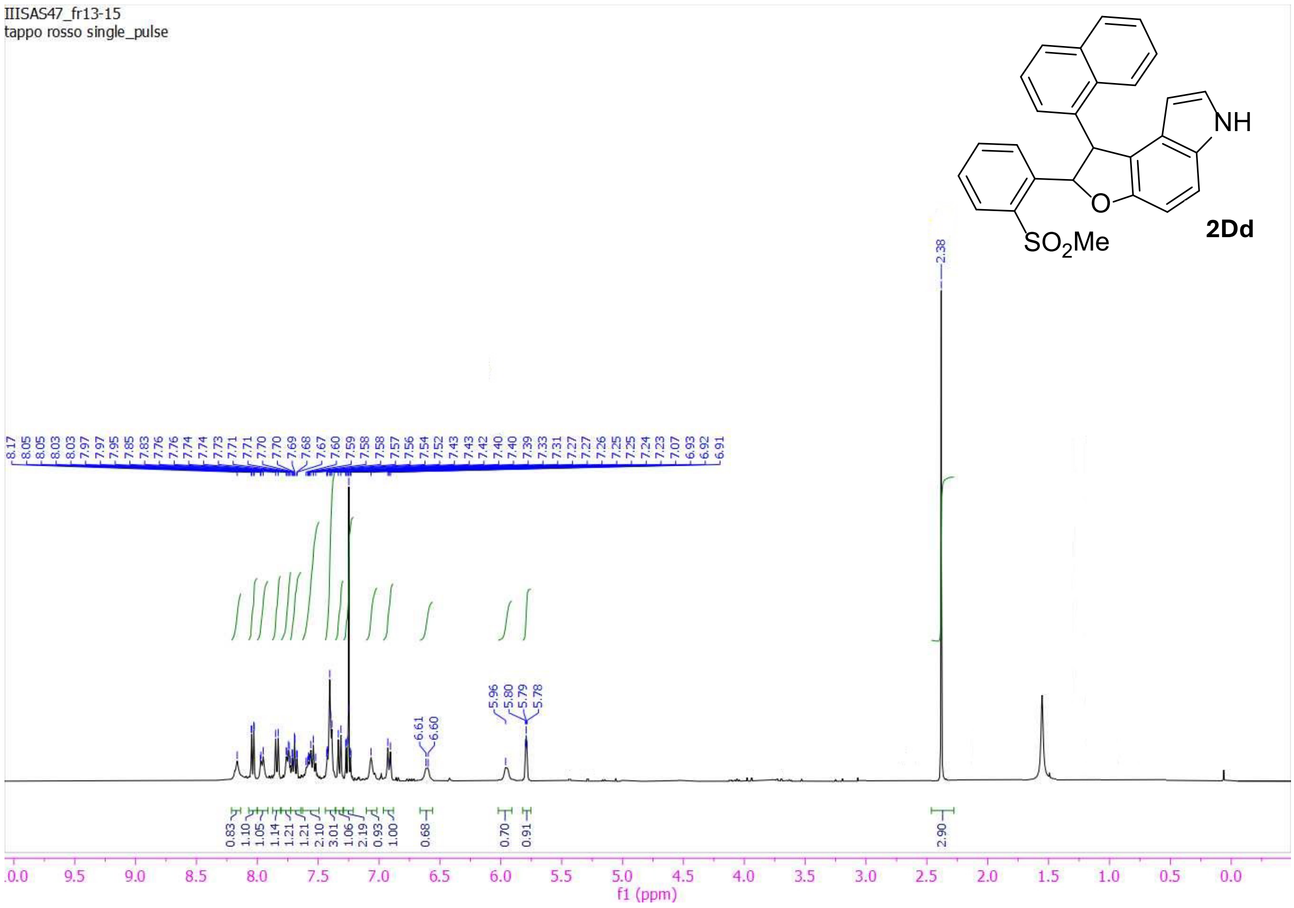
IIISAS47_fr13-15
tappo rosso single_pulse
NH
O
SO2Me
2Dd
8.17 8.05 8.03 8.03 7.97 7.95 7.85 7.83 7.76 7.76 7.74 7.74 7.73 7.71 7.71 7.70 7.69 7.68 7.67 7.60 7.59 7.58 7.58 7.57 7.56 7.54 7.52 7.43 7.43 7.42 7.40 7.40 7.39 7.33 7.31 7.27 7.27 7.26 7.25 7.25 7.24 7.23 7.07 6.93 6.92 6.91
6.61 6.60
5.96 5.80 5.79 5.78
2.38
0.83 1.10 1.05 1.14 1.21 1.21 2.10 3.01 1.06 2.19 0.93 1.00 0.68 0.70 0.91 2.90
f1 (ppm)

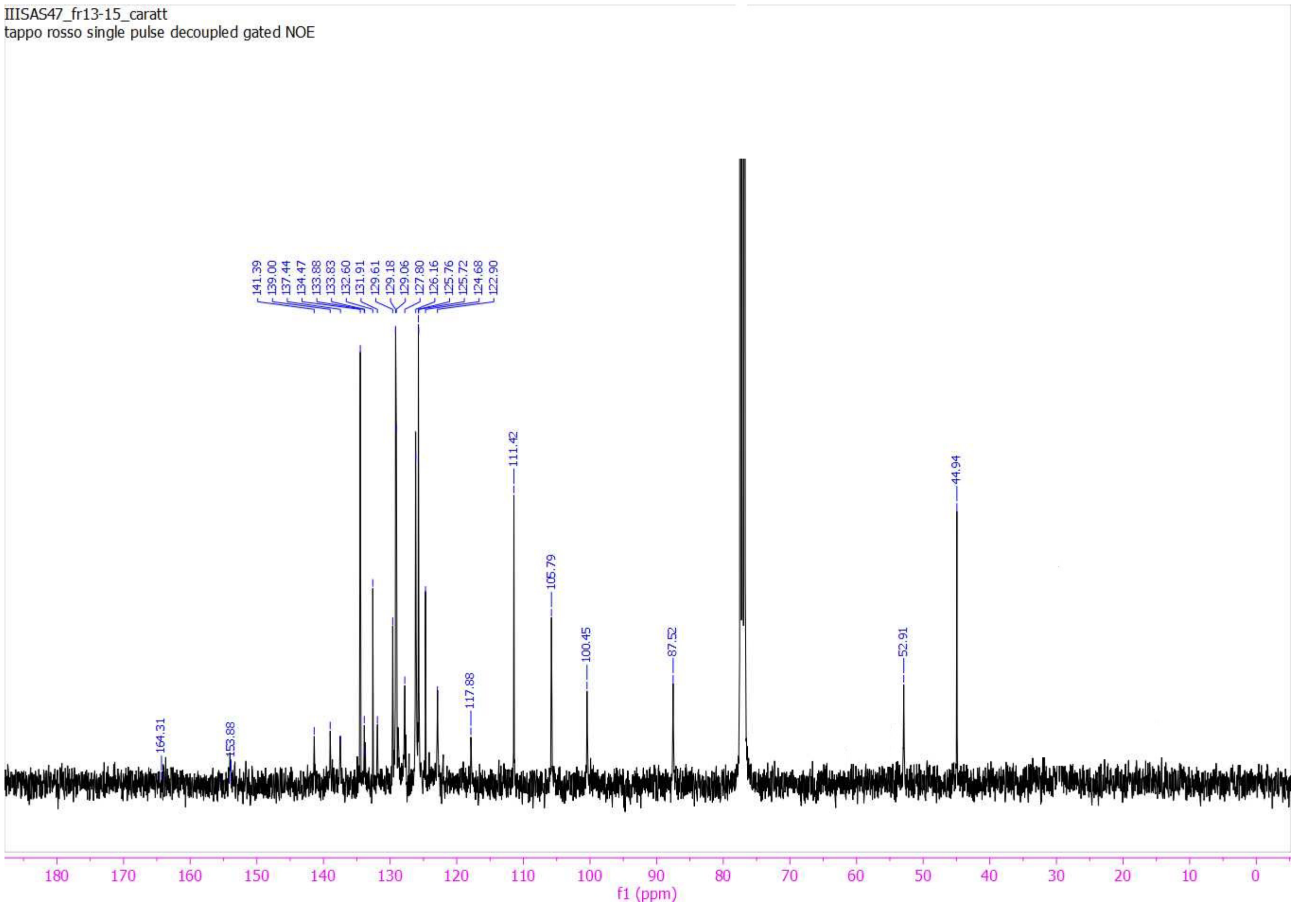
IIISAS47_fr13-15_caratt
tappo rosso single pulse decoupled gated NOE
141.39 139.00 137.44 134.47 133.88 133.83 132.60 131.91 129.61 129.18 129.06 127.80 126.16 125.76 125.72 124.68 122.90
164.31 153.88 117.88 111.42 105.79 100.45 87.52 52.91 44.94
f1 (ppm)

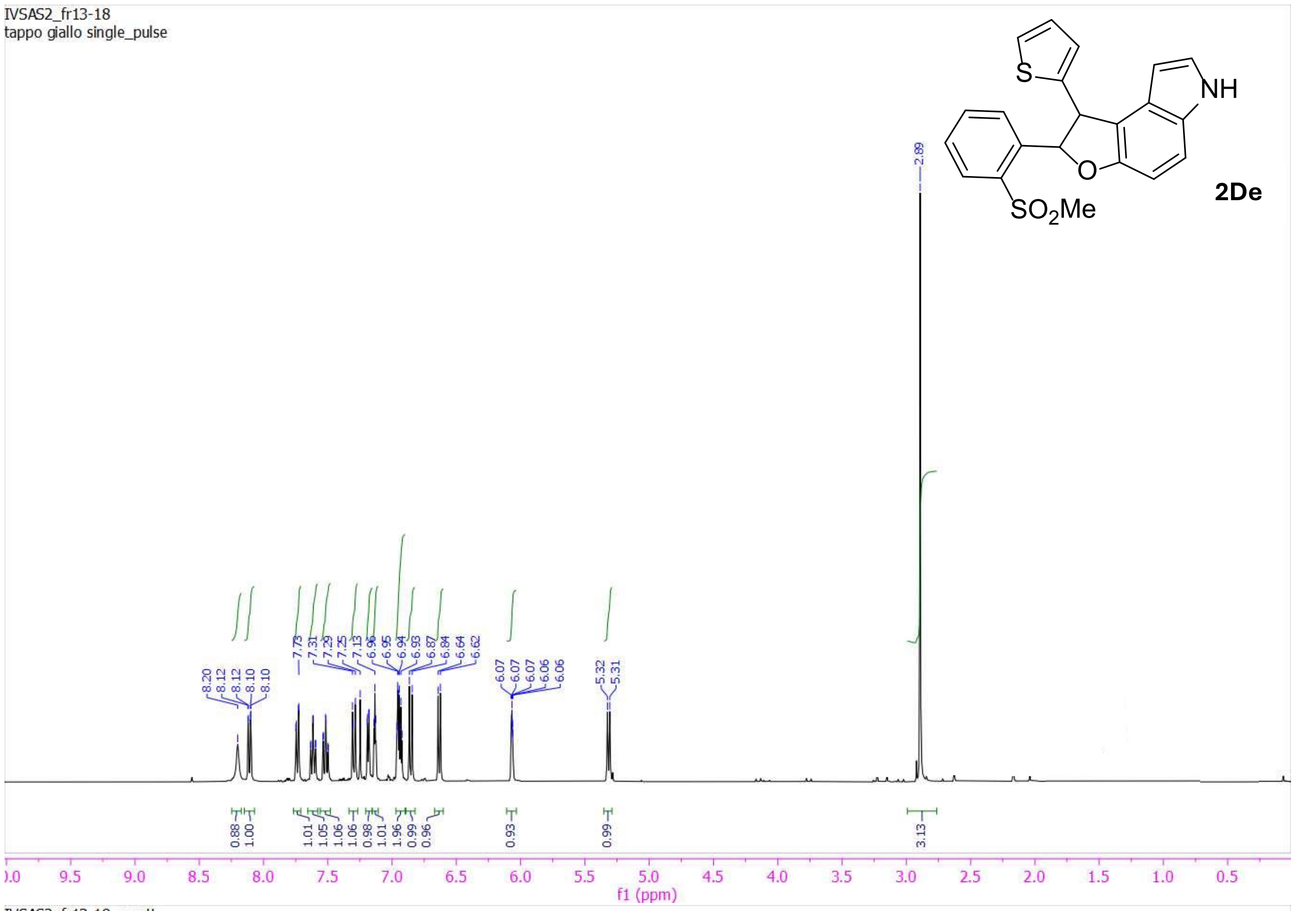
IVSAS2_fr13-18
tappo giallo single_pulse
S
NH
O
SO2Me
2De
8.20 8.12 8.12 8.10 8.10
7.73 7.31 7.29 7.25 7.13 6.96 6.95 6.94 6.93 6.87 6.84 6.64 6.62
6.07 6.07 6.07 6.06 6.06
5.32 5.31
2.89
0.88 1.00 1.01 1.05 1.06 1.06 0.98 1.01 1.96 0.99 0.96 0.93 0.99 3.13
f1 (ppm)

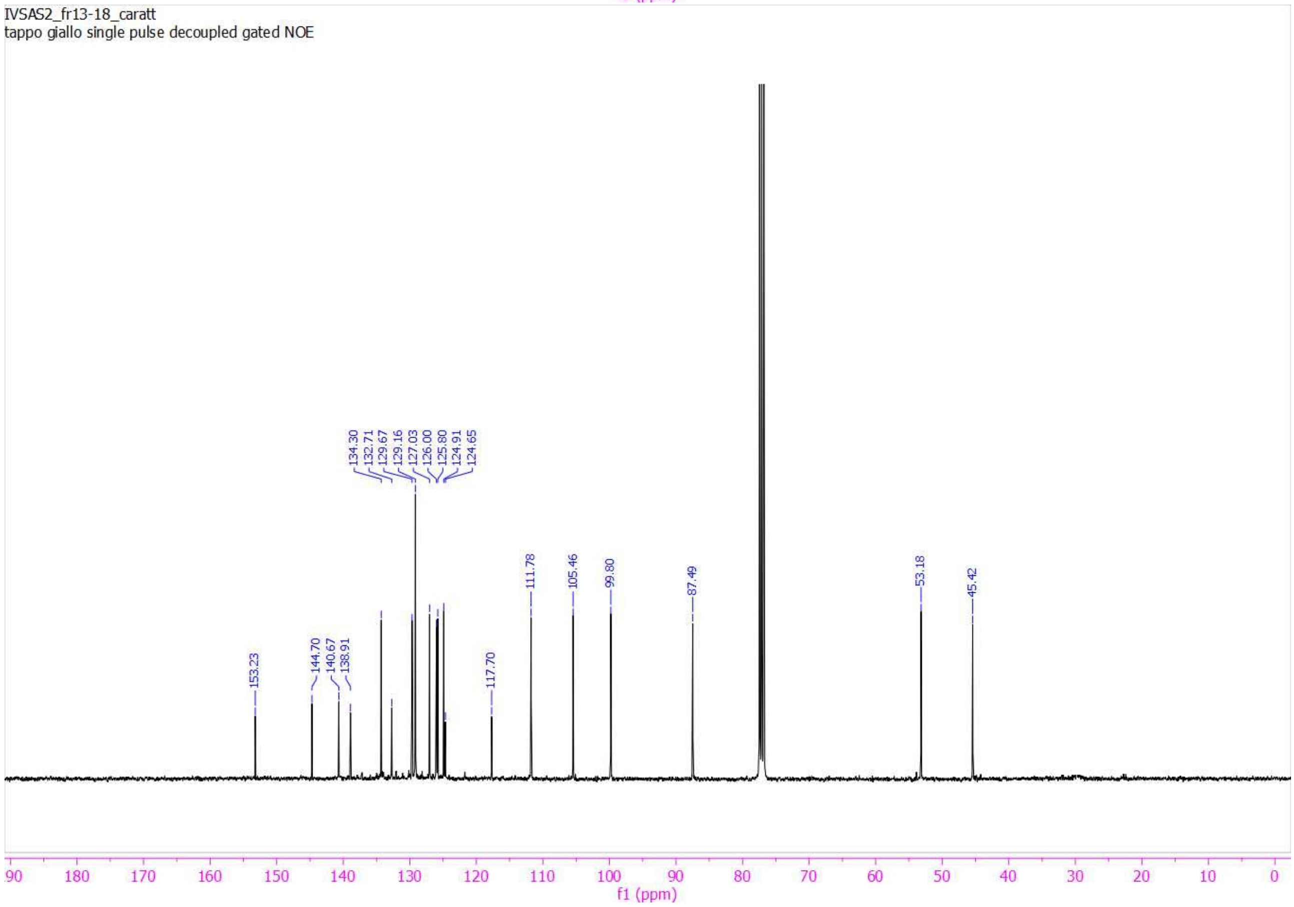
IVSAS2_fr13-18_caratt
tappo giallo single pulse decoupled gated NOE
153.23
144.70 140.67 138.91
134.30 132.71 129.67 129.16 127.03 126.00 125.80 124.91 124.65
117.70
111.78
105.46
99.80
87.49
53.18
45.42
f1 (ppm)

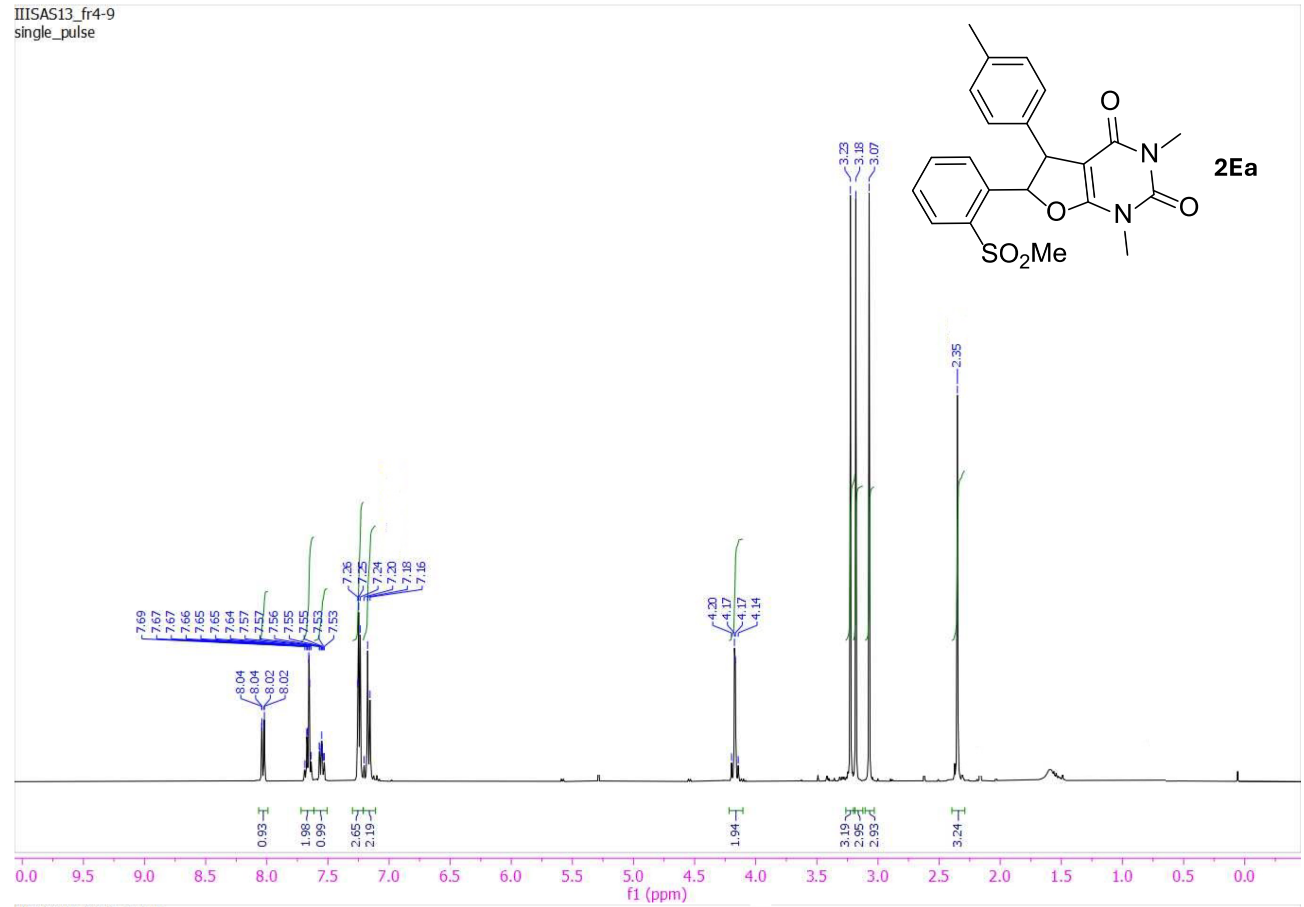
IIISAS13_fr4-9
single_pulse
SO2Me
2Ea
8.04 8.04 8.02 8.02
7.69 7.67 7.67 7.66 7.65 7.65 7.64 7.57 7.57 7.56 7.55 7.55 7.55 7.53 7.53
7.26 7.25 7.24 7.20 7.18 7.16
4.20 4.17 4.17 4.14
3.23 3.18 3.07
2.35
0.93 1.98 0.99 2.65 2.19 1.94 3.19 2.95 2.93 3.24
f1 (ppm)

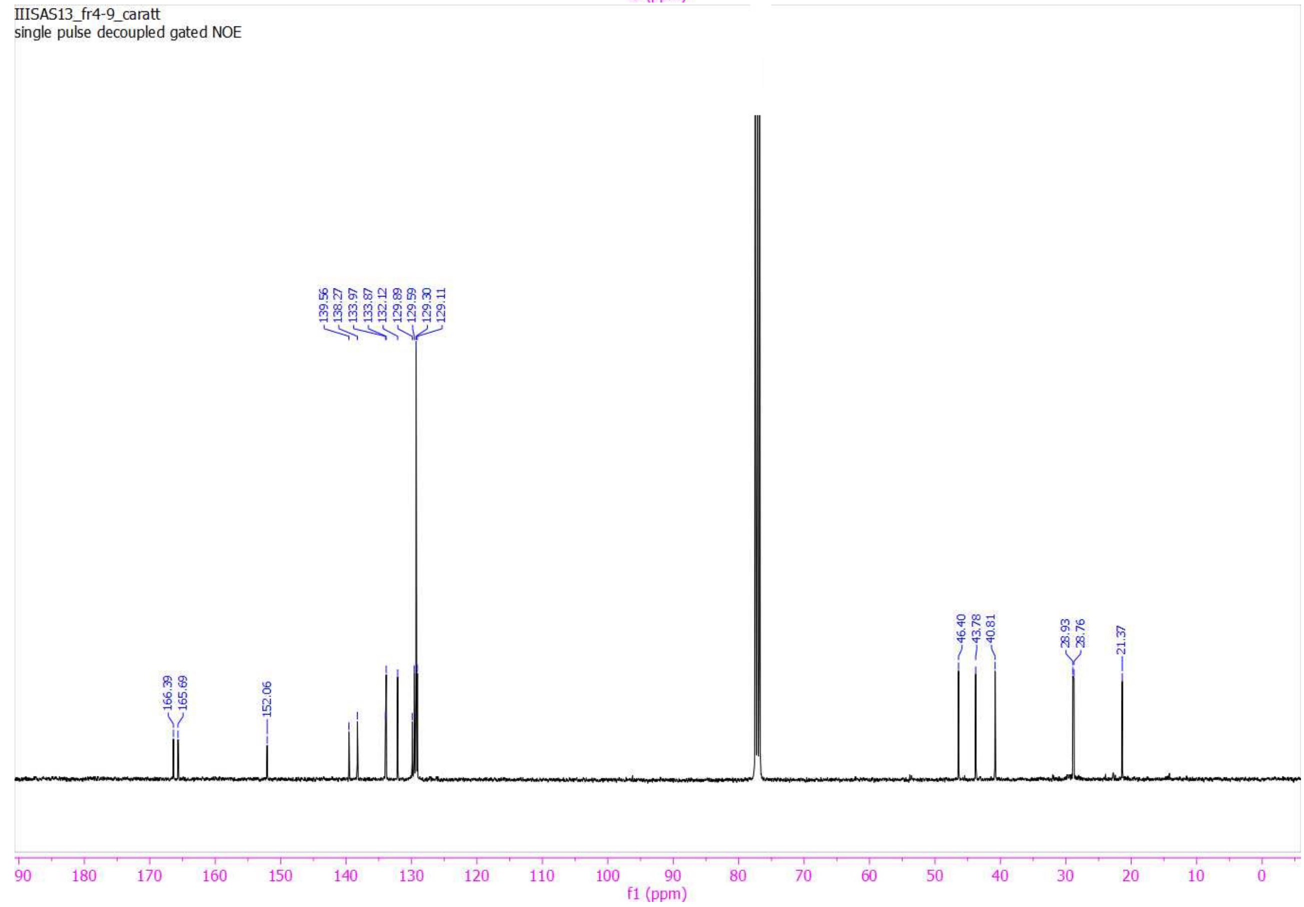
IIISAS13_fr4-9_caratt
single pulse decoupled gated NOE
166.39 165.69
152.06
139.56 138.27 133.97 133.87 132.12 129.89 129.59 129.30 129.11
46.40 43.78 40.81
28.93 28.76
21.37
f1 (ppm)

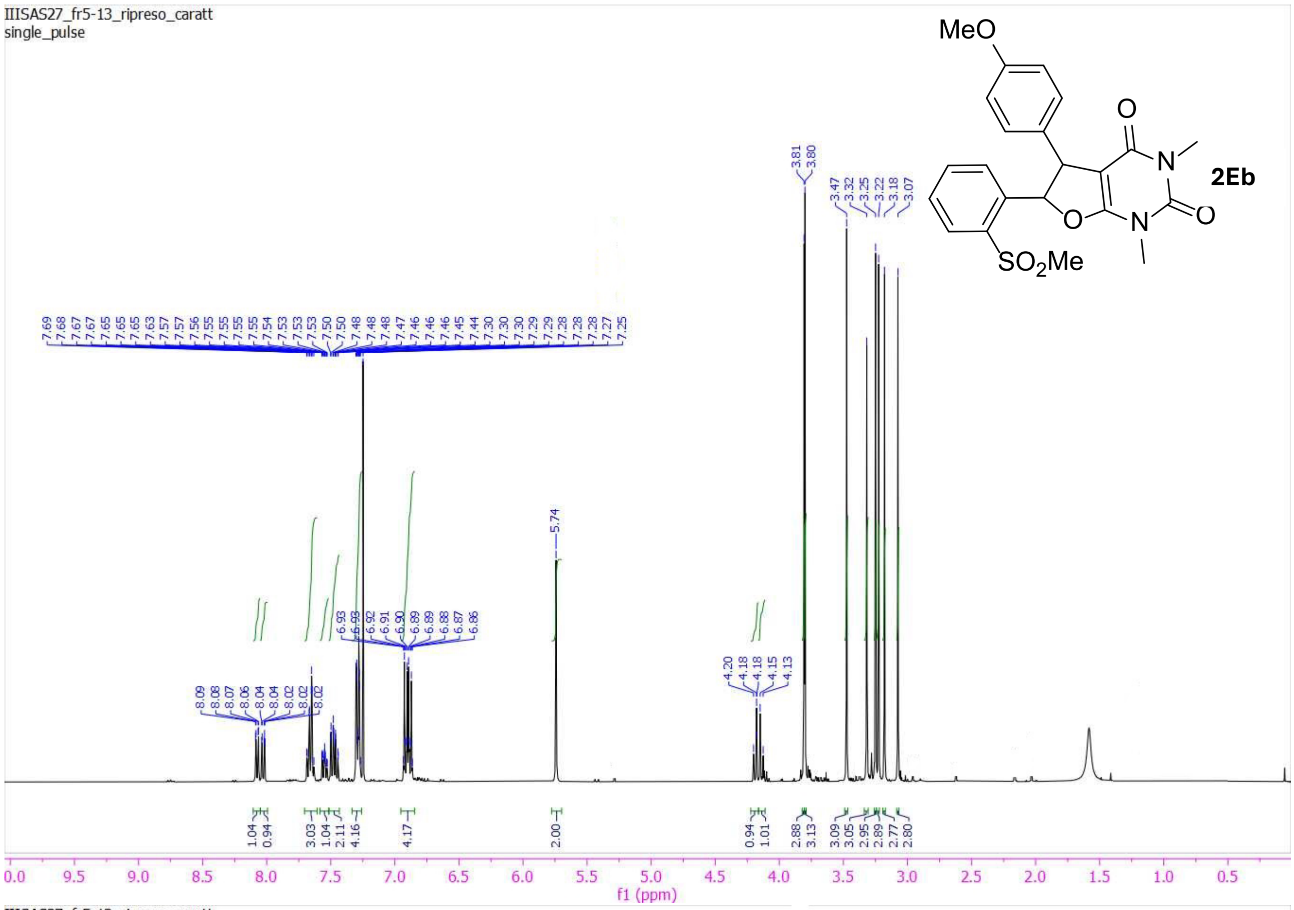
IIISAS27_fr5-13_ripreso_caratt
single_pulse
MeO
SO2Me
2Eb
7.69 7.68 7.67 7.67 7.65 7.65 7.65 7.63 7.57 7.57 7.56 7.55 7.55 7.55 7.55 7.54 7.53 7.53 7.53 7.53 7.50 7.50 7.48 7.48 7.48 7.47 7.46 7.46 7.45 7.44 7.30 7.30 7.30 7.29 7.29 7.28 7.28 7.28 7.27 7.25
3.81 3.80
3.47 3.32 3.25 3.22 3.18 3.07
5.74
6.93 6.93 6.92 6.91 6.90 6.89 6.89 6.88 6.87 6.86
8.09 8.08 8.07 8.06 8.04 8.04 8.02 8.02
4.20 4.18 4.18 4.15 4.13
1.04 0.94 3.03 1.04 2.11 4.16 4.17 2.00 0.94 1.01 2.88 3.13 3.09 3.05 2.95 2.89 2.77 2.80
f1 (ppm)

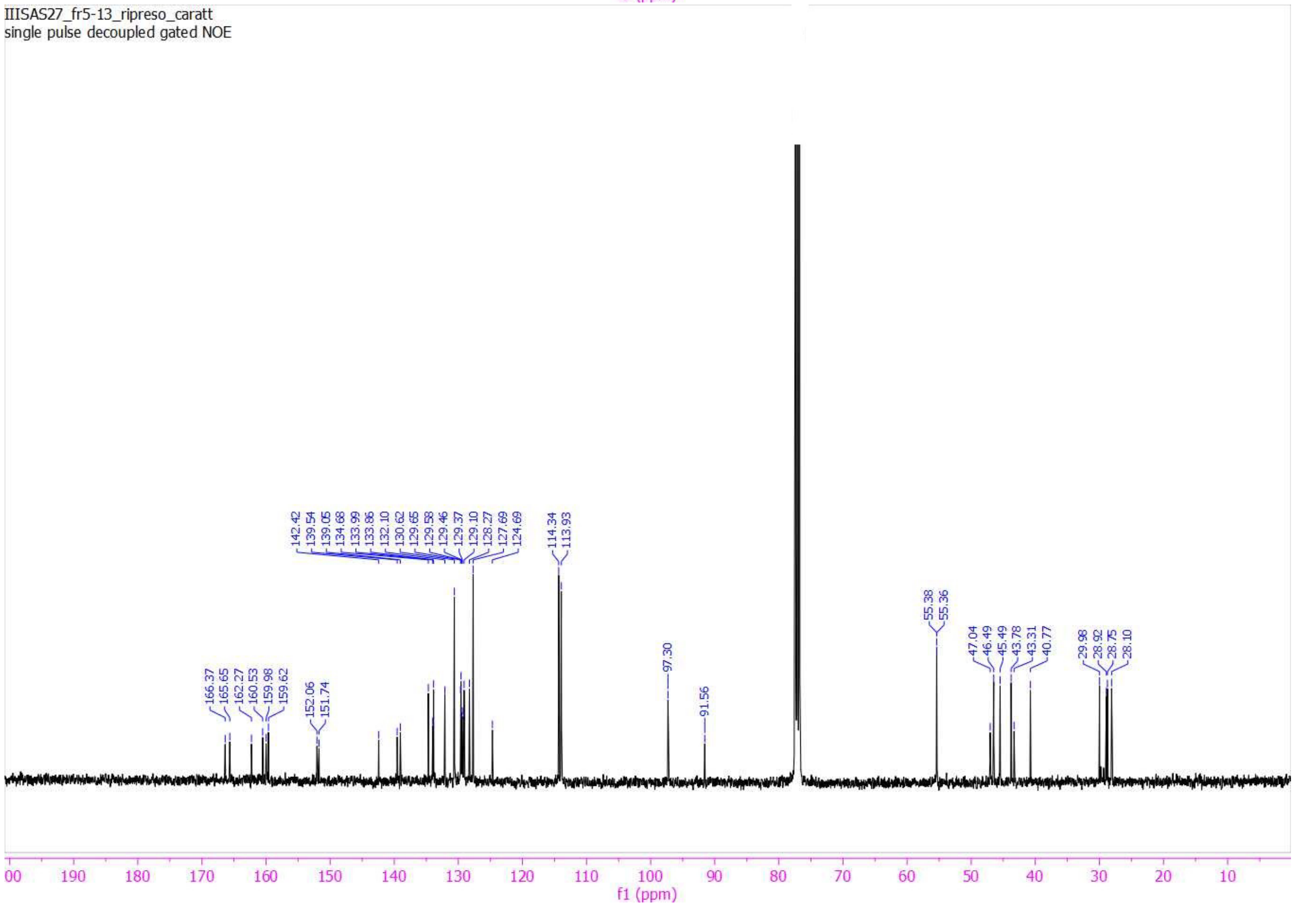
IIISAS27_fr5-13_ripreso_caratt
single pulse decoupled gated NOE
166.37 165.65 162.27 160.53 159.98 159.62
152.06 151.74
142.42 139.54 139.05 134.68 133.99 133.86 132.10 130.62 129.65 129.58 129.46 129.37 129.10 128.27 127.69 124.69
114.34 113.93
97.30
91.56
55.38 55.36
47.04 46.49 45.49 43.78 43.31 40.77
29.98 28.92 28.75 28.10
f1 (ppm)

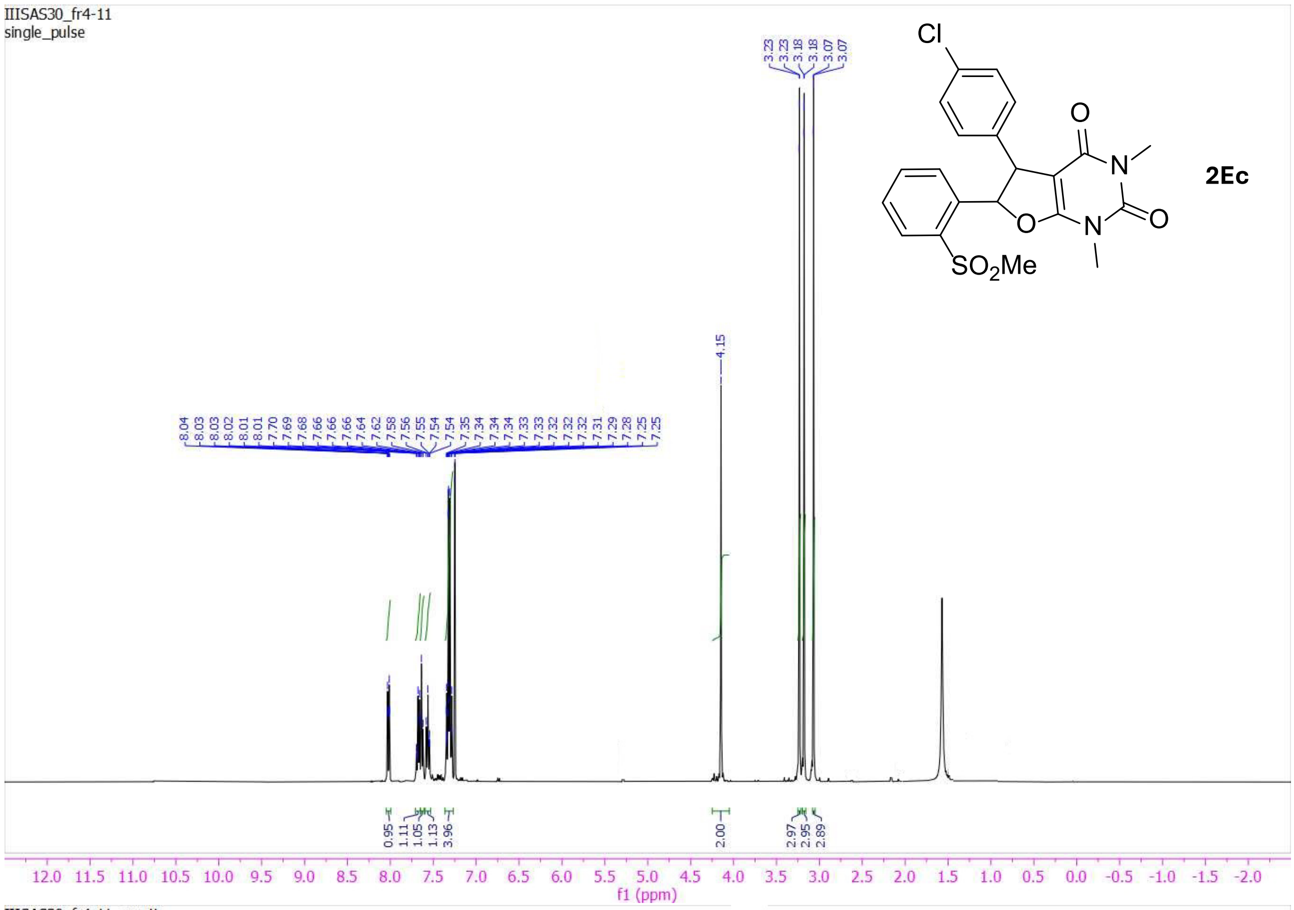
IIISAS30_fr4-11
single_pulse
8.04 8.03 8.02 8.01 8.01 7.70 7.69 7.68 7.66 7.66 7.66 7.64 7.62 7.58 7.56 7.55 7.54 7.54 7.35 7.34 7.34 7.34 7.33 7.33 7.32 7.32 7.32 7.31 7.29 7.28 7.25 7.25
4.15
3.23 3.23 3.18 3.18 3.07 3.07
0.95 1.11 1.05 1.13 3.96 2.00 2.97 2.95 2.89
Cl
O
N
O
N
O
SO_2Me
2Ec
f1 (ppm)

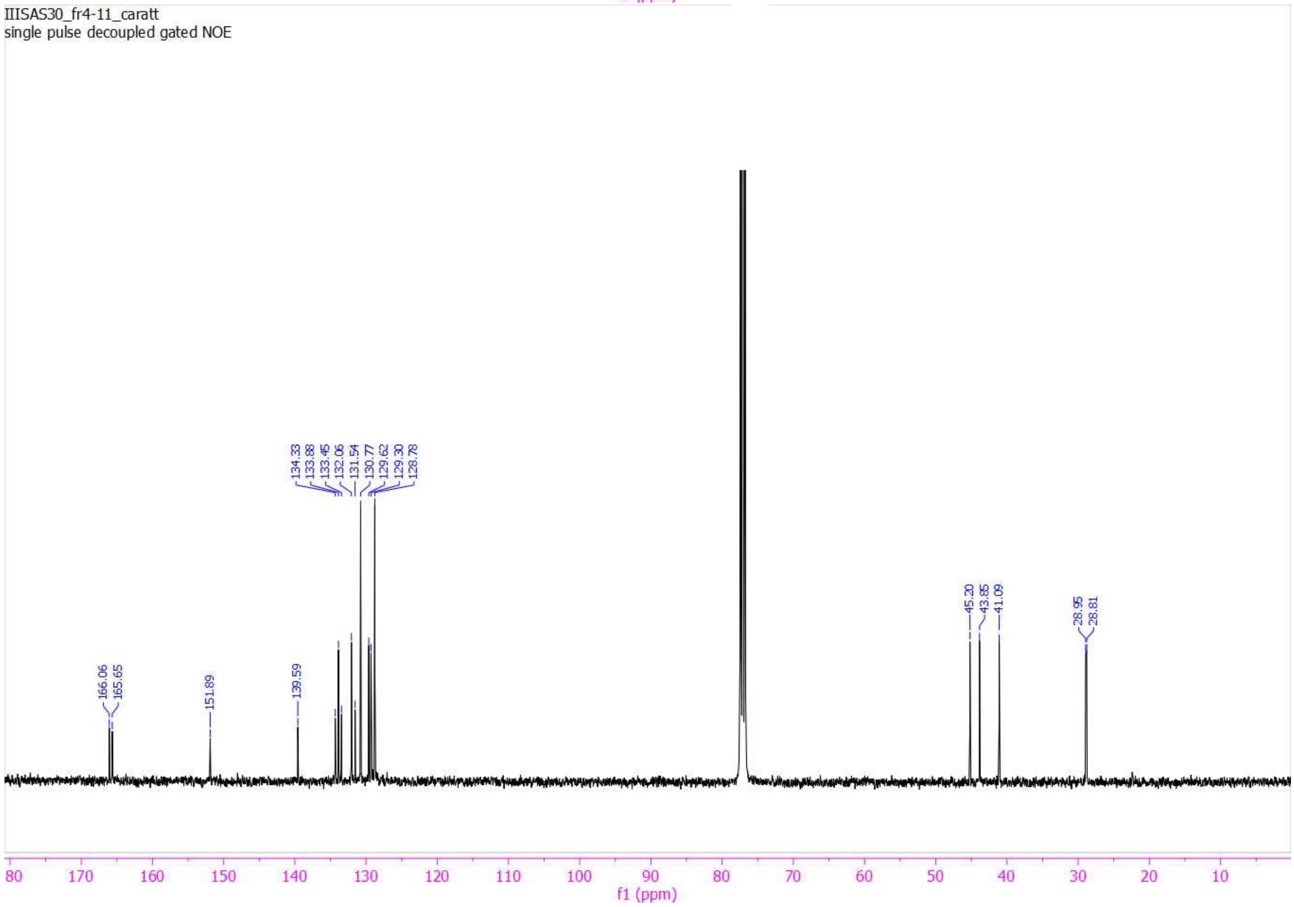
IIISAS30_fr4-11_caratt
single pulse decoupled gated NOE
166.06 165.65
151.89
139.59
134.33 133.88 133.45 132.06 131.54 130.77 129.62 129.30 128.78
45.20 43.85 41.09
28.95 28.81
f1 (ppm)

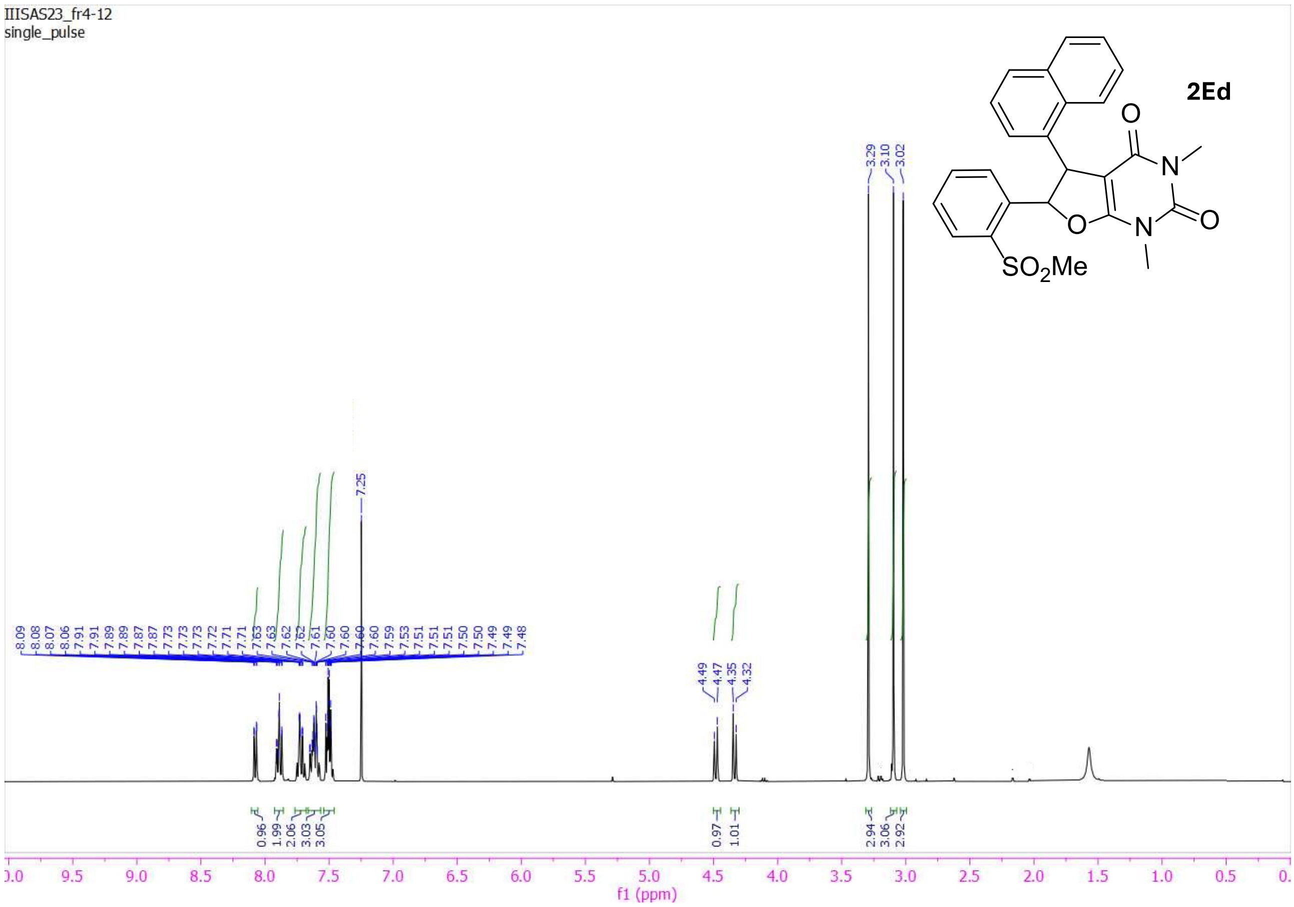
IIISAS23_fr4-12
single_pulse
2Ed
SO2Me
O
N
8.09 8.08 8.07 8.06 7.91 7.91 7.89 7.89 7.87 7.87 7.73 7.73 7.73 7.72 7.71 7.71 7.63 7.63 7.62 7.62 7.61 7.60 7.60 7.60 7.60 7.59 7.53 7.51 7.51 7.50 7.50 7.49 7.48
7.25
4.49 4.47 4.35 4.32
3.29 3.10 3.02
0.96 1.99 2.06 3.03 3.05
0.97 1.01
2.94 3.06 2.92
f1 (ppm)

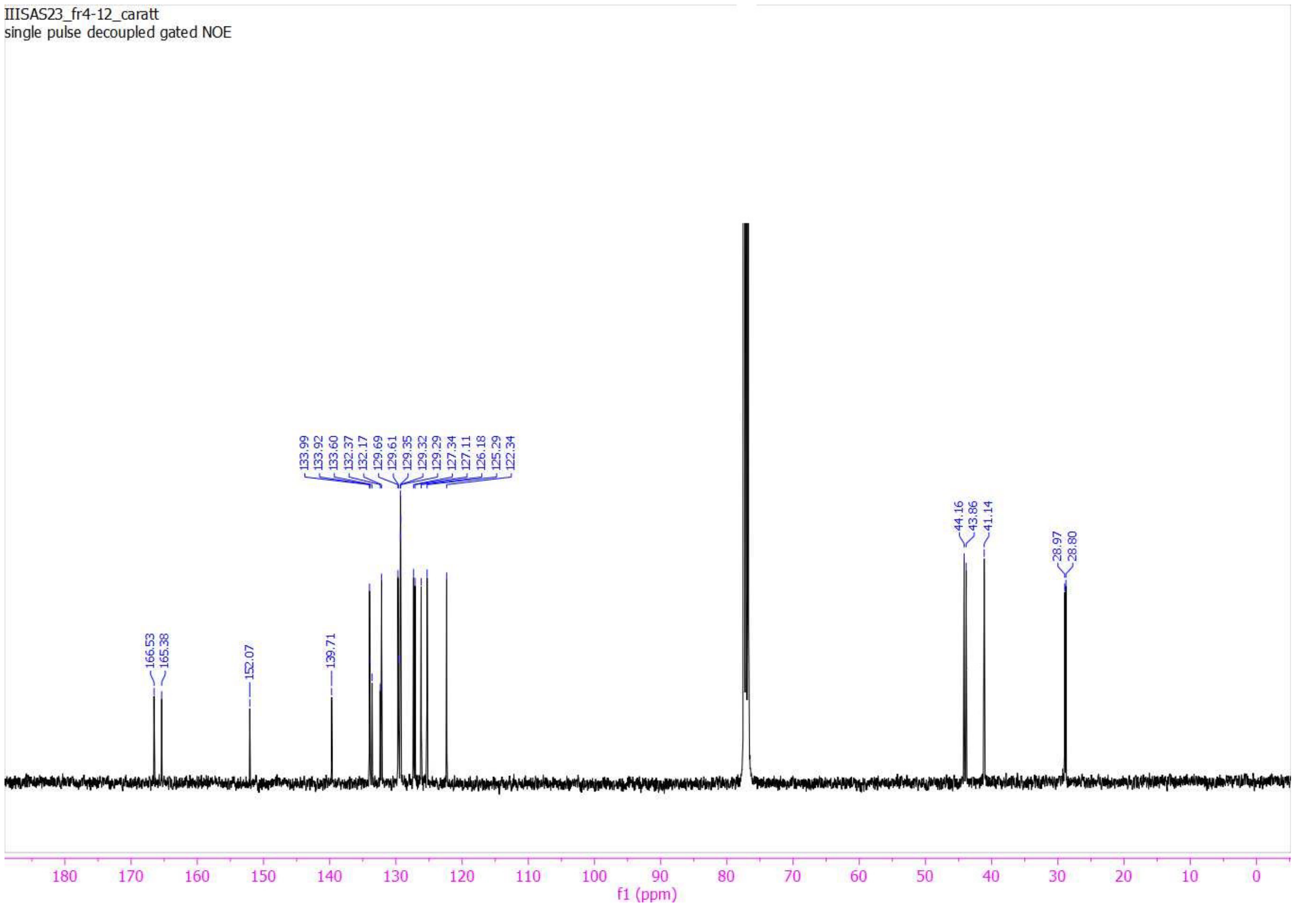
IIISAS23_fr4-12_caratt
single pulse decoupled gated NOE
166.53 165.38
152.07
139.71
133.99 133.92 133.60 132.37 132.17 129.69 129.61 129.35 129.32 129.29 127.34 127.11 126.18 125.29 122.34
44.16 43.86 41.14
28.97 28.80
f1 (ppm)

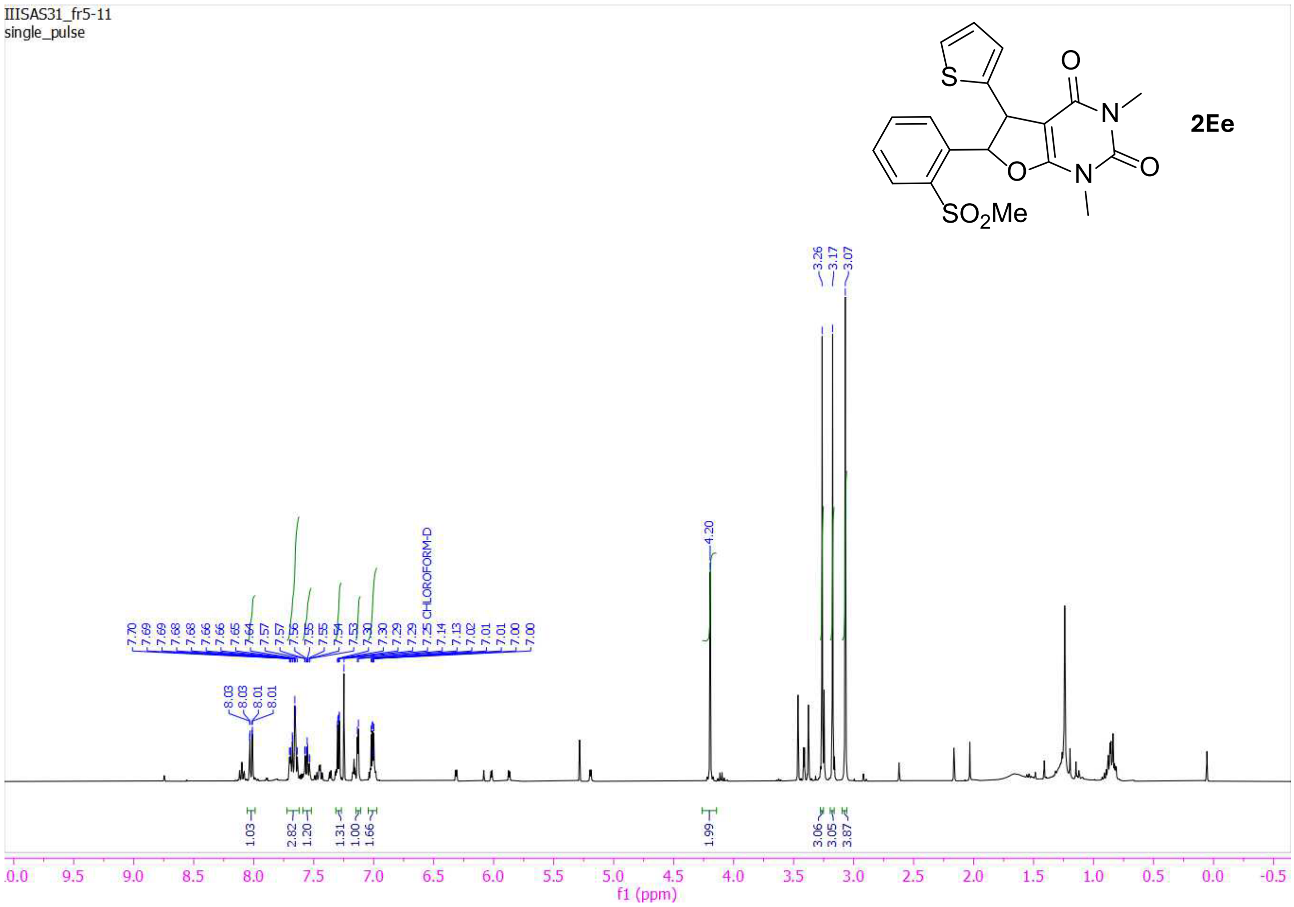

IIISAS31_fr5-11
single_pulse
2Ee
SO2Me
CHLOROFORM-D
f1 (ppm)

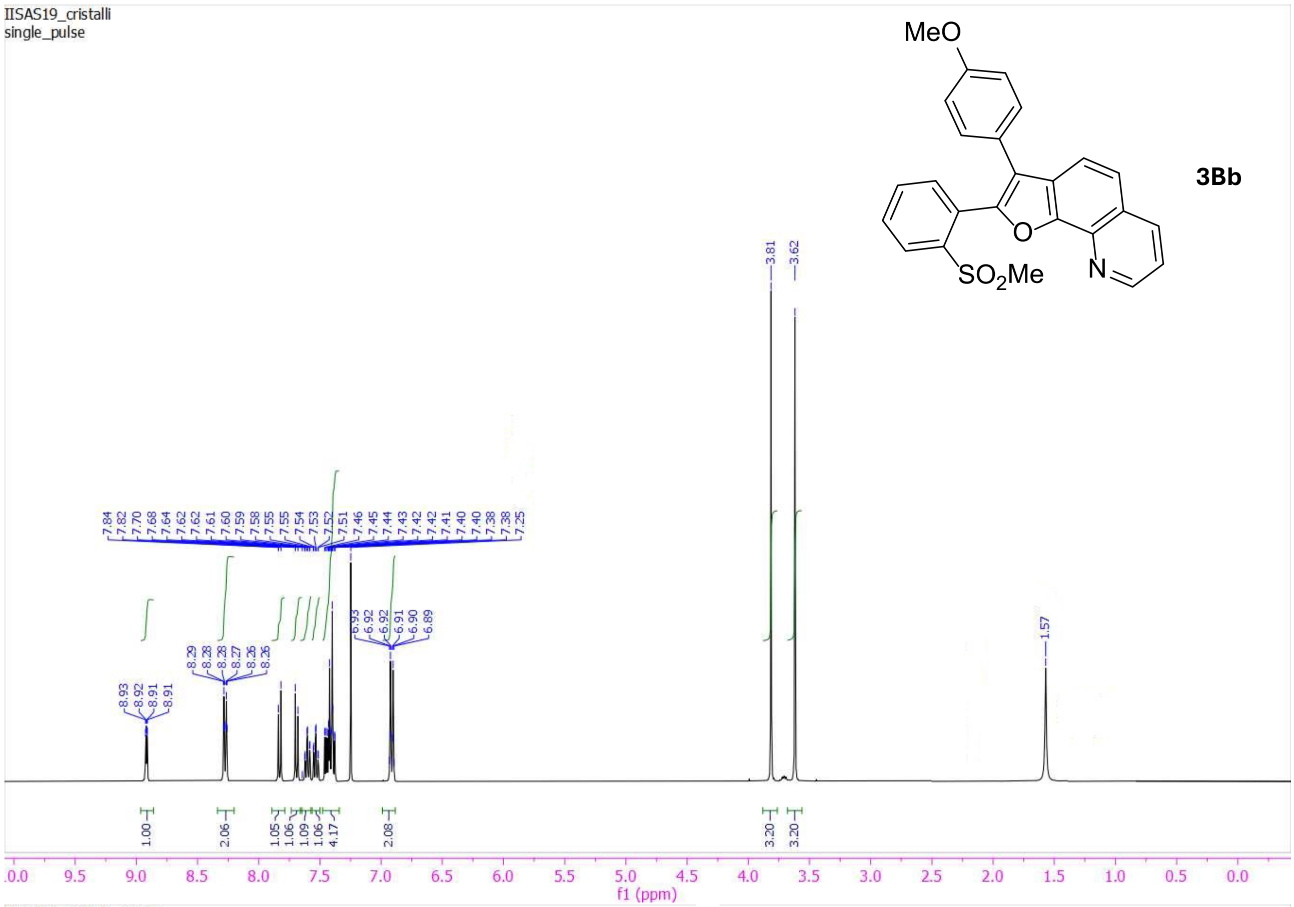
IISAS19_cristalli
single_pulse
MeO
O
N
SO2Me
3Bb
8.93 8.92 8.91 8.91
8.29 8.28 8.28 8.27 8.26 8.26
7.84 7.82 7.70 7.68 7.64 7.62 7.62 7.61 7.60 7.59 7.58 7.55 7.55 7.54 7.53 7.52 7.51 7.46 7.45 7.44 7.43 7.42 7.42 7.41 7.40 7.40 7.38 7.38 7.25
6.93 6.92 6.92 6.91 6.90 6.89
3.81
3.62
1.57
1.00
2.06
1.05 1.06 1.09 1.06 4.17
2.08
3.20
3.20
f1 (ppm)

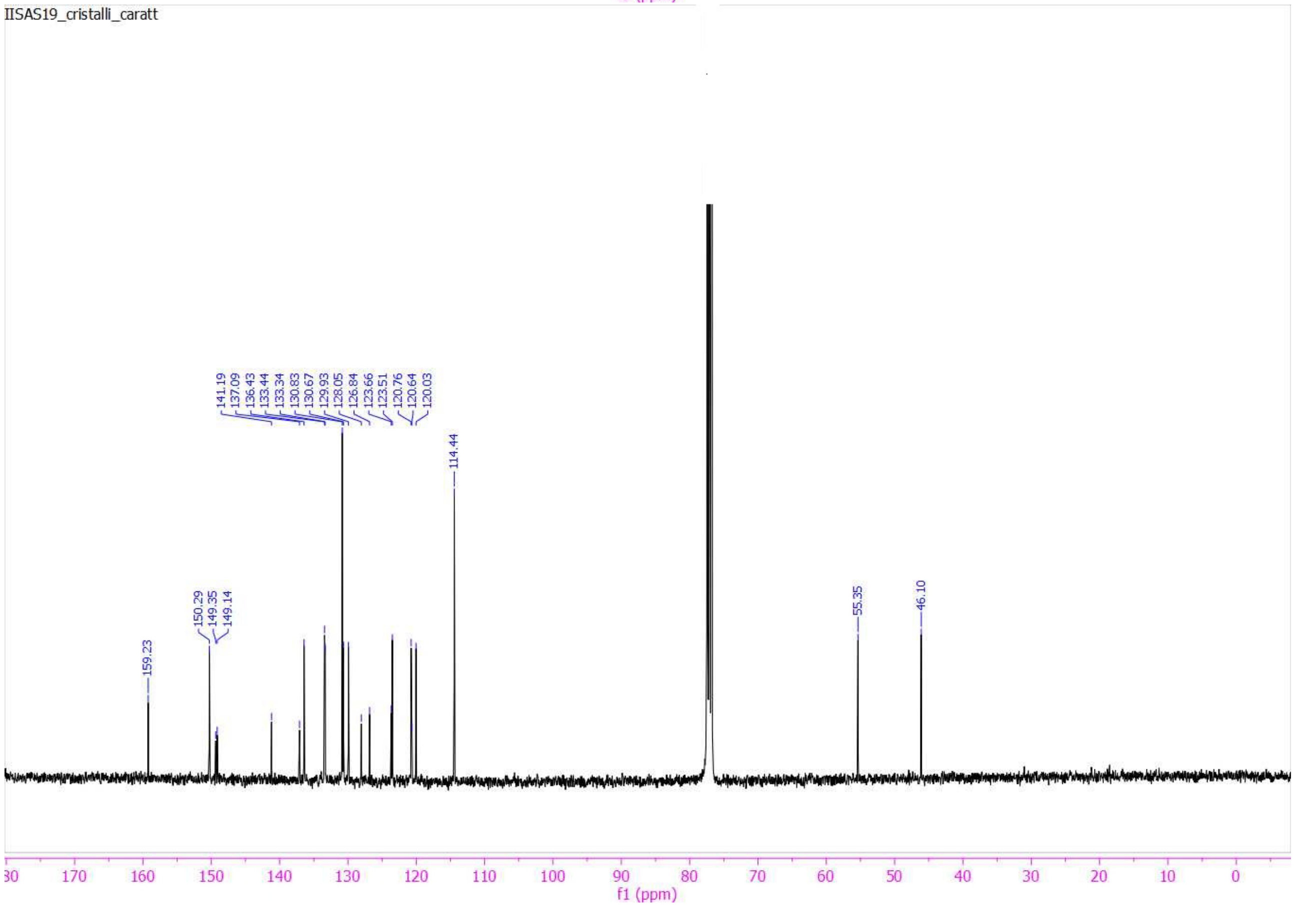
IISAS19_cristalli_caratt
159.23
150.29 149.35 149.14
141.19 137.09 136.43 133.44 133.34 130.83 130.67 129.93 128.05 126.84 123.66 123.51 120.76 120.64 120.03
114.44
55.35
46.10
f1 (ppm)

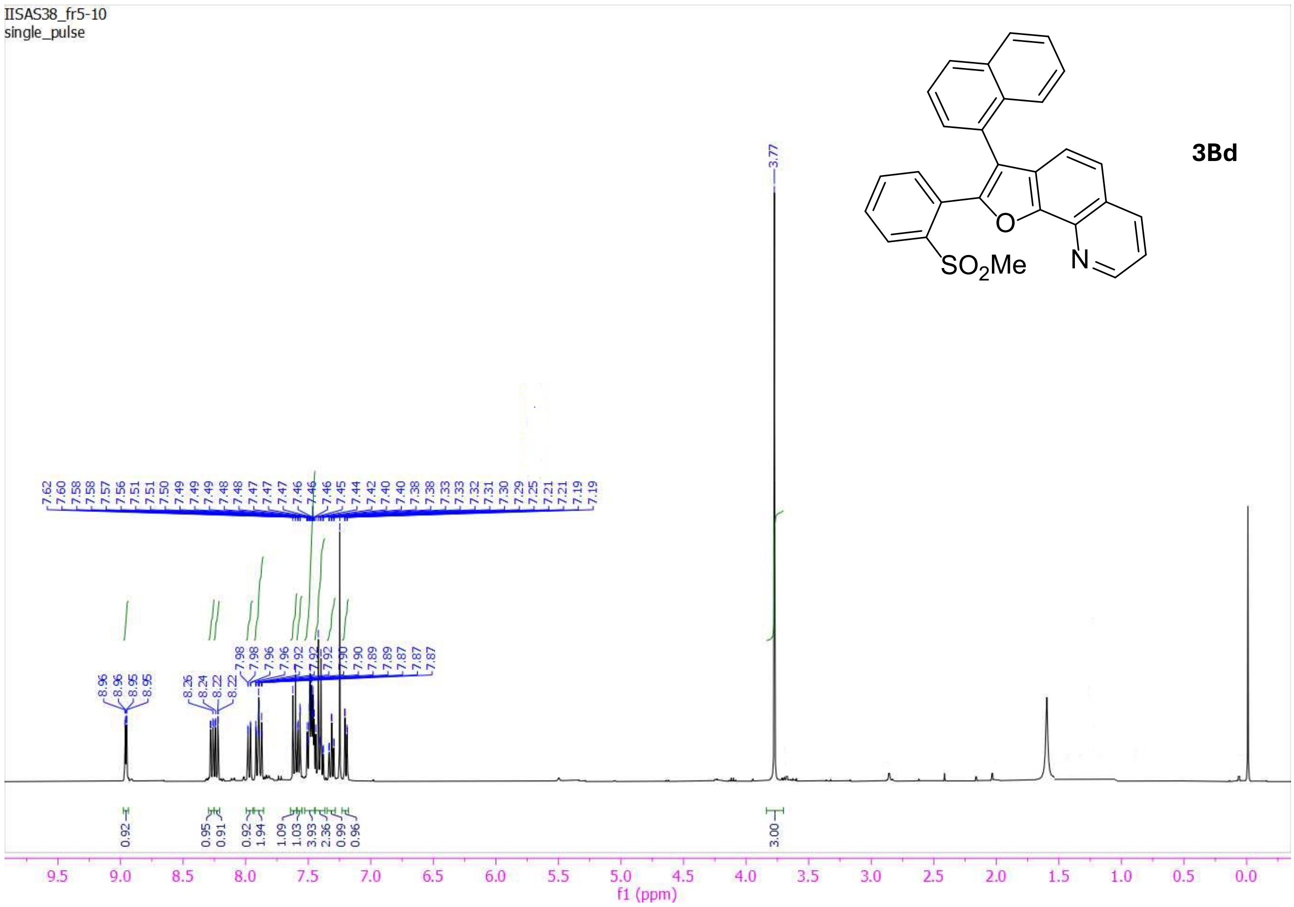
IISAS38_fr5-10
single_pulse
3Bd
SO2Me
O
N
3.77
7.62 7.60 7.58 7.58 7.57 7.56 7.51 7.51 7.50 7.49 7.49 7.48 7.48 7.47 7.47 7.47 7.46 7.46 7.46 7.45 7.44 7.42 7.40 7.38 7.38 7.33 7.33 7.32 7.31 7.30 7.29 7.25 7.21 7.21 7.19 7.19
8.96 8.96 8.95 8.95
8.26 8.24 8.22 8.22
7.98 7.98 7.96 7.96 7.92 7.92 7.92 7.90 7.89 7.87 7.87 7.87
0.92 0.95 0.91 0.92 1.94 1.09 1.03 3.93 2.36 0.99 0.96 3.00
f1 (ppm)

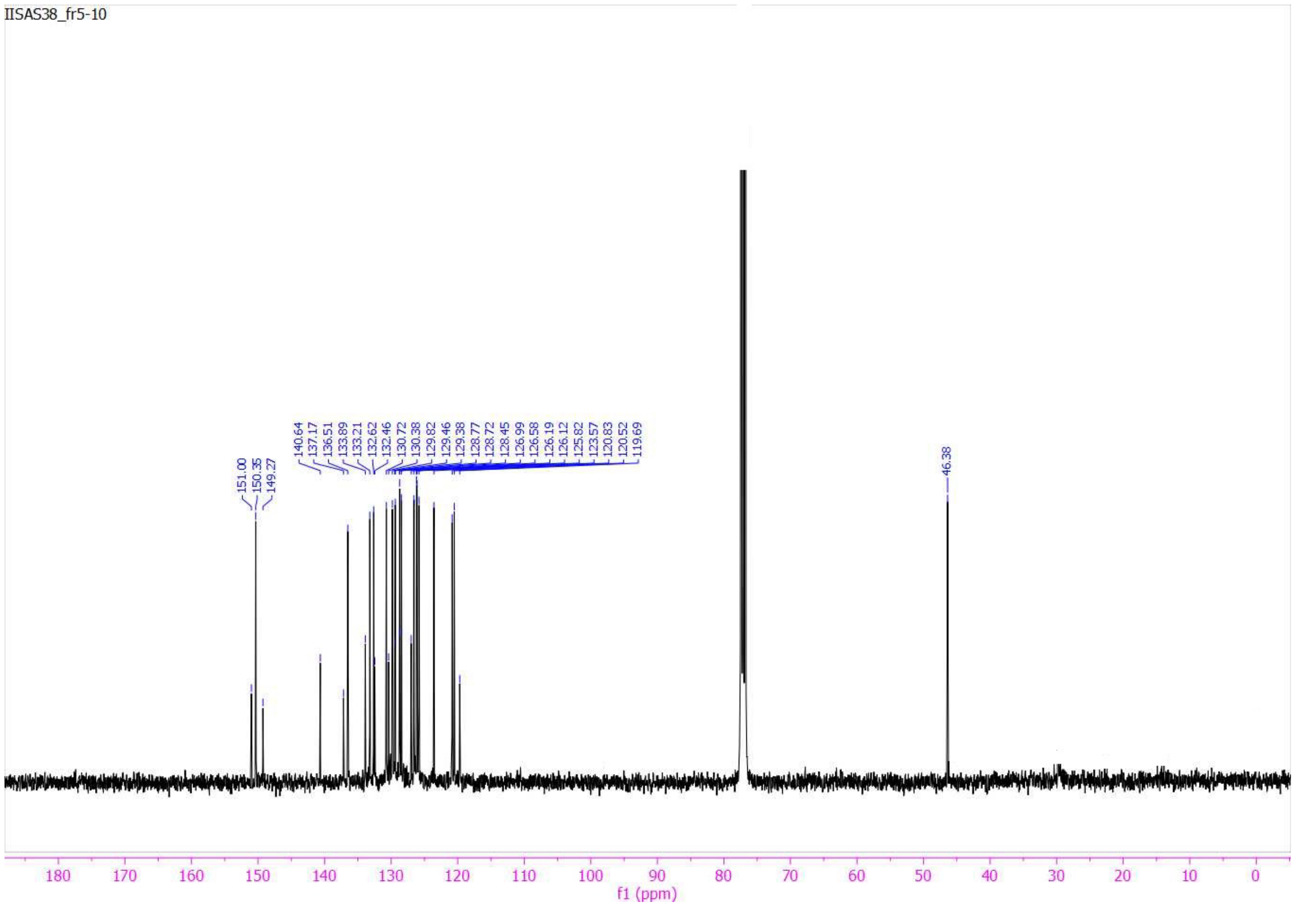
IISAS38_fr5-10
151.00 150.35 149.27
140.64 137.17 136.51 133.89 133.21 132.62 132.46 130.72 130.38 129.82 129.46 129.38 128.77 128.72 128.45 126.99 126.58 126.19 126.12 125.82 123.57 120.83 120.52 119.69
46.38
f1 (ppm)

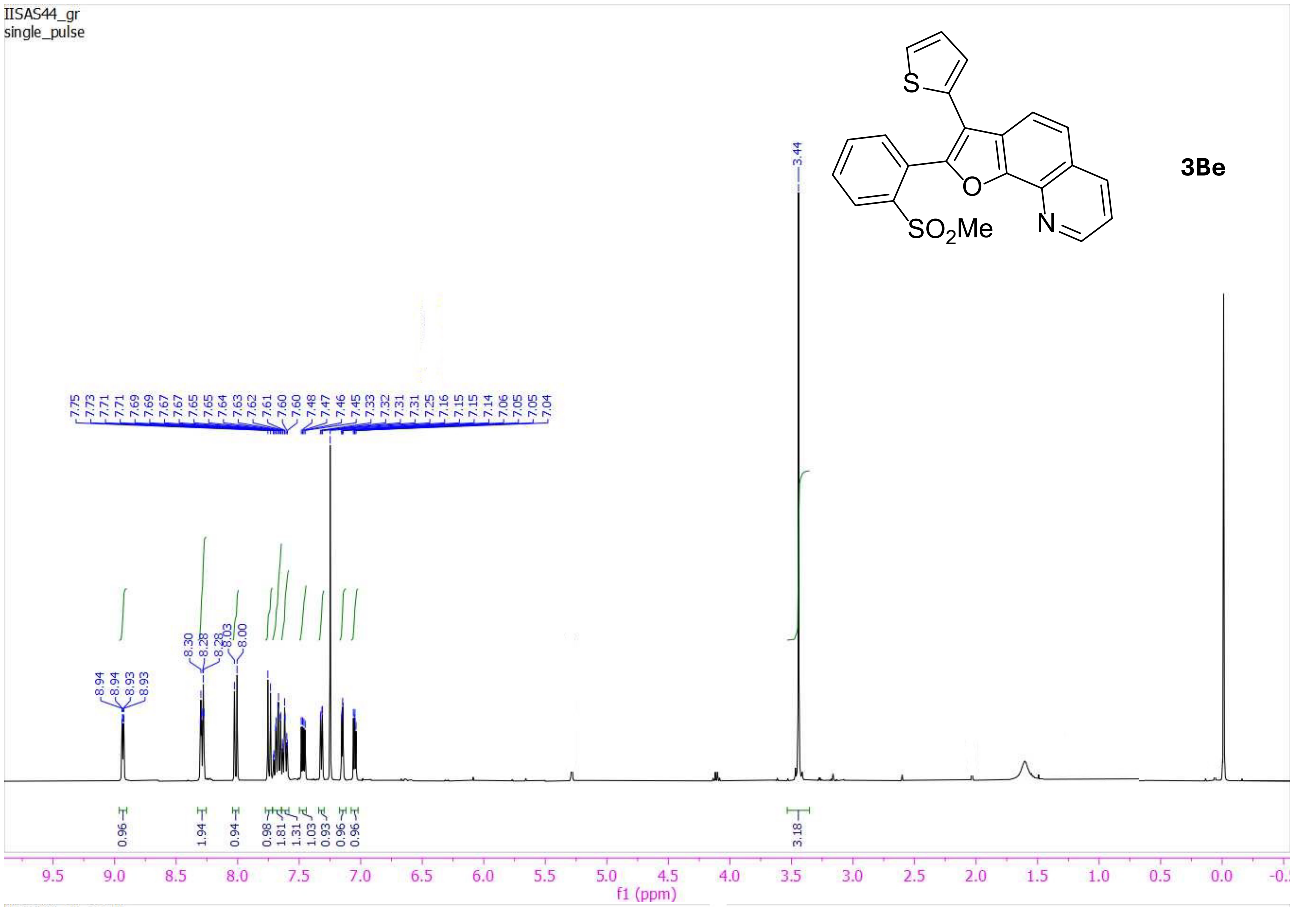
IISAS44_gr
single_pulse
3Be
S
O
SO2Me
N
7.75 7.73 7.71 7.71 7.69 7.69 7.67 7.65 7.65 7.64 7.63 7.62 7.61 7.60 7.60 7.48 7.47 7.46 7.45 7.33 7.32 7.31 7.31 7.25 7.16 7.15 7.15 7.14 7.06 7.05 7.05 7.04
8.94 8.94 8.93 8.93
8.30 8.28 8.28
8.03 8.00
3.44
0.96
1.94
0.94
0.98 1.81 1.31 1.03 0.93 0.96 0.96
3.18
9.5 9.0 8.5 8.0 7.5 7.0 6.5 6.0 5.5 5.0 4.5 4.0 3.5 3.0 2.5 2.0 1.5 1.0 0.5 0.0
f1 (ppm)

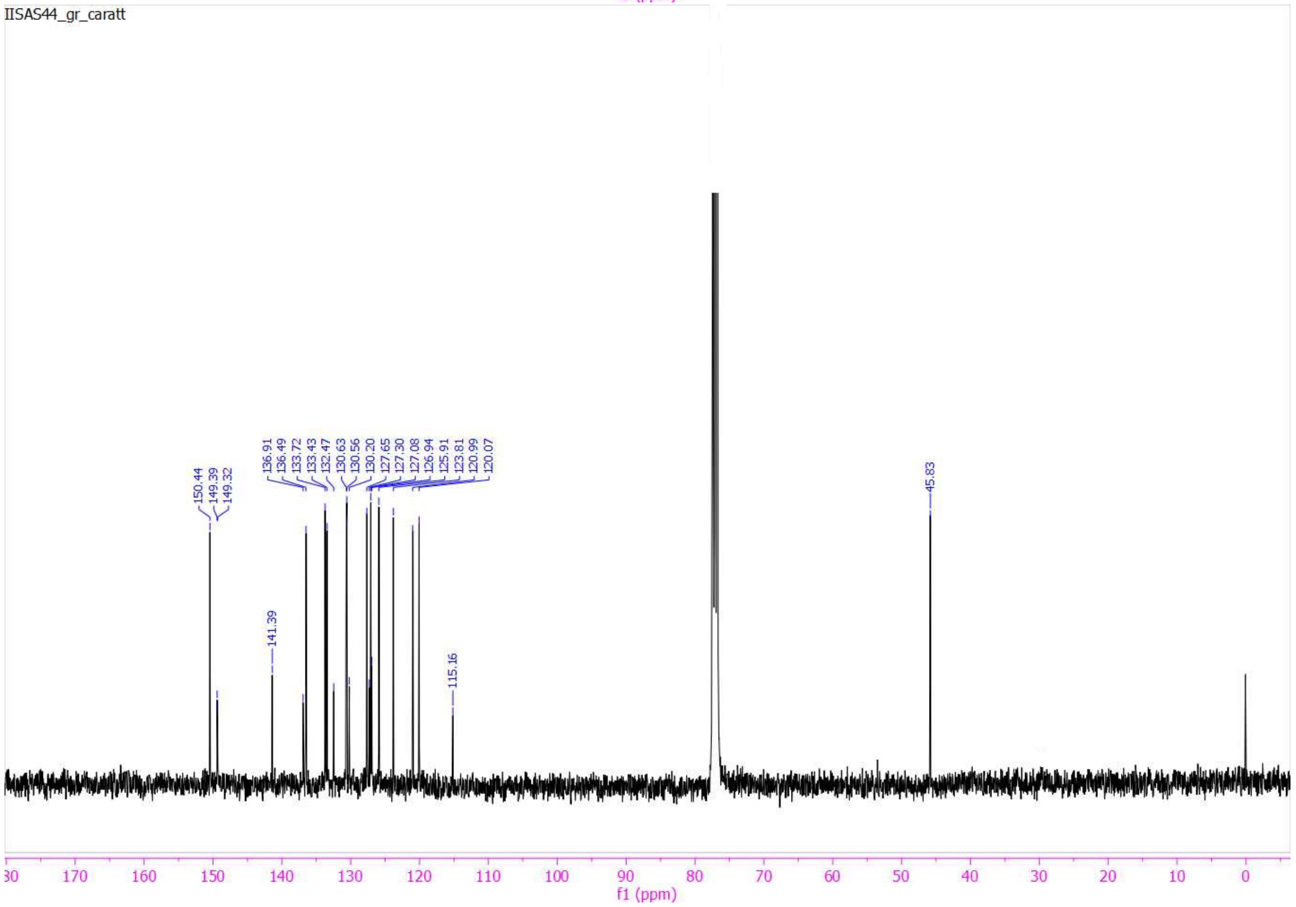
IISAS44_gr_caratt
150.44 149.39 149.32
141.39
136.91 136.49 133.72 133.43 132.47 130.63 130.56 130.20 127.65 127.30 127.08 126.94 125.91 123.81 120.99 120.07
115.16
45.83
170 160 150 140 130 120 110 100 90 80 70 60 50 40 30 20 10 0
f1 (ppm)

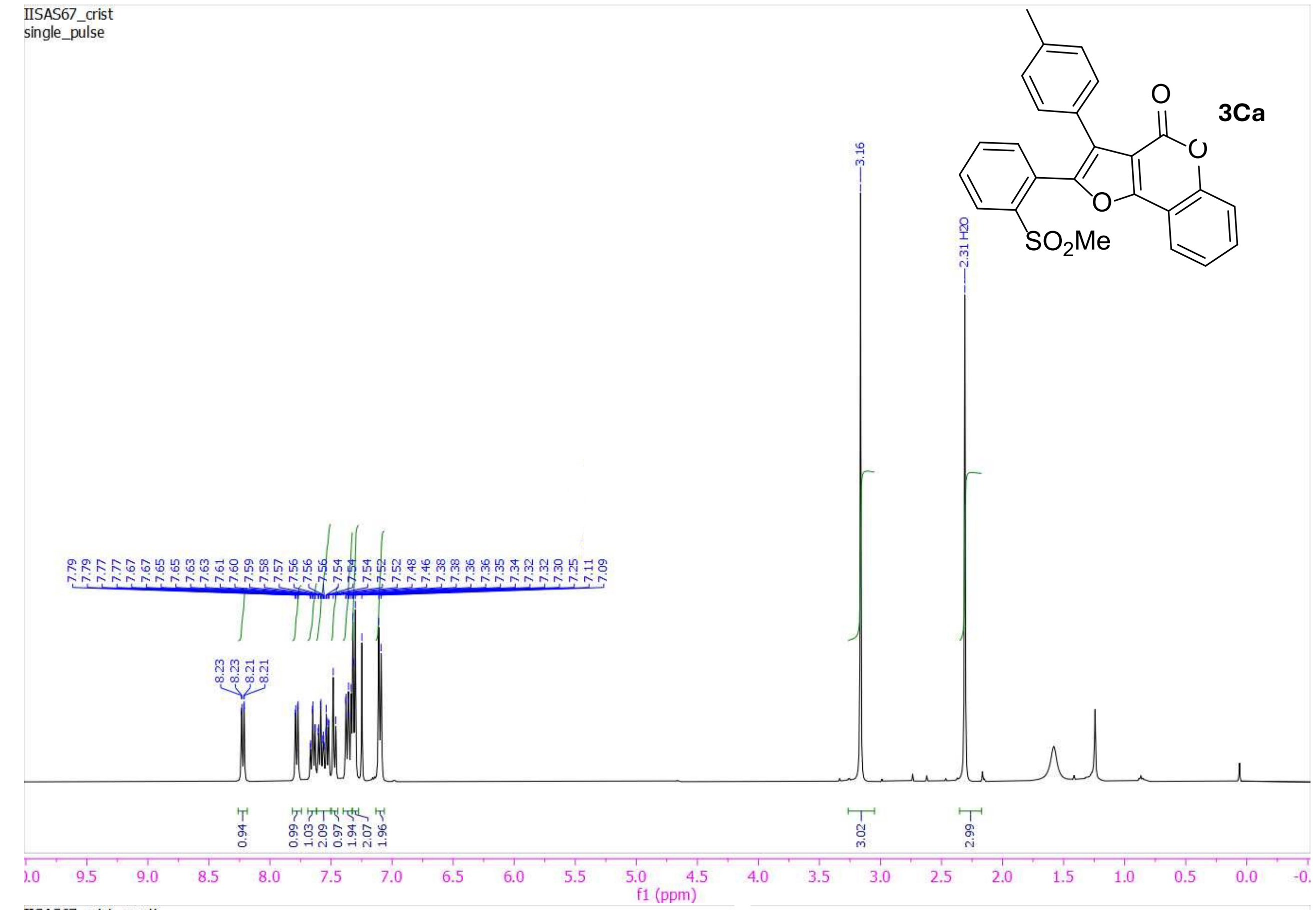
IISAS67_crist
single_pulse
3Ca
O
O
O
SO2Me
3.16
2.31 H2O
7.79 7.79 7.77 7.77 7.67 7.67 7.65 7.65 7.63 7.63 7.61 7.60 7.59 7.58 7.57 7.56 7.56 7.56 7.54 7.54 7.54 7.52 7.52 7.48 7.46 7.38 7.38 7.36 7.36 7.35 7.34 7.32 7.32 7.30 7.25 7.11 7.09
8.23 8.23 8.21 8.21
0.94 0.99 1.03 2.09 0.97 1.94 2.07 1.96 3.02 2.99
f1 (ppm)

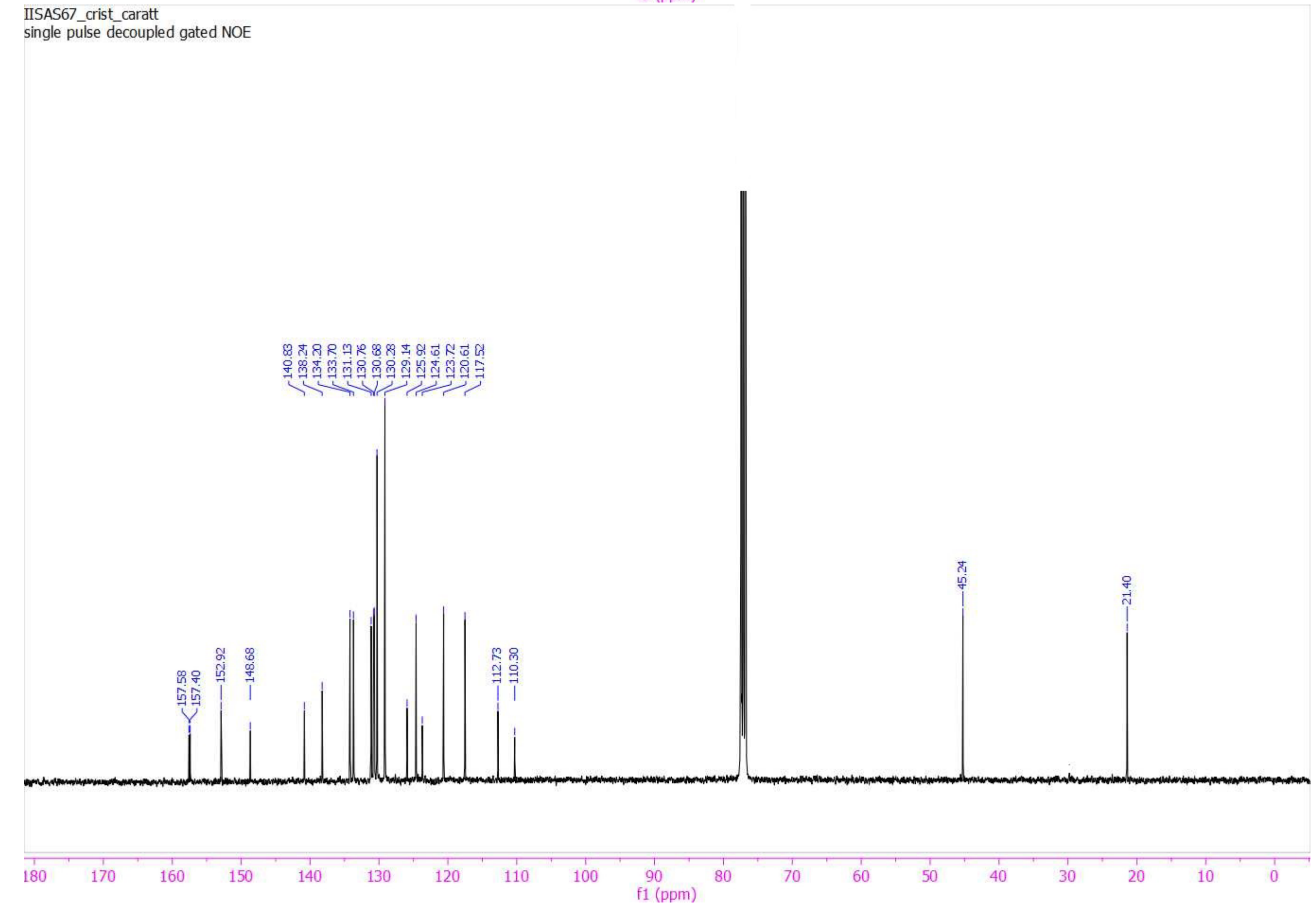
IISAS67_crist_caratt
single pulse decoupled gated NOE
140.83 138.24 134.20 133.70 131.13 130.76 130.68 130.28 129.14 125.92 124.61 123.72 120.61 117.52
157.58 157.40 152.92 148.68 112.73 110.30 45.24 21.40
f1 (ppm)

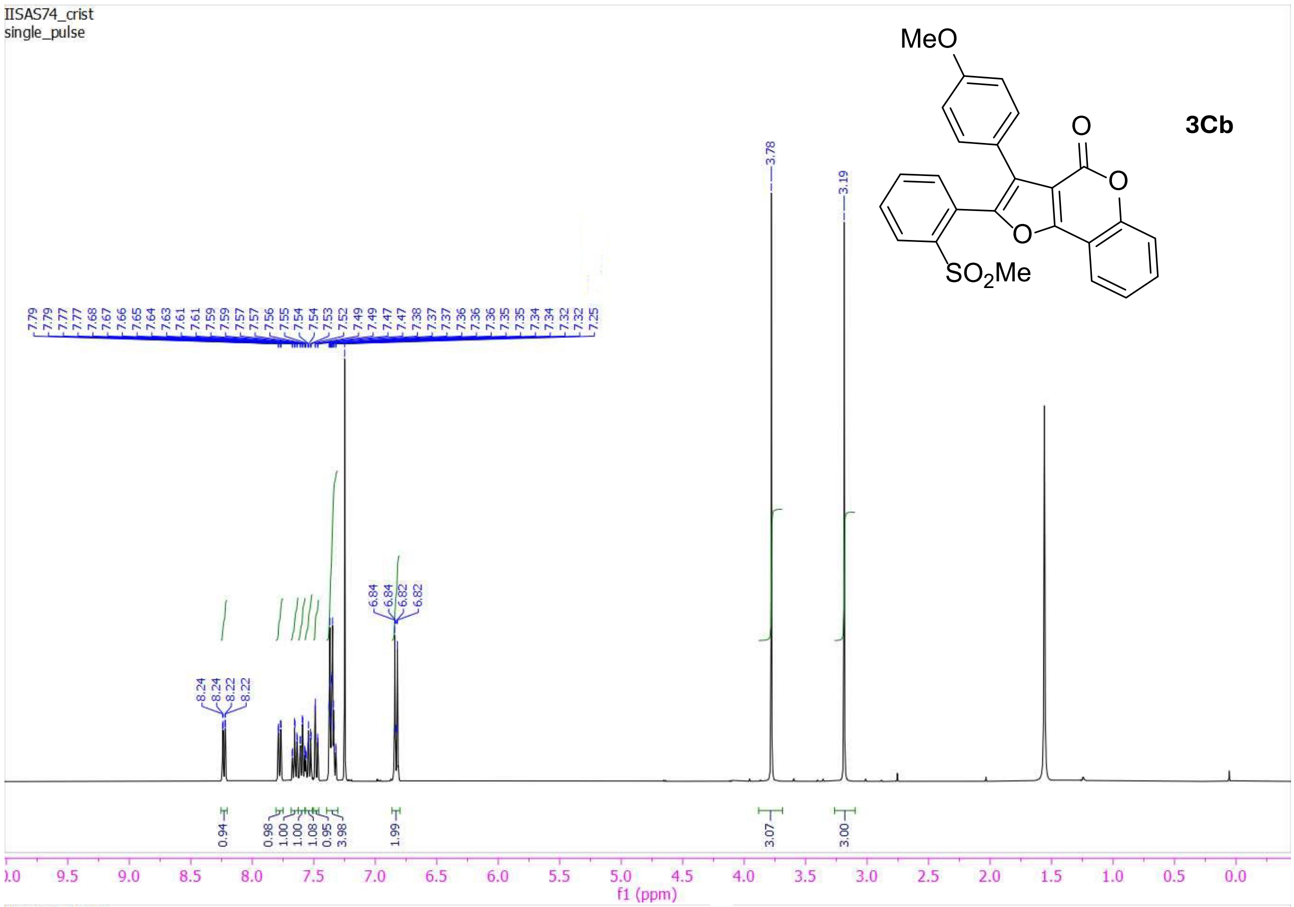
IISAS74_crist
single_pulse
MeO
O
O
O
SO2Me
3Cb
7.79 7.79 7.77 7.77 7.68 7.67 7.66 7.65 7.64 7.63 7.61 7.61 7.59 7.59 7.57 7.57 7.56 7.55 7.54 7.54 7.53 7.52 7.49 7.49 7.47 7.38 7.37 7.37 7.36 7.36 7.36 7.35 7.35 7.34 7.34 7.32 7.32 7.25
8.24 8.24 8.22 8.22
6.84 6.84 6.82 6.82
3.78
3.19
0.94
0.98 1.00 1.00 1.08 0.95 3.98
1.99
3.07
3.00
10.0 9.5 9.0 8.5 8.0 7.5 7.0 6.5 6.0 5.5 5.0 4.5 4.0 3.5 3.0 2.5 2.0 1.5 1.0 0.5 0.0
f1 (ppm)

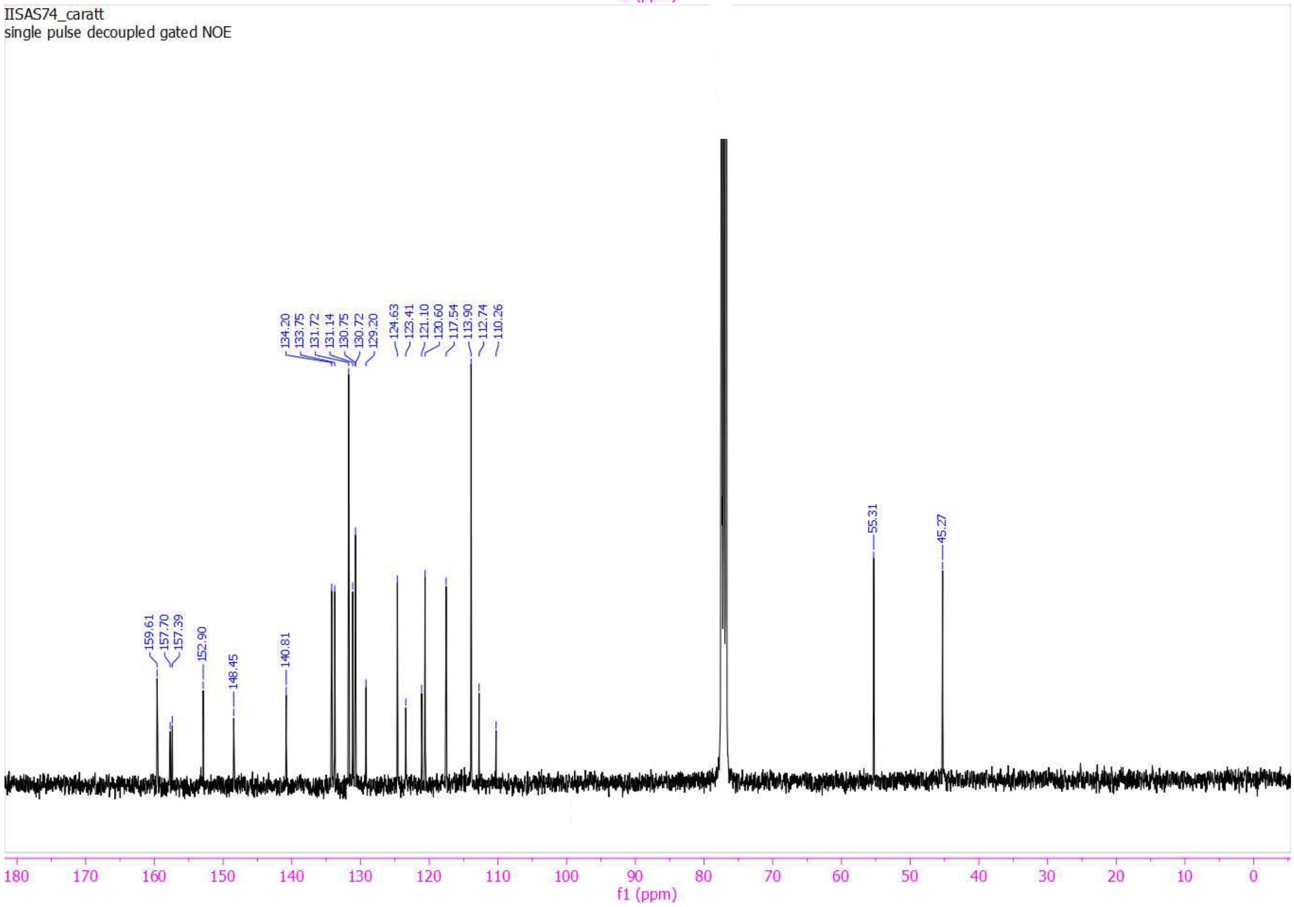
IISAS74_caratt
single pulse decoupled gated NOE
134.20 133.75 131.72 131.14 130.75 130.72 129.20
124.63 123.41 121.10 120.60 117.54 113.90 112.74 110.26
159.61 157.70 157.39
152.90
148.45
140.81
55.31
45.27
180 170 160 150 140 130 120 110 100 90 80 70 60 50 40 30 20 10 0
f1 (ppm)

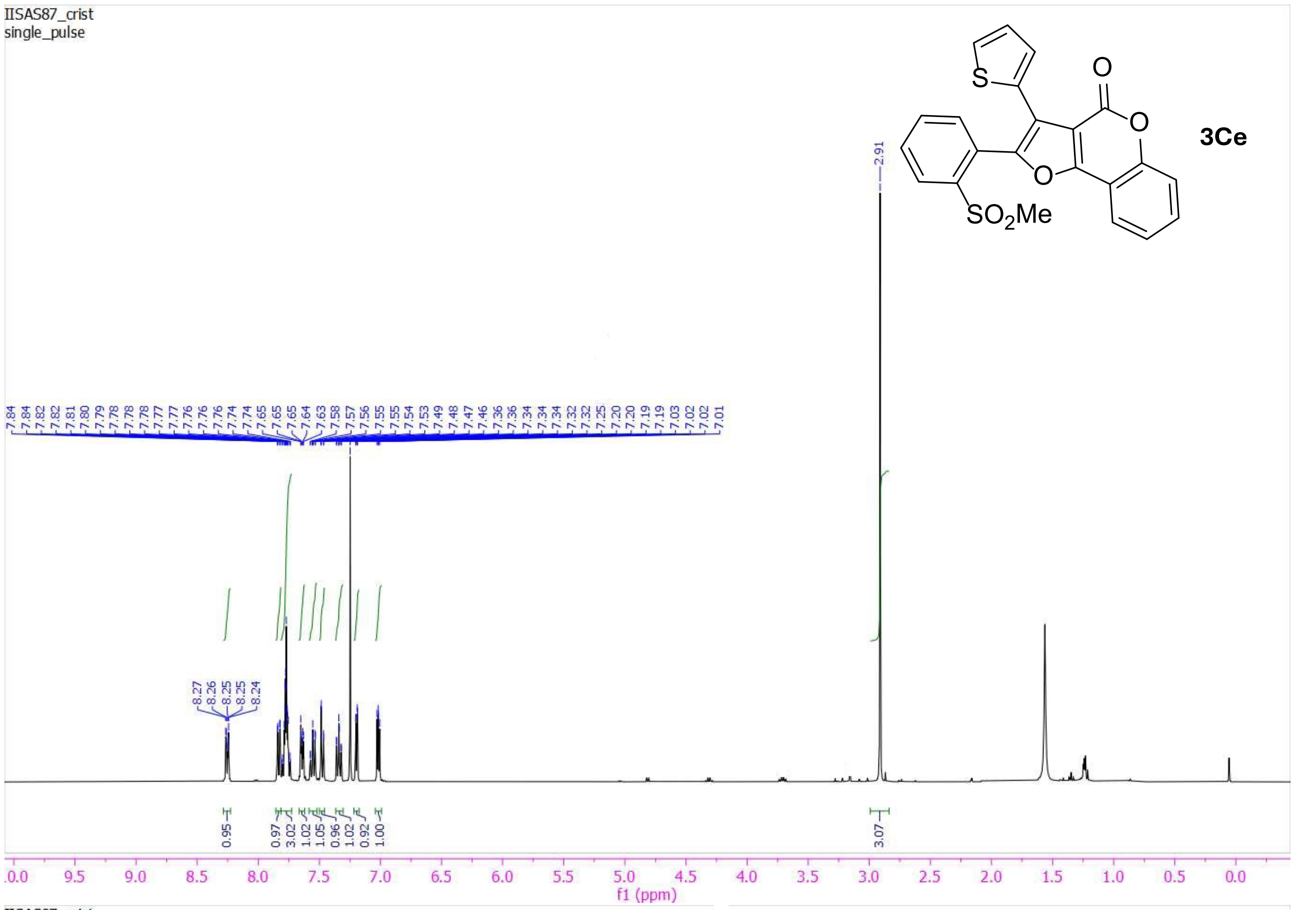

IISAS87_crist
single_pulse
3Ce
SO2Me
S
O
O
O
2.91
8.27 8.26 8.25 8.25 8.24
7.84 7.84 7.82 7.81 7.80 7.79 7.78 7.78 7.78 7.77 7.76 7.76 7.76 7.74 7.65 7.65 7.64 7.63 7.58 7.57 7.56 7.55 7.55 7.54 7.53 7.49 7.48 7.47 7.46 7.36 7.36 7.34 7.34 7.32 7.32 7.25 7.20 7.20 7.19 7.19 7.03 7.02 7.02 7.01
0.95 0.97 3.02 1.02 1.05 0.96 1.02 0.92 1.00 3.07
f1 (ppm)

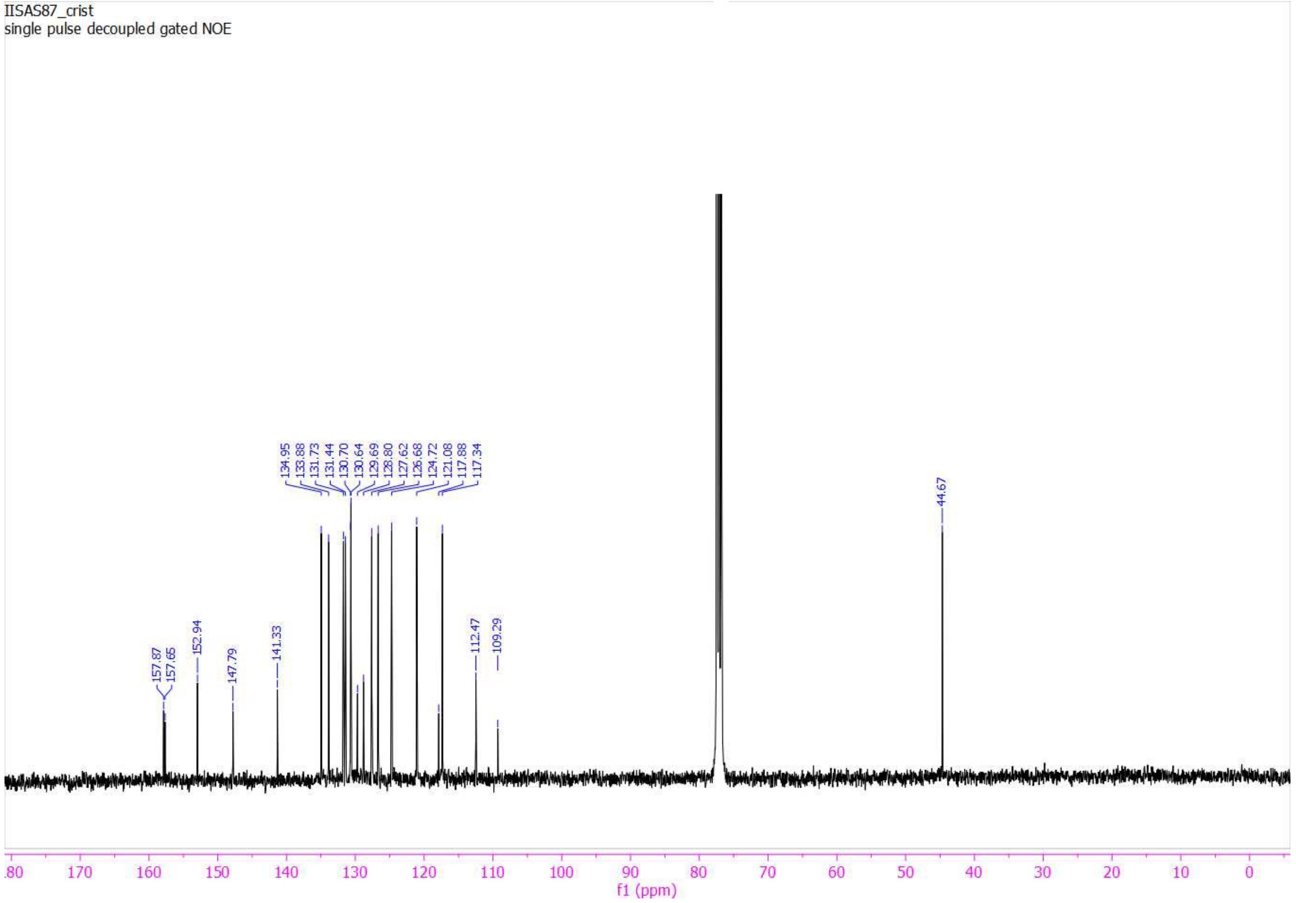

IISAS87_crist
single pulse decoupled gated NOE
157.87 157.65 152.94 147.79 141.33
134.95 133.88 131.73 131.44 130.70 130.64 129.69 128.80 127.62 126.68 124.72 121.08 117.88 117.34
112.47 109.29
44.67
f1 (ppm)

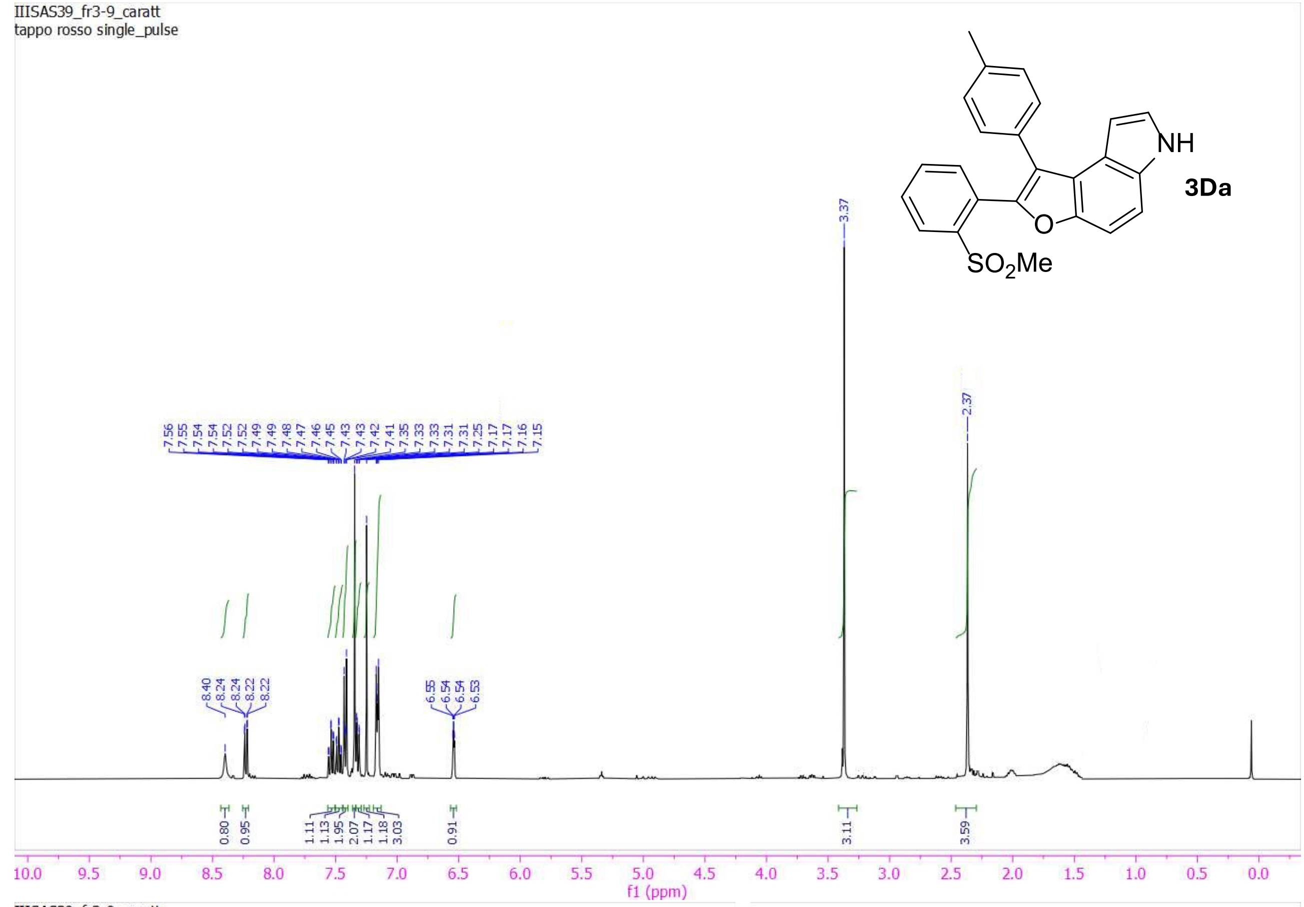
IIISAS39_fr3-9_caratt
tappo rosso single_pulse
NH
3Da
O
SO2Me
7.56 7.55 7.54 7.54 7.52 7.52 7.49 7.48 7.47 7.46 7.45 7.43 7.43 7.42 7.41 7.35 7.33 7.33 7.31 7.31 7.25 7.17 7.17 7.16 7.15
3.37
2.37
8.40 8.24 8.24 8.22 8.22
6.55 6.54 6.54 6.53
0.80 0.95 1.11 1.13 1.95 2.07 1.17 1.18 3.03 0.91 3.11 3.59
10.0 9.5 9.0 8.5 8.0 7.5 7.0 6.5 6.0 5.5 5.0 4.5 4.0 3.5 3.0 2.5 2.0 1.5 1.0 0.5 0.0
f1 (ppm)

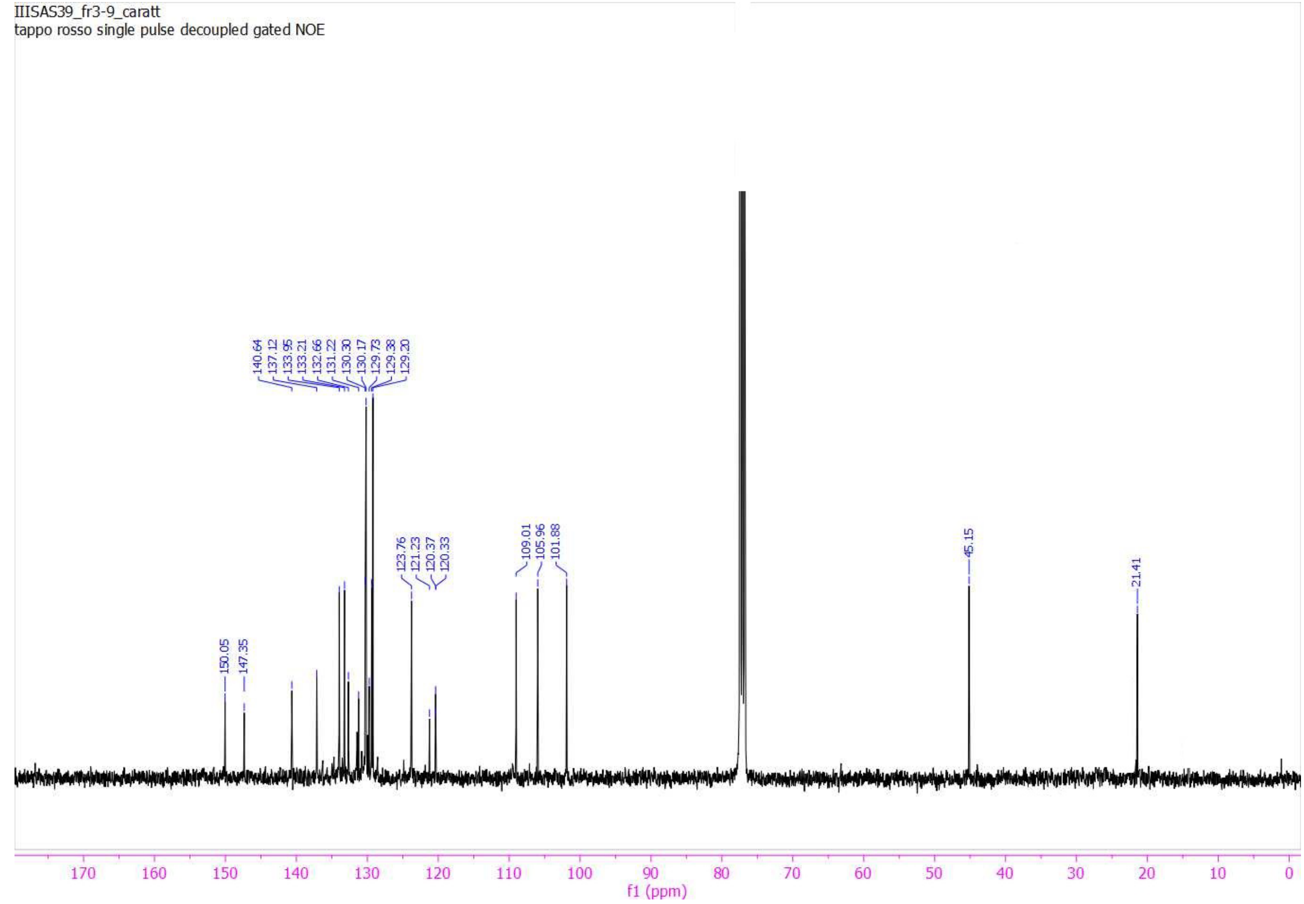
IIISAS39_fr3-9_caratt
tappo rosso single pulse decoupled gated NOE
140.64 137.12 133.95 133.21 132.66 131.22 130.30 130.17 129.73 129.38 129.20
123.76 121.23 120.37 120.33
109.01 105.96 101.88
45.15
21.41
150.05 147.35
170 160 150 140 130 120 110 100 90 80 70 60 50 40 30 20 10 0
f1 (ppm)

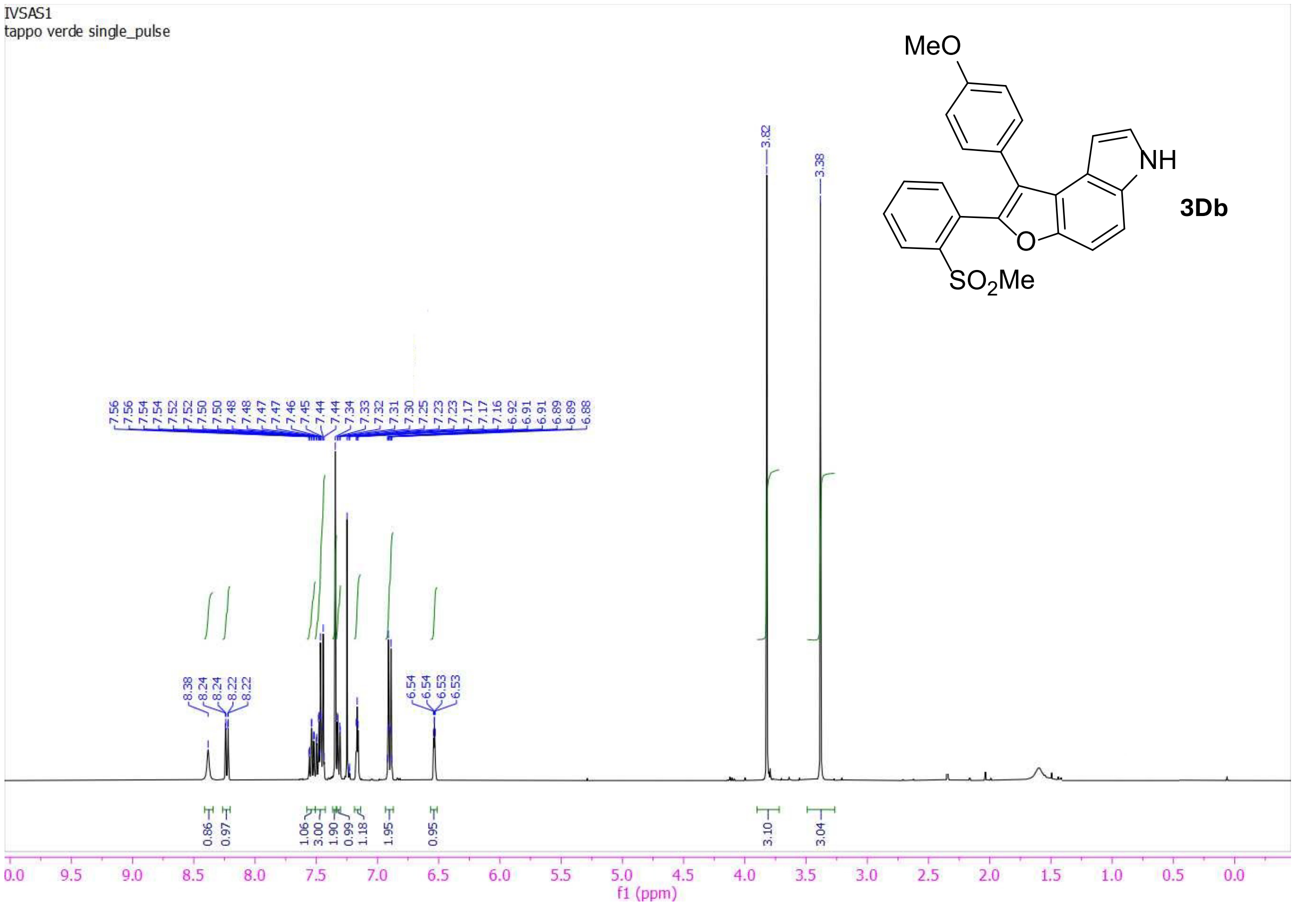
IVSAS1
tappo verde single_pulse
MeO
NH
3Db
SO2Me
7.56 7.56 7.54 7.54 7.52 7.52 7.50 7.50 7.48 7.48 7.47 7.46 7.45 7.44 7.44 7.34 7.33 7.32 7.31 7.30 7.25 7.23 7.23 7.17 7.17 7.16 6.92 6.91 6.91 6.89 6.89 6.88
3.82
3.38
8.38 8.24 8.24 8.22 8.22
6.54 6.54 6.53 6.53
0.86 0.97 1.06 3.00 1.90 0.99 1.18 1.95 0.95 3.10 3.04
f1 (ppm)

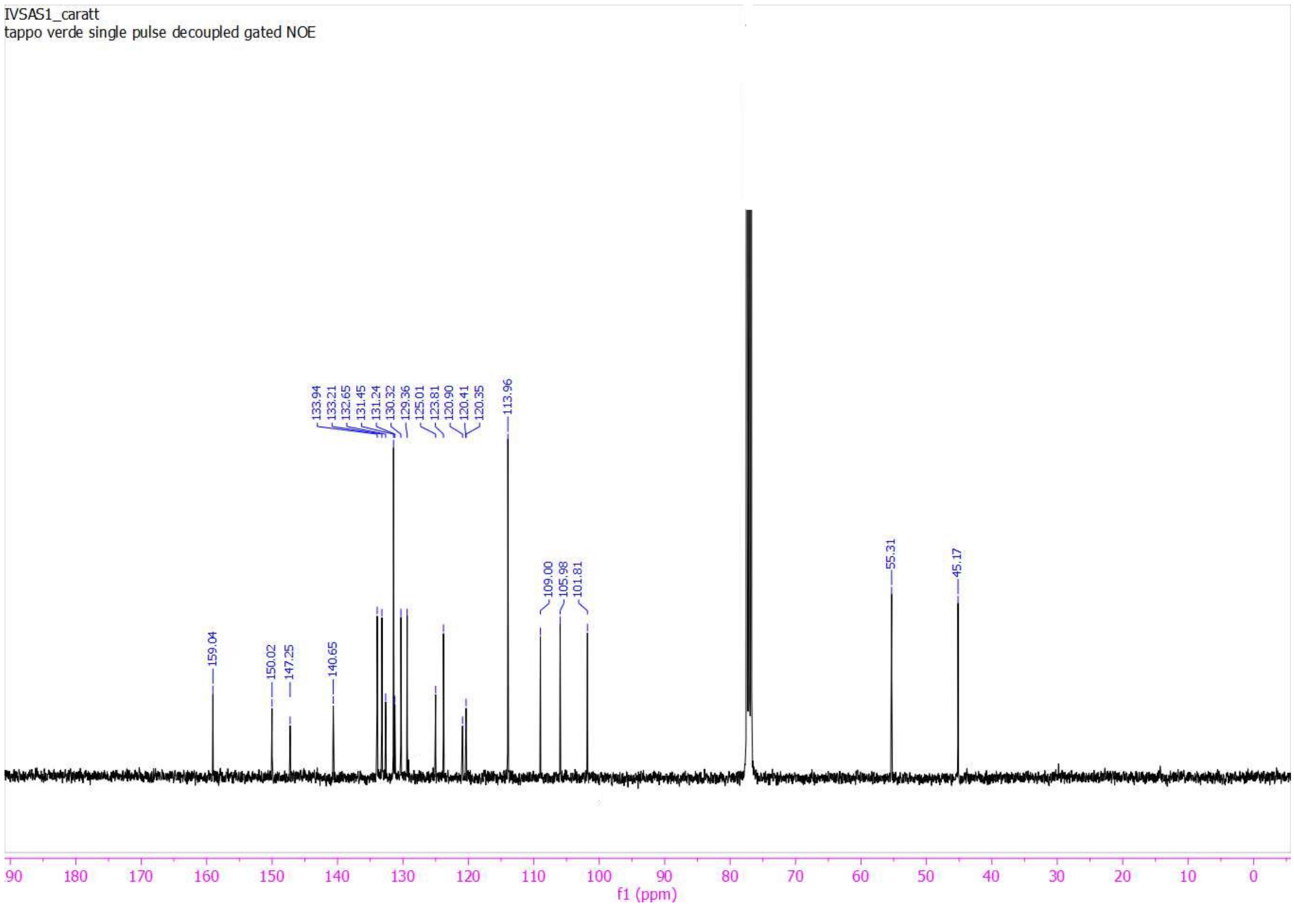
IVSAS1_caratt
tappo verde single pulse decoupled gated NOE
133.94 133.21 132.65 131.45 131.24 130.32 129.36 125.01 123.81 120.90 120.41 120.35
113.96
159.04
150.02 147.25
140.65
109.00 105.98 101.81
55.31
45.17
f1 (ppm)

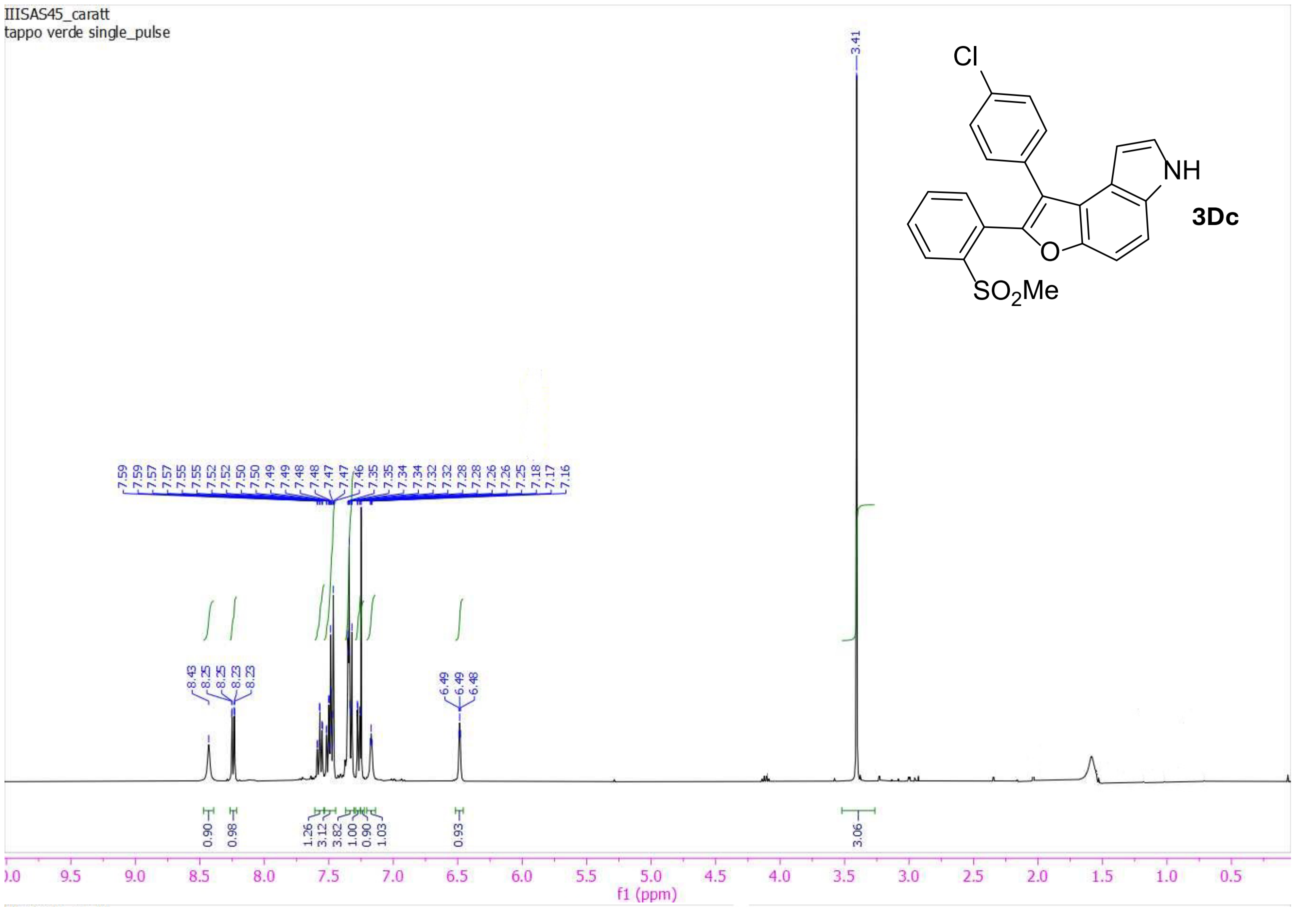
IIISAS45_caratt
tappo verde single_pulse
Cl
NH
O
SO2Me
3Dc
3.41
7.59 7.59 7.57 7.55 7.55 7.52 7.52 7.50 7.50 7.49 7.49 7.48 7.47 7.47 7.46 7.35 7.35 7.34 7.34 7.32 7.32 7.28 7.28 7.26 7.26 7.25 7.18 7.17 7.16
8.43 8.25 8.25 8.23 8.23
6.49 6.49 6.48
0.90 0.98 1.26 3.12 3.82 1.00 0.90 1.03 0.93 3.06
f1 (ppm)

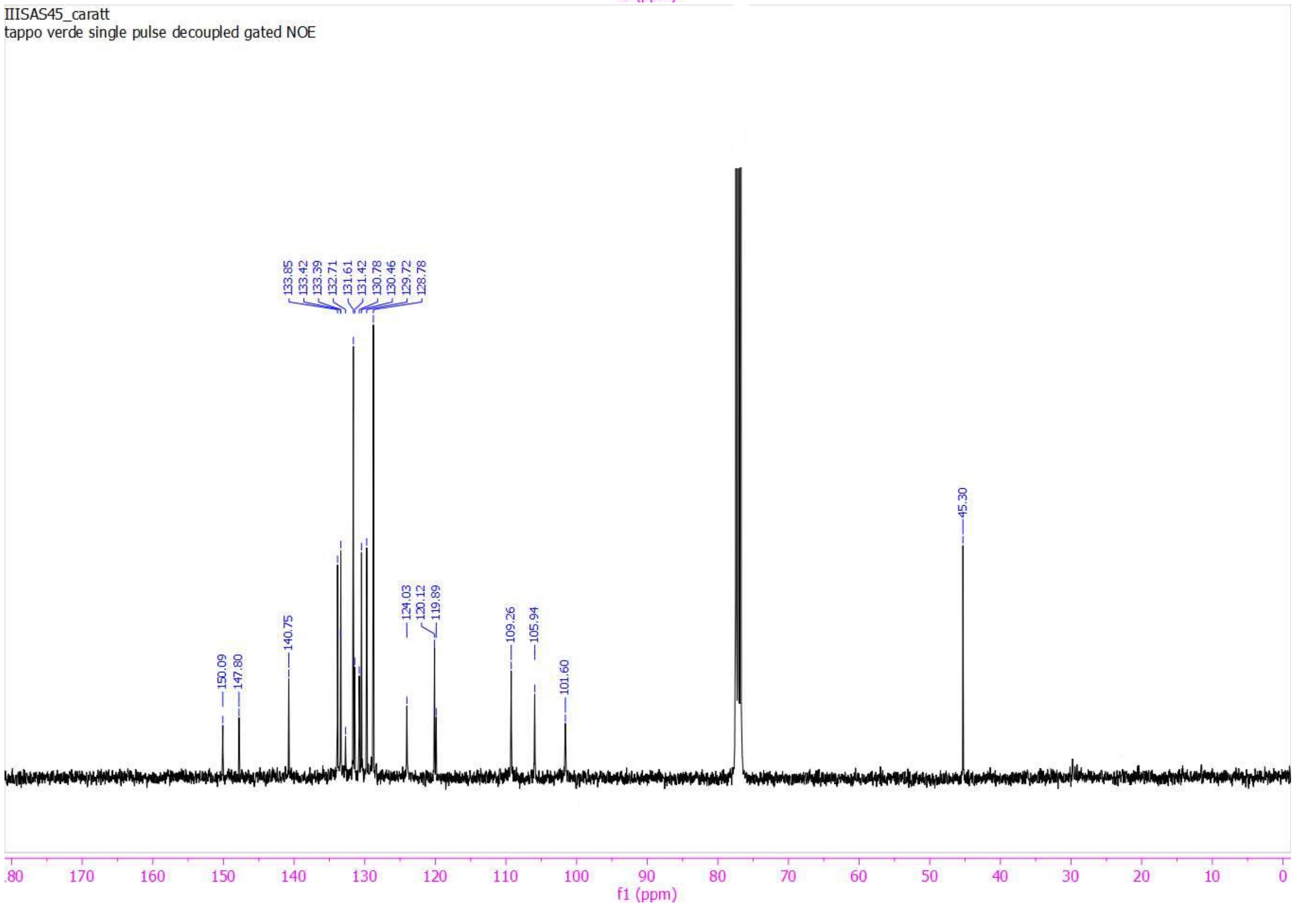
IIISAS45_caratt
tappo verde single pulse decoupled gated NOE
133.85 133.42 133.39 132.71 131.61 131.42 130.78 130.46 129.72 128.78
150.09 147.80 140.75 124.03 120.12 119.89 109.26 105.94 101.60 45.30
f1 (ppm)

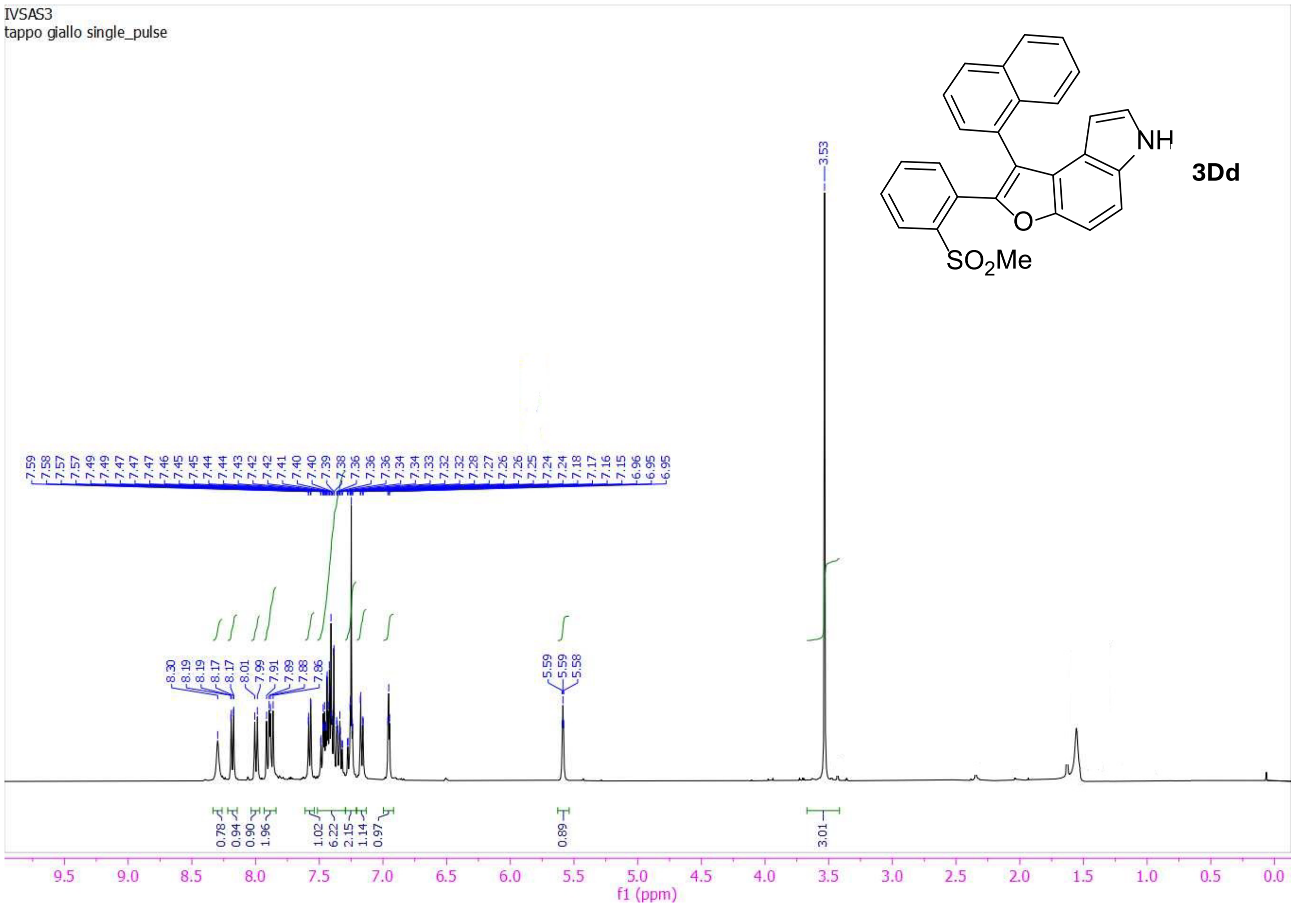
IVSAS3
tappo giallo single_pulse
3Dd
NH
O
SO2Me
3.53
7.59 7.58 7.57 7.57 7.49 7.49 7.47 7.47 7.46 7.45 7.45 7.44 7.44 7.43 7.42 7.42 7.41 7.40 7.39 7.38 7.36 7.36 7.36 7.34 7.33 7.32 7.32 7.28 7.27 7.26 7.26 7.25 7.24 7.18 7.17 7.16 7.15 6.96 6.95 6.95
8.30 8.19 8.17 8.17 8.01 7.99 7.91 7.89 7.88 7.86
5.59 5.59 5.58
0.78 0.94 0.90 1.96 1.02 6.22 2.15 1.14 0.97 0.89 3.01
f1 (ppm)

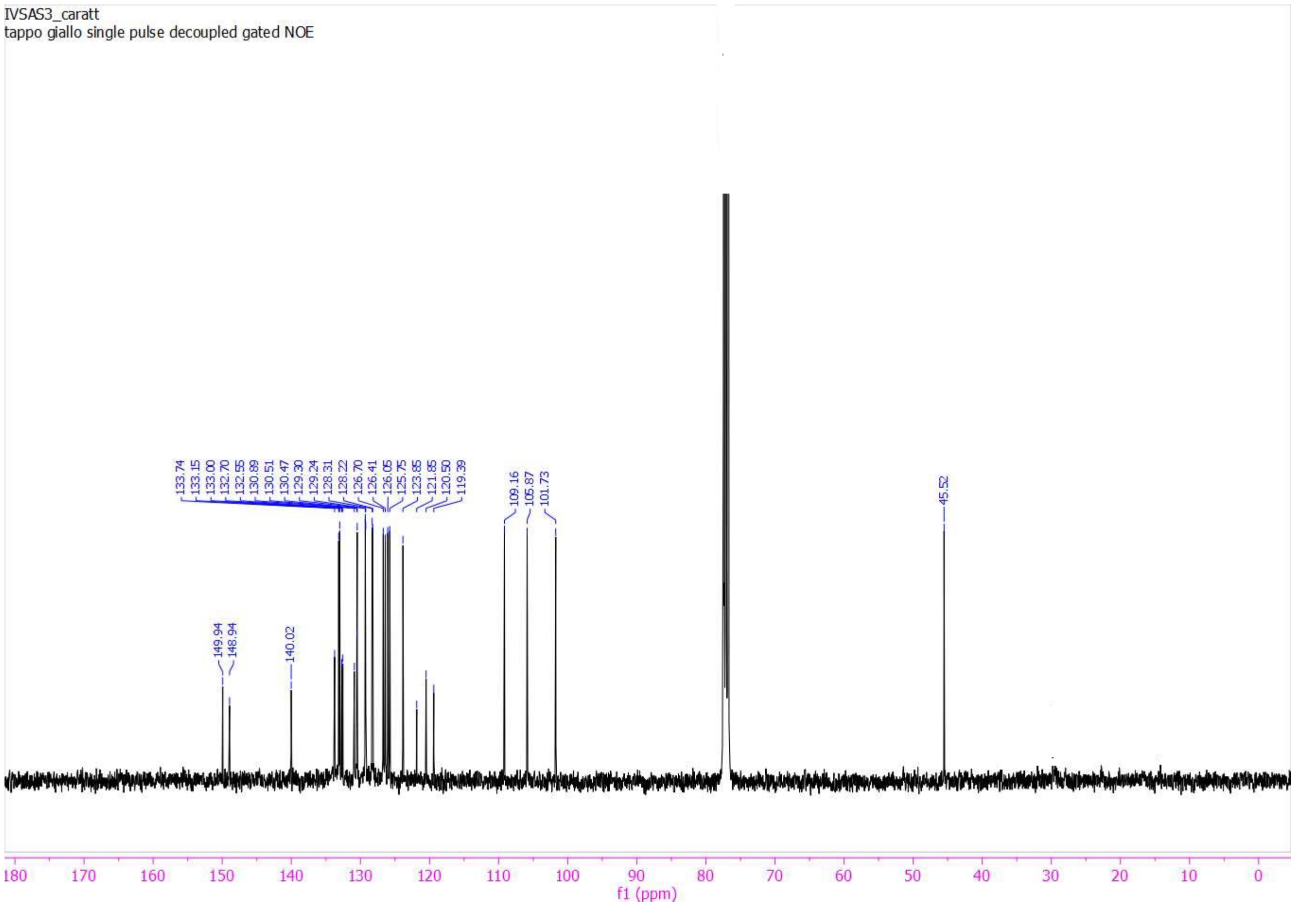
IVSAS3_caratt
tappo giallo single pulse decoupled gated NOE
133.74 133.15 133.00 132.70 132.55 130.89 130.51 130.47 129.30 129.24 128.31 128.22 126.70 126.41 126.05 125.75 123.85 121.85 120.50 119.39
109.16 105.87 101.73
45.52
149.94 148.94
140.02
f1 (ppm)

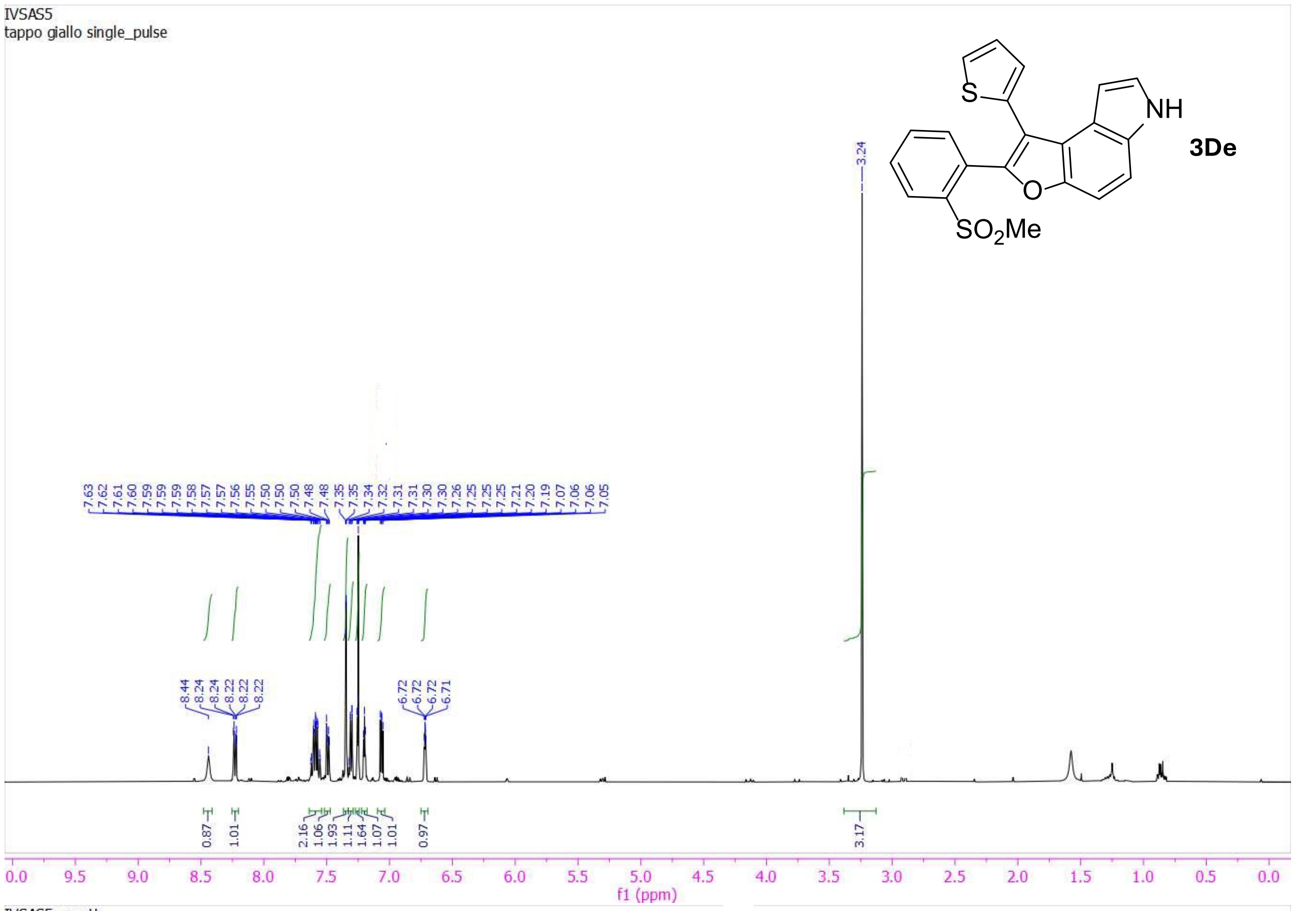
IVSAS5
tappo giallo single_pulse
NH
S
O
SO2Me
3De
3.24
7.63 7.62 7.61 7.60 7.59 7.59 7.59 7.58 7.57 7.57 7.56 7.55 7.50 7.50 7.50 7.48 7.35 7.35 7.34 7.32 7.31 7.31 7.30 7.30 7.26 7.25 7.25 7.25 7.21 7.20 7.19 7.07 7.06 7.05
8.44 8.24 8.24 8.22 8.22 8.22
6.72 6.72 6.72 6.71
0.87 1.01 2.16 1.06 1.93 1.11 1.64 1.07 1.01 0.97 3.17
0.0 9.5 9.0 8.5 8.0 7.5 7.0 6.5 6.0 5.5 5.0 4.5 4.0 3.5 3.0 2.5 2.0 1.5 1.0 0.5 0.0
f1 (ppm)

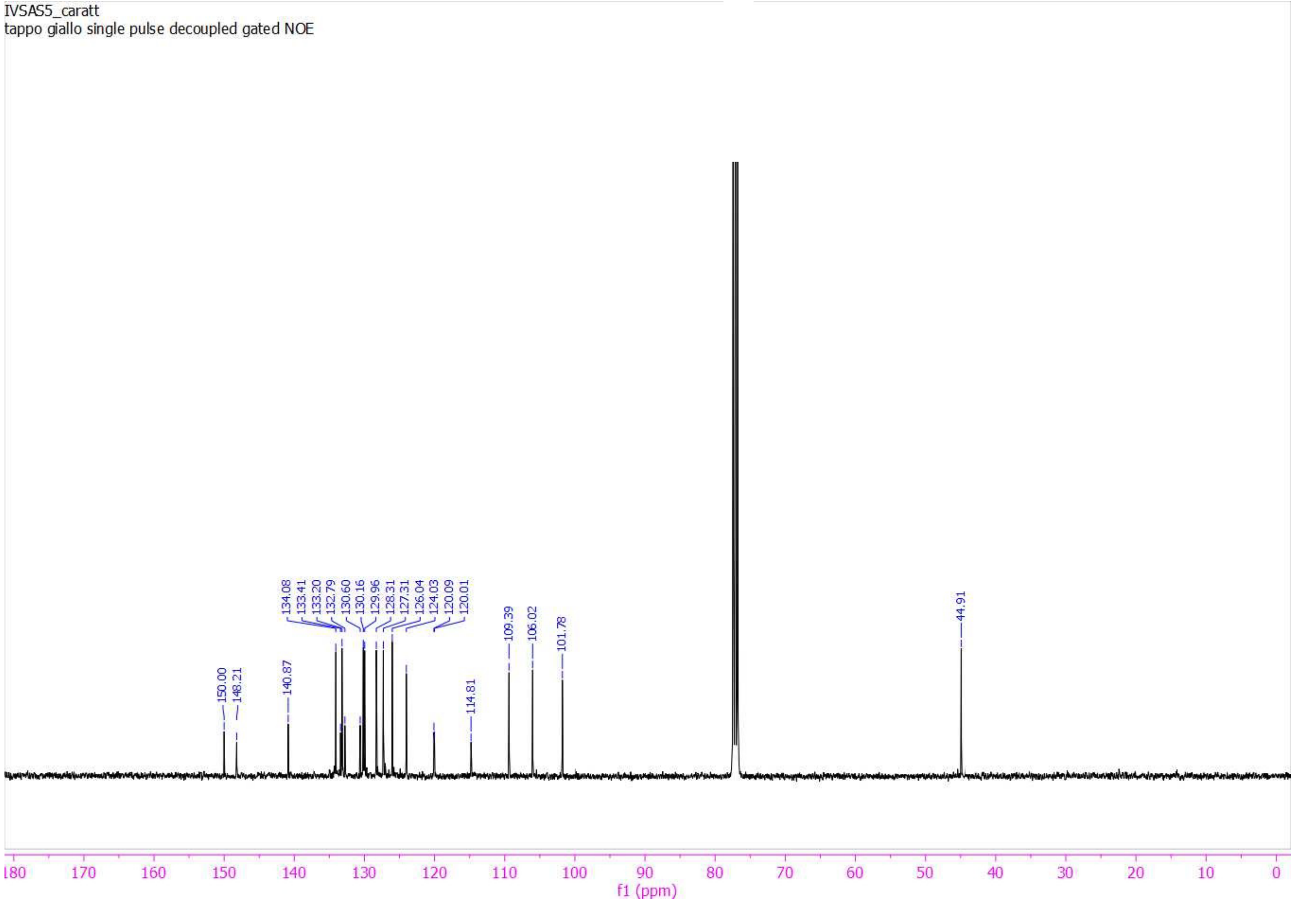
IVSAS5_caratt
tappo giallo single pulse decoupled gated NOE
134.08 133.41 133.20 132.79 130.60 130.16 129.96 128.31 127.31 126.04 124.03 120.09 120.01
150.00 148.21 140.87 114.81 109.39 106.02 101.78 44.91
180 170 160 150 140 130 120 110 100 90 80 70 60 50 40 30 20 10 0
f1 (ppm)

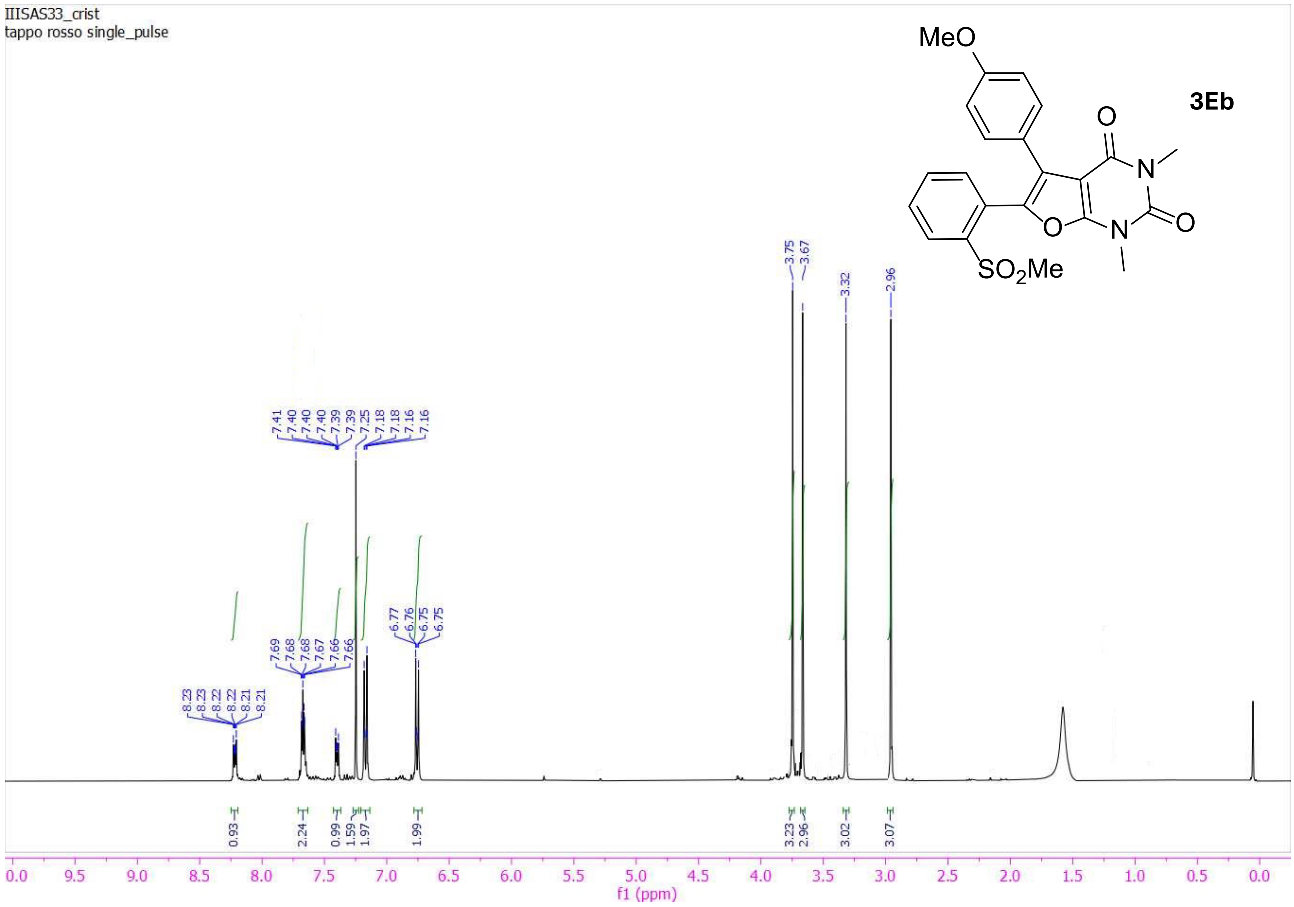
IIISAS33_crist
tappo rosso single_pulse
MeO
O
N
O
N
O
SO2Me
3Eb
8.23 8.23 8.22 8.22 8.21 8.21
7.69 7.68 7.68 7.67 7.66 7.66
7.41 7.40 7.40 7.40 7.39 7.39 7.25 7.18 7.18 7.16 7.16
6.77 6.76 6.75 6.75
3.75 3.67 3.32 2.96
0.93 2.24 0.99 1.59 1.97 1.99 3.23 2.96 3.02 3.07
f1 (ppm)

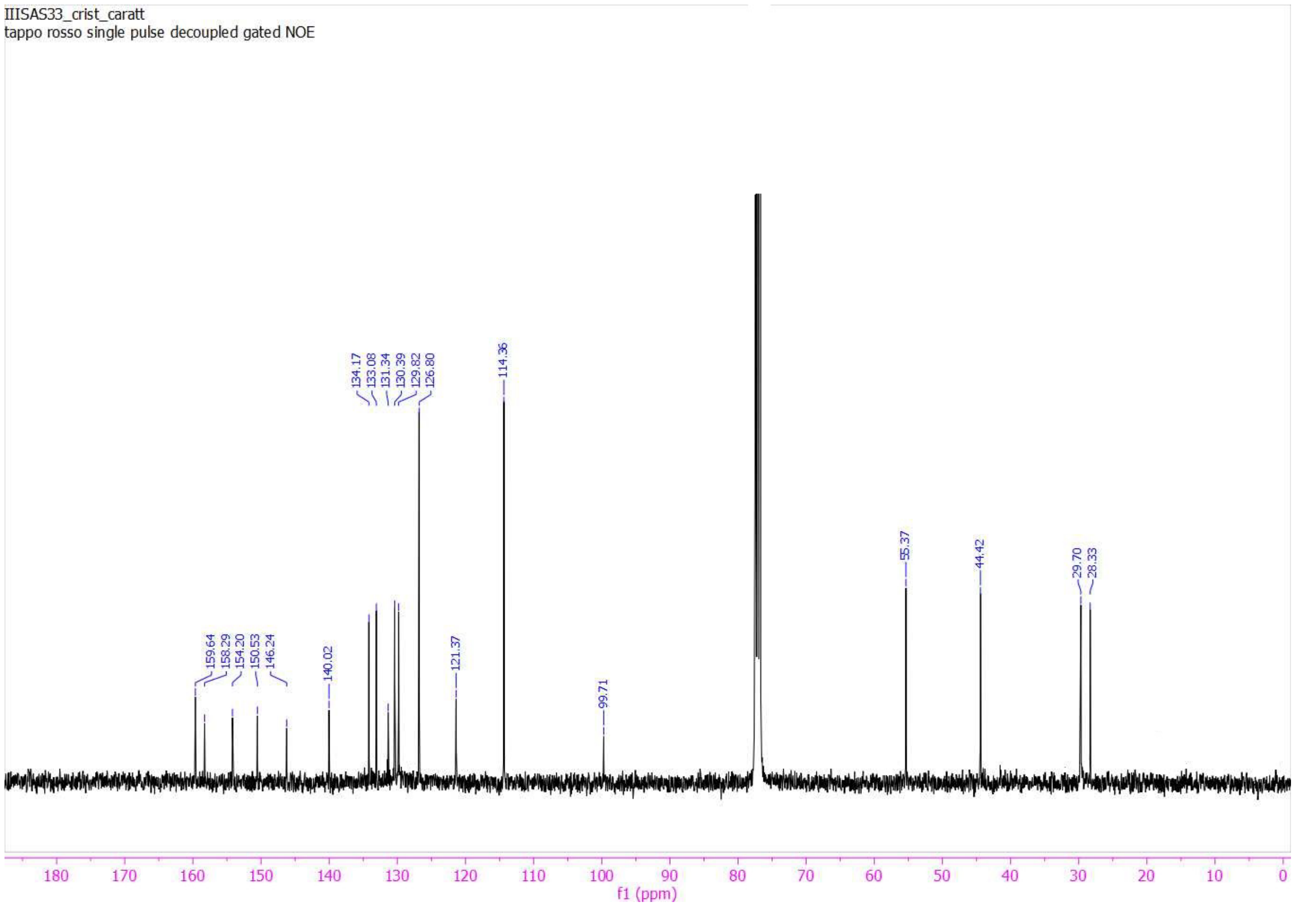
IIISAS33_crist_caratt
tappo rosso single pulse decoupled gated NOE
159.64 158.29 154.20 150.53 146.24 140.02
134.17 133.08 131.34 130.39 129.82 126.80 121.37 114.36 99.71
55.37 44.42 29.70 28.33
f1 (ppm)

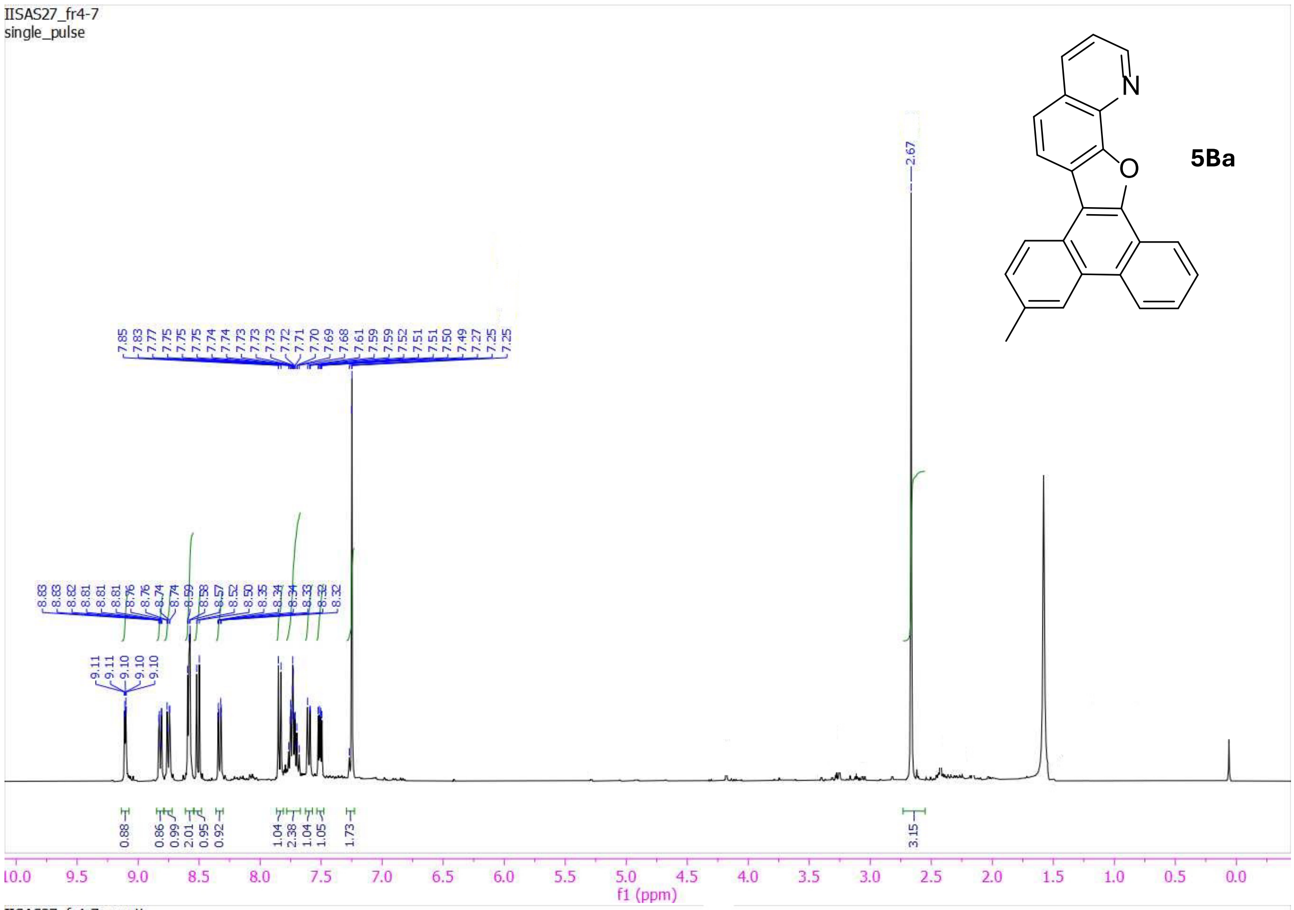
IISAS27_fr4-7
single_pulse
5Ba
N
O
7.85 7.83 7.77 7.75 7.75 7.75 7.74 7.74 7.73 7.73 7.73 7.72 7.71 7.70 7.69 7.68 7.61 7.59 7.59 7.52 7.51 7.51 7.50 7.49 7.27 7.25 7.25
2.67
8.83 8.83 8.82 8.81 8.81 8.81 8.76 8.76 8.74 8.74 8.59 8.58 8.57 8.52 8.50 8.35 8.34 8.34 8.33 8.32 8.32
9.11 9.11 9.10 9.10
0.88 0.86 0.99 2.01 0.95 0.92 1.04 2.38 1.04 1.05 1.73 3.15
f1 (ppm)

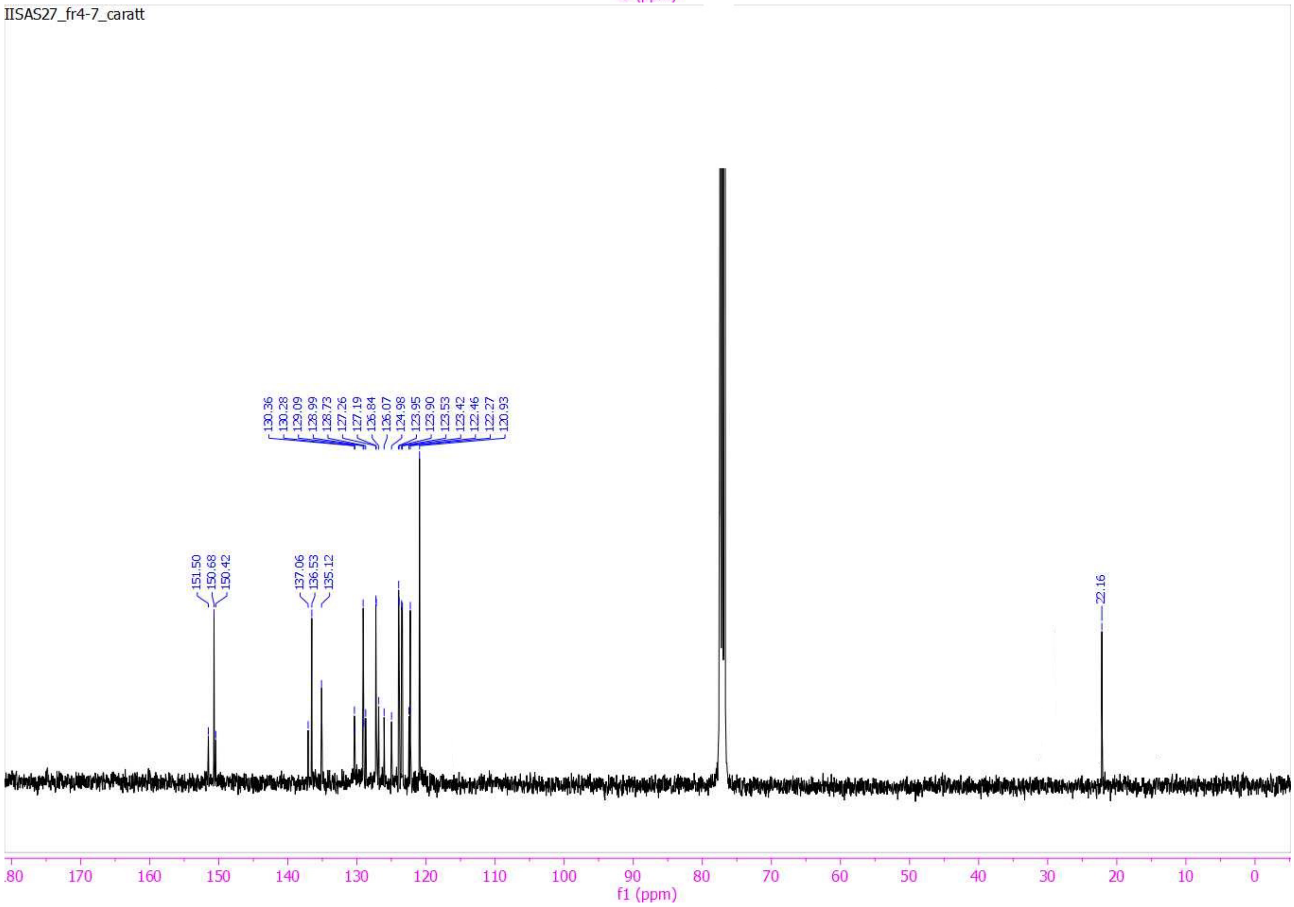
IISAS27_fr4-7_caratt
130.36 130.28 129.09 128.99 128.73 127.26 127.19 126.84 126.07 124.98 123.95 123.90 123.53 123.42 122.46 122.27 120.93
151.50 150.68 150.42
137.06 136.53 135.12
22.16
f1 (ppm)

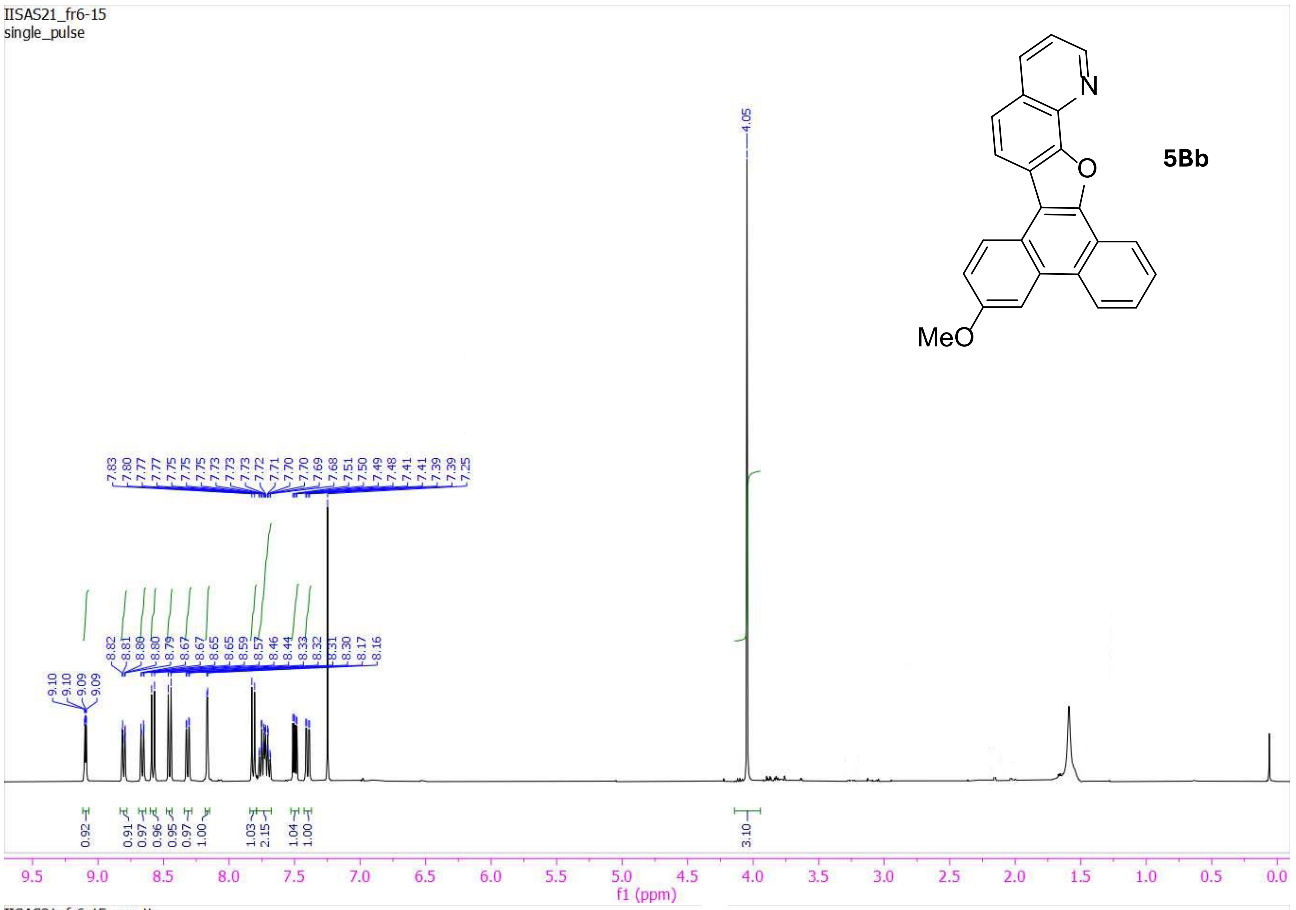
IISAS21_fr6-15
single_pulse
5Bb
N
O
MeO
4.05
7.83 7.80 7.77 7.77 7.75 7.75 7.75 7.73 7.73 7.73 7.72 7.71 7.70 7.70 7.69 7.68 7.51 7.50 7.49 7.48 7.41 7.39 7.39 7.25
9.10 9.10 9.09 9.09
8.82 8.81 8.80 8.80 8.79 8.67 8.65 8.65 8.59 8.57 8.46 8.44 8.33 8.32 8.31 8.30 8.17 8.16
0.92 0.91 0.97 0.96 0.95 0.97 1.00 1.03 2.15 1.04 1.00 3.10
f1 (ppm)

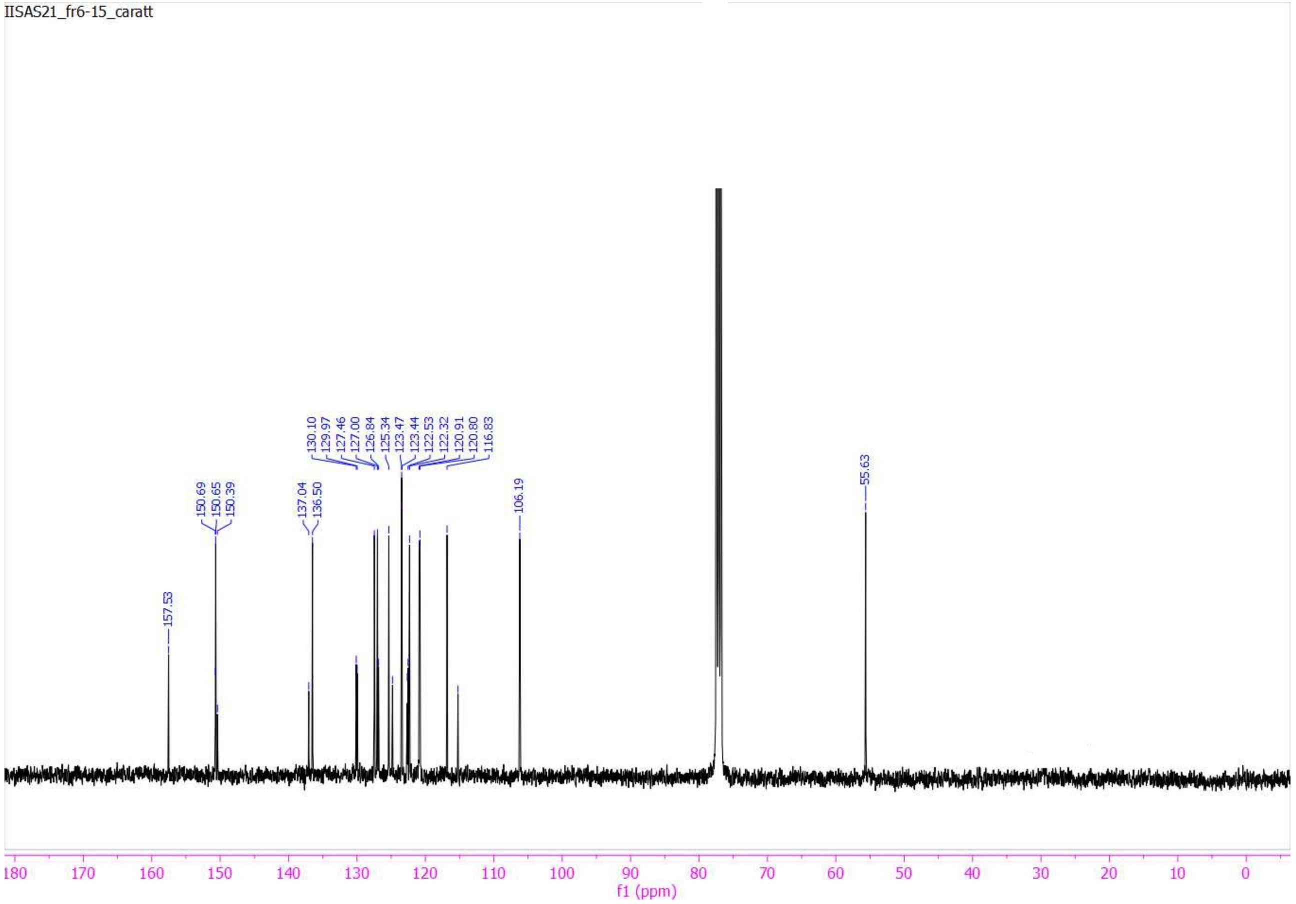
IISAS21_fr6-15_caratt
157.53
150.69 150.65 150.39
137.04 136.50
130.10 129.97 127.46 127.00 126.84 125.34 123.47 123.44 122.53 122.32 120.91 120.80 116.83
106.19
55.63
f1 (ppm)

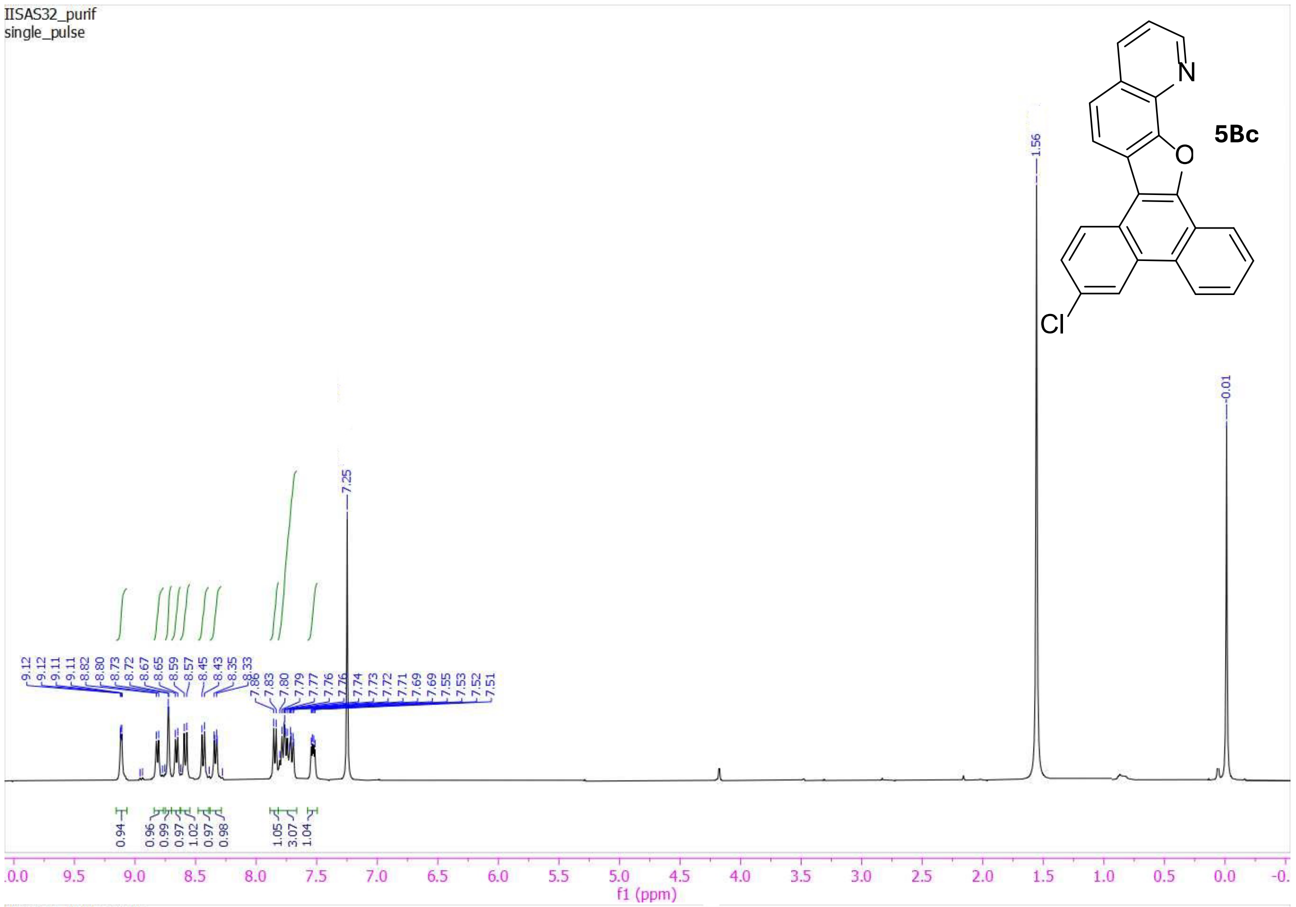
IISAS32_purif
single_pulse
N
O
5Bc
Cl
1.56
0.01
7.25
9.12 9.12 9.11 9.11 8.82 8.80 8.73 8.72 8.67 8.65 8.59 8.57 8.45 8.43 8.35 8.33 7.86 7.83 7.80 7.79 7.77 7.76 7.76 7.74 7.73 7.72 7.71 7.69 7.69 7.55 7.53 7.52 7.51
0.94 0.96 0.99 0.97 1.02 0.97 0.98 1.05 3.07 1.04
f1 (ppm)

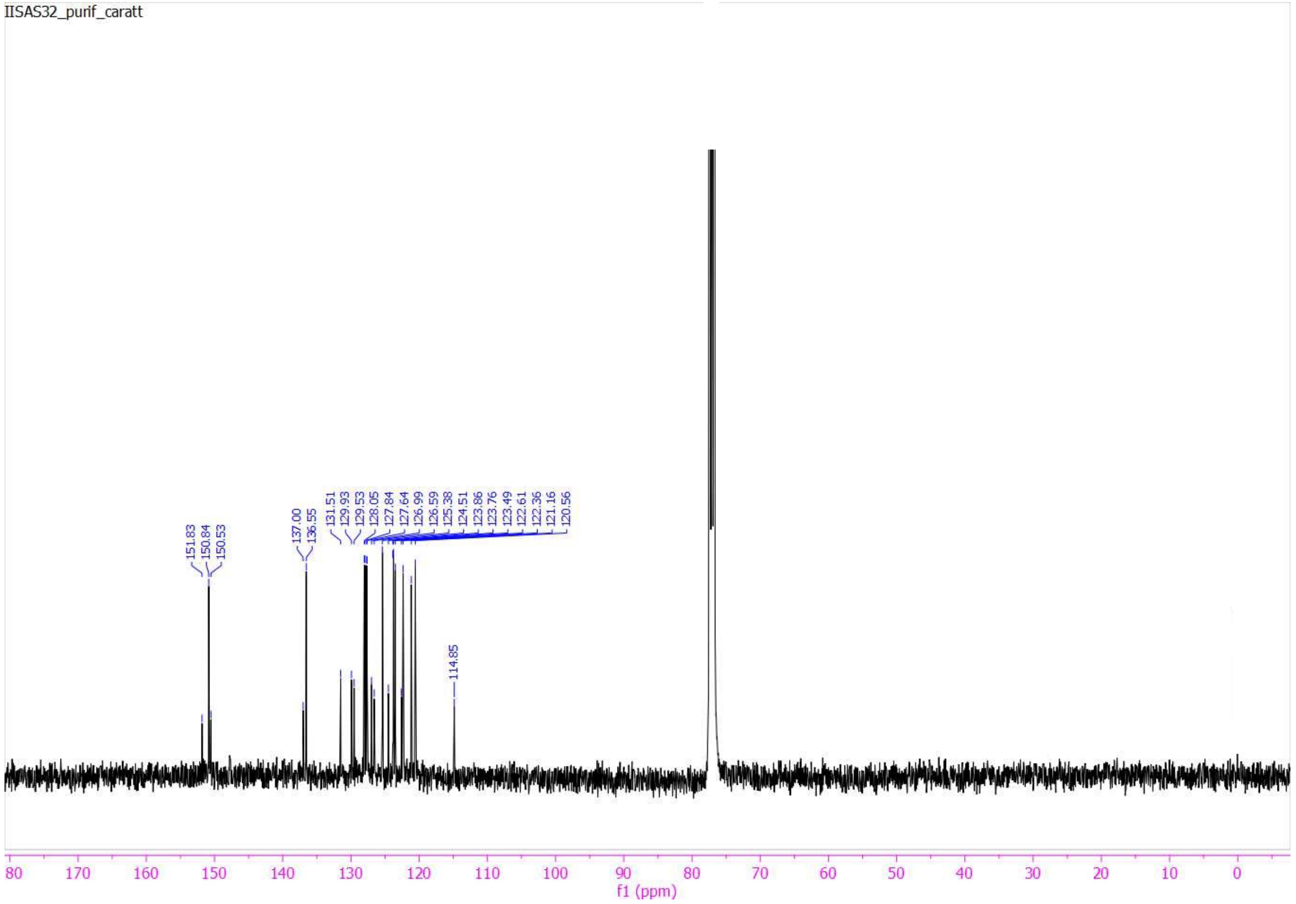
IISAS32_purif_caratt
151.83 150.84 150.53
137.00 136.55
131.51 129.93 129.53 128.05 127.84 127.64 126.99 126.59 125.38 124.51 123.86 123.76 123.49 122.61 122.36 121.16 120.56
114.85
f1 (ppm)

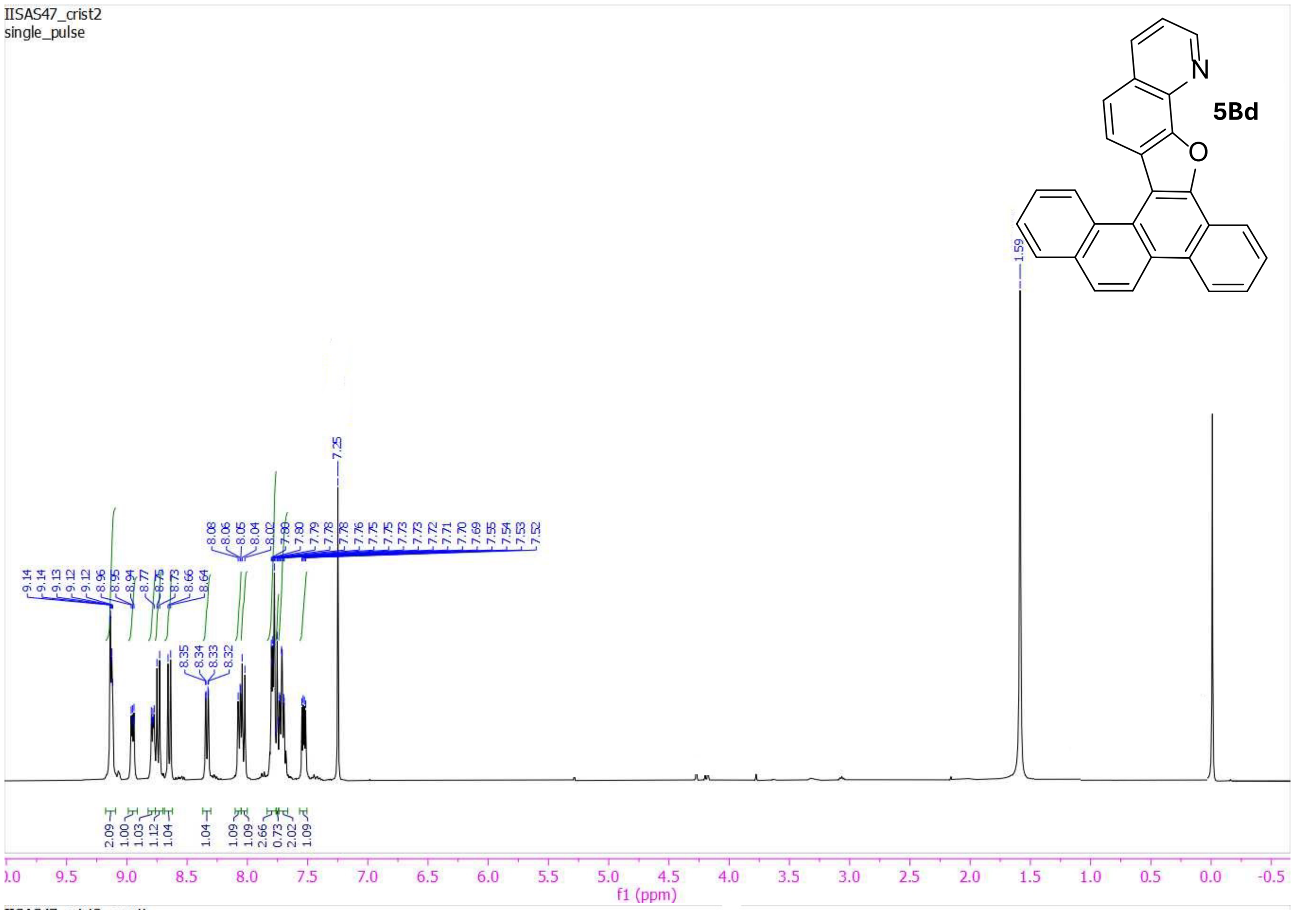
IISAS47_crist2
single_pulse
5Bd
N
O
9.14 9.14 9.13 9.12 9.12 8.96 8.95 8.94 8.77 8.75 8.73 8.66 8.64
8.35 8.34 8.33 8.32
8.08 8.06 8.05 8.04 8.02 7.80 7.80 7.79 7.78 7.78 7.76 7.75 7.75 7.73 7.73 7.72 7.71 7.70 7.69 7.55 7.54 7.53 7.52
7.25
1.59
2.09 1.00 1.03 1.12 1.04 1.04 1.09 1.09 2.66 0.73 2.02 1.09
f1 (ppm)

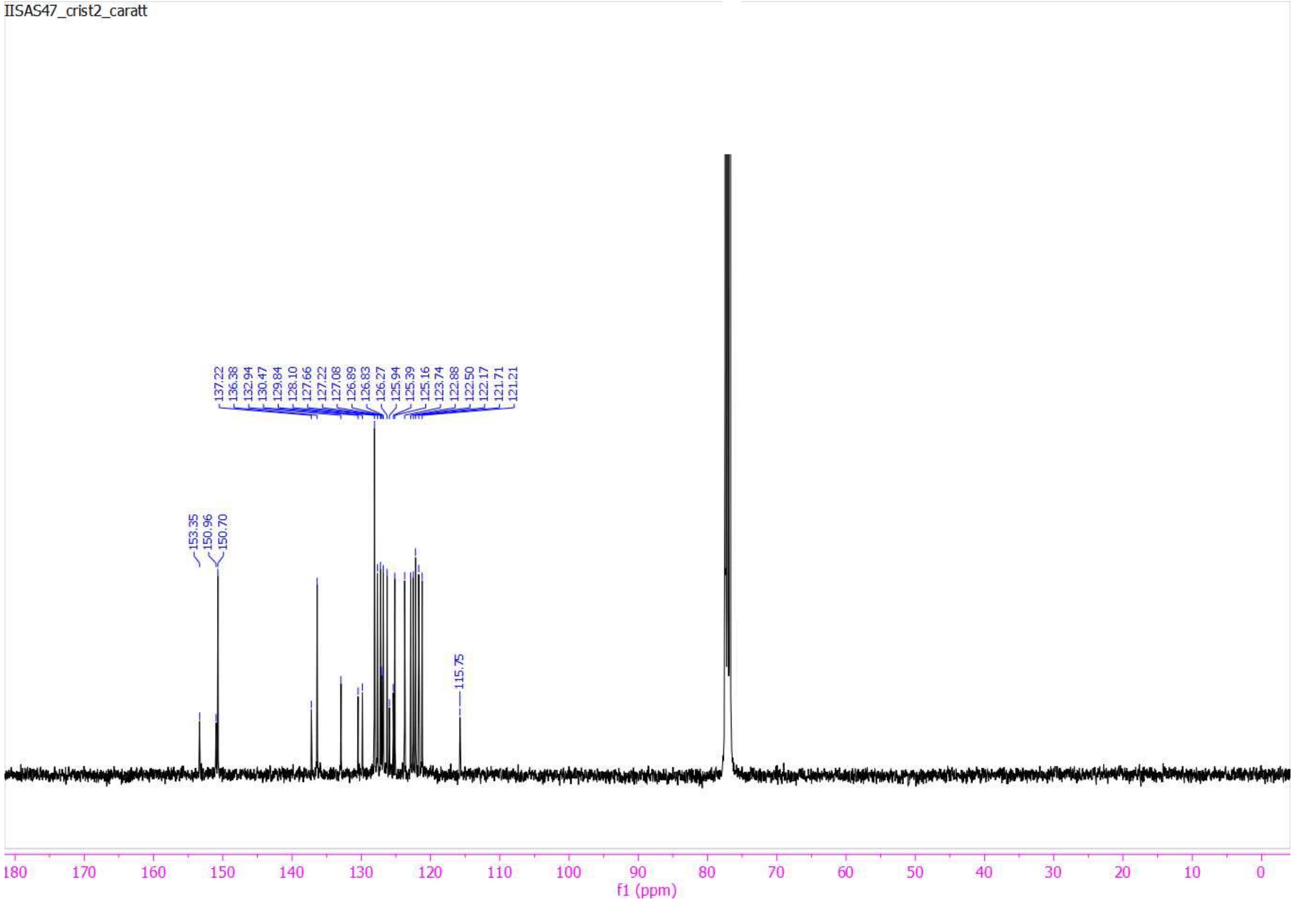
IISAS47_crist2_caratt
153.35 150.96 150.70
137.22 136.38 132.94 130.47 129.84 128.10 127.66 127.22 127.08 126.89 126.83 126.27 125.94 125.39 125.16 123.74 122.88 122.50 122.17 121.71 121.21
115.75
f1 (ppm)

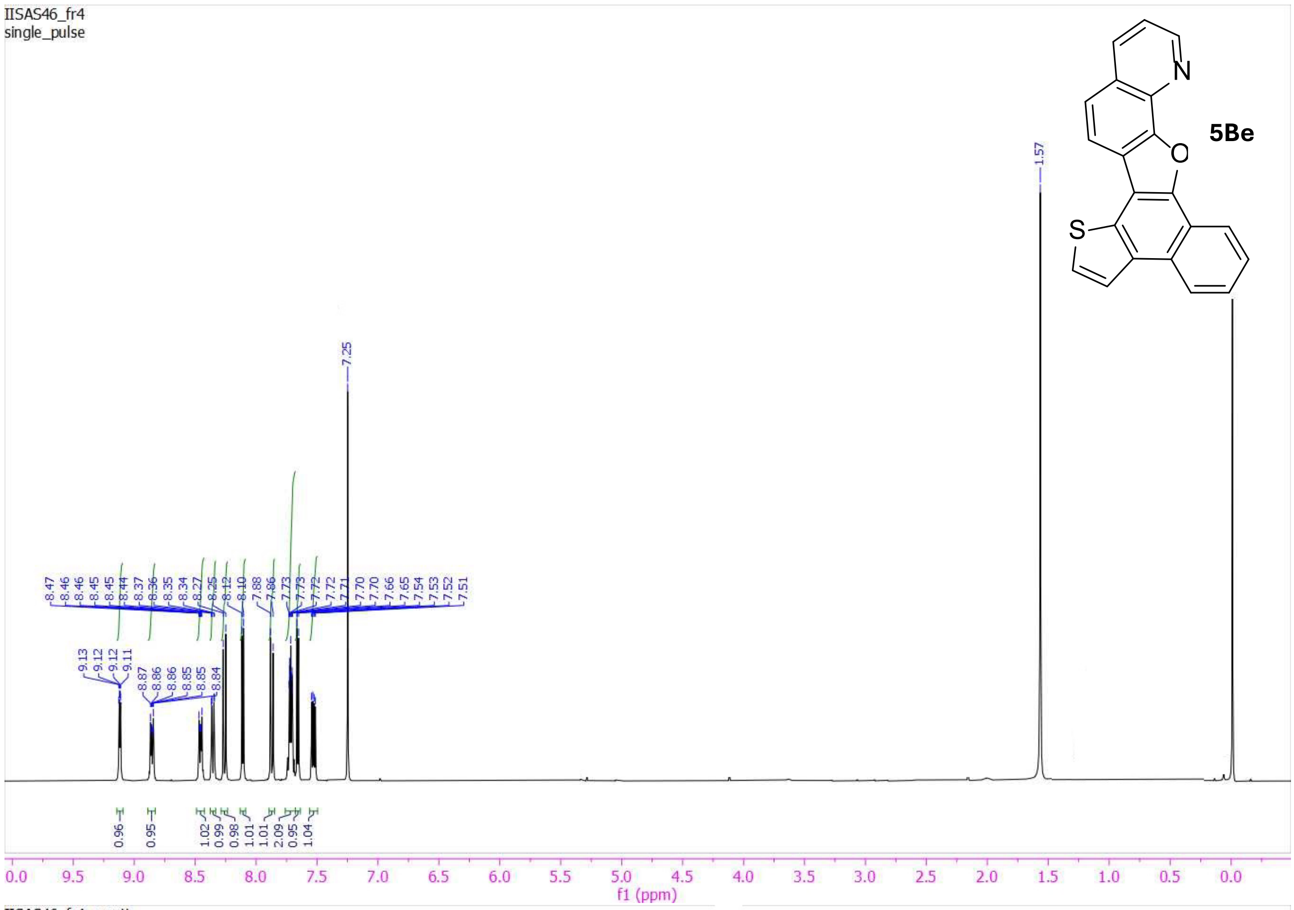
IISAS46_fr4
single_pulse
5Be
9.13 9.12 9.12 9.11
8.87 8.86 8.86 8.85 8.85 8.84
8.47 8.46 8.45 8.45 8.44 8.37 8.36 8.35 8.34 8.27 8.25 8.12 8.10 7.88 7.86 7.73 7.73 7.72 7.72 7.71 7.70 7.70 7.66 7.65 7.54 7.53 7.52 7.51
7.25
1.57
0.96 0.95 1.02 0.99 0.98 1.01 1.01 2.09 0.95 1.04
f1 (ppm)

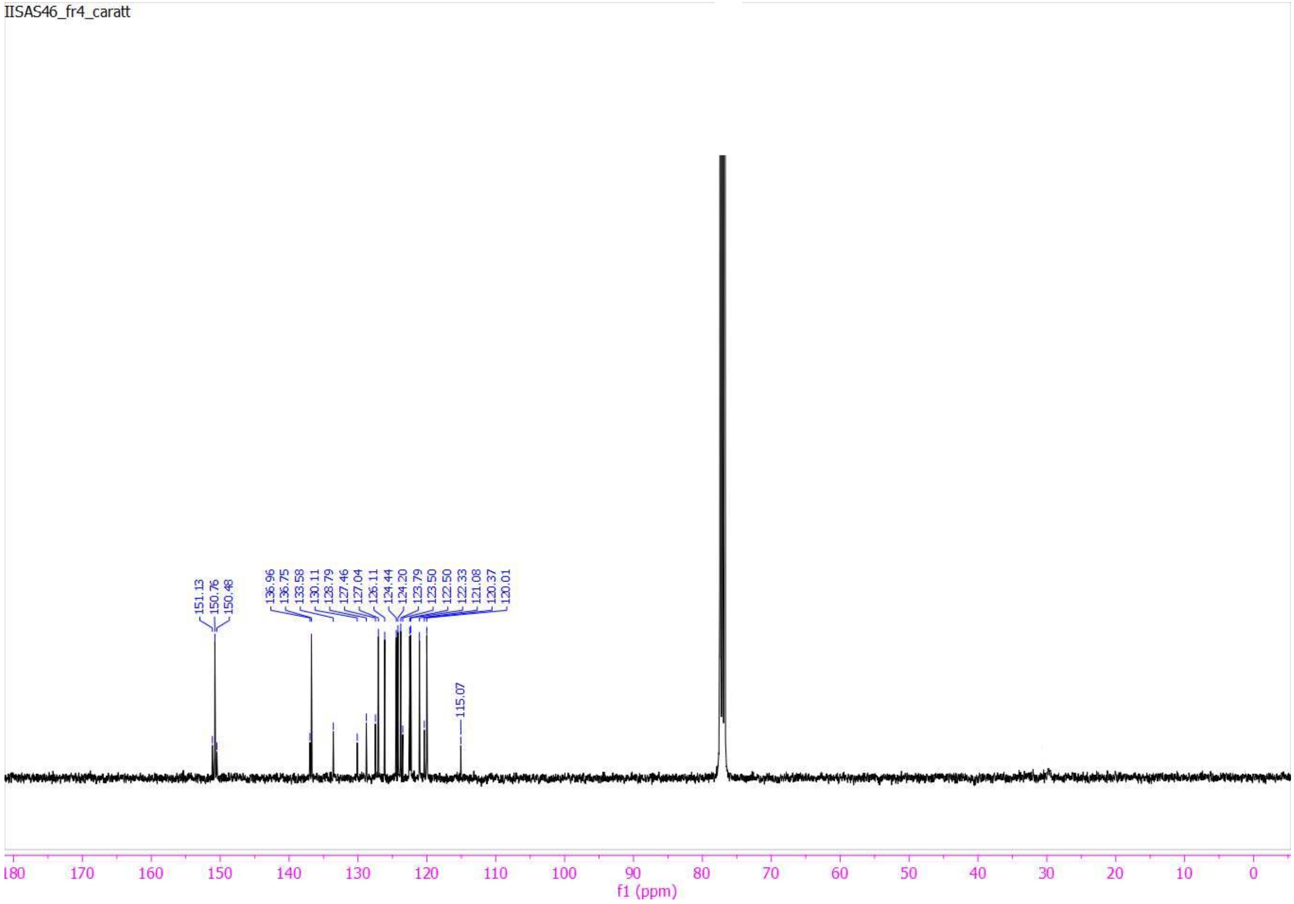
IISAS46_fr4_caratt
151.13 150.76 150.48
136.96 136.75 133.58 130.11 128.79 127.46 127.04 126.11 124.44 124.20 123.79 123.50 122.50 122.33 121.08 120.37 120.01
115.07
f1 (ppm)

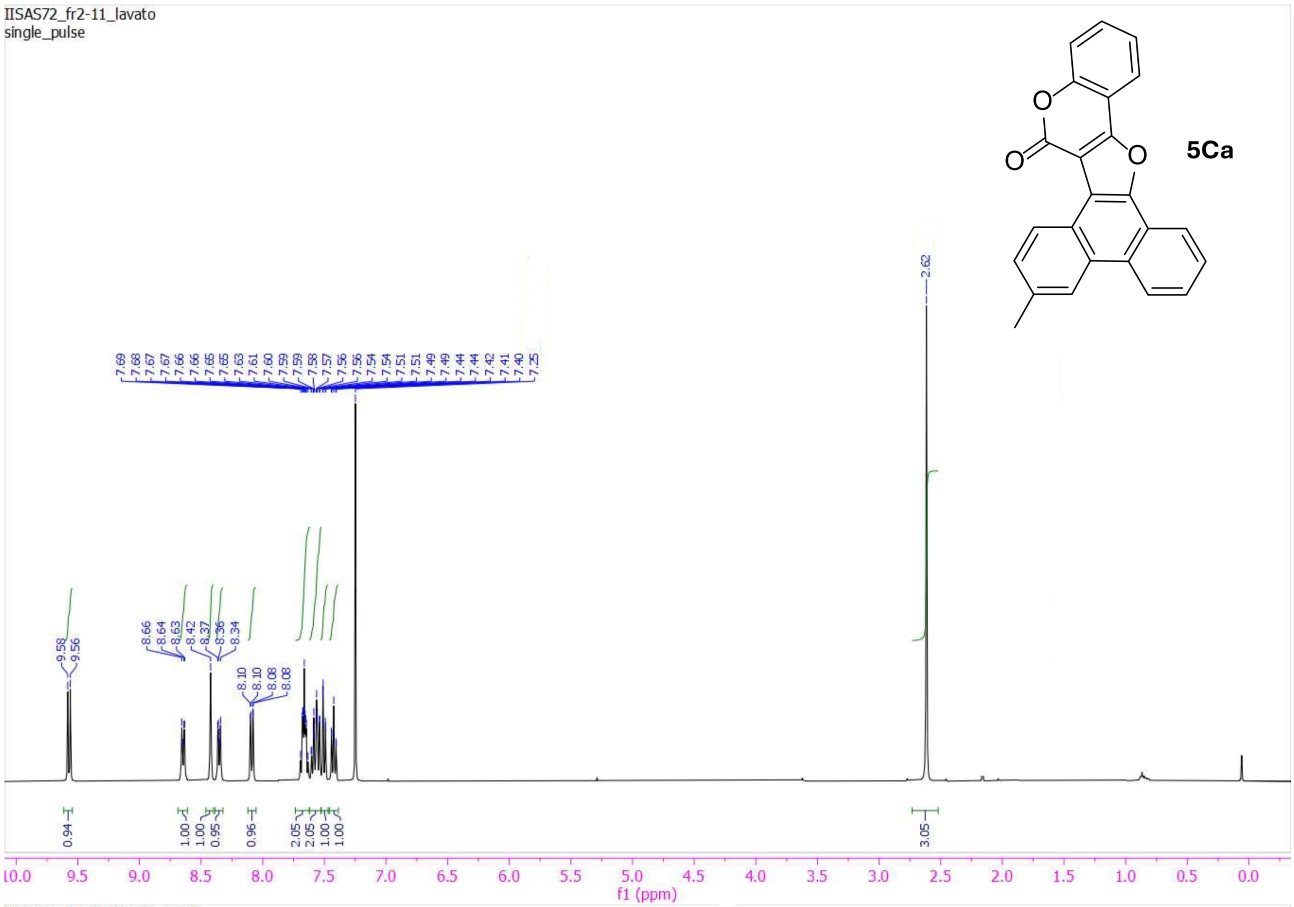
IISAS72_fr2-11_lavato
single_pulse
5Ca
O
O
O
9.58
9.56
8.66
8.64
8.63
8.42
8.37
8.36
8.34
8.10
8.10
8.08
8.08
7.69
7.68
7.67
7.67
7.66
7.65
7.65
7.63
7.61
7.60
7.59
7.59
7.58
7.57
7.56
7.56
7.54
7.54
7.51
7.51
7.49
7.49
7.44
7.42
7.41
7.40
7.25
2.62
0.94
1.00
1.00
0.95
0.96
2.05
2.05
1.00
1.00
3.05
f1 (ppm)

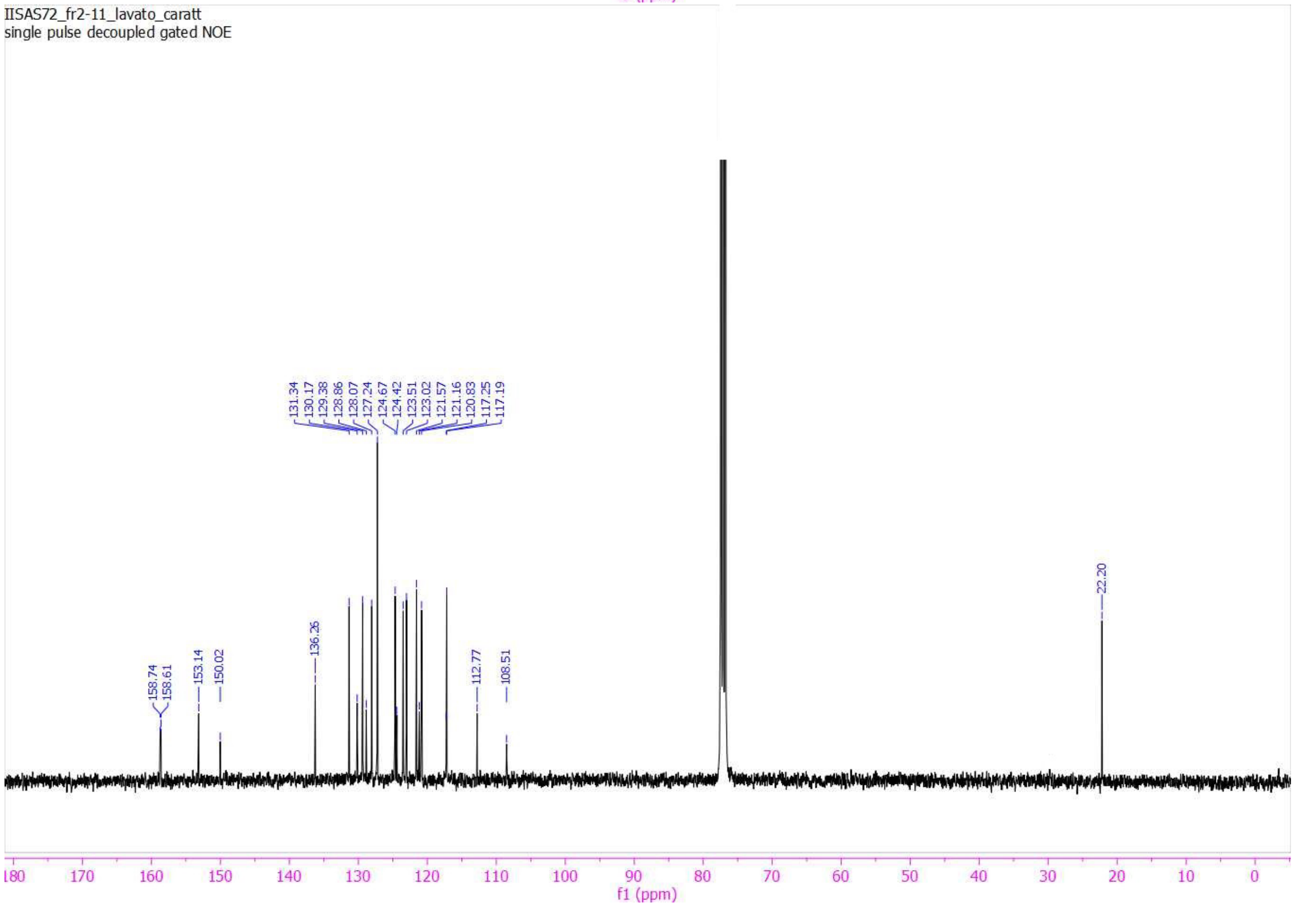
IISAS72_fr2-11_lavato_caratt
single pulse decoupled gated NOE
158.74
158.61
153.14
150.02
136.26
131.34
130.17
129.38
128.86
128.07
127.24
124.67
124.42
123.51
123.02
121.57
121.16
120.83
117.25
117.19
112.77
108.51
22.20
f1 (ppm)

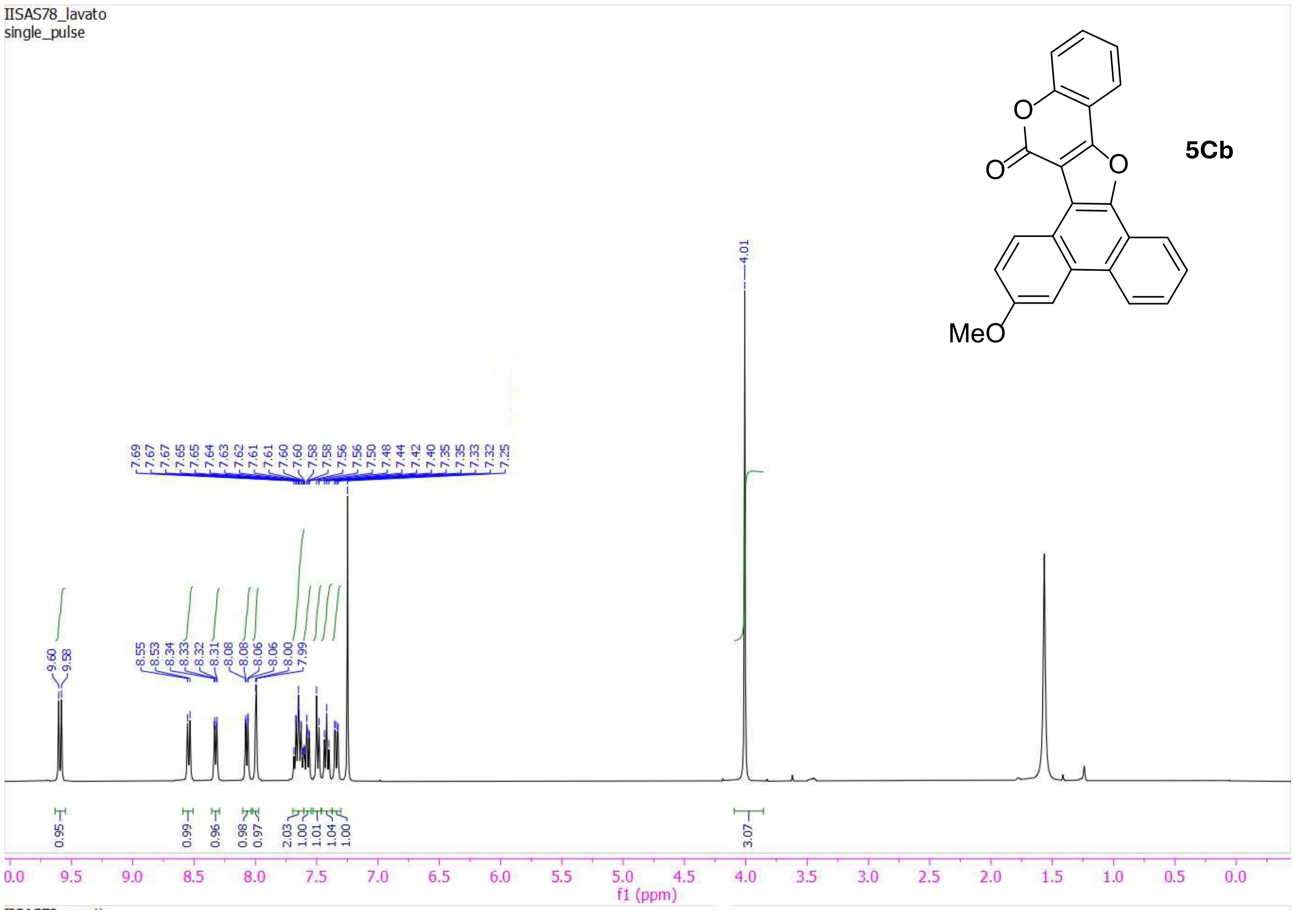
IISAS78_lavato
single_pulse
5Cb
MeO
O
O
O
O
7.69 7.67 7.67 7.65 7.65 7.64 7.63 7.62 7.61 7.61 7.60 7.60 7.58 7.58 7.56 7.56 7.50 7.48 7.44 7.42 7.40 7.35 7.35 7.33 7.32 7.25
9.60 9.58
8.55 8.53 8.34 8.33 8.32 8.31 8.08 8.08 8.06 8.06 8.00 7.99
4.01
0.95
0.99
0.96
0.98
0.97
2.03 1.00 1.01 1.04 1.00
3.07
f1 (ppm)

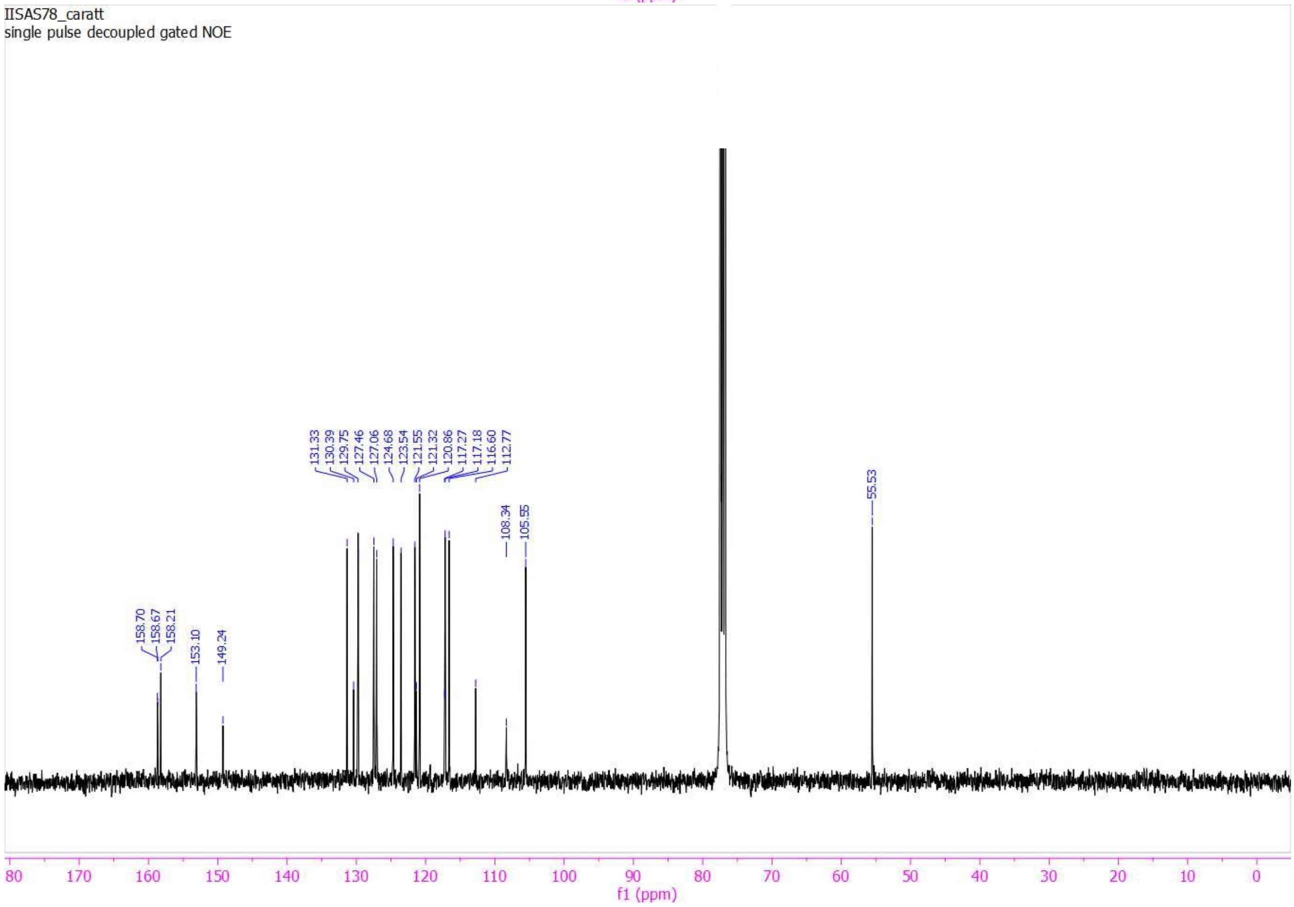
IISAS78_caratt
single pulse decoupled gated NOE
158.70 158.67 158.21
153.10
149.24
131.33 130.39 129.75 127.46 127.06 124.68 123.54 121.55 121.32 120.86 117.27 117.18 116.60 112.77
108.34
105.55
55.53
f1 (ppm)
